# Hypoxia alters vulnerability to capture and the potential for trait-based selection in a scaled-down trawl fishery

**DOI:** 10.1093/conphys/coz082

**Published:** 2019-11-27

**Authors:** Davide Thambithurai, Amelie Crespel, Tommy Norin, Anita Rácz, Jan Lindström, Kevin J Parsons, Shaun S Killen

**Affiliations:** 1 Institute of Biodiversity, Animal Health and Comparative Medicine, University of Glasgow, Graham Kerr Building, Glasgow G12 8QQ, UK; 2 DTU Aqua: National Institute of Aquatic Resources, Technical University of Denmark, Kemitorvet, Building 202, 2800 Kgs. Lyngby, Denmark; 3 Department of Genetics, Eötvös Loránd University, Pázmány P.s. 1C, H-1117 Budapest, Hungary

**Keywords:** hypoxia, fisheries-induced evolution, swimming performance, environmental stress, trawling

## Abstract

Lay summary

Selective harvest of wild organisms by humans can influence the evolution of plants and animals, and fishing is recognized as a particularly strong driver of this process. Importantly, these effects occur alongside environmental change. Here we show that aquatic hypoxia can alter which individuals within a fish population are vulnerable to capture by trawling, potentially altering the selection and evolutionary effects stemming from commercial fisheries.

## Introduction

Harvest of animals from wild populations is recognized as a driver of phenotypic and genetic change (Allendorf and Hard, 2009; Darimont *et al.*, 2015; Hendry *et al.*, 2017). Individuals of a certain size (Conover *et al.*, 2009), behavioural type (Diaz Pauli and Sih, 2017) or those displaying specific morphological characteristics (Jachmann *et al.*, 1995) have the potential to be removed preferentially from a population through exploitation. Significant effort has been directed towards understanding directional selection within commercially exploited fish species and its capacity to cause evolutionary change (Stockwell *et al.*, 2003; Nusslé *et al.*, 2016). For fisheries-associated selection to have evolutionary consequences, traits must show heritable and repeatable variation that is consistently targeted by selection (Law, 2000). Importantly, trait variation, repeatability and heritability can change across environments, as trait values are altered by phenotypic plasticity (Melbinger and Vergassola, 2015; Killen *et al.*, 2016; Norin *et al.*, 2016; Campbell *et al.*, 2017). This leads to the possibility that harvest-associated selection may be amplified or weakened under specific environmental conditions (Killen *et al.*, 2013; Hollins *et al.*, 2018).

Oxygen concentrations in aquatic environments have been in decline for the past 50 years and are projected to continue to fall over the 21st century (Schmidtko *et al.*, 2017; Breitburg *et al.*, 2018). Over the past several decades, anthropogenic carbon emissions and nutrient run-offs have acted in synergy to exacerbate natural temporal and spatial fluctuations in the oxygen content of aquatic environments (Diaz and Rosenberg, 2008). Reduced oxygen availability (environmental hypoxia) is a major environmental limitation driving morphological, physiological and behavioural adaptation in aquatic organisms (Mandic *et al.*, 2009; Richards, 2009; Stramma *et al.*, 2010; Stoffels, 2015), even reducing survival or causing migration if sufficiently severe (Breitburg, 2002). However, before reaching lethal levels, hypoxia can have important sublethal effects on spontaneous activity, growth, distribution patterns and physiological performance. Typically, the first response of fish to hypoxia is increased gill ventilation (Breitburg, 2002), quickly accompanied by behavioural avoidance. However, the point at which behavioural changes occur varies widely among species and habitats (Diaz and Breitburg, 2009; Richards, 2009). In some cases, adverse physiological effects of hypoxia may occur prior to the onset of avoidance. In fishes, for example, maximum aerobic metabolic rate (MMR) is generally reduced during exposure to hypoxia, often with corresponding reductions in locomotor performance (Norin and Clark, 2016), while the standard metabolic rate (SMR)—the rate of energy turnover required to support essential maintenance functions in ectotherms (Chabot *et al.*, 2016)—remains constant as long as the level of oxygen availability remains above the animal’s critical oxygen tension (*P*_crit_), at which point the rate of oxygen uptake becomes limited by the environment and decreases (Richards, 2009). Under hypoxic conditions that restrict aerobic metabolism, fish may be forced to prioritize specific oxygen-consuming physiological processes. Voluntary swimming activity, for instance, may be limited under hypoxia (Schurmann and Steffensen, 1994; Kraskura and Nelson, 2018), as aerobic scope will be reduced (Richards, 2009). Importantly, reductions in swimming capacity with increasing hypoxia have been shown in several fish species of commercial importance, including cod *Gadus morhua* (Schurmann and Steffensen, 1994), dogfish *Scyliorhinus canicula* (Metcalfe and Butler, 1984) and sole *Solea solea* (Dalla Via *et al.*, 1998).

Despite the fact that fishing, and potentially fisheries-induced evolution, is occurring alongside rapid changes in environmental conditions driven by climate change (Schmidtko *et al.*, 2017), little is known about possible synergistic interactions between fishery selection and climate-related stressors (Kolding *et al.*, 2008; Diaz Pauli *et al.*, 2017; Clark *et al.*, 2017; Hollins *et al.*, 2018). Highly productive aquatic habitats are often also associated with zones of hypoxia, as the coastal upwellings and stratification patterns that lead to influxes of nutrients also give rise to hypoxic events. The result is that fish tend to congregate on the edges of hypoxic fronts, owing in part to increased foraging potential (Breitburg *et al.*, 2018). This mechanism leads to so-called habitat compression, increasing densities of fish (Prince and Goodyear, 2006) and rendering them more vulnerable to exploitation (Stramma *et al.*, 2010). Although fishers typically avoid areas of severe or prolonged hypoxia (Breitburg, 2002), areas of moderate or temporary hypoxia are fished (Gray, 1992), and there is evidence to suggest that many fishes transiently occupy hypoxic areas throughout their lifetime (Thomas and Rahman, 2012; Altenritter *et al.*, 2018). The fact that fishing can occur in hypoxic areas is perhaps best reflected by the reported surges in capture where fishing and low oxygen co-occur (Gray, 1992; Breitburg, 2002). A possible explanation for this increase in catch is the constraining effect of hypoxia on fish locomotor or sensory physiology (Kraskura and Nelson, 2018), which may inhibit escape responses. Given that active fishing gears (e.g. trawls) pursue fish, it is possible that escape relies on the locomotor performance of the fish (Main and Sangster, 1983). Selection can only function on traits that show repeatable within-population variation (Wilson, 2018). Both trait variance and repeatability—the latter defined as the proportion of observed variance attributable to consistent among individual differences—are known to change in response to environmental conditions (Gibson and Dworkin, 2004; Norin *et al.*, 2016). An important mechanism that remains to be understood is how hypoxia, which may increase under a warming climate, can modulate trait variance and repeatability in such a way to influence selection.

**Figure 1:**
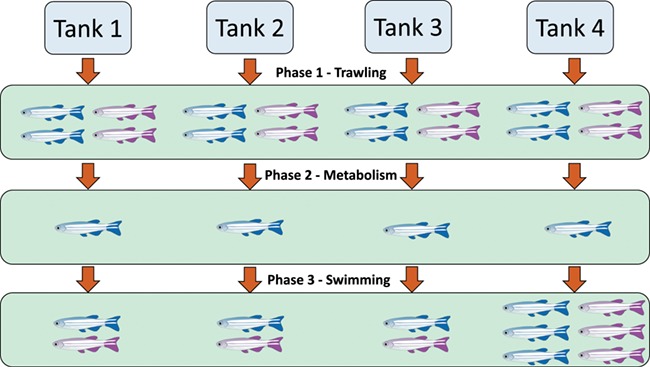
Flow chart of experimental design showing the three distinct phases of the study. Each fish represents one replicate; blue denotes normoxic conditions and purple hypoxic conditions.

Using zebrafish (*Danio rerio*) as a model organism, we conducted trawl fishing simulations to investigate how acute exposure to hypoxia affects swimming performance and vulnerability to capture. By using small-scale fishery simulations within a swim tunnel, we were able to concentrate on the crucial phase of capture that occurs at the mouth of a trawl net while maintaining fine control over oxygen availability, something impossible to achieve in a field setting (Diaz Pauli *et al.*, 2015; Thambithurai *et al.*, 2018). We exposed fish to a 35% air saturated water hypoxia level, which is less hypoxic than the level thought to induce emigration in a number of species (Breitburg, 2002), but sufficiently hypoxic to induce changes to locomotor physiology. We assessed swimming performance of individual fish by measuring their critical swimming speed (*U*_crit_), and we measured two key metabolic traits (SMR and MMR) under normoxia to understand the role that these play in vulnerability to capture. Repeatability of both swimming and capture within, as well as between, different levels of hypoxia was quantified for each fish tested. This approach will inform and help to refine lines of inquiry at larger spatial scales. We hypothesized that the constraining effect of hypoxia on MMR would increase capture vulnerability by limiting aerobic swimming capacity and that the variance and repeatability of swimming capacity and capture would decrease in hypoxia, thus leading to reduced evolutionary potential.

## Methods

### Study organism

In spring 2017, a stock population of adult zebrafish (mean ± SD body mass = 0.55 ± 0.10 g; mean ± SD standard length = 32.60 ± 2.06 mm) ~6 months of age were sourced from rearing ponds in Malaysia (JMC Aquatics, Sheffield). On arrival, and between experiments, fish were housed in 300 L stock tanks held under a 13:11 h light:dark photoperiod and supplied with dechlorinated freshwater that was continuously filtered (Hagen Fluvial Fx5 external filter and V^2^ecton 600 UV sterilizer) and maintained at 26°C. Fish density was kept at a maximum of five fish per litre. Plastic plants and fine grade coral sand were also provided as enrichment. The fish were fed *ad libitum* twice daily with a combination of commercial feed (TetraMin Tropical Flakes, ZM small granular) and live 48 h hatched *Artemia nauplii* (Sanders Great Salt Lake Artemia Cysts). A month before the start of experiments (Summer 2017) the experimental group of fish was haphazardly caught by dip net from the stock population and each individual was tagged using visual implant elastomer (North West Marine Technology, Shaw Island, WA, USA) in four dorsal locations with a unique code identifier. After tagging, fish were split into four sub-population, placed into four identical 300 L aquaria and maintained under the same husbandry conditions as before.

### Experiment summary

Experiments were carried out across three distinct phases: (i) trawl vulnerability assays; (ii) measurements of metabolic rates; and (iii) measurements of swimming performance. The initial population (*n* = 146) was randomly split into four replicate holding tanks; these fish were kept in their own tanks for the remainder of the study. To quantify fishing vulnerability, each fish was tested for vulnerability to trawling four times during the first phase of the experiment (16 days), twice at normoxia and twice at acute hypoxia (Fig. 1). In the second phase of the experiment, we measured the metabolic rates of all fish under normoxic conditions. Lastly, to examine any links between swimming performance and trawling vulnerability, we quantified *U*_crit_ under both normoxia and hypoxia; fish from tank four were tested three times over a week under each condition, while the fish in tanks one to three were measured once under each condition. The repeatability and variance of vulnerability to trawling (Phase 1) was quantified across the whole population (*n* = 146), while repeatability of swimming performance (Phase 3) was calculated for individuals that were tested three times during the *U*_crit_ assay (*n* = 36).

### Trawling simulations

Trawling simulations were performed in a temperature-regulated (26°C) 90 L Steffensen-type swim tunnel (Loligo Systems, Viborg, Denmark) filled with dechlorinated freshwater to a depth of 20 cm. Fish were trialled in the working section of the flume measuring 66 × 20 × 20 cm and covered with a transparent lid. A false bottom made of dense foam (3 cm high) was fixed to the working section to allow the installation of the miniature trawl net used in the trials. The trawl was a downscaled model of a full-size commercial demersal trawl (manufactured by the Marine Institute, Memorial University of Newfoundland, St. John’s, NL, Canada) and was 30 cm long (9 mm stretched mesh at mouth tapering to 1 mm at cod-end) with a mouth measuring 20 × 17 cm and a footrope measuring 35 cm. To maintain rigidity at the trawl mouth, the net was fixed onto a stainless-steel frame and inserted into the foam using two cylindrical plastic inserts. The net covered the whole channel with exception to two escape holes at the top of the trawl frame each measuring 3 × 3 cm. A wedge-shaped area that retained captured fish (cod-end), made from transparent acrylic sheeting, was inserted at the back of the working section of the flume and attached to the back of the net. This section allowed monitoring of fish upon capture and minimized net impingement and stress. To prevent disturbance from observers, black plastic sheeting was hung around the flume. An acclimation area was also created by cutting a hole into the test-lid chamber and sliding in a vertical mesh screen that served to isolate fish from the fishing set-up prior to the start of the trials (Supplementary Materials 1). To track fish and monitor their interactions with the trawl, we used two cameras: the first (Logitech HD Webcam c920; Logitech Europe S.A., Lausanne, Switzerland) was placed directly above the acclimation area/mouth of the net, while the second (GoPro Hero 4; San Mateo, California, USA) was placed above the retaining area to monitor fish after capture. This set-up allowed the recording of an accurate time stamp for each capture event.

Fish were fasted a minimum of 24 h before each trawling trial. Fish were generally tested in groups of 10, although on some occasions group size was reduced to a minimum of seven fish. The fish were placed in the working area of the flume with a separator dividing the net area from the acclimation area and allowed to acclimate at a water speed of 4 cm s^−1^. At this speed, fish orientated against the current and swam steadily. During the acclimation period of the hypoxic trials, the oxygen concentration within the flume was gradually decreased by bubbling nitrogen into the water instead of air. Constant monitoring and regulation of oxygen levels at the desired level was done by an Oxy-Reg controller (Loligo Systems, Viborg, Denmark) with a solenoid gas valve connected to a nitrogen cylinder and three ceramic (micro-bubble) air stones placed in the flume. Hypoxia-acclimation periods were based on the length of time it took for the water in the flume to reach the required level for the hypoxic treatment (35% air saturation). For consistency, we allowed approximately the same acclimation period across hypoxic treatments and normoxic controls (hypoxia: mean ± SD = 75 ± 38 min; normoxia: 79 ± 13 min). Following the acclimation period, the divider separating the acclimation area from the fishing area was removed and the water speed was gradually increased (over 60 s) to ~47 cm s^−1^; this speed was maintained for the following 9 min. Each fish was tested once a day and allowed a minimum of 24 h before the next test. Following the first hypoxic trial of the day, and prior to each subsequent trial, oxygen levels were brought back to normoxia by bubbling air into the flume. At the end of each test day the flume was drained and refilled with fresh dechlorinated water.

Two metrics were used to quantify fishing vulnerability for each replicate: (i) capture was quantified for each fish as a binary response variable (1 = captured, fish entered the cod-end; 0 = not captured, fish did not enter the cod-end) and (ii) time spent in cod-end (T*_net_*) in seconds was quantified for each fish caught and converted to proportion (time fish spent in net/time of trial). During trials, fish which entered the cod-end were considered captured, and no fish were observed escaping the cod-end after being caught.

### Respirometry

Approximately 1 month following trawling trials, metabolic rates (SMR and MMR) were estimated for each fish from measurements of oxygen uptake rates using intermittent-flow respirometry (Steffensen, 1989; Svendsen *et al*., 2016). Fish were fasted for at least 24 h prior to respirometry trials. Individual fish were first placed in the working area of a 30 L swim tunnel (Loligo Systems, Viborg, Denmark) at a water velocity of ~4 cm s^−1^ and allowed to settle for 10 s. Water velocity was then gradually increased until the fish displayed burst-coast swimming (Tudorache *et al*., 2013); at this point, water velocity was maintained until the fish tired and came into contact with the rear screen of the working area. Upon contact, fish were mechanically stimulated to encourage continued swimming. Following a third contact, fish were immediately removed and placed in 18 ml respirometry chambers to estimate MMR after exhaustive exercise. Maximum metabolic rate was estimated from the first complete measurement phase, for which the slope of the decrease in oxygen over time was divided into 2-min intervals, and the steepest of these slopes was used to calculate MMR (Killen *et al*., 2017). A total of 16 fish were measured daily. Water oxygen content inside the respirometry chambers was quantified every 2 s using FireStingO_2_ optical oxygen meters and associated dipping probe sensors (PyroScience GmbH, Aachen, Germany) inserted into probe holders in an in-line recirculation loop of gas-tight tubing. Respirometry chambers and probes were placed in a temperature-regulated water bath (mean ± SD = 26.6 ± 0.24°C) and were shielded from disturbance using an opaque plastic blind. A peristaltic pump in the in-line recirculation loop was used to achieve water mixing within the respirometry chambers and pass water past the oxygen probes. Every 9 min, an automated flush pump would switch on for 2 min to flush the respirometry chambers with fresh and fully aerated water. At the end of each flushing period, the respirometry chambers would be functionally sealed, allowing the decrease in oxygen to be measured, and the rate of oxygen uptake (mg O_2_ h^−1^) to be calculated as the slope of the decrease in oxygen concentration over time (mg O_2_ L^−1^ h^−1^) multiplied by the volume of the respirometry chamber (0.018 L) and associated tubing after subtracting fish volume (assuming a fish density of 1 g ml^−1^). To correct for microbial respiration, blank oxygen uptake rate measurements from empty respirometry chambers were taken at the start and end of each trial and subtracted from overall measurements by assuming an exponential increase from start to end. To minimize microbial respiration, water circulating in the respirometers was treated with ultraviolet light and the whole system was bleached daily with sodium hypochlorite. Following MMR measurements, fish remained in the same respirometry chambers overnight to allow SMR to be estimated. SMR was estimated as the lower 10th percentile of all 9-min oxygen uptake rate measurements for each fish excluding the initial slope used in MMR measurements. Fish were then removed from the respirometry chambers the following morning. Following removal, fish were measured for wet mass and standard length.

### Critical swimming speed (*U*_crit_)

Critical swimming speed tests were performed in the same swim tunnel as described for the trawl but without the trawl net in place. Fish were rested for a minimum of 5 days after respirometry assays and fasted for at least 24 h before trials. Fish were placed into the flume and acclimated in groups (*n* = 12) at a speed of 4 cm s^−1^ at 26°C. The acclimation protocol differed between the two conditions tested: under normoxia, fish were acclimated for 30 min prior to being tested for *U*_crit_, while under hypoxia fish were initially placed into the swim tunnel in normoxic water after which the oxygen content of the water was steadily decreased until 35% air saturation was achieved (mean ± SD time: 78 ± 30 min). Following acclimation, water velocity was increased in increments of 4 cm s^−1^ at 5-min intervals until the fish fatigued. Fatigue was determined as the point where a fish remained in contact with the rear screen of the working section of the flume for 3 s. The fish were then carefully removed from the swim tunnel through a small hatch in the lid above the back screen using a dip net, identified (via elastomer tag), and the corresponding time and water velocity were recorded. The critical swimming speed (*U*_crit_; cm s^−1^) was then estimated according to the equation

$$\begin{equation*}U_{\textrm{crit}} = [U + (T / Ti \times Ui)] / L\end{equation*}$$

where *U* is the highest velocity maintained for a complete interval (cm s^−1^), *T* is the time the fish swam at the last velocity before fatiguing (s), *T_i_* is the interval time between each speed increment (300 s), *U_i_* is the velocity increment (4 cm s^−1^) and *L* is the fish length (cm). The tests were repeated three times every other day for 36 fish (those from holding tank four) to estimate the repeatability of *U*_crit_ under hypoxia and normoxia. The fish in the remaining holding tanks were tested once under hypoxia and once under normoxia.

### Statistical analysis

Statistical analyses were performed in R 3.4.3 (R Core Team, 2017) and all visuals were completed using ‘ggplot2’ (Wickham, 2016). The effect of hypoxia on *U*_crit_ was estimated by using a linear mixed-effects model (LMM) fitted with the *nlme* package using a Gaussian error distribution (Pinheiro *et al.*, 2019). In this initial model, we were interested in the effect of hypoxia on the swimming performance of the fish, and therefore the model was constructed using *U*_crit_ as the response variable, oxygen availability (normoxia vs. hypoxia), SMR, MMR, mass and sex as fixed effects, and fish ID as a random effect. All continuous variables were scaled using the function *scale* in base R. We included all two-way interactions between oxygen availability and all other explanatory variables. The full model was fitted using restricted maximum likelihood (REML) estimation and compared via likelihood ratio testing to the same model without the random component (Zuur *et al*., 2009). Given the results of the likelihood ratio test (LRT) test we kept the random component and proceeded with model selection by using maximum likelihood (ML) estimation, dropping variables one by one, starting with that which had the highest *P*-value, to the point where removal of a term resulted in a poorer model (as indicated by LRTs).

To model capture vulnerability, a second model was constructed using a repeated measures generalized linear mixed model (GLMM) with a binomial error distribution in the the 'lme4' package (Bates *et al.*, 2014). Capture (1 = captured, 0 = not captured) was included as the response variable in the model; all explanatory variables were kept the same as the first model, though we added *U*_crit_ and the interaction *U*_crit_:oxygen availability. We used the package ‘lme4’ to apply ‘BOBYQA’ for bound constrained optimization (Powell, 2009) in order to reach appropriate model convergence, with the maximum number of iterations set to 100 000. REML estimation was not available for this function thus we fitted the model using ML. Again, we dropped variables with the highest *P*-values, one by one, until the minimum adequate model was established through LRT testing.

We constructed a third model using T*_net_* for fish that were caught to investigate the factors affecting the time each fish spent in the net. Here we used the ‘glmmTMB’ package (Brooks *et al.*, 2017) to fit a GLMM using a β error distribution and log-link function. A β distribution was used as the response variable was bounded between 0 and 1, and no zeros were present in the data. Model selection for the third model was carried out in a similar fashion to the first model. However, unlike the first two models, we found that fish ID did not provide sufficient information to justify its inclusion in the final model, it was thus removed (Zuur *et al*., 2009). Mass was kept in all models that contained metabolic variables regardless of significance to control for the effect of body mass on metabolism. Model marginal and conditional *r*^2^ values (*r^2^_m_* and *r*^2^_*c*_, respectively) were calculated using the package ‘MuMIn’ (Bartoń, 2013); however, owing to the β error distribution, no *r*^2^ value was calculated for the T*_net_* model.

The consistency of swimming under both oxygen availability treatments, as well as across context, was evaluated for tank four by calculating intraclass correlation coefficients as estimates of repeatability (*R*) using the ‘rptR’ package (Stoffel *et al.*, 2017). Whilst repeatability of capture was calculated for all fish from all tanks. The models controlled the variance components for factors caused by sex, length and oxygen availability. Results are based on 1000 bootstrapping and permutation runs; *P*-values are based on LRT testing. We transformed the T*_net_* data to proportion given that the variable was bounded (i.e. T*_net_* could have not taken longer than the duration of the test). Owing to the continuous and bounded nature of the T*_net_* data, repeatability could not be calculated using the same approach as for swimming and capture, and instead Pearson correlation coefficients (*r_p_*) were calculated using the measures for the two trials within each oxygen availability treatment, as well as across context (normoxia vs*.* hypoxia). Finally, a series of Levene’s tests using the package ‘lawstat’ (Gastwirth *et al.*, 2017) were used to assess whether the variance of *U*_crit_ and T*_net_* differed across hypoxia and normoxia; the results presented are based on 1000 bootstrapping runs.

## Results

**Figure 2:**
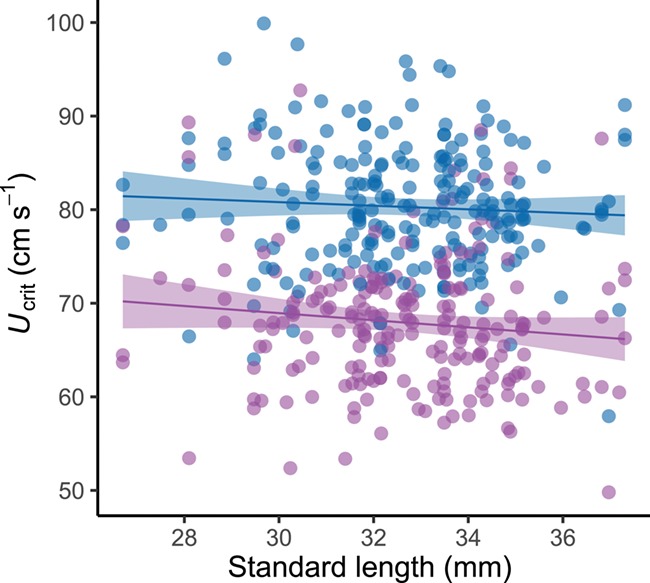
The effect of environmental oxygen availability on the swimming capacity of zebrafish. Blue points represent critical swimming speed (*U*_crit_) under normoxia (n = 217), whilst purple points represent Ucrit for the same fish under hypoxia (35% air saturation) (n = 210). For reference, lines represent the ordinary least squares regressions for each group. Regression equations are: normoxia = 86.579 − (0.192 ^*^ X ); hypoxia = 80.345 − (0.379 ^*^ X). Shaded areas correspond to 95% confidence intervals.

**Figure 3:**
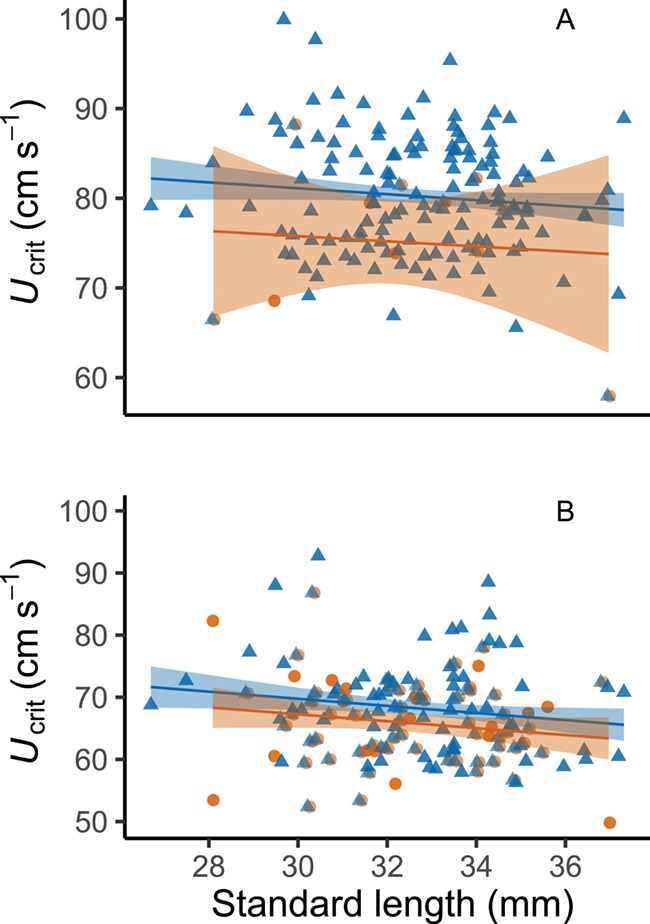
Relationships among swimming performance (*U*_crit_), standard length, and capture for the same fish in normoxia(A) and hypoxia (B). Orange circles represent fish that were caught during trials, whilst blue triangles represent fish that were not caught. For reference, lines represent the ordinary least squares regressions for both caught and uncaught fish in each oxygen availability treatment. Regression equations in A are: not caught = 91.004 -- (0.329 ^*^ X); caught = (84.352) -- (0.286 ^*^ X). Corresponding equations in B are: not caught  = 86.920 -- (0.572 ^*^ X); caught = (83.958) -- (0.556 ^*^ X). Shaded areas correspond to 95% confidence intervals.

**Figure 4:**
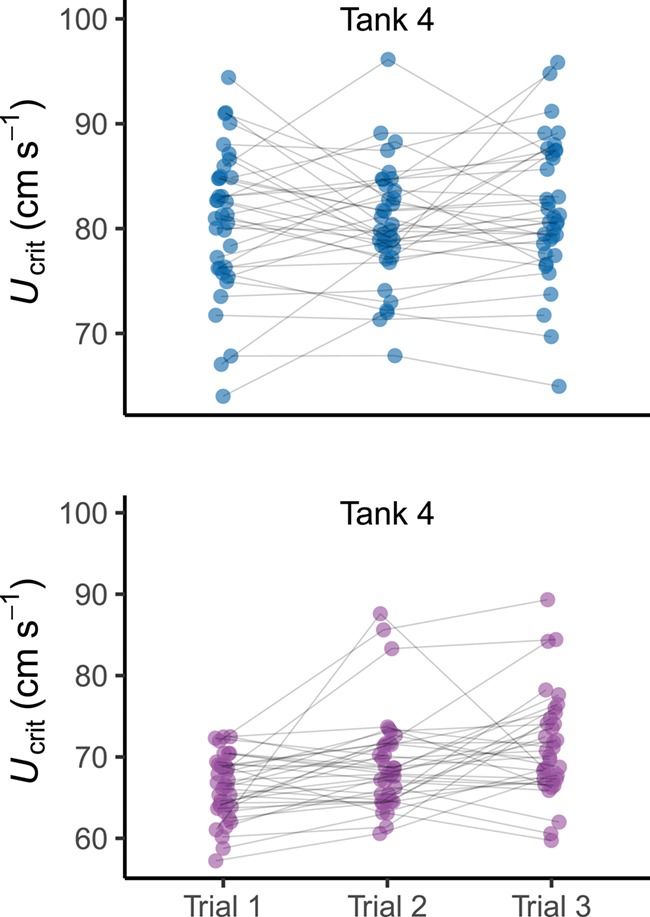
Swimming performance (*U*_crit_) within both normoxia and hypoxia over three trials Blue points represent swimming in normoxia and purple points swimming in hypoxia. Lines connecting points are representative of individual performance with each oxygen availability treatment (*n* = 106).

Swimming performance was strongly affected by hypoxia (Table 1; GLMM, *P* < 0.001; *r*^2^*_m_* = 0.51; *r*^2^*_c_* = 0.72) with mean ± SE *U*_crit_ being 67.9 ± 0.49 cm s^−1^ in hypoxia and 80.3 ± 0.45 cm s^−1^ in normoxia (Fig. 2). Capture increased significantly under hypoxic conditions (Table 2; GLMM, *P* < 0.001; *r*^2^*_m_* = 0.30; *r*^2^*_c_* = 0.46). There was an ~7-fold difference in the number of fish that were captured between normoxia and hypoxia (hypoxia, *n* = 95; normoxia, *n* = 14). Oxygen availability, however, did not affect T*_net_* of captured fish. Swimming performance was found to have a strong effect on capture across the population (Table 2), with fish displaying a lower *U*_crit_ being captured more readily (Fig. 3), but did not have an effect on T*_net_*. Only mass was found to have a significant impact on T*_net_* (Table 3; GLMM, *P* = 0.039). Metabolic rates did not affect whether a fish was caught nor how long it spent in the net, though there was a strong link between *U*_crit_ and MMR (Table 1; GLMM, *P* < 0.001; *r*^2^_*m*_ = 0.51; *r*^2^_*c*_ = 0.72). *U*_crit_ was strongly repeatable under normoxic conditions (*R* = 0.641, 95% CI = 0.464–0.779, *P* < 0.001; *n* = 108) but dropped considerably in hypoxia (*R* = 0.346, 95% CI = 0.118–0.574, *P* < 0.001; *n* = 106; Figs 4 and 6). Across contexts (normoxia and hypoxia), the repeatability of *U*_crit_ was reduced but still significant (*R* = 0.406, 95% CI = 0.247–0.558, *P* < 0.001; *n* = 214). Similarly, repeatability of capture (whether a fish was caught or not caught) was higher in normoxia than in hypoxia (normoxia *R* = 0.831, 95% CI = 0.966–0.994, *P* < 0.001; *n* = 290 vs. hypoxia *R* = 0.045, 95% CI = 0–0.163, *P* < 0.001; *n* = 291). Again, similarly to *U*_crit_, repeatability of capture across contexts (i.e. when the same fish were tested at hypoxia and normoxia) was reduced but still significant when compared to normoxic conditions (*R* = 0.195, 95% CI = 0.039–0.303, *P* < 0.001; *n* = 581).

**Table 1:** LMM (Gaussian error distribution) results for *U*_crit_. (*n* = 335)

**Term**	**Estimate**	***SE***	***t***	***P***
Intercept	−0.828	0.080	−11.179	**<0.001**
Oxygen availability—normoxia	1.293	0.058	22.014	**<0.001**
MMR	0.202	0.055	3.607	**<0.001**
Mass	−0.197	0.062	−3.122	**<0.001**
Sex—male	0.347	0.118	2.906	**<0.001**

Significant effects are indicated in bold. See supplementary materials for details on model selection.

**Figure 5:**
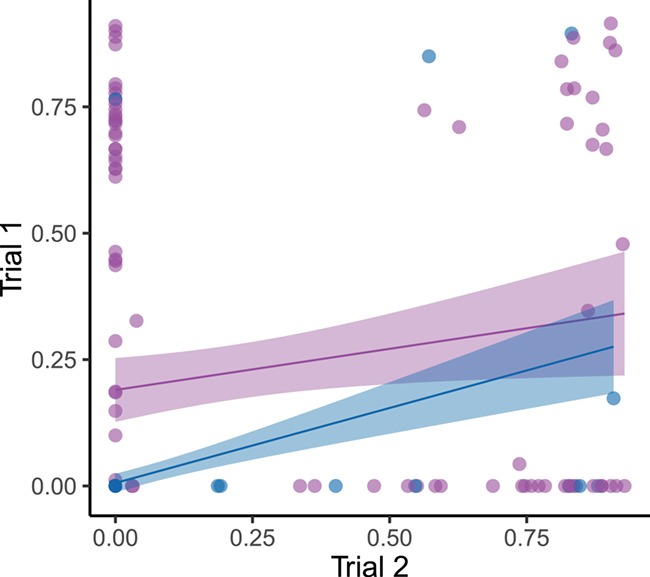
The relationship between trial 1 and trial 2 expressed as a proportion of time spent in the net (T*_net_*) for the same fish under both normoxia (blue) and hypoxia (purple). The maximum time a fish could spend in the net was 10 minutes. Pearson product-moment correlations (*r_p_*) were 0.425 in normoxia (*t*_288_ = 5.620, *P* < 0.001) and 0.172 in hypoxia (*t*_288_ = 2.095, *P* <0.05). For reference, lines represent the ordinary least squares regressions for each group. Regression equations are: normoxia = 0.189 + (0.162 ^ ^ X.

**Figure 6:**
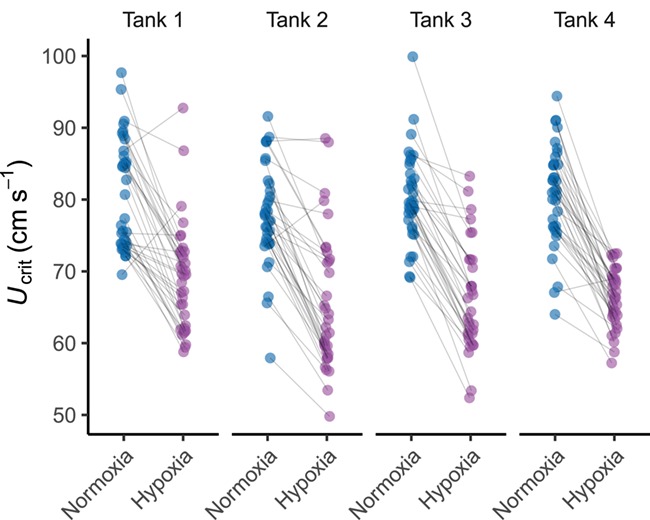
Swimming performance (*U*_crit_) in normoxic (blue) and hypoxic (purple) conditions across test tanks (n = 285). For Tank 4, only the first trial in each condition is shown (see Fig. 4 for all trials for fish in this holding tank). Lines connecting points represent the same individual within each oxygen availability treatment.

Across the two normoxic trials, measures of T*_net_* were positively correlated (*r_p_* = 0.425, *t*_288_ = 5.620, *P* < 0.001), but the strength of this correlation diminished under hypoxia (*r_p_* = 0.172, *t*_288_ = 2.095, *P* = 0.037; Fig. 5). Across context (normoxia and hypoxia) correlation of T_*net*_ was also found to be significant (*r_p_* = 0.251, *t*_578_ = 2.095, *P* =  < 0.001). Though the variance of swimming performance was reduced by hypoxia among the fish that were tested for *U*_crit_ three times under each condition (Fig. 4), we did not find a significant difference in variance between oxygen availability treatments across all fish (Levene’s test, *F*_1,283_ = 0.145, *P* = 0.708; Fig. 6). In contrast, across all fish, we saw a significant difference in the variance of T*_net_* (Levene’s test, *F*_1,579_ = 72.758, *P* < 0.001).

## Discussion

The results here indicate that exposure to hypoxia decreases swimming performance and increases capture vulnerability of individual fish, suggesting that the level of dissolved oxygen in the environment is likely an important parameter determining exploitation from fisheries. Furthermore, harvest-associated selection is likely to be attenuated by hypoxic conditions, as repeatability of both capture and key traits related to gear avoidance, such as swimming performance, were reduced under hypoxia. SMR and MMR played no clear role in driving vulnerability to capture in either normoxia or hypoxia, though they did affect swimming performance.

**Table 2:** Binomial GLMM results for capture (*n* = 496)

**Term**	**Estimate**	***SE***	***Z***	***P***
Intercept	−0.944	0.192	−4.911	**<0.001**
Oxygen availability—normoxia	−2.613	0.381	−6.851	**<0.001**
*U*_crit_	−0.422	0.171	−2.465	**0.0137**

Significant effects are indicated in bold.

### Hypoxia, swimming and implications for capture

Fish that had a higher swimming performance (higher *U*_crit_) were less vulnerable to capture in both normoxia and hypoxia (Fig. 3). Capture rate was much higher during hypoxic trials, likely owing to reduced *U*_crit_. The increase in susceptibility to capture observed here in response to hypoxia is analogous to that reported in wild fisheries where surges in capture can occur prior to the onset of extreme hypoxic or anoxic conditions (Rossignol-strick, 1985). Whereas in a natural predator–prey interaction both predator and prey would be limited by low dissolved oxygen (Domenici *et al.*, 2007), no such mechanism would be at play in a commercial fishing scenario, thus the potential for increased mortality is likely greater.

**Table 3:** Beta GLMM for vulnerability (T*_net_*; *n* = 90)

**Term**	**Estimate**	***SE***	***Z***	***P***
Intercept	0.538	0.105	5.094	**<0.001**
Mass	−0.213	0.103	−2.055	**0.039**

Significant effects are indicated in bold. Not all captured fish were included in this model owing to missing data.

*U*_crit_ displayed high within-context repeatability in normoxic conditions and a lower repeatability under hypoxic conditions. This indicates that the swimming consistency is disrupted by hypoxia and suggests that swimming capacity is more likely a target for selection under normoxia. It is notable, however, that across-context repeatability of *U*_crit_ between the hypoxic and normoxic treatments was still relatively high, meaning that some level of consistency across conditions continues to be displayed. Together, these results suggest that, although hypoxia decreases overall swimming performance within a population, fish which perform relatively well under normoxia are still likely to perform better than their counterparts in hypoxia. Other work suggests that repeatability of swimming performance in hypoxia may improve with acclimation to chronic hypoxia (Kraskura and Nelson, 2018). This, however, may not provide an advantage if a fish encountered hypoxic areas sporadically. It should be noted that, because we assayed for *U*_crit_ in groups, it is possible that some fish may have been able to gain an energetic advantage during the assay by exploiting vortices of groupmates (Hemelrijk *et al*., 2015). While this may have increased variance in the normoxic trials, the overwhelming effect of hypoxia on swimming ability is likely to have superseded any effects of hydrodynamics on swimming ability in the hypoxic trials. It is also noteworthy that, despite any increased variance during normoxia due to group swimming, individual *U*_crit_ still displayed high within-context repeatability at normoxia. Overall, our results show that capture vulnerability across a population may shift according to prevailing environmental conditions, which could serve to weaken potential evolutionary selection. Capture vulnerability was also shown to be highly repeatable in normoxia, consistent with previous findings (Killen *et al.*, 2015), but decreased in hypoxia. Hypoxia had a stronger effect on the repeatability of capture than on the repeatability of *U*_crit_. Results suggest that anthropogenic harvest may be reduced under hypoxic conditions, as the phenotype under selection may vary according to the prevailing conditions.

### Hypoxia and metabolism

Neither SMR nor MMR, as measured in normoxia, influenced susceptibility of individual fish to capture. This was surprising, especially given that MMR is related to swimming performance in some fish species (Claireaux, 2005) and has previously been found to affect capture in a simulated trawl scenario (Killen *et al.*, 2015). In the current study, we did find a relationship between MMR and swimming (Table 1), though this effect was not sufficiently strong to significantly influence capture. Additional work could examine how individual sensitivity to hypoxia, measured in terms of *P_crit_* or the magnitude of the change in MMR with hypoxia, may be related to changes in capture vulnerability. Another factor contributing to the lack of a relationship between capture vulnerability and metabolic traits could be variation in the anaerobic contribution to swimming during escape from the trawl. Aerobic metabolism may drive constant swimming in front of a net, while anaerobic metabolism may be utilized in the high-energy bursts (similar to fast-start responses; Domenici *et al.*, 2007) needed to escape a net when close to it or within its mouth (He, 2010). Evidence suggests that the effects of hypoxia are not only limited to aerobic capacity but can also extend to anaerobic swimming, primarily by impairing brain and sensory function (Domenici *et al.*, 2007). It is likely that both aerobic and anaerobic swimming are important in determining capture in trawl fisheries and associated selection (Killen *et al.*, 2015).

### Impacts of hypoxia on other traits relevant to capture

In addition to limiting swimming performance, hypoxia may modulate many other physiological and behavioural traits related to capture vulnerability. Harvest selection has been shown to operate across many fronts including behaviour (Diaz Pauli and Sih, 2017), body shape (Alós *et al.*, 2014), habitat preference (Arnason *et al.*, 2009), size (Swain *et al.*, 2007) and maturation (Olsen *et al.*, 2004). Hypoxia has the potential to interact with all of these processes upstream of capture to affect selection. For instance, hypoxia is known to impair overall cognitive capacity (Lefrançois and Domenici, 2006; Domenici *et al.*, 2007), which could have significant implications for escape success.

Effects of hypoxia can also be tissue specific or impact particular behaviours. Visual acuity, a key trait in avoiding predation encounters, is heavily reliant on oxygen availability and may be limited under low oxygen conditions. Importantly, this is likely to occur prior to any downregulation of locomotor capacity (Robinson *et al.*, 2013; McCormick and Levin, 2017). Behaviours associated with shoaling can also be impacted by hypoxia. Previous work on *Clupea harengus* has found that school length, width and area all increase under low oxygen (Domenici *et al.*, 2002). It is unclear as to how this would impact trawl capture specifically, though previous work on trapping has shown that larger shoals are more susceptible to harvest in trap gears (Thambithurai *et al.*, 2018). An avenue for future research will be to understand which traits are more likely to be the target of selection under prevailing environmental conditions.

Although sex did not dictate whether or not a fish was ultimately captured, females that were captured spent more time in the net (higher T*_net_*), indicating they were captured sooner during a given trial. This difference could be especially important during discard scenarios. Previous work has shown a positive correlation between the time for which a net is towed along the sea floor and mortality in bycatch (Heard *et al.*, 2014), suggesting that fish that spend more time in a net are more likely to sustain injuries and incur mortalities. This has the potential to translate into sexual selection, as females could potentially incur greater mortality than males. Importantly, fisheries-induced evolution originating from sexual selection has already been shown to have significant consequences for population dynamics in non-finfish fisheries (Sørdalen *et al.*, 2018). Nevertheless, this mechanism is likely to be species-specific, as differences in physiology and swimming modes may modulate capture across sexes.

For fishery selection to occur, fish must occupy the active space in which a gear is deployed (Hollins *et al.*, 2018). In the present study, we concentrated our efforts in understanding how hypoxia impacts capture once a fish is in front of a trawl. However, the nature of hypoxia means that it can be spatially restricted or extend for thousands of kilometres (Tyler *et al.*, 2009). This can have significant impacts on the movement of fish, ultimately modifying habitat use and potentially changing selection potential. Among the range of responses employed by fish in dealing with low oxygen, behavioural avoidance remains of critical importance (Breitburg, 2002). Individual physiological tolerances and behaviours of fish to hypoxia will largely dictate which species or individuals will vacate a hypoxic zone. It remains unclear whether the depression of physiological processes that affect fish–gear interactions occurs prior to the onset of behavioural avoidance. If changes to an individual’s performance occur prior to the onset of behavioural avoidance, then the potential for disruption to selection on physiological performance is greater. In addition, short exposures to low oxygen conditions may be sufficient to affect escape for some time, meaning that fish may leave an area of hypoxia but their escape performance may still be affected.

## Conclusions

Within-species selection stemming from fishing occurs within dynamically changing environments. Targeted individuals will therefore be constantly displaying behavioural and physiological plasticity as they encounter varying environmental conditions over various timescales. This plasticity will potentially alter which fish are most vulnerable to capture and therefore which traits are under selection. Individuals that are most likely to be captured under one set of conditions may not be most vulnerable under another set of conditions. Here, we show that a single environmental parameter, hypoxia, may disrupt harvest-associated selection by altering physiological performance and variation in vulnerability to capture. Swimming performance was heavily impacted by hypoxia and had a direct knock-on effect on capture increasing the number of fish caught by ~7-fold. Clearly, our results indicate that more fish are captured in hypoxic conditions, but more importantly, the key finding here is that at the level of hypoxia tested the selective mechanisms that underpin fishing are altered. It is also worth noting that this modulatory effect might be less severe or different under milder hypoxia. Our findings highlight a mechanistic synergy between hypoxic episodes and fisheries-induced evolution, two significant stressors affecting fish populations. A range of other environmental factors, including temperature and food availability, may also influence trait plasticity and therefore which phenotypes may be favoured in response to fishing pressure or other anthropogenically-induced stressors. We encourage further studies addressing harvest-associated selection under changing environments, particularly in response to anthropogenic climate change.

## Supplementary Material

Supplementary_materials_1of3_coz082

Supplementary_materials_2of3_coz082

Supplementary_materials_3of3_coz082
