# Supplementary figures and images for: Hypoxia alters vulnerability to capture and the potential for trait-based selection in a scaled-down trawl fishery
Source: Conserv Physiol. 2019 Nov 27;7(1):coz082. doi: 10.1093/conphys/coz082 (PMC6880855; doi:10.1093/conphys/coz082)

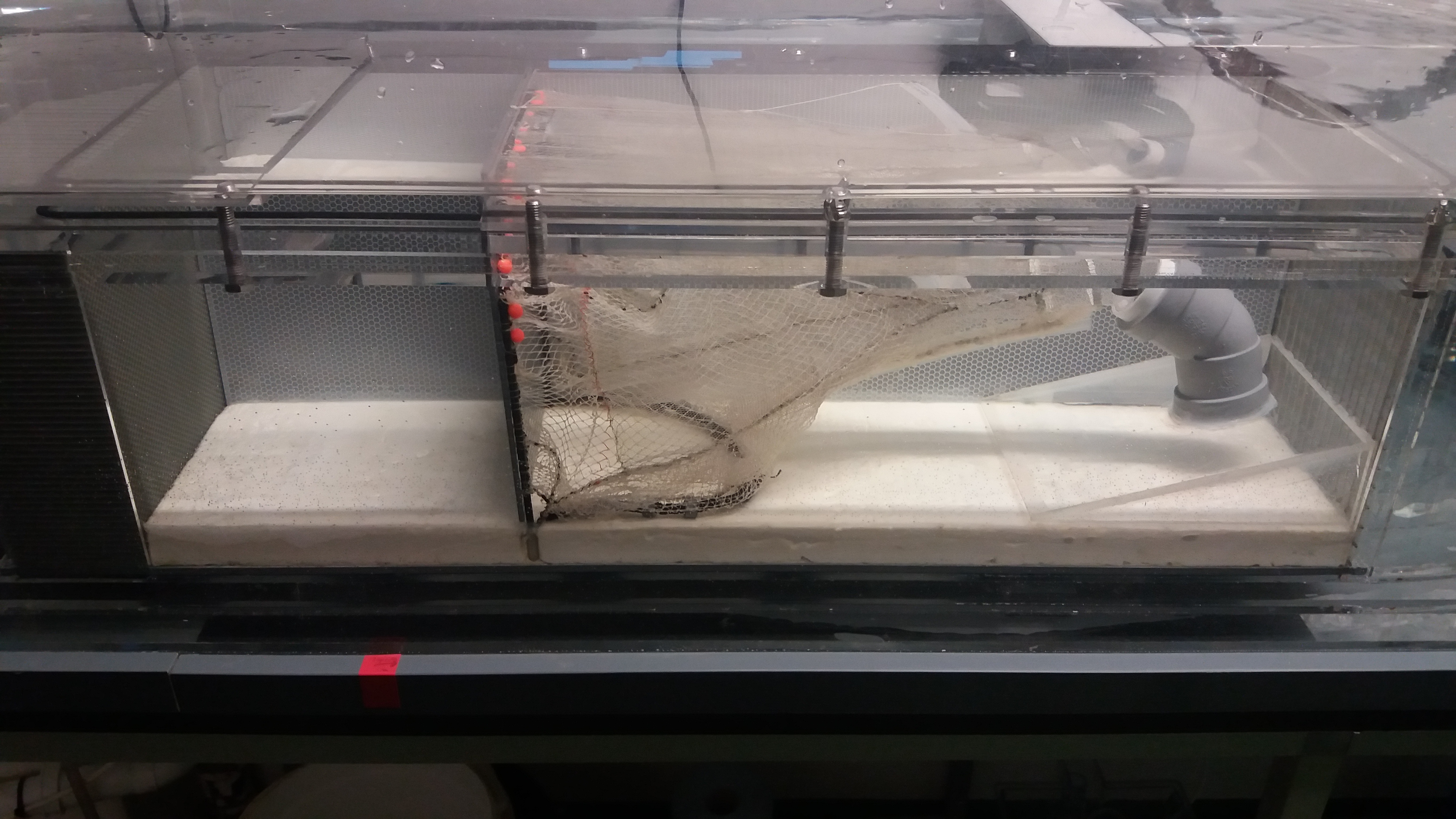

Supplement: Supplementary_materials_1of3_coz082 [file supplementary_materials_1of3_coz082.jpeg]
