# Supplementary material for: Hypoxia alters vulnerability to capture and the potential for trait-based selection in a scaled-down trawl fishery
Source: Conserv Physiol. 2019 Nov 27;7(1):coz082. doi: 10.1093/conphys/coz082 (PMC6880855; doi:10.1093/conphys/coz082)
Supplement: Supplementary_materials_2of3_coz082 [file supplementary_materials_2of3_coz082.pdf]

fish.ID:	fish ID
h.tank:	holding tank
sex:	sex
w.mass:	wet mass
s.length:	standard length
mmr:	maximum metabolic rate (mg/h)
smr:	stadard metabolic rate (mg/h)
fishing.treat:	treatment (hypoxia or normoxia) for the trawl assays
fishing.rep.no:	replicate number for trawl assay
ft.prop:	proportion of 600 s trial spent in net
cap.status:	binary capture (1 = captured, 0= not captured)
swim.treat.rep:	replicate number for normoxia (norm) or hypoxia (hyp)
Ucrit:	critical swimming speed (cm/s)

fish.ID	h.tank	sex	w.mass	s.length	mmr	smr	fishing.treat	fishing.rep.no	ft.prop	cap.status	swim.treat.rep	Ucrit
_rgr	1				1.165	0.215	normoxia	1	0	0		
_rgr	1				1.165	0.215	hypoxia	1	0	0		
_rgr	1				1.165	0.215	normoxia	2	0	0		
_rgr	1				1.165	0.215	hypoxia	2	0	0		
ggpg	4	f	0.542	31.7	1.216	0.193	normoxia	1	0	0	Ucrit1norm	80.9618
ggpg	4	f	0.542	31.7	1.216	0.193	normoxia	1	0	0	Ucrit2norm	78.9013
ggpg	4	f	0.542	31.7	1.216	0.193	normoxia	1	0	0	Ucrit3norm	78.5526
ggpg	4	f	0.542	31.7	1.216	0.193	normoxia	1	0	0	Ucrit1hyp	72.3077
ggpg	4	f	0.542	31.7	1.216	0.193	normoxia	1	0	0	Ucrit2hyp	70.2155
ggpg	4	f	0.542	31.7	1.216	0.193	normoxia	1	0	0	Ucrit3hyp	68.2818
ggpg	4	f	0.542	31.7	1.216	0.193	hypoxia	1	0	0	Ucrit1norm	80.9618
ggpg	4	f	0.542	31.7	1.216	0.193	hypoxia	1	0	0	Ucrit2norm	78.9013
ggpg	4	f	0.542	31.7	1.216	0.193	hypoxia	1	0	0	Ucrit3norm	78.5526
ggpg	4	f	0.542	31.7	1.216	0.193	hypoxia	1	0	0	Ucrit1hyp	72.3077
ggpg	4	f	0.542	31.7	1.216	0.193	hypoxia	1	0	0	Ucrit2hyp	70.2155
ggpg	4	f	0.542	31.7	1.216	0.193	hypoxia	1	0	0	Ucrit3hyp	68.2818
ggpg	4	f	0.542	31.7	1.216	0.193	normoxia	2	0	0	Ucrit1norm	80.9618
ggpg	4	f	0.542	31.7	1.216	0.193	normoxia	2	0	0	Ucrit2norm	78.9013
ggpg	4	f	0.542	31.7	1.216	0.193	normoxia	2	0	0	Ucrit3norm	78.5526
ggpg	4	f	0.542	31.7	1.216	0.193	normoxia	2	0	0	Ucrit1hyp	72.3077
ggpg	4	f	0.542	31.7	1.216	0.193	normoxia	2	0	0	Ucrit2hyp	70.2155
ggpg	4	f	0.542	31.7	1.216	0.193	normoxia	2	0	0	Ucrit3hyp	68.2818
ggpg	4	f	0.542	31.7	1.216	0.193	hypoxia	2	0	0	Ucrit1norm	80.9618
ggpg	4	f	0.542	31.7	1.216	0.193	hypoxia	2	0	0	Ucrit2norm	78.9013
ggpg	4	f	0.542	31.7	1.216	0.193	hypoxia	2	0	0	Ucrit3norm	78.5526
ggpg	4	f	0.542	31.7	1.216	0.193	hypoxia	2	0	0	Ucrit1hyp	72.3077
ggpg	4	f	0.542	31.7	1.216	0.193	hypoxia	2	0	0	Ucrit2hyp	70.2155
ggpg	4	f	0.542	31.7	1.216	0.193	hypoxia	2	0	0	Ucrit3hyp	68.2818
ggpp	3	f	0.614	32.3	1.154	0.205	hypoxia	1	0	0	Ucrit1norm	81.4606
ggpp	3	f	0.614	32.3	1.154	0.205	hypoxia	1	0	0	Ucrit2norm	
ggpp	3	f	0.614	32.3	1.154	0.205	hypoxia	1	0	0	Ucrit3norm	
ggpp	3	f	0.614	32.3	1.154	0.205	hypoxia	1	0	0	Ucrit1hyp	66.2796

ggpp	3	f	0.614	32.3	1.154	0.205	hypoxia	1	0	0	Ucrit2hyp	
ggpp	3	f	0.614	32.3	1.154	0.205	hypoxia	1	0	0	Ucrit3hyp	
ggpp	3	f	0.614	32.3	1.154	0.205	normoxia	1	0	0	Ucrit1norm	81.4606
ggpp	3	f	0.614	32.3	1.154	0.205	normoxia	1	0	0	Ucrit2norm	
ggpp	3	f	0.614	32.3	1.154	0.205	normoxia	1	0	0	Ucrit3norm	
ggpp	3	f	0.614	32.3	1.154	0.205	normoxia	1	0	0	Ucrit1hyp	66.2796
ggpp	3	f	0.614	32.3	1.154	0.205	normoxia	1	0	0	Ucrit2hyp	
ggpp	3	f	0.614	32.3	1.154	0.205	normoxia	1	0	0	Ucrit3hyp	
ggpp	3	f	0.614	32.3	1.154	0.205	hypoxia	2	0.55	1	Ucrit1norm	81.4606
ggpp	3	f	0.614	32.3	1.154	0.205	hypoxia	2	0.55	1	Ucrit2norm	
ggpp	3	f	0.614	32.3	1.154	0.205	hypoxia	2	0.55	1	Ucrit3norm	
ggpp	3	f	0.614	32.3	1.154	0.205	hypoxia	2	0.55	1	Ucrit1hyp	66.2796
ggpp	3	f	0.614	32.3	1.154	0.205	hypoxia	2	0.55	1	Ucrit2hyp	
ggpp	3	f	0.614	32.3	1.154	0.205	hypoxia	2	0.55	1	Ucrit3hyp	
ggpp	3	f	0.614	32.3	1.154	0.205	normoxia	2	0.828333333	1	Ucrit1norm	81.4606
ggpp	3	f	0.614	32.3	1.154	0.205	normoxia	2	0.828333333	1	Ucrit2norm	
ggpp	3	f	0.614	32.3	1.154	0.205	normoxia	2	0.828333333	1	Ucrit3norm	
ggpp	3	f	0.614	32.3	1.154	0.205	normoxia	2	0.828333333	1	Ucrit1hyp	66.2796
ggpp	3	f	0.614	32.3	1.154	0.205	normoxia	2	0.828333333	1	Ucrit2hyp	
ggpp	3	f	0.614	32.3	1.154	0.205	normoxia	2	0.828333333	1	Ucrit3hyp	
ggpr	1	f	0.499	32	1.148	0.184	normoxia	1	0	0	Ucrit1norm	73.888
ggpr	1	f	0.499	32	1.148	0.184	normoxia	1	0	0	Ucrit2norm	
ggpr	1	f	0.499	32	1.148	0.184	normoxia	1	0	0	Ucrit3norm	
ggpr	1	f	0.499	32	1.148	0.184	normoxia	1	0	0	Ucrit1hyp	72.704
ggpr	1	f	0.499	32	1.148	0.184	normoxia	1	0	0	Ucrit2hyp	
ggpr	1	f	0.499	32	1.148	0.184	normoxia	1	0	0	Ucrit3hyp	
ggpr	1	f	0.499	32	1.148	0.184	hypoxia	1	0.776666667	1	Ucrit1norm	73.888
ggpr	1	f	0.499	32	1.148	0.184	hypoxia	1	0.776666667	1	Ucrit2norm	
ggpr	1	f	0.499	32	1.148	0.184	hypoxia	1	0.776666667	1	Ucrit3norm	
ggpr	1	f	0.499	32	1.148	0.184	hypoxia	1	0.776666667	1	Ucrit1hyp	72.704
ggpr	1	f	0.499	32	1.148	0.184	hypoxia	1	0.776666667	1	Ucrit2hyp	
ggpr	1	f	0.499	32	1.148	0.184	hypoxia	1	0.776666667	1	Ucrit3hyp	
ggpr	1	f	0.499	32	1.148	0.184	normoxia	2	0	0	Ucrit1norm	73.888

ggpr	1	f	0.499	32	1.148	0.184	normoxia	2	0	0	Ucrit2norm	
ggpr	1	f	0.499	32	1.148	0.184	normoxia	2	0	0	Ucrit3norm	
ggpr	1	f	0.499	32	1.148	0.184	normoxia	2	0	0	Ucrit1hyp	72.704
ggpr	1	f	0.499	32	1.148	0.184	normoxia	2	0	0	Ucrit2hyp	
ggpr	1	f	0.499	32	1.148	0.184	normoxia	2	0	0	Ucrit3hyp	
ggpr	1	f	0.499	32	1.148	0.184	hypoxia	2	0	0	Ucrit1norm	73.888
ggpr	1	f	0.499	32	1.148	0.184	hypoxia	2	0	0	Ucrit2norm	
ggpr	1	f	0.499	32	1.148	0.184	hypoxia	2	0	0	Ucrit3norm	
ggpr	1	f	0.499	32	1.148	0.184	hypoxia	2	0	0	Ucrit1hyp	72.704
ggpr	1	f	0.499	32	1.148	0.184	hypoxia	2	0	0	Ucrit2hyp	
ggpr	1	f	0.499	32	1.148	0.184	hypoxia	2	0	0	Ucrit3hyp	
ggrr	2	f	0.559	32.84	0.906	0.17	hypoxia	1	0	0	Ucrit1norm	73.52876
ggrr	2	f	0.559	32.84	0.906	0.17	hypoxia	1	0	0	Ucrit2norm	
ggrr	2	f	0.559	32.84	0.906	0.17	hypoxia	1	0	0	Ucrit3norm	
ggrr	2	f	0.559	32.84	0.906	0.17	hypoxia	1	0	0	Ucrit1hyp	79.83404
ggrr	2	f	0.559	32.84	0.906	0.17	hypoxia	1	0	0	Ucrit2hyp	
ggrr	2	f	0.559	32.84	0.906	0.17	hypoxia	1	0	0	Ucrit3hyp	
ggrr	2	f	0.559	32.84	0.906	0.17	normoxia	1	0	0	Ucrit1norm	73.52876
ggrr	2	f	0.559	32.84	0.906	0.17	normoxia	1	0	0	Ucrit2norm	
ggrr	2	f	0.559	32.84	0.906	0.17	normoxia	1	0	0	Ucrit3norm	
ggrr	2	f	0.559	32.84	0.906	0.17	normoxia	1	0	0	Ucrit1hyp	79.83404
ggrr	2	f	0.559	32.84	0.906	0.17	normoxia	1	0	0	Ucrit2hyp	
ggrr	2	f	0.559	32.84	0.906	0.17	normoxia	1	0	0	Ucrit3hyp	
ggrr	2	f	0.559	32.84	0.906	0.17	hypoxia	2	0	0	Ucrit1norm	73.52876
ggrr	2	f	0.559	32.84	0.906	0.17	hypoxia	2	0	0	Ucrit2norm	
ggrr	2	f	0.559	32.84	0.906	0.17	hypoxia	2	0	0	Ucrit3norm	
ggrr	2	f	0.559	32.84	0.906	0.17	hypoxia	2	0	0	Ucrit1hyp	79.83404
ggrr	2	f	0.559	32.84	0.906	0.17	hypoxia	2	0	0	Ucrit2hyp	
ggrr	2	f	0.559	32.84	0.906	0.17	hypoxia	2	0	0	Ucrit3hyp	
ggrr	2	f	0.559	32.84	0.906	0.17	normoxia	2	0	0	Ucrit1norm	73.52876
ggrr	2	f	0.559	32.84	0.906	0.17	normoxia	2	0	0	Ucrit2norm	
ggrr	2	f	0.559	32.84	0.906	0.17	normoxia	2	0	0	Ucrit3norm	
ggrr	2	f	0.559	32.84	0.906	0.17	normoxia	2	0	0	Ucrit1hyp	79.83404

ggrr	2	f	0.559	32.84	0.906	0.17	normoxia	2	0	0	Ucrit2hyp	
ggrr	2	f	0.559	32.84	0.906	0.17	normoxia	2	0	0	Ucrit3hyp	
ggry	3	f	0.733	34.51	1.067	0.176	hypoxia	1	0.186666667	1	Ucrit1norm	77.16436
ggry	3	f	0.733	34.51	1.067	0.176	hypoxia	1	0.186666667	1	Ucrit2norm	
ggry	3	f	0.733	34.51	1.067	0.176	hypoxia	1	0.186666667	1	Ucrit3norm	
ggry	3	f	0.733	34.51	1.067	0.176	hypoxia	1	0.186666667	1	Ucrit1hyp	63.4984
ggry	3	f	0.733	34.51	1.067	0.176	hypoxia	1	0.186666667	1	Ucrit2hyp	
ggry	3	f	0.733	34.51	1.067	0.176	hypoxia	1	0.186666667	1	Ucrit3hyp	
ggry	3	f	0.733	34.51	1.067	0.176	normoxia	1	0	0	Ucrit1norm	77.16436
ggry	3	f	0.733	34.51	1.067	0.176	normoxia	1	0	0	Ucrit2norm	
ggry	3	f	0.733	34.51	1.067	0.176	normoxia	1	0	0	Ucrit3norm	
ggry	3	f	0.733	34.51	1.067	0.176	normoxia	1	0	0	Ucrit1hyp	63.4984
ggry	3	f	0.733	34.51	1.067	0.176	normoxia	1	0	0	Ucrit2hyp	
ggry	3	f	0.733	34.51	1.067	0.176	normoxia	1	0	0	Ucrit3hyp	
ggry	3	f	0.733	34.51	1.067	0.176	hypoxia	2	0	0	Ucrit1norm	77.16436
ggry	3	f	0.733	34.51	1.067	0.176	hypoxia	2	0	0	Ucrit2norm	
ggry	3	f	0.733	34.51	1.067	0.176	hypoxia	2	0	0	Ucrit3norm	
ggry	3	f	0.733	34.51	1.067	0.176	hypoxia	2	0	0	Ucrit1hyp	63.4984
ggry	3	f	0.733	34.51	1.067	0.176	hypoxia	2	0	0	Ucrit2hyp	
ggry	3	f	0.733	34.51	1.067	0.176	hypoxia	2	0	0	Ucrit3hyp	
ggry	3	f	0.733	34.51	1.067	0.176	normoxia	2	0	0	Ucrit1norm	77.16436
ggry	3	f	0.733	34.51	1.067	0.176	normoxia	2	0	0	Ucrit2norm	
ggry	3	f	0.733	34.51	1.067	0.176	normoxia	2	0	0	Ucrit3norm	
ggry	3	f	0.733	34.51	1.067	0.176	normoxia	2	0	0	Ucrit1hyp	63.4984
ggry	3	f	0.733	34.51	1.067	0.176	normoxia	2	0	0	Ucrit2hyp	
ggry	3	f	0.733	34.51	1.067	0.176	normoxia	2	0	0	Ucrit3hyp	
ggyg	3	m	0.727	36.42	1.183	0.232	hypoxia	1	0	0	Ucrit1norm	78.08448
ggyg	3	m	0.727	36.42	1.183	0.232	hypoxia	1	0	0	Ucrit2norm	
ggyg	3	m	0.727	36.42	1.183	0.232	hypoxia	1	0	0	Ucrit3norm	
ggyg	3	m	0.727	36.42	1.183	0.232	hypoxia	1	0	0	Ucrit1hyp	61.40412
ggyg	3	m	0.727	36.42	1.183	0.232	hypoxia	1	0	0	Ucrit2hyp	
ggyg	3	m	0.727	36.42	1.183	0.232	hypoxia	1	0	0	Ucrit3hyp	
ggyg	3	m	0.727	36.42	1.183	0.232	normoxia	1	0	0	Ucrit1norm	78.08448

ggyg	3	m	0.727	36.42	1.183	0.232	normoxia	1	0	0	Ucrit2norm	
ggyg	3	m	0.727	36.42	1.183	0.232	normoxia	1	0	0	Ucrit3norm	
ggyg	3	m	0.727	36.42	1.183	0.232	normoxia	1	0	0	Ucrit1hyp	61.40412
ggyg	3	m	0.727	36.42	1.183	0.232	normoxia	1	0	0	Ucrit2hyp	
ggyg	3	m	0.727	36.42	1.183	0.232	normoxia	1	0	0	Ucrit3hyp	
ggyg	3	m	0.727	36.42	1.183	0.232	hypoxia	2	0	0	Ucrit1norm	78.08448
ggyg	3	m	0.727	36.42	1.183	0.232	hypoxia	2	0	0	Ucrit2norm	
ggyg	3	m	0.727	36.42	1.183	0.232	hypoxia	2	0	0	Ucrit3norm	
ggyg	3	m	0.727	36.42	1.183	0.232	hypoxia	2	0	0	Ucrit1hyp	61.40412
ggyg	3	m	0.727	36.42	1.183	0.232	hypoxia	2	0	0	Ucrit2hyp	
ggyg	3	m	0.727	36.42	1.183	0.232	hypoxia	2	0	0	Ucrit3hyp	
ggyg	3	m	0.727	36.42	1.183	0.232	normoxia	2	0	0	Ucrit1norm	78.08448
ggyg	3	m	0.727	36.42	1.183	0.232	normoxia	2	0	0	Ucrit2norm	
ggyg	3	m	0.727	36.42	1.183	0.232	normoxia	2	0	0	Ucrit3norm	
ggyg	3	m	0.727	36.42	1.183	0.232	normoxia	2	0	0	Ucrit1hyp	61.40412
ggyg	3	m	0.727	36.42	1.183	0.232	normoxia	2	0	0	Ucrit2hyp	
ggyg	3	m	0.727	36.42	1.183	0.232	normoxia	2	0	0	Ucrit3hyp	
ggyp	2	f	0.735	36.46	1.385	0.232	hypoxia	1	0	0	Ucrit1norm	77.95148
ggyp	2	f	0.735	36.46	1.385	0.232	hypoxia	1	0	0	Ucrit2norm	
ggyp	2	f	0.735	36.46	1.385	0.232	hypoxia	1	0	0	Ucrit3norm	
ggyp	2	f	0.735	36.46	1.385	0.232	hypoxia	1	0	0	Ucrit1hyp	60.01316
ggyp	2	f	0.735	36.46	1.385	0.232	hypoxia	1	0	0	Ucrit2hyp	
ggyp	2	f	0.735	36.46	1.385	0.232	hypoxia	1	0	0	Ucrit3hyp	
ggyp	2	f	0.735	36.46	1.385	0.232	normoxia	1	0	0	Ucrit1norm	77.95148
ggyp	2	f	0.735	36.46	1.385	0.232	normoxia	1	0	0	Ucrit2norm	
ggyp	2	f	0.735	36.46	1.385	0.232	normoxia	1	0	0	Ucrit3norm	
ggyp	2	f	0.735	36.46	1.385	0.232	normoxia	1	0	0	Ucrit1hyp	60.01316
ggyp	2	f	0.735	36.46	1.385	0.232	normoxia	1	0	0	Ucrit2hyp	
ggyp	2	f	0.735	36.46	1.385	0.232	normoxia	1	0	0	Ucrit3hyp	
ggyp	2	f	0.735	36.46	1.385	0.232	hypoxia	2	0	0	Ucrit1norm	77.95148
ggyp	2	f	0.735	36.46	1.385	0.232	hypoxia	2	0	0	Ucrit2norm	
ggyp	2	f	0.735	36.46	1.385	0.232	hypoxia	2	0	0	Ucrit3norm	
ggyp	2	f	0.735	36.46	1.385	0.232	hypoxia	2	0	0	Ucrit1hyp	60.01316

ggyp	2	f	0.735	36.46	1.385	0.232	hypoxia	2	0	0	Ucrit2hyp	
ggyp	2	f	0.735	36.46	1.385	0.232	hypoxia	2	0	0	Ucrit3hyp	
ggyp	2	f	0.735	36.46	1.385	0.232	normoxia	2	0	0	Ucrit1norm	77.95148
ggyp	2	f	0.735	36.46	1.385	0.232	normoxia	2	0	0	Ucrit2norm	
ggyp	2	f	0.735	36.46	1.385	0.232	normoxia	2	0	0	Ucrit3norm	
ggyp	2	f	0.735	36.46	1.385	0.232	normoxia	2	0	0	Ucrit1hyp	60.01316
ggyp	2	f	0.735	36.46	1.385	0.232	normoxia	2	0	0	Ucrit2hyp	
ggyp	2	f	0.735	36.46	1.385	0.232	normoxia	2	0	0	Ucrit3hyp	
ggyr	1	m	0.602	33.41	0.853	0.21	normoxia	1	0	0	Ucrit1norm	95.35214
ggyr	1	m	0.602	33.41	0.853	0.21	normoxia	1	0	0	Ucrit2norm	
ggyr	1	m	0.602	33.41	0.853	0.21	normoxia	1	0	0	Ucrit3norm	
ggyr	1	m	0.602	33.41	0.853	0.21	normoxia	1	0	0	Ucrit1hyp	74.90522
ggyr	1	m	0.602	33.41	0.853	0.21	normoxia	1	0	0	Ucrit2hyp	
ggyr	1	m	0.602	33.41	0.853	0.21	normoxia	1	0	0	Ucrit3hyp	
ggyr	1	m	0.602	33.41	0.853	0.21	hypoxia	1	0	0	Ucrit1norm	95.35214
ggyr	1	m	0.602	33.41	0.853	0.21	hypoxia	1	0	0	Ucrit2norm	
ggyr	1	m	0.602	33.41	0.853	0.21	hypoxia	1	0	0	Ucrit3norm	
ggyr	1	m	0.602	33.41	0.853	0.21	hypoxia	1	0	0	Ucrit1hyp	74.90522
ggyr	1	m	0.602	33.41	0.853	0.21	hypoxia	1	0	0	Ucrit2hyp	
ggyr	1	m	0.602	33.41	0.853	0.21	hypoxia	1	0	0	Ucrit3hyp	
ggyr	1	m	0.602	33.41	0.853	0.21	normoxia	2	0	0	Ucrit1norm	95.35214
ggyr	1	m	0.602	33.41	0.853	0.21	normoxia	2	0	0	Ucrit2norm	
ggyr	1	m	0.602	33.41	0.853	0.21	normoxia	2	0	0	Ucrit3norm	
ggyr	1	m	0.602	33.41	0.853	0.21	normoxia	2	0	0	Ucrit1hyp	74.90522
ggyr	1	m	0.602	33.41	0.853	0.21	normoxia	2	0	0	Ucrit2hyp	
ggyr	1	m	0.602	33.41	0.853	0.21	normoxia	2	0	0	Ucrit3hyp	
ggyr	1	m	0.602	33.41	0.853	0.21	hypoxia	2	0	0	Ucrit1norm	95.35214
ggyr	1	m	0.602	33.41	0.853	0.21	hypoxia	2	0	0	Ucrit2norm	
ggyr	1	m	0.602	33.41	0.853	0.21	hypoxia	2	0	0	Ucrit3norm	
ggyr	1	m	0.602	33.41	0.853	0.21	hypoxia	2	0	0	Ucrit1hyp	74.90522
ggyr	1	m	0.602	33.41	0.853	0.21	hypoxia	2	0	0	Ucrit2hyp	
ggyr	1	m	0.602	33.41	0.853	0.21	hypoxia	2	0	0	Ucrit3hyp	
gp_y	1				1.625	0.263	normoxia	1	0	0		

gp_y	1				1.625	0.263	hypoxia	1	0	0		
gp_y	1				1.625	0.263	normoxia	2	0	0		
gp_y	1				1.625	0.263	hypoxia	2	0	0		
gpgg	2	m	0.533	32.17	1.176	0.152	hypoxia	1	0	0	Ucrit1norm	78.14093
gpgg	2	m	0.533	32.17	1.176	0.152	hypoxia	1	0	0	Ucrit2norm	
gpgg	2	m	0.533	32.17	1.176	0.152	hypoxia	1	0	0	Ucrit3norm	
gpgg	2	m	0.533	32.17	1.176	0.152	hypoxia	1	0	0	Ucrit1hyp	65.24076
gpgg	2	m	0.533	32.17	1.176	0.152	hypoxia	1	0	0	Ucrit2hyp	
gpgg	2	m	0.533	32.17	1.176	0.152	hypoxia	1	0	0	Ucrit3hyp	
gpgg	2	m	0.533	32.17	1.176	0.152	normoxia	1	0	0	Ucrit1norm	78.14093
gpgg	2	m	0.533	32.17	1.176	0.152	normoxia	1	0	0	Ucrit2norm	
gpgg	2	m	0.533	32.17	1.176	0.152	normoxia	1	0	0	Ucrit3norm	
gpgg	2	m	0.533	32.17	1.176	0.152	normoxia	1	0	0	Ucrit1hyp	65.24076
gpgg	2	m	0.533	32.17	1.176	0.152	normoxia	1	0	0	Ucrit2hyp	
gpgg	2	m	0.533	32.17	1.176	0.152	normoxia	1	0	0	Ucrit3hyp	
gpgg	2	m	0.533	32.17	1.176	0.152	hypoxia	2	0	0	Ucrit1norm	78.14093
gpgg	2	m	0.533	32.17	1.176	0.152	hypoxia	2	0	0	Ucrit2norm	
gpgg	2	m	0.533	32.17	1.176	0.152	hypoxia	2	0	0	Ucrit3norm	
gpgg	2	m	0.533	32.17	1.176	0.152	hypoxia	2	0	0	Ucrit1hyp	65.24076
gpgg	2	m	0.533	32.17	1.176	0.152	hypoxia	2	0	0	Ucrit2hyp	
gpgg	2	m	0.533	32.17	1.176	0.152	hypoxia	2	0	0	Ucrit3hyp	
gpgg	2	m	0.533	32.17	1.176	0.152	normoxia	2	0	0	Ucrit1norm	78.14093
gpgg	2	m	0.533	32.17	1.176	0.152	normoxia	2	0	0	Ucrit2norm	
gpgg	2	m	0.533	32.17	1.176	0.152	normoxia	2	0	0	Ucrit3norm	
gpgg	2	m	0.533	32.17	1.176	0.152	normoxia	2	0	0	Ucrit1hyp	65.24076
gpgg	2	m	0.533	32.17	1.176	0.152	normoxia	2	0	0	Ucrit2hyp	
gpgg	2	m	0.533	32.17	1.176	0.152	normoxia	2	0	0	Ucrit3hyp	
gpgp	1	m	0.496	31.89	0.955	0.248	normoxia	1	0	0	Ucrit1norm	76.40844
gpgp	1	m	0.496	31.89	0.955	0.248	normoxia	1	0	0	Ucrit2norm	
gpgp	1	m	0.496	31.89	0.955	0.248	normoxia	1	0	0	Ucrit3norm	
gpgp	1	m	0.496	31.89	0.955	0.248	normoxia	1	0	0	Ucrit1hyp	61.89849
gpgp	1	m	0.496	31.89	0.955	0.248	normoxia	1	0	0	Ucrit2hyp	
gpgp	1	m	0.496	31.89	0.955	0.248	normoxia	1	0	0	Ucrit3hyp	

gpgp	1	m	0.496	31.89	0.955	0.248	hypoxia	1	0.786666667	1	Ucrit1norm	76.40844
gpgp	1	m	0.496	31.89	0.955	0.248	hypoxia	1	0.786666667	1	Ucrit2norm	
gpgp	1	m	0.496	31.89	0.955	0.248	hypoxia	1	0.786666667	1	Ucrit3norm	
gpgp	1	m	0.496	31.89	0.955	0.248	hypoxia	1	0.786666667	1	Ucrit1hyp	61.89849
gpgp	1	m	0.496	31.89	0.955	0.248	hypoxia	1	0.786666667	1	Ucrit2hyp	
gpgp	1	m	0.496	31.89	0.955	0.248	hypoxia	1	0.786666667	1	Ucrit3hyp	
gpgp	1	m	0.496	31.89	0.955	0.248	normoxia	2	0	0	Ucrit1norm	76.40844
gpgp	1	m	0.496	31.89	0.955	0.248	normoxia	2	0	0	Ucrit2norm	
gpgp	1	m	0.496	31.89	0.955	0.248	normoxia	2	0	0	Ucrit3norm	
gpgp	1	m	0.496	31.89	0.955	0.248	normoxia	2	0	0	Ucrit1hyp	61.89849
gpgp	1	m	0.496	31.89	0.955	0.248	normoxia	2	0	0	Ucrit2hyp	
gpgp	1	m	0.496	31.89	0.955	0.248	normoxia	2	0	0	Ucrit3hyp	
gpgp	1	m	0.496	31.89	0.955	0.248	hypoxia	2	0	0	Ucrit1norm	76.40844
gpgp	1	m	0.496	31.89	0.955	0.248	hypoxia	2	0	0	Ucrit2norm	
gpgp	1	m	0.496	31.89	0.955	0.248	hypoxia	2	0	0	Ucrit3norm	
gpgp	1	m	0.496	31.89	0.955	0.248	hypoxia	2	0	0	Ucrit1hyp	61.89849
gpgp	1	m	0.496	31.89	0.955	0.248	hypoxia	2	0	0	Ucrit2hyp	
gpgp	1	m	0.496	31.89	0.955	0.248	hypoxia	2	0	0	Ucrit3hyp	
gpgr	2	f	0.54	32.84	1.319	0.251	hypoxia	1	0	0	Ucrit1norm	77.73228
gpgr	2	f	0.54	32.84	1.319	0.251	hypoxia	1	0	0	Ucrit2norm	
gpgr	2	f	0.54	32.84	1.319	0.251	hypoxia	1	0	0	Ucrit3norm	
gpgr	2	f	0.54	32.84	1.319	0.251	hypoxia	1	0	0	Ucrit1hyp	60.29424
gpgr	2	f	0.54	32.84	1.319	0.251	hypoxia	1	0	0	Ucrit2hyp	
gpgr	2	f	0.54	32.84	1.319	0.251	hypoxia	1	0	0	Ucrit3hyp	
gpgr	2	f	0.54	32.84	1.319	0.251	normoxia	1	0	0	Ucrit1norm	77.73228
gpgr	2	f	0.54	32.84	1.319	0.251	normoxia	1	0	0	Ucrit2norm	
gpgr	2	f	0.54	32.84	1.319	0.251	normoxia	1	0	0	Ucrit3norm	
gpgr	2	f	0.54	32.84	1.319	0.251	normoxia	1	0	0	Ucrit1hyp	60.29424
gpgr	2	f	0.54	32.84	1.319	0.251	normoxia	1	0	0	Ucrit2hyp	
gpgr	2	f	0.54	32.84	1.319	0.251	normoxia	1	0	0	Ucrit3hyp	
gpgr	2	f	0.54	32.84	1.319	0.251	hypoxia	2	0	0	Ucrit1norm	77.73228
gpgr	2	f	0.54	32.84	1.319	0.251	hypoxia	2	0	0	Ucrit2norm	
gpgr	2	f	0.54	32.84	1.319	0.251	hypoxia	2	0	0	Ucrit3norm	

gpgr	2	f	0.54	32.84	1.319	0.251	hypoxia	2	0	0	Ucrit1hyp	60.29424
gpgr	2	f	0.54	32.84	1.319	0.251	hypoxia	2	0	0	Ucrit2hyp	
gpgr	2	f	0.54	32.84	1.319	0.251	hypoxia	2	0	0	Ucrit3hyp	
gpgr	2	f	0.54	32.84	1.319	0.251	normoxia	2	0	0	Ucrit1norm	77.73228
gpgr	2	f	0.54	32.84	1.319	0.251	normoxia	2	0	0	Ucrit2norm	
gpgr	2	f	0.54	32.84	1.319	0.251	normoxia	2	0	0	Ucrit3norm	
gpgr	2	f	0.54	32.84	1.319	0.251	normoxia	2	0	0	Ucrit1hyp	60.29424
gpgr	2	f	0.54	32.84	1.319	0.251	normoxia	2	0	0	Ucrit2hyp	
gpgr	2	f	0.54	32.84	1.319	0.251	normoxia	2	0	0	Ucrit3hyp	
gpgy	4	f	0.686	33.49	0.978	0.199	normoxia	1	0	0	Ucrit1norm	71.73558
gpgy	4	f	0.686	33.49	0.978	0.199	normoxia	1	0	0	Ucrit2norm	71.3337
gpgy	4	f	0.686	33.49	0.978	0.199	normoxia	1	0	0	Ucrit3norm	71.73558
gpgy	4	f	0.686	33.49	0.978	0.199	normoxia	1	0	0	Ucrit1hyp	57.23441
gpgy	4	f	0.686	33.49	0.978	0.199	normoxia	1	0	0	Ucrit2hyp	60.58341
gpgy	4	f	0.686	33.49	0.978	0.199	normoxia	1	0	0	Ucrit3hyp	67.3149
gpgy	4	f	0.686	33.49	0.978	0.199	hypoxia	1	0	0	Ucrit1norm	71.73558
gpgy	4	f	0.686	33.49	0.978	0.199	hypoxia	1	0	0	Ucrit2norm	71.3337
gpgy	4	f	0.686	33.49	0.978	0.199	hypoxia	1	0	0	Ucrit3norm	71.73558
gpgy	4	f	0.686	33.49	0.978	0.199	hypoxia	1	0	0	Ucrit1hyp	57.23441
gpgy	4	f	0.686	33.49	0.978	0.199	hypoxia	1	0	0	Ucrit2hyp	60.58341
gpgy	4	f	0.686	33.49	0.978	0.199	hypoxia	1	0	0	Ucrit3hyp	67.3149
gpgy	4	f	0.686	33.49	0.978	0.199	normoxia	2	0	0	Ucrit1norm	71.73558
gpgy	4	f	0.686	33.49	0.978	0.199	normoxia	2	0	0	Ucrit2norm	71.3337
gpgy	4	f	0.686	33.49	0.978	0.199	normoxia	2	0	0	Ucrit3norm	71.73558
gpgy	4	f	0.686	33.49	0.978	0.199	normoxia	2	0	0	Ucrit1hyp	57.23441
gpgy	4	f	0.686	33.49	0.978	0.199	normoxia	2	0	0	Ucrit2hyp	60.58341
gpgy	4	f	0.686	33.49	0.978	0.199	normoxia	2	0	0	Ucrit3hyp	67.3149
gpgy	4	f	0.686	33.49	0.978	0.199	hypoxia	2	0	0	Ucrit1norm	71.73558
gpgy	4	f	0.686	33.49	0.978	0.199	hypoxia	2	0	0	Ucrit2norm	71.3337
gpgy	4	f	0.686	33.49	0.978	0.199	hypoxia	2	0	0	Ucrit3norm	71.73558
gpgy	4	f	0.686	33.49	0.978	0.199	hypoxia	2	0	0	Ucrit1hyp	57.23441
gpgy	4	f	0.686	33.49	0.978	0.199	hypoxia	2	0	0	Ucrit2hyp	60.58341
gpgy	4	f	0.686	33.49	0.978	0.199	hypoxia	2	0	0	Ucrit3hyp	67.3149

gpyg	3	m	0.462	33.66	1.365	0.181	hypoxia	1	0	0	Ucrit1norm	86.64084
gpyg	3	m	0.462	33.66	1.365	0.181	hypoxia	1	0	0	Ucrit2norm	
gpyg	3	m	0.462	33.66	1.365	0.181	hypoxia	1	0	0	Ucrit3norm	
gpyg	3	m	0.462	33.66	1.365	0.181	hypoxia	1	0	0	Ucrit1hyp	81.15426
gpyg	3	m	0.462	33.66	1.365	0.181	hypoxia	1	0	0	Ucrit2hyp	
gpyg	3	m	0.462	33.66	1.365	0.181	hypoxia	1	0	0	Ucrit3hyp	
gpyg	3	m	0.462	33.66	1.365	0.181	normoxia	1	0	0	Ucrit1norm	86.64084
gpyg	3	m	0.462	33.66	1.365	0.181	normoxia	1	0	0	Ucrit2norm	
gpyg	3	m	0.462	33.66	1.365	0.181	normoxia	1	0	0	Ucrit3norm	
gpyg	3	m	0.462	33.66	1.365	0.181	normoxia	1	0	0	Ucrit1hyp	81.15426
gpyg	3	m	0.462	33.66	1.365	0.181	normoxia	1	0	0	Ucrit2hyp	
gpyg	3	m	0.462	33.66	1.365	0.181	normoxia	1	0	0	Ucrit3hyp	
gpyg	3	m	0.462	33.66	1.365	0.181	hypoxia	2	0	0	Ucrit1norm	86.64084
gpyg	3	m	0.462	33.66	1.365	0.181	hypoxia	2	0	0	Ucrit2norm	
gpyg	3	m	0.462	33.66	1.365	0.181	hypoxia	2	0	0	Ucrit3norm	
gpyg	3	m	0.462	33.66	1.365	0.181	hypoxia	2	0	0	Ucrit1hyp	81.15426
gpyg	3	m	0.462	33.66	1.365	0.181	hypoxia	2	0	0	Ucrit2hyp	
gpyg	3	m	0.462	33.66	1.365	0.181	hypoxia	2	0	0	Ucrit3hyp	
gpyg	3	m	0.462	33.66	1.365	0.181	normoxia	2	0	0	Ucrit1norm	86.64084
gpyg	3	m	0.462	33.66	1.365	0.181	normoxia	2	0	0	Ucrit2norm	
gpyg	3	m	0.462	33.66	1.365	0.181	normoxia	2	0	0	Ucrit3norm	
gpyg	3	m	0.462	33.66	1.365	0.181	normoxia	2	0	0	Ucrit1hyp	81.15426
gpyg	3	m	0.462	33.66	1.365	0.181	normoxia	2	0	0	Ucrit2hyp	
gpyg	3	m	0.462	33.66	1.365	0.181	normoxia	2	0	0	Ucrit3hyp	
gpyr	3	f	0.576	33.28	1.227	0.175	hypoxia	1	0	0	Ucrit1norm	79.57248
gpyr	3	f	0.576	33.28	1.227	0.175	hypoxia	1	0	0	Ucrit2norm	
gpyr	3	f	0.576	33.28	1.227	0.175	hypoxia	1	0	0	Ucrit3norm	
gpyr	3	f	0.576	33.28	1.227	0.175	hypoxia	1	0	0	Ucrit1hyp	61.10208
gpyr	3	f	0.576	33.28	1.227	0.175	hypoxia	1	0	0	Ucrit2hyp	
gpyr	3	f	0.576	33.28	1.227	0.175	hypoxia	1	0	0	Ucrit3hyp	
gpyr	3	f	0.576	33.28	1.227	0.175	normoxia	1	0	0	Ucrit1norm	79.57248
gpyr	3	f	0.576	33.28	1.227	0.175	normoxia	1	0	0	Ucrit2norm	
gpyr	3	f	0.576	33.28	1.227	0.175	normoxia	1	0	0	Ucrit3norm	

gpyr	3	f	0.576	33.28	1.227	0.175	normoxia	1	0	0	Ucrit1hyp	61.10208
gpyr	3	f	0.576	33.28	1.227	0.175	normoxia	1	0	0	Ucrit2hyp	
gpyr	3	f	0.576	33.28	1.227	0.175	normoxia	1	0	0	Ucrit3hyp	
gpyr	3	f	0.576	33.28	1.227	0.175	hypoxia	2	0	0	Ucrit1norm	79.57248
gpyr	3	f	0.576	33.28	1.227	0.175	hypoxia	2	0	0	Ucrit2norm	
gpyr	3	f	0.576	33.28	1.227	0.175	hypoxia	2	0	0	Ucrit3norm	
gpyr	3	f	0.576	33.28	1.227	0.175	hypoxia	2	0	0	Ucrit1hyp	61.10208
gpyr	3	f	0.576	33.28	1.227	0.175	hypoxia	2	0	0	Ucrit2hyp	
gpyr	3	f	0.576	33.28	1.227	0.175	hypoxia	2	0	0	Ucrit3hyp	
gpyr	3	f	0.576	33.28	1.227	0.175	normoxia	2	0.401666667	1	Ucrit1norm	79.57248
gpyr	3	f	0.576	33.28	1.227	0.175	normoxia	2	0.401666667	1	Ucrit2norm	
gpyr	3	f	0.576	33.28	1.227	0.175	normoxia	2	0.401666667	1	Ucrit3norm	
gpyr	3	f	0.576	33.28	1.227	0.175	normoxia	2	0.401666667	1	Ucrit1hyp	61.10208
gpyr	3	f	0.576	33.28	1.227	0.175	normoxia	2	0.401666667	1	Ucrit2hyp	
gpyr	3	f	0.576	33.28	1.227	0.175	normoxia	2	0.401666667	1	Ucrit3hyp	
grgy	4	f	0.79	36.82	1.326	0.257	normoxia	1	0	0	Ucrit1norm	80.04668
grgy	4	f	0.79	36.82	1.326	0.257	normoxia	1	0	0	Ucrit2norm	79.78894
grgy	4	f	0.79	36.82	1.326	0.257	normoxia	1	0	0	Ucrit3norm	79.45756
grgy	4	f	0.79	36.82	1.326	0.257	normoxia	1	0	0	Ucrit1hyp	61.04756
grgy	4	f	0.79	36.82	1.326	0.257	normoxia	1	0	0	Ucrit2hyp	87.59478
grgy	4	f	0.79	36.82	1.326	0.257	normoxia	1	0	0	Ucrit3hyp	68.55884
grgy	4	f	0.79	36.82	1.326	0.257	hypoxia	1	0.9	1	Ucrit1norm	80.04668
grgy	4	f	0.79	36.82	1.326	0.257	hypoxia	1	0.9	1	Ucrit2norm	79.78894
grgy	4	f	0.79	36.82	1.326	0.257	hypoxia	1	0.9	1	Ucrit3norm	79.45756
grgy	4	f	0.79	36.82	1.326	0.257	hypoxia	1	0.9	1	Ucrit1hyp	61.04756
grgy	4	f	0.79	36.82	1.326	0.257	hypoxia	1	0.9	1	Ucrit2hyp	87.59478
grgy	4	f	0.79	36.82	1.326	0.257	hypoxia	1	0.9	1	Ucrit3hyp	68.55884
grgy	4	f	0.79	36.82	1.326	0.257	normoxia	2	0	0	Ucrit1norm	80.04668
grgy	4	f	0.79	36.82	1.326	0.257	normoxia	2	0	0	Ucrit2norm	79.78894
grgy	4	f	0.79	36.82	1.326	0.257	normoxia	2	0	0	Ucrit3norm	79.45756
grgy	4	f	0.79	36.82	1.326	0.257	normoxia	2	0	0	Ucrit1hyp	61.04756
grgy	4	f	0.79	36.82	1.326	0.257	normoxia	2	0	0	Ucrit2hyp	87.59478
grgy	4	f	0.79	36.82	1.326	0.257	normoxia	2	0	0	Ucrit3hyp	68.55884

grgy	4	f	0.79	36.82	1.326	0.257	hypoxia	2	0	0	Ucrit1norm	80.04668
grgy	4	f	0.79	36.82	1.326	0.257	hypoxia	2	0	0	Ucrit2norm	79.78894
grgy	4	f	0.79	36.82	1.326	0.257	hypoxia	2	0	0	Ucrit3norm	79.45756
grgy	4	f	0.79	36.82	1.326	0.257	hypoxia	2	0	0	Ucrit1hyp	61.04756
grgy	4	f	0.79	36.82	1.326	0.257	hypoxia	2	0	0	Ucrit2hyp	87.59478
grgy	4	f	0.79	36.82	1.326	0.257	hypoxia	2	0	0	Ucrit3hyp	68.55884
grpp	4	m	0.49	31.8			normoxia	1	0	0	Ucrit1norm	82.68
grpp	4	m	0.49	31.8			normoxia	1	0	0	Ucrit2norm	89.1036
grpp	4	m	0.49	31.8			normoxia	1	0	0	Ucrit3norm	89.1036
grpp	4	m	0.49	31.8			normoxia	1	0	0	Ucrit1hyp	69.3876
grpp	4	m	0.49	31.8			normoxia	1	0	0	Ucrit2hyp	67.1934
grpp	4	m	0.49	31.8			normoxia	1	0	0	Ucrit3hyp	66.5892
grpp	4	m	0.49	31.8			hypoxia	1	0	0	Ucrit1norm	82.68
grpp	4	m	0.49	31.8			hypoxia	1	0	0	Ucrit2norm	89.1036
grpp	4	m	0.49	31.8			hypoxia	1	0	0	Ucrit3norm	89.1036
grpp	4	m	0.49	31.8			hypoxia	1	0	0	Ucrit1hyp	69.3876
grpp	4	m	0.49	31.8			hypoxia	1	0	0	Ucrit2hyp	67.1934
grpp	4	m	0.49	31.8			hypoxia	1	0	0	Ucrit3hyp	66.5892
grpp	4	m	0.49	31.8			normoxia	2	0	0	Ucrit1norm	82.68
grpp	4	m	0.49	31.8			normoxia	2	0	0	Ucrit2norm	89.1036
grpp	4	m	0.49	31.8			normoxia	2	0	0	Ucrit3norm	89.1036
grpp	4	m	0.49	31.8			normoxia	2	0	0	Ucrit1hyp	69.3876
grpp	4	m	0.49	31.8			normoxia	2	0	0	Ucrit2hyp	67.1934
grpp	4	m	0.49	31.8			normoxia	2	0	0	Ucrit3hyp	66.5892
grpp	4	m	0.49	31.8			hypoxia	2	0	0	Ucrit1norm	82.68
grpp	4	m	0.49	31.8			hypoxia	2	0	0	Ucrit2norm	89.1036
grpp	4	m	0.49	31.8			hypoxia	2	0	0	Ucrit3norm	89.1036
grpp	4	m	0.49	31.8			hypoxia	2	0	0	Ucrit1hyp	69.3876
grpp	4	m	0.49	31.8			hypoxia	2	0	0	Ucrit2hyp	67.1934
grpp	4	m	0.49	31.8			hypoxia	2	0	0	Ucrit3hyp	66.5892
grpy	4	f	0.595	32.31	0.873	0.18	normoxia	1	0	0	Ucrit1norm	77.25321
grpy	4	f	0.595	32.31	0.873	0.18	normoxia	1	0	0	Ucrit2norm	81.0981
grpy	4	f	0.595	32.31	0.873	0.18	normoxia	1	0	0	Ucrit3norm	77.60862

grpy	4	f	0.595	32.31	0.873	0.18	normoxia	1	0	0	Ucrit1hyp	66.84939
grpy	4	f	0.595	32.31	0.873	0.18	normoxia	1	0	0	Ucrit2hyp	65.42775
grpy	4	f	0.595	32.31	0.873	0.18	normoxia	1	0	0	Ucrit3hyp	72.47133
grpy	4	f	0.595	32.31	0.873	0.18	hypoxia	1	0	0	Ucrit1norm	77.25321
grpy	4	f	0.595	32.31	0.873	0.18	hypoxia	1	0	0	Ucrit2norm	81.0981
grpy	4	f	0.595	32.31	0.873	0.18	hypoxia	1	0	0	Ucrit3norm	77.60862
grpy	4	f	0.595	32.31	0.873	0.18	hypoxia	1	0	0	Ucrit1hyp	66.84939
grpy	4	f	0.595	32.31	0.873	0.18	hypoxia	1	0	0	Ucrit2hyp	65.42775
grpy	4	f	0.595	32.31	0.873	0.18	hypoxia	1	0	0	Ucrit3hyp	72.47133
grpy	4	f	0.595	32.31	0.873	0.18	normoxia	2	0	0	Ucrit1norm	77.25321
grpy	4	f	0.595	32.31	0.873	0.18	normoxia	2	0	0	Ucrit2norm	81.0981
grpy	4	f	0.595	32.31	0.873	0.18	normoxia	2	0	0	Ucrit3norm	77.60862
grpy	4	f	0.595	32.31	0.873	0.18	normoxia	2	0	0	Ucrit1hyp	66.84939
grpy	4	f	0.595	32.31	0.873	0.18	normoxia	2	0	0	Ucrit2hyp	65.42775
grpy	4	f	0.595	32.31	0.873	0.18	normoxia	2	0	0	Ucrit3hyp	72.47133
grpy	4	f	0.595	32.31	0.873	0.18	hypoxia	2	0.84	1	Ucrit1norm	77.25321
grpy	4	f	0.595	32.31	0.873	0.18	hypoxia	2	0.84	1	Ucrit2norm	81.0981
grpy	4	f	0.595	32.31	0.873	0.18	hypoxia	2	0.84	1	Ucrit3norm	77.60862
grpy	4	f	0.595	32.31	0.873	0.18	hypoxia	2	0.84	1	Ucrit1hyp	66.84939
grpy	4	f	0.595	32.31	0.873	0.18	hypoxia	2	0.84	1	Ucrit2hyp	65.42775
grpy	4	f	0.595	32.31	0.873	0.18	hypoxia	2	0.84	1	Ucrit3hyp	72.47133
grrg	3	m	0.556	33.1			hypoxia	1	0	0	Ucrit1norm	78.9104
grrg	3	m	0.556	33.1			hypoxia	1	0	0	Ucrit2norm	
grrg	3	m	0.556	33.1			hypoxia	1	0	0	Ucrit3norm	
grrg	3	m	0.556	33.1			hypoxia	1	0	0	Ucrit1hyp	
grrg	3	m	0.556	33.1			hypoxia	1	0	0	Ucrit2hyp	
grrg	3	m	0.556	33.1			hypoxia	1	0	0	Ucrit3hyp	
grrg	3	m	0.556	33.1			normoxia	1	0	0	Ucrit1norm	78.9104
grrg	3	m	0.556	33.1			normoxia	1	0	0	Ucrit2norm	
grrg	3	m	0.556	33.1			normoxia	1	0	0	Ucrit3norm	
grrg	3	m	0.556	33.1			normoxia	1	0	0	Ucrit1hyp	
grrg	3	m	0.556	33.1			normoxia	1	0	0	Ucrit2hyp	
grrg	3	m	0.556	33.1			normoxia	1	0	0	Ucrit3hyp	

grrg	3	m	0.556	33.1			hypoxia	2	0	0	Ucrit1norm	78.9104
grrg	3	m	0.556	33.1			hypoxia	2	0	0	Ucrit2norm	
grrg	3	m	0.556	33.1			hypoxia	2	0	0	Ucrit3norm	
grrg	3	m	0.556	33.1			hypoxia	2	0	0	Ucrit1hyp	
grrg	3	m	0.556	33.1			hypoxia	2	0	0	Ucrit2hyp	
grrg	3	m	0.556	33.1			hypoxia	2	0	0	Ucrit3hyp	
grrg	3	m	0.556	33.1			normoxia	2	0	0	Ucrit1norm	78.9104
grrg	3	m	0.556	33.1			normoxia	2	0	0	Ucrit2norm	
grrg	3	m	0.556	33.1			normoxia	2	0	0	Ucrit3norm	
grrg	3	m	0.556	33.1			normoxia	2	0	0	Ucrit1hyp	
grrg	3	m	0.556	33.1			normoxia	2	0	0	Ucrit2hyp	
grrg	3	m	0.556	33.1			normoxia	2	0	0	Ucrit3hyp	
grrp	4	m	0.26	26.7	0.598	0.132	normoxia	1	0	0	Ucrit1norm	82.6632
grrp	4	m	0.26	26.7	0.598	0.132	normoxia	1	0	0	Ucrit2norm	78.3645
grrp	4	m	0.26	26.7	0.598	0.132	normoxia	1	0	0	Ucrit3norm	76.4421
grrp	4	m	0.26	26.7	0.598	0.132	normoxia	1	0	0	Ucrit1hyp	63.6795
grrp	4	m	0.26	26.7	0.598	0.132	normoxia	1	0	0	Ucrit2hyp	64.4538
grrp	4	m	0.26	26.7	0.598	0.132	normoxia	1	0	0	Ucrit3hyp	78.231
grrp	4	m	0.26	26.7	0.598	0.132	hypoxia	1	0	0	Ucrit1norm	82.6632
grrp	4	m	0.26	26.7	0.598	0.132	hypoxia	1	0	0	Ucrit2norm	78.3645
grrp	4	m	0.26	26.7	0.598	0.132	hypoxia	1	0	0	Ucrit3norm	76.4421
grrp	4	m	0.26	26.7	0.598	0.132	hypoxia	1	0	0	Ucrit1hyp	63.6795
grrp	4	m	0.26	26.7	0.598	0.132	hypoxia	1	0	0	Ucrit2hyp	64.4538
grrp	4	m	0.26	26.7	0.598	0.132	hypoxia	1	0	0	Ucrit3hyp	78.231
grrp	4	m	0.26	26.7	0.598	0.132	normoxia	2	0	0	Ucrit1norm	82.6632
grrp	4	m	0.26	26.7	0.598	0.132	normoxia	2	0	0	Ucrit2norm	78.3645
grrp	4	m	0.26	26.7	0.598	0.132	normoxia	2	0	0	Ucrit3norm	76.4421
grrp	4	m	0.26	26.7	0.598	0.132	normoxia	2	0	0	Ucrit1hyp	63.6795
grrp	4	m	0.26	26.7	0.598	0.132	normoxia	2	0	0	Ucrit2hyp	64.4538
grrp	4	m	0.26	26.7	0.598	0.132	normoxia	2	0	0	Ucrit3hyp	78.231
grrp	4	m	0.26	26.7	0.598	0.132	hypoxia	2	0	0	Ucrit1norm	82.6632
grrp	4	m	0.26	26.7	0.598	0.132	hypoxia	2	0	0	Ucrit2norm	78.3645
grrp	4	m	0.26	26.7	0.598	0.132	hypoxia	2	0	0	Ucrit3norm	76.4421

grrp	4	m	0.26	26.7	0.598	0.132	hypoxia	2	0	0	Ucrit1hyp	63.6795
grrp	4	m	0.26	26.7	0.598	0.132	hypoxia	2	0	0	Ucrit2hyp	64.4538
grrp	4	m	0.26	26.7	0.598	0.132	hypoxia	2	0	0	Ucrit3hyp	78.231
grrr	3	f	0.612	32.95	0.985	0.34	hypoxia	1	0	0	Ucrit1norm	71.33675
grrr	3	f	0.612	32.95	0.985	0.34	hypoxia	1	0	0	Ucrit2norm	
grrr	3	f	0.612	32.95	0.985	0.34	hypoxia	1	0	0	Ucrit3norm	
grrr	3	f	0.612	32.95	0.985	0.34	hypoxia	1	0	0	Ucrit1hyp	58.68395
grrr	3	f	0.612	32.95	0.985	0.34	hypoxia	1	0	0	Ucrit2hyp	
grrr	3	f	0.612	32.95	0.985	0.34	hypoxia	1	0	0	Ucrit3hyp	
grrr	3	f	0.612	32.95	0.985	0.34	normoxia	1	0	0	Ucrit1norm	71.33675
grrr	3	f	0.612	32.95	0.985	0.34	normoxia	1	0	0	Ucrit2norm	
grrr	3	f	0.612	32.95	0.985	0.34	normoxia	1	0	0	Ucrit3norm	
grrr	3	f	0.612	32.95	0.985	0.34	normoxia	1	0	0	Ucrit1hyp	58.68395
grrr	3	f	0.612	32.95	0.985	0.34	normoxia	1	0	0	Ucrit2hyp	
grrr	3	f	0.612	32.95	0.985	0.34	normoxia	1	0	0	Ucrit3hyp	
grrr	3	f	0.612	32.95	0.985	0.34	hypoxia	2	0	0	Ucrit1norm	71.33675
grrr	3	f	0.612	32.95	0.985	0.34	hypoxia	2	0	0	Ucrit2norm	
grrr	3	f	0.612	32.95	0.985	0.34	hypoxia	2	0	0	Ucrit3norm	
grrr	3	f	0.612	32.95	0.985	0.34	hypoxia	2	0	0	Ucrit1hyp	58.68395
grrr	3	f	0.612	32.95	0.985	0.34	hypoxia	2	0	0	Ucrit2hyp	
grrr	3	f	0.612	32.95	0.985	0.34	hypoxia	2	0	0	Ucrit3hyp	
grrr	3	f	0.612	32.95	0.985	0.34	normoxia	2	0	0	Ucrit1norm	71.33675
grrr	3	f	0.612	32.95	0.985	0.34	normoxia	2	0	0	Ucrit2norm	
grrr	3	f	0.612	32.95	0.985	0.34	normoxia	2	0	0	Ucrit3norm	
grrr	3	f	0.612	32.95	0.985	0.34	normoxia	2	0	0	Ucrit1hyp	58.68395
grrr	3	f	0.612	32.95	0.985	0.34	normoxia	2	0	0	Ucrit2hyp	
grrr	3	f	0.612	32.95	0.985	0.34	normoxia	2	0	0	Ucrit3hyp	
grry	2	f	0.636	33.54	1.31	0.238	hypoxia	1	0	0	Ucrit1norm	85.42638
grry	2	f	0.636	33.54	1.31	0.238	hypoxia	1	0	0	Ucrit2norm	
grry	2	f	0.636	33.54	1.31	0.238	hypoxia	1	0	0	Ucrit3norm	
grry	2	f	0.636	33.54	1.31	0.238	hypoxia	1	0	0	Ucrit1hyp	59.7012
grry	2	f	0.636	33.54	1.31	0.238	hypoxia	1	0	0	Ucrit2hyp	
grry	2	f	0.636	33.54	1.31	0.238	hypoxia	1	0	0	Ucrit3hyp	

grry	2	f	0.636	33.54	1.31	0.238	normoxia	1	0	0	Ucrit1norm	85.42638
grry	2	f	0.636	33.54	1.31	0.238	normoxia	1	0	0	Ucrit2norm	
grry	2	f	0.636	33.54	1.31	0.238	normoxia	1	0	0	Ucrit3norm	
grry	2	f	0.636	33.54	1.31	0.238	normoxia	1	0	0	Ucrit1hyp	59.7012
grry	2	f	0.636	33.54	1.31	0.238	normoxia	1	0	0	Ucrit2hyp	
grry	2	f	0.636	33.54	1.31	0.238	normoxia	1	0	0	Ucrit3hyp	
grry	2	f	0.636	33.54	1.31	0.238	hypoxia	2	0.835	1	Ucrit1norm	85.42638
grry	2	f	0.636	33.54	1.31	0.238	hypoxia	2	0.835	1	Ucrit2norm	
grry	2	f	0.636	33.54	1.31	0.238	hypoxia	2	0.835	1	Ucrit3norm	
grry	2	f	0.636	33.54	1.31	0.238	hypoxia	2	0.835	1	Ucrit1hyp	59.7012
grry	2	f	0.636	33.54	1.31	0.238	hypoxia	2	0.835	1	Ucrit2hyp	
grry	2	f	0.636	33.54	1.31	0.238	hypoxia	2	0.835	1	Ucrit3hyp	
grry	2	f	0.636	33.54	1.31	0.238	normoxia	2	0	0	Ucrit1norm	85.42638
grry	2	f	0.636	33.54	1.31	0.238	normoxia	2	0	0	Ucrit2norm	
grry	2	f	0.636	33.54	1.31	0.238	normoxia	2	0	0	Ucrit3norm	
grry	2	f	0.636	33.54	1.31	0.238	normoxia	2	0	0	Ucrit1hyp	59.7012
grry	2	f	0.636	33.54	1.31	0.238	normoxia	2	0	0	Ucrit2hyp	
grry	2	f	0.636	33.54	1.31	0.238	normoxia	2	0	0	Ucrit3hyp	
gryg	1	m	0.504	30.39	1.396	0.211	normoxia	1	0	0	Ucrit1norm	97.67346
gryg	1	m	0.504	30.39	1.396	0.211	normoxia	1	0	0	Ucrit2norm	
gryg	1	m	0.504	30.39	1.396	0.211	normoxia	1	0	0	Ucrit3norm	
gryg	1	m	0.504	30.39	1.396	0.211	normoxia	1	0	0	Ucrit1hyp	70.71753
gryg	1	m	0.504	30.39	1.396	0.211	normoxia	1	0	0	Ucrit2hyp	
gryg	1	m	0.504	30.39	1.396	0.211	normoxia	1	0	0	Ucrit3hyp	
gryg	1	m	0.504	30.39	1.396	0.211	hypoxia	1	0.73	1	Ucrit1norm	97.67346
gryg	1	m	0.504	30.39	1.396	0.211	hypoxia	1	0.73	1	Ucrit2norm	
gryg	1	m	0.504	30.39	1.396	0.211	hypoxia	1	0.73	1	Ucrit3norm	
gryg	1	m	0.504	30.39	1.396	0.211	hypoxia	1	0.73	1	Ucrit1hyp	70.71753
gryg	1	m	0.504	30.39	1.396	0.211	hypoxia	1	0.73	1	Ucrit2hyp	
gryg	1	m	0.504	30.39	1.396	0.211	hypoxia	1	0.73	1	Ucrit3hyp	
gryg	1	m	0.504	30.39	1.396	0.211	normoxia	2	0	0	Ucrit1norm	97.67346
gryg	1	m	0.504	30.39	1.396	0.211	normoxia	2	0	0	Ucrit2norm	
gryg	1	m	0.504	30.39	1.396	0.211	normoxia	2	0	0	Ucrit3norm	

gryg	1	m	0.504	30.39	1.396	0.211	normoxia	2	0	0	Ucrit1hyp	70.71753
gryg	1	m	0.504	30.39	1.396	0.211	normoxia	2	0	0	Ucrit2hyp	
gryg	1	m	0.504	30.39	1.396	0.211	normoxia	2	0	0	Ucrit3hyp	
gryg	1	m	0.504	30.39	1.396	0.211	hypoxia	2	0	0	Ucrit1norm	97.67346
gryg	1	m	0.504	30.39	1.396	0.211	hypoxia	2	0	0	Ucrit2norm	
gryg	1	m	0.504	30.39	1.396	0.211	hypoxia	2	0	0	Ucrit3norm	
gryg	1	m	0.504	30.39	1.396	0.211	hypoxia	2	0	0	Ucrit1hyp	70.71753
gryg	1	m	0.504	30.39	1.396	0.211	hypoxia	2	0	0	Ucrit2hyp	
gryg	1	m	0.504	30.39	1.396	0.211	hypoxia	2	0	0	Ucrit3hyp	
gryp	1	m	0.507	34.05	1.056	0.135	normoxia	1	0	0	Ucrit1norm	75.3186
gryp	1	m	0.507	34.05	1.056	0.135	normoxia	1	0	0	Ucrit2norm	
gryp	1	m	0.507	34.05	1.056	0.135	normoxia	1	0	0	Ucrit3norm	
gryp	1	m	0.507	34.05	1.056	0.135	normoxia	1	0	0	Ucrit1hyp	75.0462
gryp	1	m	0.507	34.05	1.056	0.135	normoxia	1	0	0	Ucrit2hyp	
gryp	1	m	0.507	34.05	1.056	0.135	normoxia	1	0	0	Ucrit3hyp	
gryp	1	m	0.507	34.05	1.056	0.135	hypoxia	1	0.768333333	1	Ucrit1norm	75.3186
gryp	1	m	0.507	34.05	1.056	0.135	hypoxia	1	0.768333333	1	Ucrit2norm	
gryp	1	m	0.507	34.05	1.056	0.135	hypoxia	1	0.768333333	1	Ucrit3norm	
gryp	1	m	0.507	34.05	1.056	0.135	hypoxia	1	0.768333333	1	Ucrit1hyp	75.0462
gryp	1	m	0.507	34.05	1.056	0.135	hypoxia	1	0.768333333	1	Ucrit2hyp	
gryp	1	m	0.507	34.05	1.056	0.135	hypoxia	1	0.768333333	1	Ucrit3hyp	
gryp	1	m	0.507	34.05	1.056	0.135	normoxia	2	0	0	Ucrit1norm	75.3186
gryp	1	m	0.507	34.05	1.056	0.135	normoxia	2	0	0	Ucrit2norm	
gryp	1	m	0.507	34.05	1.056	0.135	normoxia	2	0	0	Ucrit3norm	
gryp	1	m	0.507	34.05	1.056	0.135	normoxia	2	0	0	Ucrit1hyp	75.0462
gryp	1	m	0.507	34.05	1.056	0.135	normoxia	2	0	0	Ucrit2hyp	
gryp	1	m	0.507	34.05	1.056	0.135	normoxia	2	0	0	Ucrit3hyp	
gryp	1	m	0.507	34.05	1.056	0.135	hypoxia	2	0.87	1	Ucrit1norm	75.3186
gryp	1	m	0.507	34.05	1.056	0.135	hypoxia	2	0.87	1	Ucrit2norm	
gryp	1	m	0.507	34.05	1.056	0.135	hypoxia	2	0.87	1	Ucrit3norm	
gryp	1	m	0.507	34.05	1.056	0.135	hypoxia	2	0.87	1	Ucrit1hyp	75.0462
gryp	1	m	0.507	34.05	1.056	0.135	hypoxia	2	0.87	1	Ucrit2hyp	
gryp	1	m	0.507	34.05	1.056	0.135	hypoxia	2	0.87	1	Ucrit3hyp	

gryr	4	m	0.536	32.82	1.007	0.177	normoxia	1	0	0	Ucrit1norm	76.20804
gryr	4	m	0.536	32.82	1.007	0.177	normoxia	1	0	0	Ucrit2norm	84.74124
gryr	4	m	0.536	32.82	1.007	0.177	normoxia	1	0	0	Ucrit3norm	76.60188
gryr	4	m	0.536	32.82	1.007	0.177	normoxia	1	0	0	Ucrit1hyp	67.90458
gryr	4	m	0.536	32.82	1.007	0.177	normoxia	1	0	0	Ucrit2hyp	69.80814
gryr	4	m	0.536	32.82	1.007	0.177	normoxia	1	0	0	Ucrit3hyp	70.75992
gryr	4	m	0.536	32.82	1.007	0.177	hypoxia	1	0.626666667	1	Ucrit1norm	76.20804
gryr	4	m	0.536	32.82	1.007	0.177	hypoxia	1	0.626666667	1	Ucrit2norm	84.74124
gryr	4	m	0.536	32.82	1.007	0.177	hypoxia	1	0.626666667	1	Ucrit3norm	76.60188
gryr	4	m	0.536	32.82	1.007	0.177	hypoxia	1	0.626666667	1	Ucrit1hyp	67.90458
gryr	4	m	0.536	32.82	1.007	0.177	hypoxia	1	0.626666667	1	Ucrit2hyp	69.80814
gryr	4	m	0.536	32.82	1.007	0.177	hypoxia	1	0.626666667	1	Ucrit3hyp	70.75992
gryr	4	m	0.536	32.82	1.007	0.177	normoxia	2	0	0	Ucrit1norm	76.20804
gryr	4	m	0.536	32.82	1.007	0.177	normoxia	2	0	0	Ucrit2norm	84.74124
gryr	4	m	0.536	32.82	1.007	0.177	normoxia	2	0	0	Ucrit3norm	76.60188
gryr	4	m	0.536	32.82	1.007	0.177	normoxia	2	0	0	Ucrit1hyp	67.90458
gryr	4	m	0.536	32.82	1.007	0.177	normoxia	2	0	0	Ucrit2hyp	69.80814
gryr	4	m	0.536	32.82	1.007	0.177	normoxia	2	0	0	Ucrit3hyp	70.75992
gryr	4	m	0.536	32.82	1.007	0.177	hypoxia	2	0	0	Ucrit1norm	76.20804
gryr	4	m	0.536	32.82	1.007	0.177	hypoxia	2	0	0	Ucrit2norm	84.74124
gryr	4	m	0.536	32.82	1.007	0.177	hypoxia	2	0	0	Ucrit3norm	76.60188
gryr	4	m	0.536	32.82	1.007	0.177	hypoxia	2	0	0	Ucrit1hyp	67.90458
gryr	4	m	0.536	32.82	1.007	0.177	hypoxia	2	0	0	Ucrit2hyp	69.80814
gryr	4	m	0.536	32.82	1.007	0.177	hypoxia	2	0	0	Ucrit3hyp	70.75992
gygg	2	m	0.53	33.46	0.897	0.144	hypoxia	1	0	0	Ucrit1norm	85.72452
gygg	2	m	0.53	33.46	0.897	0.144	hypoxia	1	0	0	Ucrit2norm	
gygg	2	m	0.53	33.46	0.897	0.144	hypoxia	1	0	0	Ucrit3norm	
gygg	2	m	0.53	33.46	0.897	0.144	hypoxia	1	0	0	Ucrit1hyp	80.87282
gygg	2	m	0.53	33.46	0.897	0.144	hypoxia	1	0	0	Ucrit2hyp	
gygg	2	m	0.53	33.46	0.897	0.144	hypoxia	1	0	0	Ucrit3hyp	
gygg	2	m	0.53	33.46	0.897	0.144	normoxia	1	0	0	Ucrit1norm	85.72452
gygg	2	m	0.53	33.46	0.897	0.144	normoxia	1	0	0	Ucrit2norm	
gygg	2	m	0.53	33.46	0.897	0.144	normoxia	1	0	0	Ucrit3norm	

gygg	2	m	0.53	33.46	0.897	0.144	normoxia	1	0	0	Ucrit1hyp	80.87282
gygg	2	m	0.53	33.46	0.897	0.144	normoxia	1	0	0	Ucrit2hyp	
gygg	2	m	0.53	33.46	0.897	0.144	normoxia	1	0	0	Ucrit3hyp	
gygg	2	m	0.53	33.46	0.897	0.144	hypoxia	2	0	0	Ucrit1norm	85.72452
gygg	2	m	0.53	33.46	0.897	0.144	hypoxia	2	0	0	Ucrit2norm	
gygg	2	m	0.53	33.46	0.897	0.144	hypoxia	2	0	0	Ucrit3norm	
gygg	2	m	0.53	33.46	0.897	0.144	hypoxia	2	0	0	Ucrit1hyp	80.87282
gygg	2	m	0.53	33.46	0.897	0.144	hypoxia	2	0	0	Ucrit2hyp	
gygg	2	m	0.53	33.46	0.897	0.144	hypoxia	2	0	0	Ucrit3hyp	
gygg	2	m	0.53	33.46	0.897	0.144	normoxia	2	0	0	Ucrit1norm	85.72452
gygg	2	m	0.53	33.46	0.897	0.144	normoxia	2	0	0	Ucrit2norm	
gygg	2	m	0.53	33.46	0.897	0.144	normoxia	2	0	0	Ucrit3norm	
gygg	2	m	0.53	33.46	0.897	0.144	normoxia	2	0	0	Ucrit1hyp	80.87282
gygg	2	m	0.53	33.46	0.897	0.144	normoxia	2	0	0	Ucrit2hyp	
gygg	2	m	0.53	33.46	0.897	0.144	normoxia	2	0	0	Ucrit3hyp	
gygp	3	f	0.747	37.19	1.063	0.201	hypoxia	1	0	0	Ucrit1norm	69.28497
gygp	3	f	0.747	37.19	1.063	0.201	hypoxia	1	0	0	Ucrit2norm	
gygp	3	f	0.747	37.19	1.063	0.201	hypoxia	1	0	0	Ucrit3norm	
gygp	3	f	0.747	37.19	1.063	0.201	hypoxia	1	0	0	Ucrit1hyp	60.47094
gygp	3	f	0.747	37.19	1.063	0.201	hypoxia	1	0	0	Ucrit2hyp	
gygp	3	f	0.747	37.19	1.063	0.201	hypoxia	1	0	0	Ucrit3hyp	
gygp	3	f	0.747	37.19	1.063	0.201	normoxia	1	0	0	Ucrit1norm	69.28497
gygp	3	f	0.747	37.19	1.063	0.201	normoxia	1	0	0	Ucrit2norm	
gygp	3	f	0.747	37.19	1.063	0.201	normoxia	1	0	0	Ucrit3norm	
gygp	3	f	0.747	37.19	1.063	0.201	normoxia	1	0	0	Ucrit1hyp	60.47094
gygp	3	f	0.747	37.19	1.063	0.201	normoxia	1	0	0	Ucrit2hyp	
gygp	3	f	0.747	37.19	1.063	0.201	normoxia	1	0	0	Ucrit3hyp	
gygp	3	f	0.747	37.19	1.063	0.201	hypoxia	2	0	0	Ucrit1norm	69.28497
gygp	3	f	0.747	37.19	1.063	0.201	hypoxia	2	0	0	Ucrit2norm	
gygp	3	f	0.747	37.19	1.063	0.201	hypoxia	2	0	0	Ucrit3norm	
gygp	3	f	0.747	37.19	1.063	0.201	hypoxia	2	0	0	Ucrit1hyp	60.47094
gygp	3	f	0.747	37.19	1.063	0.201	hypoxia	2	0	0	Ucrit2hyp	
gygp	3	f	0.747	37.19	1.063	0.201	hypoxia	2	0	0	Ucrit3hyp	

gygp	3	f	0.747	37.19	1.063	0.201	normoxia	2	0	0	Ucrit1norm	69.28497
gygp	3	f	0.747	37.19	1.063	0.201	normoxia	2	0	0	Ucrit2norm	
gygp	3	f	0.747	37.19	1.063	0.201	normoxia	2	0	0	Ucrit3norm	
gygp	3	f	0.747	37.19	1.063	0.201	normoxia	2	0	0	Ucrit1hyp	60.47094
gygp	3	f	0.747	37.19	1.063	0.201	normoxia	2	0	0	Ucrit2hyp	
gygp	3	f	0.747	37.19	1.063	0.201	normoxia	2	0	0	Ucrit3hyp	
gygy	1	f	0.475	29.71	0.726	0.163	normoxia	1	0	0	Ucrit1norm	73.79964
gygy	1	f	0.475	29.71	0.726	0.163	normoxia	1	0	0	Ucrit2norm	
gygy	1	f	0.475	29.71	0.726	0.163	normoxia	1	0	0	Ucrit3norm	
gygy	1	f	0.475	29.71	0.726	0.163	normoxia	1	0	0	Ucrit1hyp	65.39171
gygy	1	f	0.475	29.71	0.726	0.163	normoxia	1	0	0	Ucrit2hyp	
gygy	1	f	0.475	29.71	0.726	0.163	normoxia	1	0	0	Ucrit3hyp	
gygy	1	f	0.475	29.71	0.726	0.163	hypoxia	1	0.745	1	Ucrit1norm	73.79964
gygy	1	f	0.475	29.71	0.726	0.163	hypoxia	1	0.745	1	Ucrit2norm	
gygy	1	f	0.475	29.71	0.726	0.163	hypoxia	1	0.745	1	Ucrit3norm	
gygy	1	f	0.475	29.71	0.726	0.163	hypoxia	1	0.745	1	Ucrit1hyp	65.39171
gygy	1	f	0.475	29.71	0.726	0.163	hypoxia	1	0.745	1	Ucrit2hyp	
gygy	1	f	0.475	29.71	0.726	0.163	hypoxia	1	0.745	1	Ucrit3hyp	
gygy	1	f	0.475	29.71	0.726	0.163	normoxia	2	0	0	Ucrit1norm	73.79964
gygy	1	f	0.475	29.71	0.726	0.163	normoxia	2	0	0	Ucrit2norm	
gygy	1	f	0.475	29.71	0.726	0.163	normoxia	2	0	0	Ucrit3norm	
gygy	1	f	0.475	29.71	0.726	0.163	normoxia	2	0	0	Ucrit1hyp	65.39171
gygy	1	f	0.475	29.71	0.726	0.163	normoxia	2	0	0	Ucrit2hyp	
gygy	1	f	0.475	29.71	0.726	0.163	normoxia	2	0	0	Ucrit3hyp	
gygy	1	f	0.475	29.71	0.726	0.163	hypoxia	2	0	0	Ucrit1norm	73.79964
gygy	1	f	0.475	29.71	0.726	0.163	hypoxia	2	0	0	Ucrit2norm	
gygy	1	f	0.475	29.71	0.726	0.163	hypoxia	2	0	0	Ucrit3norm	
gygy	1	f	0.475	29.71	0.726	0.163	hypoxia	2	0	0	Ucrit1hyp	65.39171
gygy	1	f	0.475	29.71	0.726	0.163	hypoxia	2	0	0	Ucrit2hyp	
gygy	1	f	0.475	29.71	0.726	0.163	hypoxia	2	0	0	Ucrit3hyp	
gypg	1	m	0.46	31.26	1.028	0.181	normoxia	1	0	0	Ucrit1norm	73.05462
gypg	1	m	0.46	31.26	1.028	0.181	normoxia	1	0	0	Ucrit2norm	
gypg	1	m	0.46	31.26	1.028	0.181	normoxia	1	0	0	Ucrit3norm	

gypg	1	m	0.46	31.26	1.028	0.181	normoxia	1	0	0	Ucrit1hyp	67.0527
gypg	1	m	0.46	31.26	1.028	0.181	normoxia	1	0	0	Ucrit2hyp	
gypg	1	m	0.46	31.26	1.028	0.181	normoxia	1	0	0	Ucrit3hyp	
gypg	1	m	0.46	31.26	1.028	0.181	hypoxia	1	0.448333333	1	Ucrit1norm	73.05462
gypg	1	m	0.46	31.26	1.028	0.181	hypoxia	1	0.448333333	1	Ucrit2norm	
gypg	1	m	0.46	31.26	1.028	0.181	hypoxia	1	0.448333333	1	Ucrit3norm	
gypg	1	m	0.46	31.26	1.028	0.181	hypoxia	1	0.448333333	1	Ucrit1hyp	67.0527
gypg	1	m	0.46	31.26	1.028	0.181	hypoxia	1	0.448333333	1	Ucrit2hyp	
gypg	1	m	0.46	31.26	1.028	0.181	hypoxia	1	0.448333333	1	Ucrit3hyp	
gypg	1	m	0.46	31.26	1.028	0.181	normoxia	2	0	0	Ucrit1norm	73.05462
gypg	1	m	0.46	31.26	1.028	0.181	normoxia	2	0	0	Ucrit2norm	
gypg	1	m	0.46	31.26	1.028	0.181	normoxia	2	0	0	Ucrit3norm	
gypg	1	m	0.46	31.26	1.028	0.181	normoxia	2	0	0	Ucrit1hyp	67.0527
gypg	1	m	0.46	31.26	1.028	0.181	normoxia	2	0	0	Ucrit2hyp	
gypg	1	m	0.46	31.26	1.028	0.181	normoxia	2	0	0	Ucrit3hyp	
gypg	1	m	0.46	31.26	1.028	0.181	hypoxia	2	0	0	Ucrit1norm	73.05462
gypg	1	m	0.46	31.26	1.028	0.181	hypoxia	2	0	0	Ucrit2norm	
gypg	1	m	0.46	31.26	1.028	0.181	hypoxia	2	0	0	Ucrit3norm	
gypg	1	m	0.46	31.26	1.028	0.181	hypoxia	2	0	0	Ucrit1hyp	67.0527
gypg	1	m	0.46	31.26	1.028	0.181	hypoxia	2	0	0	Ucrit2hyp	
gypg	1	m	0.46	31.26	1.028	0.181	hypoxia	2	0	0	Ucrit3hyp	
gypp	1	f	0.503	34.29	1.118	0.177	normoxia	1	0	0	Ucrit1norm	69.54012
gypp	1	f	0.503	34.29	1.118	0.177	normoxia	1	0	0	Ucrit2norm	
gypp	1	f	0.503	34.29	1.118	0.177	normoxia	1	0	0	Ucrit3norm	
gypp	1	f	0.503	34.29	1.118	0.177	normoxia	1	0	0	Ucrit1hyp	79.07274
gypp	1	f	0.503	34.29	1.118	0.177	normoxia	1	0	0	Ucrit2hyp	
gypp	1	f	0.503	34.29	1.118	0.177	normoxia	1	0	0	Ucrit3hyp	
gypp	1	f	0.503	34.29	1.118	0.177	hypoxia	1	0	0	Ucrit1norm	69.54012
gypp	1	f	0.503	34.29	1.118	0.177	hypoxia	1	0	0	Ucrit2norm	
gypp	1	f	0.503	34.29	1.118	0.177	hypoxia	1	0	0	Ucrit3norm	
gypp	1	f	0.503	34.29	1.118	0.177	hypoxia	1	0	0	Ucrit1hyp	79.07274
gypp	1	f	0.503	34.29	1.118	0.177	hypoxia	1	0	0	Ucrit2hyp	
gypp	1	f	0.503	34.29	1.118	0.177	hypoxia	1	0	0	Ucrit3hyp	

gypp	1	f	0.503	34.29	1.118	0.177	normoxia	2	0	0	Ucrit1norm	69.54012
gypp	1	f	0.503	34.29	1.118	0.177	normoxia	2	0	0	Ucrit2norm	
gypp	1	f	0.503	34.29	1.118	0.177	normoxia	2	0	0	Ucrit3norm	
gypp	1	f	0.503	34.29	1.118	0.177	normoxia	2	0	0	Ucrit1hyp	79.07274
gypp	1	f	0.503	34.29	1.118	0.177	normoxia	2	0	0	Ucrit2hyp	
gypp	1	f	0.503	34.29	1.118	0.177	normoxia	2	0	0	Ucrit3hyp	
gypp	1	f	0.503	34.29	1.118	0.177	hypoxia	2	0	0	Ucrit1norm	69.54012
gypp	1	f	0.503	34.29	1.118	0.177	hypoxia	2	0	0	Ucrit2norm	
gypp	1	f	0.503	34.29	1.118	0.177	hypoxia	2	0	0	Ucrit3norm	
gypp	1	f	0.503	34.29	1.118	0.177	hypoxia	2	0	0	Ucrit1hyp	79.07274
gypp	1	f	0.503	34.29	1.118	0.177	hypoxia	2	0	0	Ucrit2hyp	
gypp	1	f	0.503	34.29	1.118	0.177	hypoxia	2	0	0	Ucrit3hyp	
gypr	2	f	0.471	29.63	1.05	0.233	hypoxia	1	0	0	Ucrit1norm	76.20836
gypr	2	f	0.471	29.63	1.05	0.233	hypoxia	1	0	0	Ucrit2norm	
gypr	2	f	0.471	29.63	1.05	0.233	hypoxia	1	0	0	Ucrit3norm	
gypr	2	f	0.471	29.63	1.05	0.233	hypoxia	1	0	0	Ucrit1hyp	59.58593
gypr	2	f	0.471	29.63	1.05	0.233	hypoxia	1	0	0	Ucrit2hyp	
gypr	2	f	0.471	29.63	1.05	0.233	hypoxia	1	0	0	Ucrit3hyp	
gypr	2	f	0.471	29.63	1.05	0.233	normoxia	1	0	0	Ucrit1norm	76.20836
gypr	2	f	0.471	29.63	1.05	0.233	normoxia	1	0	0	Ucrit2norm	
gypr	2	f	0.471	29.63	1.05	0.233	normoxia	1	0	0	Ucrit3norm	
gypr	2	f	0.471	29.63	1.05	0.233	normoxia	1	0	0	Ucrit1hyp	59.58593
gypr	2	f	0.471	29.63	1.05	0.233	normoxia	1	0	0	Ucrit2hyp	
gypr	2	f	0.471	29.63	1.05	0.233	normoxia	1	0	0	Ucrit3hyp	
gypr	2	f	0.471	29.63	1.05	0.233	hypoxia	2	0	0	Ucrit1norm	76.20836
gypr	2	f	0.471	29.63	1.05	0.233	hypoxia	2	0	0	Ucrit2norm	
gypr	2	f	0.471	29.63	1.05	0.233	hypoxia	2	0	0	Ucrit3norm	
gypr	2	f	0.471	29.63	1.05	0.233	hypoxia	2	0	0	Ucrit1hyp	59.58593
gypr	2	f	0.471	29.63	1.05	0.233	hypoxia	2	0	0	Ucrit2hyp	
gypr	2	f	0.471	29.63	1.05	0.233	hypoxia	2	0	0	Ucrit3hyp	
gypr	2	f	0.471	29.63	1.05	0.233	normoxia	2	0	0	Ucrit1norm	76.20836
gypr	2	f	0.471	29.63	1.05	0.233	normoxia	2	0	0	Ucrit2norm	
gypr	2	f	0.471	29.63	1.05	0.233	normoxia	2	0	0	Ucrit3norm	

gypr	2	f	0.471	29.63	1.05	0.233	normoxia	2	0	0	Ucrit1hyp	59.58593
gypr	2	f	0.471	29.63	1.05	0.233	normoxia	2	0	0	Ucrit2hyp	
gypr	2	f	0.471	29.63	1.05	0.233	normoxia	2	0	0	Ucrit3hyp	
gyrg	4	f	0.518	31.54			normoxia	1	0	0	Ucrit1norm	84.77952
gyrg	4	f	0.518	31.54			normoxia	1	0	0	Ucrit2norm	81.75168
gyrg	4	f	0.518	31.54			normoxia	1	0	0	Ucrit3norm	82.7925
gyrg	4	f	0.518	31.54			normoxia	1	0	0	Ucrit1hyp	69.04106
gyrg	4	f	0.518	31.54			normoxia	1	0	0	Ucrit2hyp	63.14308
gyrg	4	f	0.518	31.54			normoxia	1	0	0	Ucrit3hyp	68.56796
gyrg	4	f	0.518	31.54			hypoxia	1	0	0	Ucrit1norm	84.77952
gyrg	4	f	0.518	31.54			hypoxia	1	0	0	Ucrit2norm	81.75168
gyrg	4	f	0.518	31.54			hypoxia	1	0	0	Ucrit3norm	82.7925
gyrg	4	f	0.518	31.54			hypoxia	1	0	0	Ucrit1hyp	69.04106
gyrg	4	f	0.518	31.54			hypoxia	1	0	0	Ucrit2hyp	63.14308
gyrg	4	f	0.518	31.54			hypoxia	1	0	0	Ucrit3hyp	68.56796
gyrg	4	f	0.518	31.54			normoxia	2	0	0	Ucrit1norm	84.77952
gyrg	4	f	0.518	31.54			normoxia	2	0	0	Ucrit2norm	81.75168
gyrg	4	f	0.518	31.54			normoxia	2	0	0	Ucrit3norm	82.7925
gyrg	4	f	0.518	31.54			normoxia	2	0	0	Ucrit1hyp	69.04106
gyrg	4	f	0.518	31.54			normoxia	2	0	0	Ucrit2hyp	63.14308
gyrg	4	f	0.518	31.54			normoxia	2	0	0	Ucrit3hyp	68.56796
gyrg	4	f	0.518	31.54			hypoxia	2	0	0	Ucrit1norm	84.77952
gyrg	4	f	0.518	31.54			hypoxia	2	0	0	Ucrit2norm	81.75168
gyrg	4	f	0.518	31.54			hypoxia	2	0	0	Ucrit3norm	82.7925
gyrg	4	f	0.518	31.54			hypoxia	2	0	0	Ucrit1hyp	69.04106
gyrg	4	f	0.518	31.54			hypoxia	2	0	0	Ucrit2hyp	63.14308
gyrg	4	f	0.518	31.54			hypoxia	2	0	0	Ucrit3hyp	68.56796
gyrp	1	m	0.476	32.47	1.354	0.133	normoxia	1	0	0	Ucrit1norm	89.26003
gyrp	1	m	0.476	32.47	1.354	0.133	normoxia	1	0	0	Ucrit2norm	
gyrp	1	m	0.476	32.47	1.354	0.133	normoxia	1	0	0	Ucrit3norm	
gyrp	1	m	0.476	32.47	1.354	0.133	normoxia	1	0	0	Ucrit1hyp	72.86268
gyrp	1	m	0.476	32.47	1.354	0.133	normoxia	1	0	0	Ucrit2hyp	
gyrp	1	m	0.476	32.47	1.354	0.133	normoxia	1	0	0	Ucrit3hyp	

gyrp	1	m	0.476	32.47	1.354	0.133	hypoxia	1	0	0	Ucrit1norm	89.26003
gyrp	1	m	0.476	32.47	1.354	0.133	hypoxia	1	0	0	Ucrit2norm	
gyrp	1	m	0.476	32.47	1.354	0.133	hypoxia	1	0	0	Ucrit3norm	
gyrp	1	m	0.476	32.47	1.354	0.133	hypoxia	1	0	0	Ucrit1hyp	72.86268
gyrp	1	m	0.476	32.47	1.354	0.133	hypoxia	1	0	0	Ucrit2hyp	
gyrp	1	m	0.476	32.47	1.354	0.133	hypoxia	1	0	0	Ucrit3hyp	
gyrp	1	m	0.476	32.47	1.354	0.133	normoxia	2	0	0	Ucrit1norm	89.26003
gyrp	1	m	0.476	32.47	1.354	0.133	normoxia	2	0	0	Ucrit2norm	
gyrp	1	m	0.476	32.47	1.354	0.133	normoxia	2	0	0	Ucrit3norm	
gyrp	1	m	0.476	32.47	1.354	0.133	normoxia	2	0	0	Ucrit1hyp	72.86268
gyrp	1	m	0.476	32.47	1.354	0.133	normoxia	2	0	0	Ucrit2hyp	
gyrp	1	m	0.476	32.47	1.354	0.133	normoxia	2	0	0	Ucrit3hyp	
gyrp	1	m	0.476	32.47	1.354	0.133	hypoxia	2	0	0	Ucrit1norm	89.26003
gyrp	1	m	0.476	32.47	1.354	0.133	hypoxia	2	0	0	Ucrit2norm	
gyrp	1	m	0.476	32.47	1.354	0.133	hypoxia	2	0	0	Ucrit3norm	
gyrp	1	m	0.476	32.47	1.354	0.133	hypoxia	2	0	0	Ucrit1hyp	72.86268
gyrp	1	m	0.476	32.47	1.354	0.133	hypoxia	2	0	0	Ucrit2hyp	
gyrp	1	m	0.476	32.47	1.354	0.133	hypoxia	2	0	0	Ucrit3hyp	
gyrr	4	f	0.595	33.42			normoxia	1	0	0	Ucrit1norm	83.0487
gyrr	4	f	0.595	33.42			normoxia	1	0	0	Ucrit2norm	84.68628
gyrr	4	f	0.595	33.42			normoxia	1	0	0	Ucrit3norm	85.65546
gyrr	4	f	0.595	33.42			normoxia	1	0	0	Ucrit1hyp	65.3361
gyrr	4	f	0.595	33.42			normoxia	1	0	0	Ucrit2hyp	64.26666
gyrr	4	f	0.595	33.42			normoxia	1	0	0	Ucrit3hyp	74.12556
gyrr	4	f	0.595	33.42			hypoxia	1	0	0	Ucrit1norm	83.0487
gyrr	4	f	0.595	33.42			hypoxia	1	0	0	Ucrit2norm	84.68628
gyrr	4	f	0.595	33.42			hypoxia	1	0	0	Ucrit3norm	85.65546
gyrr	4	f	0.595	33.42			hypoxia	1	0	0	Ucrit1hyp	65.3361
gyrr	4	f	0.595	33.42			hypoxia	1	0	0	Ucrit2hyp	64.26666
gyrr	4	f	0.595	33.42			hypoxia	1	0	0	Ucrit3hyp	74.12556
gyrr	4	f	0.595	33.42			normoxia	2	0	0	Ucrit1norm	83.0487
gyrr	4	f	0.595	33.42			normoxia	2	0	0	Ucrit2norm	84.68628
gyrr	4	f	0.595	33.42			normoxia	2	0	0	Ucrit3norm	85.65546

gyrr	4	f	0.595	33.42	normoxia	2	0	0	Ucrit1hyp	65.3361
gyrr	4	f	0.595	33.42	normoxia	2	0	0	Ucrit2hyp	64.26666
gyrr	4	f	0.595	33.42	normoxia	2	0	0	Ucrit3hyp	74.12556
gyrr	4	f	0.595	33.42	hypoxia	2	0	0	Ucrit1norm	83.0487
gyrr	4	f	0.595	33.42	hypoxia	2	0	0	Ucrit2norm	84.68628
gyrr	4	f	0.595	33.42	hypoxia	2	0	0	Ucrit3norm	85.65546
gyrr	4	f	0.595	33.42	hypoxia	2	0	0	Ucrit1hyp	65.3361
gyrr	4	f	0.595	33.42	hypoxia	2	0	0	Ucrit2hyp	64.26666
gyrr	4	f	0.595	33.42	hypoxia	2	0	0	Ucrit3hyp	74.12556
gyry	3	m	0.57	34.37	hypoxia	1	0	0	Ucrit1norm	84.8939
gyry	3	m	0.57	34.37	hypoxia	1	0	0	Ucrit2norm	
gyry	3	m	0.57	34.37	hypoxia	1	0	0	Ucrit3norm	
gyry	3	m	0.57	34.37	hypoxia	1	0	0	Ucrit1hyp	
gyry	3	m	0.57	34.37	hypoxia	1	0	0	Ucrit2hyp	
gyry	3	m	0.57	34.37	hypoxia	1	0	0	Ucrit3hyp	
gyry	3	m	0.57	34.37	normoxia	1	0	0	Ucrit1norm	84.8939
gyry	3	m	0.57	34.37	normoxia	1	0	0	Ucrit2norm	
gyry	3	m	0.57	34.37	normoxia	1	0	0	Ucrit3norm	
gyry	3	m	0.57	34.37	normoxia	1	0	0	Ucrit1hyp	
gyry	3	m	0.57	34.37	normoxia	1	0	0	Ucrit2hyp	
gyry	3	m	0.57	34.37	normoxia	1	0	0	Ucrit3hyp	
gyry	3	m	0.57	34.37	hypoxia	2	0.03	1	Ucrit1norm	84.8939
gyry	3	m	0.57	34.37	hypoxia	2	0.03	1	Ucrit2norm	
gyry	3	m	0.57	34.37	hypoxia	2	0.03	1	Ucrit3norm	
gyry	3	m	0.57	34.37	hypoxia	2	0.03	1	Ucrit1hyp	
gyry	3	m	0.57	34.37	hypoxia	2	0.03	1	Ucrit2hyp	
gyry	3	m	0.57	34.37	hypoxia	2	0.03	1	Ucrit3hyp	
gyry	3	m	0.57	34.37	normoxia	2	0	0	Ucrit1norm	84.8939
gyry	3	m	0.57	34.37	normoxia	2	0	0	Ucrit2norm	
gyry	3	m	0.57	34.37	normoxia	2	0	0	Ucrit3norm	
gyry	3	m	0.57	34.37	normoxia	2	0	0	Ucrit1hyp	
gyry	3	m	0.57	34.37	normoxia	2	0	0	Ucrit2hyp	
gyry	3	m	0.57	34.37	normoxia	2	0	0	Ucrit3hyp	

gyyg	2				1.023	0.173	hypoxia	1	0.631666667	1		
gyyg	2				1.023	0.173	normoxia	1	0	0		
gyyg	2				1.023	0.173	hypoxia	2	0	0		
gyyg	2				1.023	0.173	normoxia	2	0	0		
gyyp	1	m	0.59	35.6	1.149	0.178	normoxia	1	0	0	Ucrit1norm	84.5856
gyyp	1	m	0.59	35.6	1.149	0.178	normoxia	1	0	0	Ucrit2norm	
gyyp	1	m	0.59	35.6	1.149	0.178	normoxia	1	0	0	Ucrit3norm	
gyyp	1	m	0.59	35.6	1.149	0.178	normoxia	1	0	0	Ucrit1hyp	68.4588
gyyp	1	m	0.59	35.6	1.149	0.178	normoxia	1	0	0	Ucrit2hyp	
gyyp	1	m	0.59	35.6	1.149	0.178	normoxia	1	0	0	Ucrit3hyp	
gyyp	1	m	0.59	35.6	1.149	0.178	hypoxia	1	0.705	1	Ucrit1norm	84.5856
gyyp	1	m	0.59	35.6	1.149	0.178	hypoxia	1	0.705	1	Ucrit2norm	
gyyp	1	m	0.59	35.6	1.149	0.178	hypoxia	1	0.705	1	Ucrit3norm	
gyyp	1	m	0.59	35.6	1.149	0.178	hypoxia	1	0.705	1	Ucrit1hyp	68.4588
gyyp	1	m	0.59	35.6	1.149	0.178	hypoxia	1	0.705	1	Ucrit2hyp	
gyyp	1	m	0.59	35.6	1.149	0.178	hypoxia	1	0.705	1	Ucrit3hyp	
gyyp	1	m	0.59	35.6	1.149	0.178	normoxia	2	0	0	Ucrit1norm	84.5856
gyyp	1	m	0.59	35.6	1.149	0.178	normoxia	2	0	0	Ucrit2norm	
gyyp	1	m	0.59	35.6	1.149	0.178	normoxia	2	0	0	Ucrit3norm	
gyyp	1	m	0.59	35.6	1.149	0.178	normoxia	2	0	0	Ucrit1hyp	68.4588
gyyp	1	m	0.59	35.6	1.149	0.178	normoxia	2	0	0	Ucrit2hyp	
gyyp	1	m	0.59	35.6	1.149	0.178	normoxia	2	0	0	Ucrit3hyp	
gyyp	1	m	0.59	35.6	1.149	0.178	hypoxia	2	0.888333333	1	Ucrit1norm	84.5856
gyyp	1	m	0.59	35.6	1.149	0.178	hypoxia	2	0.888333333	1	Ucrit2norm	
gyyp	1	m	0.59	35.6	1.149	0.178	hypoxia	2	0.888333333	1	Ucrit3norm	
gyyp	1	m	0.59	35.6	1.149	0.178	hypoxia	2	0.888333333	1	Ucrit1hyp	68.4588
gyyp	1	m	0.59	35.6	1.149	0.178	hypoxia	2	0.888333333	1	Ucrit2hyp	
gyyp	1	m	0.59	35.6	1.149	0.178	hypoxia	2	0.888333333	1	Ucrit3hyp	
gyyr	2	f	0.659	34.84	0.973	0.185	hypoxia	1	0	0	Ucrit1norm	74.06984
gyyr	2	f	0.659	34.84	0.973	0.185	hypoxia	1	0	0	Ucrit2norm	
gyyr	2	f	0.659	34.84	0.973	0.185	hypoxia	1	0	0	Ucrit3norm	
gyyr	2	f	0.659	34.84	0.973	0.185	hypoxia	1	0	0	Ucrit1hyp	56.64984
gyyr	2	f	0.659	34.84	0.973	0.185	hypoxia	1	0	0	Ucrit2hyp	

gyyr	2	f	0.659	34.84	0.973	0.185	hypoxia	1	0	0	Ucrit3hyp	
gyyr	2	f	0.659	34.84	0.973	0.185	normoxia	1	0	0	Ucrit1norm	74.06984
gyyr	2	f	0.659	34.84	0.973	0.185	normoxia	1	0	0	Ucrit2norm	
gyyr	2	f	0.659	34.84	0.973	0.185	normoxia	1	0	0	Ucrit3norm	
gyyr	2	f	0.659	34.84	0.973	0.185	normoxia	1	0	0	Ucrit1hyp	56.64984
gyyr	2	f	0.659	34.84	0.973	0.185	normoxia	1	0	0	Ucrit2hyp	
gyyr	2	f	0.659	34.84	0.973	0.185	normoxia	1	0	0	Ucrit3hyp	
gyyr	2	f	0.659	34.84	0.973	0.185	hypoxia	2	0.741666667	1	Ucrit1norm	74.06984
gyyr	2	f	0.659	34.84	0.973	0.185	hypoxia	2	0.741666667	1	Ucrit2norm	
gyyr	2	f	0.659	34.84	0.973	0.185	hypoxia	2	0.741666667	1	Ucrit3norm	
gyyr	2	f	0.659	34.84	0.973	0.185	hypoxia	2	0.741666667	1	Ucrit1hyp	56.64984
gyyr	2	f	0.659	34.84	0.973	0.185	hypoxia	2	0.741666667	1	Ucrit2hyp	
gyyr	2	f	0.659	34.84	0.973	0.185	hypoxia	2	0.741666667	1	Ucrit3hyp	
gyyr	2	f	0.659	34.84	0.973	0.185	normoxia	2	0	0	Ucrit1norm	74.06984
gyyr	2	f	0.659	34.84	0.973	0.185	normoxia	2	0	0	Ucrit2norm	
gyyr	2	f	0.659	34.84	0.973	0.185	normoxia	2	0	0	Ucrit3norm	
gyyr	2	f	0.659	34.84	0.973	0.185	normoxia	2	0	0	Ucrit1hyp	56.64984
gyyr	2	f	0.659	34.84	0.973	0.185	normoxia	2	0	0	Ucrit2hyp	
gyyr	2	f	0.659	34.84	0.973	0.185	normoxia	2	0	0	Ucrit3hyp	
pggg	4	f	0.393	28.09			normoxia	1	0	0	Ucrit1norm	84.77562
pggg	4	f	0.393	28.09			normoxia	1	0	0	Ucrit2norm	79.46661
pggg	4	f	0.393	28.09			normoxia	1	0	0	Ucrit3norm	87.6408
pggg	4	f	0.393	28.09			normoxia	1	0	0	Ucrit1hyp	71.96658
pggg	4	f	0.393	28.09			normoxia	1	0	0	Ucrit2hyp	85.61832
pggg	4	f	0.393	28.09			normoxia	1	0	0	Ucrit3hyp	89.3262
pggg	4	f	0.393	28.09			hypoxia	1	0.666666667	1	Ucrit1norm	84.77562
pggg	4	f	0.393	28.09			hypoxia	1	0.666666667	1	Ucrit2norm	79.46661
pggg	4	f	0.393	28.09			hypoxia	1	0.666666667	1	Ucrit3norm	87.6408
pggg	4	f	0.393	28.09			hypoxia	1	0.666666667	1	Ucrit1hyp	71.96658
pggg	4	f	0.393	28.09			hypoxia	1	0.666666667	1	Ucrit2hyp	85.61832
pggg	4	f	0.393	28.09			hypoxia	1	0.666666667	1	Ucrit3hyp	89.3262
pggg	4	f	0.393	28.09			normoxia	2	0	0	Ucrit1norm	84.77562
pggg	4	f	0.393	28.09			normoxia	2	0	0	Ucrit2norm	79.46661

pggg	4	f	0.393	28.09			normoxia	2	0	0	Ucrit3norm	87.6408
pggg	4	f	0.393	28.09			normoxia	2	0	0	Ucrit1hyp	71.96658
pggg	4	f	0.393	28.09			normoxia	2	0	0	Ucrit2hyp	85.61832
pggg	4	f	0.393	28.09			normoxia	2	0	0	Ucrit3hyp	89.3262
pggg	4	f	0.393	28.09			hypoxia	2	0.895	1	Ucrit1norm	84.77562
pggg	4	f	0.393	28.09			hypoxia	2	0.895	1	Ucrit2norm	79.46661
pggg	4	f	0.393	28.09			hypoxia	2	0.895	1	Ucrit3norm	87.6408
pggg	4	f	0.393	28.09			hypoxia	2	0.895	1	Ucrit1hyp	71.96658
pggg	4	f	0.393	28.09			hypoxia	2	0.895	1	Ucrit2hyp	85.61832
pggg	4	f	0.393	28.09			hypoxia	2	0.895	1	Ucrit3hyp	89.3262
pggp	3	m	0.397	30.29	1.442	0.197	hypoxia	1	0.611666667	1	Ucrit1norm	78.57226
pggp	3	m	0.397	30.29	1.442	0.197	hypoxia	1	0.611666667	1	Ucrit2norm	
pggp	3	m	0.397	30.29	1.442	0.197	hypoxia	1	0.611666667	1	Ucrit3norm	
pggp	3	m	0.397	30.29	1.442	0.197	hypoxia	1	0.611666667	1	Ucrit1hyp	62.88204
pggp	3	m	0.397	30.29	1.442	0.197	hypoxia	1	0.611666667	1	Ucrit2hyp	
pggp	3	m	0.397	30.29	1.442	0.197	hypoxia	1	0.611666667	1	Ucrit3hyp	
pggp	3	m	0.397	30.29	1.442	0.197	normoxia	1	0	0	Ucrit1norm	78.57226
pggp	3	m	0.397	30.29	1.442	0.197	normoxia	1	0	0	Ucrit2norm	
pggp	3	m	0.397	30.29	1.442	0.197	normoxia	1	0	0	Ucrit3norm	
pggp	3	m	0.397	30.29	1.442	0.197	normoxia	1	0	0	Ucrit1hyp	62.88204
pggp	3	m	0.397	30.29	1.442	0.197	normoxia	1	0	0	Ucrit2hyp	
pggp	3	m	0.397	30.29	1.442	0.197	normoxia	1	0	0	Ucrit3hyp	
pggp	3	m	0.397	30.29	1.442	0.197	hypoxia	2	0	0	Ucrit1norm	78.57226
pggp	3	m	0.397	30.29	1.442	0.197	hypoxia	2	0	0	Ucrit2norm	
pggp	3	m	0.397	30.29	1.442	0.197	hypoxia	2	0	0	Ucrit3norm	
pggp	3	m	0.397	30.29	1.442	0.197	hypoxia	2	0	0	Ucrit1hyp	62.88204
pggp	3	m	0.397	30.29	1.442	0.197	hypoxia	2	0	0	Ucrit2hyp	
pggp	3	m	0.397	30.29	1.442	0.197	hypoxia	2	0	0	Ucrit3hyp	
pggp	3	m	0.397	30.29	1.442	0.197	normoxia	2	0	0	Ucrit1norm	78.57226
pggp	3	m	0.397	30.29	1.442	0.197	normoxia	2	0	0	Ucrit2norm	
pggp	3	m	0.397	30.29	1.442	0.197	normoxia	2	0	0	Ucrit3norm	
pggp	3	m	0.397	30.29	1.442	0.197	normoxia	2	0	0	Ucrit1hyp	62.88204
pggp	3	m	0.397	30.29	1.442	0.197	normoxia	2	0	0	Ucrit2hyp	

pggp	3	m	0.397	30.29	1.442	0.197	normoxia	2	0	0	Ucrit3hyp	
pggr	4				0.904	0.179	normoxia	1	0	0		
pggr	4				0.904	0.179	hypoxia	1	0	0		
pggr	4				0.904	0.179	normoxia	2	0	0		
pggr	4				0.904	0.179	hypoxia	2	0	0		
pggy	4	f	0.648	33.71	1.035	0.189	normoxia	1	0	0	Ucrit1norm	76.21831
pggy	4	f	0.648	33.71	1.035	0.189	normoxia	1	0	0	Ucrit2norm	78.64543
pggy	4	f	0.648	33.71	1.035	0.189	normoxia	1	0	0	Ucrit3norm	81.88159
pggy	4	f	0.648	33.71	1.035	0.189	normoxia	1	0	0	Ucrit1hyp	64.01529
pggy	4	f	0.648	33.71	1.035	0.189	normoxia	1	0	0	Ucrit2hyp	71.36407
pggy	4	f	0.648	33.71	1.035	0.189	normoxia	1	0	0	Ucrit3hyp	84.20758
pggy	4	f	0.648	33.71	1.035	0.189	hypoxia	1	0	0	Ucrit1norm	76.21831
pggy	4	f	0.648	33.71	1.035	0.189	hypoxia	1	0	0	Ucrit2norm	78.64543
pggy	4	f	0.648	33.71	1.035	0.189	hypoxia	1	0	0	Ucrit3norm	81.88159
pggy	4	f	0.648	33.71	1.035	0.189	hypoxia	1	0	0	Ucrit1hyp	64.01529
pggy	4	f	0.648	33.71	1.035	0.189	hypoxia	1	0	0	Ucrit2hyp	71.36407
pggy	4	f	0.648	33.71	1.035	0.189	hypoxia	1	0	0	Ucrit3hyp	84.20758
pggy	4	f	0.648	33.71	1.035	0.189	normoxia	2	0	0	Ucrit1norm	76.21831
pggy	4	f	0.648	33.71	1.035	0.189	normoxia	2	0	0	Ucrit2norm	78.64543
pggy	4	f	0.648	33.71	1.035	0.189	normoxia	2	0	0	Ucrit3norm	81.88159
pggy	4	f	0.648	33.71	1.035	0.189	normoxia	2	0	0	Ucrit1hyp	64.01529
pggy	4	f	0.648	33.71	1.035	0.189	normoxia	2	0	0	Ucrit2hyp	71.36407
pggy	4	f	0.648	33.71	1.035	0.189	normoxia	2	0	0	Ucrit3hyp	84.20758
pggy	4	f	0.648	33.71	1.035	0.189	hypoxia	2	0	0	Ucrit1norm	76.21831
pggy	4	f	0.648	33.71	1.035	0.189	hypoxia	2	0	0	Ucrit2norm	78.64543
pggy	4	f	0.648	33.71	1.035	0.189	hypoxia	2	0	0	Ucrit3norm	81.88159
pggy	4	f	0.648	33.71	1.035	0.189	hypoxia	2	0	0	Ucrit1hyp	64.01529
pggy	4	f	0.648	33.71	1.035	0.189	hypoxia	2	0	0	Ucrit2hyp	71.36407
pggy	4	f	0.648	33.71	1.035	0.189	hypoxia	2	0	0	Ucrit3hyp	84.20758
pgpp	2	m	0.546	34.27	1.235	0.133	hypoxia	1	0	0	Ucrit1norm	88.10817
pgpp	2	m	0.546	34.27	1.235	0.133	hypoxia	1	0	0	Ucrit2norm	
pgpp	2	m	0.546	34.27	1.235	0.133	hypoxia	1	0	0	Ucrit3norm	
pgpp	2	m	0.546	34.27	1.235	0.133	hypoxia	1	0	0	Ucrit1hyp	88.51941

pgpp	2	m	0.546	34.27	1.235	0.133	hypoxia	1	0	0	Ucrit2hyp	
pgpp	2	m	0.546	34.27	1.235	0.133	hypoxia	1	0	0	Ucrit3hyp	
pgpp	2	m	0.546	34.27	1.235	0.133	normoxia	1	0	0	Ucrit1norm	88.10817
pgpp	2	m	0.546	34.27	1.235	0.133	normoxia	1	0	0	Ucrit2norm	
pgpp	2	m	0.546	34.27	1.235	0.133	normoxia	1	0	0	Ucrit3norm	
pgpp	2	m	0.546	34.27	1.235	0.133	normoxia	1	0	0	Ucrit1hyp	88.51941
pgpp	2	m	0.546	34.27	1.235	0.133	normoxia	1	0	0	Ucrit2hyp	
pgpp	2	m	0.546	34.27	1.235	0.133	normoxia	1	0	0	Ucrit3hyp	
pgpp	2	m	0.546	34.27	1.235	0.133	hypoxia	2	0	0	Ucrit1norm	88.10817
pgpp	2	m	0.546	34.27	1.235	0.133	hypoxia	2	0	0	Ucrit2norm	
pgpp	2	m	0.546	34.27	1.235	0.133	hypoxia	2	0	0	Ucrit3norm	
pgpp	2	m	0.546	34.27	1.235	0.133	hypoxia	2	0	0	Ucrit1hyp	88.51941
pgpp	2	m	0.546	34.27	1.235	0.133	hypoxia	2	0	0	Ucrit2hyp	
pgpp	2	m	0.546	34.27	1.235	0.133	hypoxia	2	0	0	Ucrit3hyp	
pgpp	2	m	0.546	34.27	1.235	0.133	normoxia	2	0	0	Ucrit1norm	88.10817
pgpp	2	m	0.546	34.27	1.235	0.133	normoxia	2	0	0	Ucrit2norm	
pgpp	2	m	0.546	34.27	1.235	0.133	normoxia	2	0	0	Ucrit3norm	
pgpp	2	m	0.546	34.27	1.235	0.133	normoxia	2	0	0	Ucrit1hyp	88.51941
pgpp	2	m	0.546	34.27	1.235	0.133	normoxia	2	0	0	Ucrit2hyp	
pgpp	2	m	0.546	34.27	1.235	0.133	normoxia	2	0	0	Ucrit3hyp	
pgrp	1	f	0.698	33.48	1.041	0.196	normoxia	1	0	0	Ucrit1norm	73.38816
pgrp	1	f	0.698	33.48	1.041	0.196	normoxia	1	0	0	Ucrit2norm	
pgrp	1	f	0.698	33.48	1.041	0.196	normoxia	1	0	0	Ucrit3norm	
pgrp	1	f	0.698	33.48	1.041	0.196	normoxia	1	0	0	Ucrit1hyp	69.6384
pgrp	1	f	0.698	33.48	1.041	0.196	normoxia	1	0	0	Ucrit2hyp	
pgrp	1	f	0.698	33.48	1.041	0.196	normoxia	1	0	0	Ucrit3hyp	
pgrp	1	f	0.698	33.48	1.041	0.196	hypoxia	1	0	0	Ucrit1norm	73.38816
pgrp	1	f	0.698	33.48	1.041	0.196	hypoxia	1	0	0	Ucrit2norm	
pgrp	1	f	0.698	33.48	1.041	0.196	hypoxia	1	0	0	Ucrit3norm	
pgrp	1	f	0.698	33.48	1.041	0.196	hypoxia	1	0	0	Ucrit1hyp	69.6384
pgrp	1	f	0.698	33.48	1.041	0.196	hypoxia	1	0	0	Ucrit2hyp	
pgrp	1	f	0.698	33.48	1.041	0.196	hypoxia	1	0	0	Ucrit3hyp	
pgrp	1	f	0.698	33.48	1.041	0.196	normoxia	2	0	0	Ucrit1norm	73.38816

pgrp	1	f	0.698	33.48	1.041	0.196	normoxia	2	0	0	Ucrit2norm	
pgrp	1	f	0.698	33.48	1.041	0.196	normoxia	2	0	0	Ucrit3norm	
pgrp	1	f	0.698	33.48	1.041	0.196	normoxia	2	0	0	Ucrit1hyp	69.6384
pgrp	1	f	0.698	33.48	1.041	0.196	normoxia	2	0	0	Ucrit2hyp	
pgrp	1	f	0.698	33.48	1.041	0.196	normoxia	2	0	0	Ucrit3hyp	
pgrp	1	f	0.698	33.48	1.041	0.196	hypoxia	2	0	0	Ucrit1norm	73.38816
pgrp	1	f	0.698	33.48	1.041	0.196	hypoxia	2	0	0	Ucrit2norm	
pgrp	1	f	0.698	33.48	1.041	0.196	hypoxia	2	0	0	Ucrit3norm	
pgrp	1	f	0.698	33.48	1.041	0.196	hypoxia	2	0	0	Ucrit1hyp	69.6384
pgrp	1	f	0.698	33.48	1.041	0.196	hypoxia	2	0	0	Ucrit2hyp	
pgrp	1	f	0.698	33.48	1.041	0.196	hypoxia	2	0	0	Ucrit3hyp	
pgrr	1	m	0.492	32.16	1.234	0.175	normoxia	1	0	0	Ucrit1norm	84.70944
pgrr	1	m	0.492	32.16	1.234	0.175	normoxia	1	0	0	Ucrit2norm	
pgrr	1	m	0.492	32.16	1.234	0.175	normoxia	1	0	0	Ucrit3norm	
pgrr	1	m	0.492	32.16	1.234	0.175	normoxia	1	0	0	Ucrit1hyp	72.23136
pgrr	1	m	0.492	32.16	1.234	0.175	normoxia	1	0	0	Ucrit2hyp	
pgrr	1	m	0.492	32.16	1.234	0.175	normoxia	1	0	0	Ucrit3hyp	
pgrr	1	m	0.492	32.16	1.234	0.175	hypoxia	1	0	0	Ucrit1norm	84.70944
pgrr	1	m	0.492	32.16	1.234	0.175	hypoxia	1	0	0	Ucrit2norm	
pgrr	1	m	0.492	32.16	1.234	0.175	hypoxia	1	0	0	Ucrit3norm	
pgrr	1	m	0.492	32.16	1.234	0.175	hypoxia	1	0	0	Ucrit1hyp	72.23136
pgrr	1	m	0.492	32.16	1.234	0.175	hypoxia	1	0	0	Ucrit2hyp	
pgrr	1	m	0.492	32.16	1.234	0.175	hypoxia	1	0	0	Ucrit3hyp	
pgrr	1	m	0.492	32.16	1.234	0.175	normoxia	2	0	0	Ucrit1norm	84.70944
pgrr	1	m	0.492	32.16	1.234	0.175	normoxia	2	0	0	Ucrit2norm	
pgrr	1	m	0.492	32.16	1.234	0.175	normoxia	2	0	0	Ucrit3norm	
pgrr	1	m	0.492	32.16	1.234	0.175	normoxia	2	0	0	Ucrit1hyp	72.23136
pgrr	1	m	0.492	32.16	1.234	0.175	normoxia	2	0	0	Ucrit2hyp	
pgrr	1	m	0.492	32.16	1.234	0.175	normoxia	2	0	0	Ucrit3hyp	
pgrr	1	m	0.492	32.16	1.234	0.175	hypoxia	2	0	0	Ucrit1norm	84.70944
pgrr	1	m	0.492	32.16	1.234	0.175	hypoxia	2	0	0	Ucrit2norm	
pgrr	1	m	0.492	32.16	1.234	0.175	hypoxia	2	0	0	Ucrit3norm	
pgrr	1	m	0.492	32.16	1.234	0.175	hypoxia	2	0	0	Ucrit1hyp	72.23136

pgrr	1	m	0.492	32.16	1.234	0.175	hypoxia	2	0	0	Ucrit2hyp	
pgrr	1	m	0.492	32.16	1.234	0.175	hypoxia	2	0	0	Ucrit3hyp	
pgyg	2	f	0.651	33.25	1.333	0.259	hypoxia	1	0	0	Ucrit1norm	78.76925
pgyg	2	f	0.651	33.25	1.333	0.259	hypoxia	1	0	0	Ucrit2norm	
pgyg	2	f	0.651	33.25	1.333	0.259	hypoxia	1	0	0	Ucrit3norm	
pgyg	2	f	0.651	33.25	1.333	0.259	hypoxia	1	0	0	Ucrit1hyp	64.8375
pgyg	2	f	0.651	33.25	1.333	0.259	hypoxia	1	0	0	Ucrit2hyp	
pgyg	2	f	0.651	33.25	1.333	0.259	hypoxia	1	0	0	Ucrit3hyp	
pgyg	2	f	0.651	33.25	1.333	0.259	normoxia	1	0	0	Ucrit1norm	78.76925
pgyg	2	f	0.651	33.25	1.333	0.259	normoxia	1	0	0	Ucrit2norm	
pgyg	2	f	0.651	33.25	1.333	0.259	normoxia	1	0	0	Ucrit3norm	
pgyg	2	f	0.651	33.25	1.333	0.259	normoxia	1	0	0	Ucrit1hyp	64.8375
pgyg	2	f	0.651	33.25	1.333	0.259	normoxia	1	0	0	Ucrit2hyp	
pgyg	2	f	0.651	33.25	1.333	0.259	normoxia	1	0	0	Ucrit3hyp	
pgyg	2	f	0.651	33.25	1.333	0.259	hypoxia	2	0	0	Ucrit1norm	78.76925
pgyg	2	f	0.651	33.25	1.333	0.259	hypoxia	2	0	0	Ucrit2norm	
pgyg	2	f	0.651	33.25	1.333	0.259	hypoxia	2	0	0	Ucrit3norm	
pgyg	2	f	0.651	33.25	1.333	0.259	hypoxia	2	0	0	Ucrit1hyp	64.8375
pgyg	2	f	0.651	33.25	1.333	0.259	hypoxia	2	0	0	Ucrit2hyp	
pgyg	2	f	0.651	33.25	1.333	0.259	hypoxia	2	0	0	Ucrit3hyp	
pgyg	2	f	0.651	33.25	1.333	0.259	normoxia	2	0	0	Ucrit1norm	78.76925
pgyg	2	f	0.651	33.25	1.333	0.259	normoxia	2	0	0	Ucrit2norm	
pgyg	2	f	0.651	33.25	1.333	0.259	normoxia	2	0	0	Ucrit3norm	
pgyg	2	f	0.651	33.25	1.333	0.259	normoxia	2	0	0	Ucrit1hyp	64.8375
pgyg	2	f	0.651	33.25	1.333	0.259	normoxia	2	0	0	Ucrit2hyp	
pgyg	2	f	0.651	33.25	1.333	0.259	normoxia	2	0	0	Ucrit3hyp	
pgyy	3	f	0.641	33.83	1.171	0.202	hypoxia	1	0.185	1	Ucrit1norm	85.31926
pgyy	3	f	0.641	33.83	1.171	0.202	hypoxia	1	0.185	1	Ucrit2norm	
pgyy	3	f	0.641	33.83	1.171	0.202	hypoxia	1	0.185	1	Ucrit3norm	
pgyy	3	f	0.641	33.83	1.171	0.202	hypoxia	1	0.185	1	Ucrit1hyp	71.65194
pgyy	3	f	0.641	33.83	1.171	0.202	hypoxia	1	0.185	1	Ucrit2hyp	
pgyy	3	f	0.641	33.83	1.171	0.202	hypoxia	1	0.185	1	Ucrit3hyp	
pgyy	3	f	0.641	33.83	1.171	0.202	normoxia	1	0	0	Ucrit1norm	85.31926

pgyy	3	f	0.641	33.83	1.171	0.202	normoxia	1	0	0	Ucrit2norm	
pgyy	3	f	0.641	33.83	1.171	0.202	normoxia	1	0	0	Ucrit3norm	
pgyy	3	f	0.641	33.83	1.171	0.202	normoxia	1	0	0	Ucrit1hyp	71.65194
pgyy	3	f	0.641	33.83	1.171	0.202	normoxia	1	0	0	Ucrit2hyp	
pgyy	3	f	0.641	33.83	1.171	0.202	normoxia	1	0	0	Ucrit3hyp	
pgyy	3	f	0.641	33.83	1.171	0.202	hypoxia	2	0	0	Ucrit1norm	85.31926
pgyy	3	f	0.641	33.83	1.171	0.202	hypoxia	2	0	0	Ucrit2norm	
pgyy	3	f	0.641	33.83	1.171	0.202	hypoxia	2	0	0	Ucrit3norm	
pgyy	3	f	0.641	33.83	1.171	0.202	hypoxia	2	0	0	Ucrit1hyp	71.65194
pgyy	3	f	0.641	33.83	1.171	0.202	hypoxia	2	0	0	Ucrit2hyp	
pgyy	3	f	0.641	33.83	1.171	0.202	hypoxia	2	0	0	Ucrit3hyp	
pgyy	3	f	0.641	33.83	1.171	0.202	normoxia	2	0	0	Ucrit1norm	85.31926
pgyy	3	f	0.641	33.83	1.171	0.202	normoxia	2	0	0	Ucrit2norm	
pgyy	3	f	0.641	33.83	1.171	0.202	normoxia	2	0	0	Ucrit3norm	
pgyy	3	f	0.641	33.83	1.171	0.202	normoxia	2	0	0	Ucrit1hyp	71.65194
pgyy	3	f	0.641	33.83	1.171	0.202	normoxia	2	0	0	Ucrit2hyp	
pgyy	3	f	0.641	33.83	1.171	0.202	normoxia	2	0	0	Ucrit3hyp	
ppgg	2	m	0.674	33.49	1.585	0.208	hypoxia	1	0	0	Ucrit1norm	88.01172
ppgg	2	m	0.674	33.49	1.585	0.208	hypoxia	1	0	0	Ucrit2norm	
ppgg	2	m	0.674	33.49	1.585	0.208	hypoxia	1	0	0	Ucrit3norm	
ppgg	2	m	0.674	33.49	1.585	0.208	hypoxia	1	0	0	Ucrit1hyp	73.27612
ppgg	2	m	0.674	33.49	1.585	0.208	hypoxia	1	0	0	Ucrit2hyp	
ppgg	2	m	0.674	33.49	1.585	0.208	hypoxia	1	0	0	Ucrit3hyp	
ppgg	2	m	0.674	33.49	1.585	0.208	normoxia	1	0	0	Ucrit1norm	88.01172
ppgg	2	m	0.674	33.49	1.585	0.208	normoxia	1	0	0	Ucrit2norm	
ppgg	2	m	0.674	33.49	1.585	0.208	normoxia	1	0	0	Ucrit3norm	
ppgg	2	m	0.674	33.49	1.585	0.208	normoxia	1	0	0	Ucrit1hyp	73.27612
ppgg	2	m	0.674	33.49	1.585	0.208	normoxia	1	0	0	Ucrit2hyp	
ppgg	2	m	0.674	33.49	1.585	0.208	normoxia	1	0	0	Ucrit3hyp	
ppgg	2	m	0.674	33.49	1.585	0.208	hypoxia	2	0	0	Ucrit1norm	88.01172
ppgg	2	m	0.674	33.49	1.585	0.208	hypoxia	2	0	0	Ucrit2norm	
ppgg	2	m	0.674	33.49	1.585	0.208	hypoxia	2	0	0	Ucrit3norm	
ppgg	2	m	0.674	33.49	1.585	0.208	hypoxia	2	0	0	Ucrit1hyp	73.27612

ppgg	2	m	0.674	33.49	1.585	0.208	hypoxia	2	0	0	Ucrit2hyp	
ppgg	2	m	0.674	33.49	1.585	0.208	hypoxia	2	0	0	Ucrit3hyp	
ppgg	2	m	0.674	33.49	1.585	0.208	normoxia	2	0	0	Ucrit1norm	88.01172
ppgg	2	m	0.674	33.49	1.585	0.208	normoxia	2	0	0	Ucrit2norm	
ppgg	2	m	0.674	33.49	1.585	0.208	normoxia	2	0	0	Ucrit3norm	
ppgg	2	m	0.674	33.49	1.585	0.208	normoxia	2	0	0	Ucrit1hyp	73.27612
ppgg	2	m	0.674	33.49	1.585	0.208	normoxia	2	0	0	Ucrit2hyp	
ppgg	2	m	0.674	33.49	1.585	0.208	normoxia	2	0	0	Ucrit3hyp	
ppgr	1	m	0.46	31.4			normoxia	1	0	0	Ucrit1norm	75.6112
ppgr	1	m	0.46	31.4			normoxia	1	0	0	Ucrit2norm	
ppgr	1	m	0.46	31.4			normoxia	1	0	0	Ucrit3norm	
ppgr	1	m	0.46	31.4			normoxia	1	0	0	Ucrit1hyp	61.1672
ppgr	1	m	0.46	31.4			normoxia	1	0	0	Ucrit2hyp	
ppgr	1	m	0.46	31.4			normoxia	1	0	0	Ucrit3hyp	
ppgr	1	m	0.46	31.4			hypoxia	1	0.795	1	Ucrit1norm	75.6112
ppgr	1	m	0.46	31.4			hypoxia	1	0.795	1	Ucrit2norm	
ppgr	1	m	0.46	31.4			hypoxia	1	0.795	1	Ucrit3norm	
ppgr	1	m	0.46	31.4			hypoxia	1	0.795	1	Ucrit1hyp	61.1672
ppgr	1	m	0.46	31.4			hypoxia	1	0.795	1	Ucrit2hyp	
ppgr	1	m	0.46	31.4			hypoxia	1	0.795	1	Ucrit3hyp	
ppgr	1	m	0.46	31.4			normoxia	2	0	0	Ucrit1norm	75.6112
ppgr	1	m	0.46	31.4			normoxia	2	0	0	Ucrit2norm	
ppgr	1	m	0.46	31.4			normoxia	2	0	0	Ucrit3norm	
ppgr	1	m	0.46	31.4			normoxia	2	0	0	Ucrit1hyp	61.1672
ppgr	1	m	0.46	31.4			normoxia	2	0	0	Ucrit2hyp	
ppgr	1	m	0.46	31.4			normoxia	2	0	0	Ucrit3hyp	
ppgr	1	m	0.46	31.4			hypoxia	2	0	0	Ucrit1norm	75.6112
ppgr	1	m	0.46	31.4			hypoxia	2	0	0	Ucrit2norm	
ppgr	1	m	0.46	31.4			hypoxia	2	0	0	Ucrit3norm	
ppgr	1	m	0.46	31.4			hypoxia	2	0	0	Ucrit1hyp	61.1672
ppgr	1	m	0.46	31.4			hypoxia	2	0	0	Ucrit2hyp	
ppgr	1	m	0.46	31.4			hypoxia	2	0	0	Ucrit3hyp	
ppgy	3	m	0.538	30.24	1.444	0.218	hypoxia	1	0.1	1	Ucrit1norm	69.12864

ppgy	3	m	0.538	30.24	1.444	0.218	hypoxia	1	0.1	1	Ucrit2norm	
ppgy	3	m	0.538	30.24	1.444	0.218	hypoxia	1	0.1	1	Ucrit3norm	
ppgy	3	m	0.538	30.24	1.444	0.218	hypoxia	1	0.1	1	Ucrit1hyp	52.37568
ppgy	3	m	0.538	30.24	1.444	0.218	hypoxia	1	0.1	1	Ucrit2hyp	
ppgy	3	m	0.538	30.24	1.444	0.218	hypoxia	1	0.1	1	Ucrit3hyp	
ppgy	3	m	0.538	30.24	1.444	0.218	normoxia	1	0	0	Ucrit1norm	69.12864
ppgy	3	m	0.538	30.24	1.444	0.218	normoxia	1	0	0	Ucrit2norm	
ppgy	3	m	0.538	30.24	1.444	0.218	normoxia	1	0	0	Ucrit3norm	
ppgy	3	m	0.538	30.24	1.444	0.218	normoxia	1	0	0	Ucrit1hyp	52.37568
ppgy	3	m	0.538	30.24	1.444	0.218	normoxia	1	0	0	Ucrit2hyp	
ppgy	3	m	0.538	30.24	1.444	0.218	normoxia	1	0	0	Ucrit3hyp	
ppgy	3	m	0.538	30.24	1.444	0.218	hypoxia	2	0	0	Ucrit1norm	69.12864
ppgy	3	m	0.538	30.24	1.444	0.218	hypoxia	2	0	0	Ucrit2norm	
ppgy	3	m	0.538	30.24	1.444	0.218	hypoxia	2	0	0	Ucrit3norm	
ppgy	3	m	0.538	30.24	1.444	0.218	hypoxia	2	0	0	Ucrit1hyp	52.37568
ppgy	3	m	0.538	30.24	1.444	0.218	hypoxia	2	0	0	Ucrit2hyp	
ppgy	3	m	0.538	30.24	1.444	0.218	hypoxia	2	0	0	Ucrit3hyp	
ppgy	3	m	0.538	30.24	1.444	0.218	normoxia	2	0	0	Ucrit1norm	69.12864
ppgy	3	m	0.538	30.24	1.444	0.218	normoxia	2	0	0	Ucrit2norm	
ppgy	3	m	0.538	30.24	1.444	0.218	normoxia	2	0	0	Ucrit3norm	
ppgy	3	m	0.538	30.24	1.444	0.218	normoxia	2	0	0	Ucrit1hyp	52.37568
ppgy	3	m	0.538	30.24	1.444	0.218	normoxia	2	0	0	Ucrit2hyp	
ppgy	3	m	0.538	30.24	1.444	0.218	normoxia	2	0	0	Ucrit3hyp	
pppg	4	f	0.52	30.71			normoxia	1	0	0	Ucrit1norm	84.97457
pppg	4	f	0.52	30.71			normoxia	1	0	0	Ucrit2norm	81.65789
pppg	4	f	0.52	30.71			normoxia	1	0	0	Ucrit3norm	82.45635
pppg	4	f	0.52	30.71			normoxia	1	0	0	Ucrit1hyp	64.15319
pppg	4	f	0.52	30.71			normoxia	1	0	0	Ucrit2hyp	70.23377
pppg	4	f	0.52	30.71			normoxia	1	0	0	Ucrit3hyp	
pppg	4	f	0.52	30.71			hypoxia	1	0.148333333	1	Ucrit1norm	84.97457
pppg	4	f	0.52	30.71			hypoxia	1	0.148333333	1	Ucrit2norm	81.65789
pppg	4	f	0.52	30.71			hypoxia	1	0.148333333	1	Ucrit3norm	82.45635
pppg	4	f	0.52	30.71			hypoxia	1	0.148333333	1	Ucrit1hyp	64.15319

pppg	4	f	0.52	30.71			hypoxia	1	0.148333333	1	Ucrit2hyp	70.23377
pppg	4	f	0.52	30.71			hypoxia	1	0.148333333	1	Ucrit3hyp	
pppg	4	f	0.52	30.71			normoxia	2	0	0	Ucrit1norm	84.97457
pppg	4	f	0.52	30.71			normoxia	2	0	0	Ucrit2norm	81.65789
pppg	4	f	0.52	30.71			normoxia	2	0	0	Ucrit3norm	82.45635
pppg	4	f	0.52	30.71			normoxia	2	0	0	Ucrit1hyp	64.15319
pppg	4	f	0.52	30.71			normoxia	2	0	0	Ucrit2hyp	70.23377
pppg	4	f	0.52	30.71			normoxia	2	0	0	Ucrit3hyp	
pppg	4	f	0.52	30.71			hypoxia	2	0	0	Ucrit1norm	84.97457
pppg	4	f	0.52	30.71			hypoxia	2	0	0	Ucrit2norm	81.65789
pppg	4	f	0.52	30.71			hypoxia	2	0	0	Ucrit3norm	82.45635
pppg	4	f	0.52	30.71			hypoxia	2	0	0	Ucrit1hyp	64.15319
pppg	4	f	0.52	30.71			hypoxia	2	0	0	Ucrit2hyp	70.23377
pppg	4	f	0.52	30.71			hypoxia	2	0	0	Ucrit3hyp	
pppr	4	f	0.622	33.82	0.706	0.126	normoxia	1	0	0	Ucrit1norm	73.52468
pppr	4	f	0.622	33.82	0.706	0.126	normoxia	1	0	0	Ucrit2norm	74.09962
pppr	4	f	0.622	33.82	0.706	0.126	normoxia	1	0	0	Ucrit3norm	75.7568
pppr	4	f	0.622	33.82	0.706	0.126	normoxia	1	0	0	Ucrit1hyp	64.5962
pppr	4	f	0.622	33.82	0.706	0.126	normoxia	1	0	0	Ucrit2hyp	67.03124
pppr	4	f	0.622	33.82	0.706	0.126	normoxia	1	0	0	Ucrit3hyp	65.88136
pppr	4	f	0.622	33.82	0.706	0.126	hypoxia	1	0	0	Ucrit1norm	73.52468
pppr	4	f	0.622	33.82	0.706	0.126	hypoxia	1	0	0	Ucrit2norm	74.09962
pppr	4	f	0.622	33.82	0.706	0.126	hypoxia	1	0	0	Ucrit3norm	75.7568
pppr	4	f	0.622	33.82	0.706	0.126	hypoxia	1	0	0	Ucrit1hyp	64.5962
pppr	4	f	0.622	33.82	0.706	0.126	hypoxia	1	0	0	Ucrit2hyp	67.03124
pppr	4	f	0.622	33.82	0.706	0.126	hypoxia	1	0	0	Ucrit3hyp	65.88136
pppr	4	f	0.622	33.82	0.706	0.126	normoxia	2	0	0	Ucrit1norm	73.52468
pppr	4	f	0.622	33.82	0.706	0.126	normoxia	2	0	0	Ucrit2norm	74.09962
pppr	4	f	0.622	33.82	0.706	0.126	normoxia	2	0	0	Ucrit3norm	75.7568
pppr	4	f	0.622	33.82	0.706	0.126	normoxia	2	0	0	Ucrit1hyp	64.5962
pppr	4	f	0.622	33.82	0.706	0.126	normoxia	2	0	0	Ucrit2hyp	67.03124
pppr	4	f	0.622	33.82	0.706	0.126	normoxia	2	0	0	Ucrit3hyp	65.88136
pppr	4	f	0.622	33.82	0.706	0.126	hypoxia	2	0.363333333	1	Ucrit1norm	73.52468

pppr	4	f	0.622	33.82	0.706	0.126	hypoxia	2	0.363333333	1	Ucrit2norm	74.09962
pppr	4	f	0.622	33.82	0.706	0.126	hypoxia	2	0.363333333	1	Ucrit3norm	75.7568
pppr	4	f	0.622	33.82	0.706	0.126	hypoxia	2	0.363333333	1	Ucrit1hyp	64.5962
pppr	4	f	0.622	33.82	0.706	0.126	hypoxia	2	0.363333333	1	Ucrit2hyp	67.03124
pppr	4	f	0.622	33.82	0.706	0.126	hypoxia	2	0.363333333	1	Ucrit3hyp	65.88136
pprg	4	f	0.479	30.3	0.91	0.136	normoxia	1	0	0	Ucrit1norm	67.0539
pprg	4	f	0.479	30.3	0.91	0.136	normoxia	1	0	0	Ucrit2norm	78.2043
pprg	4	f	0.479	30.3	0.91	0.136	normoxia	1	0	0	Ucrit3norm	80.6283
pprg	4	f	0.479	30.3	0.91	0.136	normoxia	1	0	0	Ucrit1hyp	68.9931
pprg	4	f	0.479	30.3	0.91	0.136	normoxia	1	0	0	Ucrit2hyp	68.7507
pprg	4	f	0.479	30.3	0.91	0.136	normoxia	1	0	0	Ucrit3hyp	70.0839
pprg	4	f	0.479	30.3	0.91	0.136	hypoxia	1	0	0	Ucrit1norm	67.0539
pprg	4	f	0.479	30.3	0.91	0.136	hypoxia	1	0	0	Ucrit2norm	78.2043
pprg	4	f	0.479	30.3	0.91	0.136	hypoxia	1	0	0	Ucrit3norm	80.6283
pprg	4	f	0.479	30.3	0.91	0.136	hypoxia	1	0	0	Ucrit1hyp	68.9931
pprg	4	f	0.479	30.3	0.91	0.136	hypoxia	1	0	0	Ucrit2hyp	68.7507
pprg	4	f	0.479	30.3	0.91	0.136	hypoxia	1	0	0	Ucrit3hyp	70.0839
pprg	4	f	0.479	30.3	0.91	0.136	normoxia	2	0	0	Ucrit1norm	67.0539
pprg	4	f	0.479	30.3	0.91	0.136	normoxia	2	0	0	Ucrit2norm	78.2043
pprg	4	f	0.479	30.3	0.91	0.136	normoxia	2	0	0	Ucrit3norm	80.6283
pprg	4	f	0.479	30.3	0.91	0.136	normoxia	2	0	0	Ucrit1hyp	68.9931
pprg	4	f	0.479	30.3	0.91	0.136	normoxia	2	0	0	Ucrit2hyp	68.7507
pprg	4	f	0.479	30.3	0.91	0.136	normoxia	2	0	0	Ucrit3hyp	70.0839
pprg	4	f	0.479	30.3	0.91	0.136	hypoxia	2	0.903333333	1	Ucrit1norm	67.0539
pprg	4	f	0.479	30.3	0.91	0.136	hypoxia	2	0.903333333	1	Ucrit2norm	78.2043
pprg	4	f	0.479	30.3	0.91	0.136	hypoxia	2	0.903333333	1	Ucrit3norm	80.6283
pprg	4	f	0.479	30.3	0.91	0.136	hypoxia	2	0.903333333	1	Ucrit1hyp	68.9931
pprg	4	f	0.479	30.3	0.91	0.136	hypoxia	2	0.903333333	1	Ucrit2hyp	68.7507
pprg	4	f	0.479	30.3	0.91	0.136	hypoxia	2	0.903333333	1	Ucrit3hyp	70.0839
pprr	1	m	0.674	34.41	1.382	0.15	normoxia	1	0	0	Ucrit1norm	89.53482
pprr	1	m	0.674	34.41	1.382	0.15	normoxia	1	0	0	Ucrit2norm	
pprr	1	m	0.674	34.41	1.382	0.15	normoxia	1	0	0	Ucrit3norm	
pprr	1	m	0.674	34.41	1.382	0.15	normoxia	1	0	0	Ucrit1hyp	70.36845

pprr	1	m	0.674	34.41	1.382	0.15	normoxia	1	0	0	Ucrit2hyp	
pprr	1	m	0.674	34.41	1.382	0.15	normoxia	1	0	0	Ucrit3hyp	
pprr	1	m	0.674	34.41	1.382	0.15	hypoxia	1	0.723333333	1	Ucrit1norm	89.53482
pprr	1	m	0.674	34.41	1.382	0.15	hypoxia	1	0.723333333	1	Ucrit2norm	
pprr	1	m	0.674	34.41	1.382	0.15	hypoxia	1	0.723333333	1	Ucrit3norm	
pprr	1	m	0.674	34.41	1.382	0.15	hypoxia	1	0.723333333	1	Ucrit1hyp	70.36845
pprr	1	m	0.674	34.41	1.382	0.15	hypoxia	1	0.723333333	1	Ucrit2hyp	
pprr	1	m	0.674	34.41	1.382	0.15	hypoxia	1	0.723333333	1	Ucrit3hyp	
pprr	1	m	0.674	34.41	1.382	0.15	normoxia	2	0	0	Ucrit1norm	89.53482
pprr	1	m	0.674	34.41	1.382	0.15	normoxia	2	0	0	Ucrit2norm	
pprr	1	m	0.674	34.41	1.382	0.15	normoxia	2	0	0	Ucrit3norm	
pprr	1	m	0.674	34.41	1.382	0.15	normoxia	2	0	0	Ucrit1hyp	70.36845
pprr	1	m	0.674	34.41	1.382	0.15	normoxia	2	0	0	Ucrit2hyp	
pprr	1	m	0.674	34.41	1.382	0.15	normoxia	2	0	0	Ucrit3hyp	
pprr	1	m	0.674	34.41	1.382	0.15	hypoxia	2	0	0	Ucrit1norm	89.53482
pprr	1	m	0.674	34.41	1.382	0.15	hypoxia	2	0	0	Ucrit2norm	
pprr	1	m	0.674	34.41	1.382	0.15	hypoxia	2	0	0	Ucrit3norm	
pprr	1	m	0.674	34.41	1.382	0.15	hypoxia	2	0	0	Ucrit1hyp	70.36845
pprr	1	m	0.674	34.41	1.382	0.15	hypoxia	2	0	0	Ucrit2hyp	
pprr	1	m	0.674	34.41	1.382	0.15	hypoxia	2	0	0	Ucrit3hyp	
ppry	4	m	0.625	33.59			normoxia	1	0	0	Ucrit1norm	83.06807
ppry	4	m	0.625	33.59			normoxia	1	0	0	Ucrit2norm	84.14295
ppry	4	m	0.625	33.59			normoxia	1	0	0	Ucrit3norm	94.79098
ppry	4	m	0.625	33.59			normoxia	1	0	0	Ucrit1hyp	68.75873
ppry	4	m	0.625	33.59			normoxia	1	0	0	Ucrit2hyp	71.64747
ppry	4	m	0.625	33.59			normoxia	1	0	0	Ucrit3hyp	69.69925
ppry	4	m	0.625	33.59			hypoxia	1	0.286666667	1	Ucrit1norm	83.06807
ppry	4	m	0.625	33.59			hypoxia	1	0.286666667	1	Ucrit2norm	84.14295
ppry	4	m	0.625	33.59			hypoxia	1	0.286666667	1	Ucrit3norm	94.79098
ppry	4	m	0.625	33.59			hypoxia	1	0.286666667	1	Ucrit1hyp	68.75873
ppry	4	m	0.625	33.59			hypoxia	1	0.286666667	1	Ucrit2hyp	71.64747
ppry	4	m	0.625	33.59			hypoxia	1	0.286666667	1	Ucrit3hyp	69.69925
ppry	4	m	0.625	33.59			normoxia	2	0	0	Ucrit1norm	83.06807

ppry	4	m	0.625	33.59			normoxia	2	0	0	Ucrit2norm	84.14295
ppry	4	m	0.625	33.59			normoxia	2	0	0	Ucrit3norm	94.79098
ppry	4	m	0.625	33.59			normoxia	2	0	0	Ucrit1hyp	68.75873
ppry	4	m	0.625	33.59			normoxia	2	0	0	Ucrit2hyp	71.64747
ppry	4	m	0.625	33.59			normoxia	2	0	0	Ucrit3hyp	69.69925
ppry	4	m	0.625	33.59			hypoxia	2	0	0	Ucrit1norm	83.06807
ppry	4	m	0.625	33.59			hypoxia	2	0	0	Ucrit2norm	84.14295
ppry	4	m	0.625	33.59			hypoxia	2	0	0	Ucrit3norm	94.79098
ppry	4	m	0.625	33.59			hypoxia	2	0	0	Ucrit1hyp	68.75873
ppry	4	m	0.625	33.59			hypoxia	2	0	0	Ucrit2hyp	71.64747
ppry	4	m	0.625	33.59			hypoxia	2	0	0	Ucrit3hyp	69.69925
prgy	2	f	0.71	34.99	0.878	0.182	hypoxia	1	0	0	Ucrit1norm	74.56369
prgy	2	f	0.71	34.99	0.878	0.182	hypoxia	1	0	0	Ucrit2norm	
prgy	2	f	0.71	34.99	0.878	0.182	hypoxia	1	0	0	Ucrit3norm	
prgy	2	f	0.71	34.99	0.878	0.182	hypoxia	1	0	0	Ucrit1hyp	62.21222
prgy	2	f	0.71	34.99	0.878	0.182	hypoxia	1	0	0	Ucrit2hyp	
prgy	2	f	0.71	34.99	0.878	0.182	hypoxia	1	0	0	Ucrit3hyp	
prgy	2	f	0.71	34.99	0.878	0.182	normoxia	1	0	0	Ucrit1norm	74.56369
prgy	2	f	0.71	34.99	0.878	0.182	normoxia	1	0	0	Ucrit2norm	
prgy	2	f	0.71	34.99	0.878	0.182	normoxia	1	0	0	Ucrit3norm	
prgy	2	f	0.71	34.99	0.878	0.182	normoxia	1	0	0	Ucrit1hyp	62.21222
prgy	2	f	0.71	34.99	0.878	0.182	normoxia	1	0	0	Ucrit2hyp	
prgy	2	f	0.71	34.99	0.878	0.182	normoxia	1	0	0	Ucrit3hyp	
prgy	2	f	0.71	34.99	0.878	0.182	hypoxia	2	0	0	Ucrit1norm	74.56369
prgy	2	f	0.71	34.99	0.878	0.182	hypoxia	2	0	0	Ucrit2norm	
prgy	2	f	0.71	34.99	0.878	0.182	hypoxia	2	0	0	Ucrit3norm	
prgy	2	f	0.71	34.99	0.878	0.182	hypoxia	2	0	0	Ucrit1hyp	62.21222
prgy	2	f	0.71	34.99	0.878	0.182	hypoxia	2	0	0	Ucrit2hyp	
prgy	2	f	0.71	34.99	0.878	0.182	hypoxia	2	0	0	Ucrit3hyp	
prgy	2	f	0.71	34.99	0.878	0.182	normoxia	2	0	0	Ucrit1norm	74.56369
prgy	2	f	0.71	34.99	0.878	0.182	normoxia	2	0	0	Ucrit2norm	
prgy	2	f	0.71	34.99	0.878	0.182	normoxia	2	0	0	Ucrit3norm	
prgy	2	f	0.71	34.99	0.878	0.182	normoxia	2	0	0	Ucrit1hyp	62.21222

prgy	2	f	0.71	34.99	0.878	0.182	normoxia	2	0	0	Ucrit2hyp	
prgy	2	f	0.71	34.99	0.878	0.182	normoxia	2	0	0	Ucrit3hyp	
prpg	3	f	0.675	34.13			hypoxia	1	0.651666667	1	Ucrit1norm	83.58437
prpg	3	f	0.675	34.13			hypoxia	1	0.651666667	1	Ucrit2norm	
prpg	3	f	0.675	34.13			hypoxia	1	0.651666667	1	Ucrit3norm	
prpg	3	f	0.675	34.13			hypoxia	1	0.651666667	1	Ucrit1hyp	
prpg	3	f	0.675	34.13			hypoxia	1	0.651666667	1	Ucrit2hyp	
prpg	3	f	0.675	34.13			hypoxia	1	0.651666667	1	Ucrit3hyp	
prpg	3	f	0.675	34.13			normoxia	1	0	0	Ucrit1norm	83.58437
prpg	3	f	0.675	34.13			normoxia	1	0	0	Ucrit2norm	
prpg	3	f	0.675	34.13			normoxia	1	0	0	Ucrit3norm	
prpg	3	f	0.675	34.13			normoxia	1	0	0	Ucrit1hyp	
prpg	3	f	0.675	34.13			normoxia	1	0	0	Ucrit2hyp	
prpg	3	f	0.675	34.13			normoxia	1	0	0	Ucrit3hyp	
prpg	3	f	0.675	34.13			hypoxia	2	0	0	Ucrit1norm	83.58437
prpg	3	f	0.675	34.13			hypoxia	2	0	0	Ucrit2norm	
prpg	3	f	0.675	34.13			hypoxia	2	0	0	Ucrit3norm	
prpg	3	f	0.675	34.13			hypoxia	2	0	0	Ucrit1hyp	
prpg	3	f	0.675	34.13			hypoxia	2	0	0	Ucrit2hyp	
prpg	3	f	0.675	34.13			hypoxia	2	0	0	Ucrit3hyp	
prpg	3	f	0.675	34.13			normoxia	2	0	0	Ucrit1norm	83.58437
prpg	3	f	0.675	34.13			normoxia	2	0	0	Ucrit2norm	
prpg	3	f	0.675	34.13			normoxia	2	0	0	Ucrit3norm	
prpg	3	f	0.675	34.13			normoxia	2	0	0	Ucrit1hyp	
prpg	3	f	0.675	34.13			normoxia	2	0	0	Ucrit2hyp	
prpg	3	f	0.675	34.13			normoxia	2	0	0	Ucrit3hyp	
prpp	2	m	0.407	29.92	1.354	0.172	hypoxia	1	0.861666667	1	Ucrit1norm	88.20416
prpp	2	m	0.407	29.92	1.354	0.172	hypoxia	1	0.861666667	1	Ucrit2norm	
prpp	2	m	0.407	29.92	1.354	0.172	hypoxia	1	0.861666667	1	Ucrit3norm	
prpp	2	m	0.407	29.92	1.354	0.172	hypoxia	1	0.861666667	1	Ucrit1hyp	73.39376
prpp	2	m	0.407	29.92	1.354	0.172	hypoxia	1	0.861666667	1	Ucrit2hyp	
prpp	2	m	0.407	29.92	1.354	0.172	hypoxia	1	0.861666667	1	Ucrit3hyp	
prpp	2	m	0.407	29.92	1.354	0.172	normoxia	1	0	0	Ucrit1norm	88.20416

prpp	2	m	0.407	29.92	1.354	0.172	normoxia	1	0	0	Ucrit2norm	
prpp	2	m	0.407	29.92	1.354	0.172	normoxia	1	0	0	Ucrit3norm	
prpp	2	m	0.407	29.92	1.354	0.172	normoxia	1	0	0	Ucrit1hyp	73.39376
prpp	2	m	0.407	29.92	1.354	0.172	normoxia	1	0	0	Ucrit2hyp	
prpp	2	m	0.407	29.92	1.354	0.172	normoxia	1	0	0	Ucrit3hyp	
prpp	2	m	0.407	29.92	1.354	0.172	hypoxia	2	0.911666667	1	Ucrit1norm	88.20416
prpp	2	m	0.407	29.92	1.354	0.172	hypoxia	2	0.911666667	1	Ucrit2norm	
prpp	2	m	0.407	29.92	1.354	0.172	hypoxia	2	0.911666667	1	Ucrit3norm	
prpp	2	m	0.407	29.92	1.354	0.172	hypoxia	2	0.911666667	1	Ucrit1hyp	73.39376
prpp	2	m	0.407	29.92	1.354	0.172	hypoxia	2	0.911666667	1	Ucrit2hyp	
prpp	2	m	0.407	29.92	1.354	0.172	hypoxia	2	0.911666667	1	Ucrit3hyp	
prpp	2	m	0.407	29.92	1.354	0.172	normoxia	2	0.846666667	1	Ucrit1norm	88.20416
prpp	2	m	0.407	29.92	1.354	0.172	normoxia	2	0.846666667	1	Ucrit2norm	
prpp	2	m	0.407	29.92	1.354	0.172	normoxia	2	0.846666667	1	Ucrit3norm	
prpp	2	m	0.407	29.92	1.354	0.172	normoxia	2	0.846666667	1	Ucrit1hyp	73.39376
prpp	2	m	0.407	29.92	1.354	0.172	normoxia	2	0.846666667	1	Ucrit2hyp	
prpp	2	m	0.407	29.92	1.354	0.172	normoxia	2	0.846666667	1	Ucrit3hyp	
prpr	4	f	0.644	34.84	1.19	0.127	normoxia	1	0	0	Ucrit1norm	81.24688
prpr	4	f	0.644	34.84	1.19	0.127	normoxia	1	0	0	Ucrit2norm	77.10092
prpr	4	f	0.644	34.84	1.19	0.127	normoxia	1	0	0	Ucrit3norm	78.94744
prpr	4	f	0.644	34.84	1.19	0.127	normoxia	1	0	0	Ucrit1hyp	60.16868
prpr	4	f	0.644	34.84	1.19	0.127	normoxia	1	0	0	Ucrit2hyp	61.38808
prpr	4	f	0.644	34.84	1.19	0.127	normoxia	1	0	0	Ucrit3hyp	74.7318
prpr	4	f	0.644	34.84	1.19	0.127	hypoxia	1	0	0	Ucrit1norm	81.24688
prpr	4	f	0.644	34.84	1.19	0.127	hypoxia	1	0	0	Ucrit2norm	77.10092
prpr	4	f	0.644	34.84	1.19	0.127	hypoxia	1	0	0	Ucrit3norm	78.94744
prpr	4	f	0.644	34.84	1.19	0.127	hypoxia	1	0	0	Ucrit1hyp	60.16868
prpr	4	f	0.644	34.84	1.19	0.127	hypoxia	1	0	0	Ucrit2hyp	61.38808
prpr	4	f	0.644	34.84	1.19	0.127	hypoxia	1	0	0	Ucrit3hyp	74.7318
prpr	4	f	0.644	34.84	1.19	0.127	normoxia	2	0	0	Ucrit1norm	81.24688
prpr	4	f	0.644	34.84	1.19	0.127	normoxia	2	0	0	Ucrit2norm	77.10092
prpr	4	f	0.644	34.84	1.19	0.127	normoxia	2	0	0	Ucrit3norm	78.94744
prpr	4	f	0.644	34.84	1.19	0.127	normoxia	2	0	0	Ucrit1hyp	60.16868

prpr	4	f	0.644	34.84	1.19	0.127	normoxia	2	0	0	Ucrit2hyp	61.38808
prpr	4	f	0.644	34.84	1.19	0.127	normoxia	2	0	0	Ucrit3hyp	74.7318
prpr	4	f	0.644	34.84	1.19	0.127	hypoxia	2	0	0	Ucrit1norm	81.24688
prpr	4	f	0.644	34.84	1.19	0.127	hypoxia	2	0	0	Ucrit2norm	77.10092
prpr	4	f	0.644	34.84	1.19	0.127	hypoxia	2	0	0	Ucrit3norm	78.94744
prpr	4	f	0.644	34.84	1.19	0.127	hypoxia	2	0	0	Ucrit1hyp	60.16868
prpr	4	f	0.644	34.84	1.19	0.127	hypoxia	2	0	0	Ucrit2hyp	61.38808
prpr	4	f	0.644	34.84	1.19	0.127	hypoxia	2	0	0	Ucrit3hyp	74.7318
prpy	2	f	0.65	35.48	1.281	0.132	hypoxia	1	0	0	Ucrit1norm	76.14008
prpy	2	f	0.65	35.48	1.281	0.132	hypoxia	1	0	0	Ucrit2norm	
prpy	2	f	0.65	35.48	1.281	0.132	hypoxia	1	0	0	Ucrit3norm	
prpy	2	f	0.65	35.48	1.281	0.132	hypoxia	1	0	0	Ucrit1hyp	61.06108
prpy	2	f	0.65	35.48	1.281	0.132	hypoxia	1	0	0	Ucrit2hyp	
prpy	2	f	0.65	35.48	1.281	0.132	hypoxia	1	0	0	Ucrit3hyp	
prpy	2	f	0.65	35.48	1.281	0.132	normoxia	1	0	0	Ucrit1norm	76.14008
prpy	2	f	0.65	35.48	1.281	0.132	normoxia	1	0	0	Ucrit2norm	
prpy	2	f	0.65	35.48	1.281	0.132	normoxia	1	0	0	Ucrit3norm	
prpy	2	f	0.65	35.48	1.281	0.132	normoxia	1	0	0	Ucrit1hyp	61.06108
prpy	2	f	0.65	35.48	1.281	0.132	normoxia	1	0	0	Ucrit2hyp	
prpy	2	f	0.65	35.48	1.281	0.132	normoxia	1	0	0	Ucrit3hyp	
prpy	2	f	0.65	35.48	1.281	0.132	hypoxia	2	0.783333333	1	Ucrit1norm	76.14008
prpy	2	f	0.65	35.48	1.281	0.132	hypoxia	2	0.783333333	1	Ucrit2norm	
prpy	2	f	0.65	35.48	1.281	0.132	hypoxia	2	0.783333333	1	Ucrit3norm	
prpy	2	f	0.65	35.48	1.281	0.132	hypoxia	2	0.783333333	1	Ucrit1hyp	61.06108
prpy	2	f	0.65	35.48	1.281	0.132	hypoxia	2	0.783333333	1	Ucrit2hyp	
prpy	2	f	0.65	35.48	1.281	0.132	hypoxia	2	0.783333333	1	Ucrit3hyp	
prpy	2	f	0.65	35.48	1.281	0.132	normoxia	2	0	0	Ucrit1norm	76.14008
prpy	2	f	0.65	35.48	1.281	0.132	normoxia	2	0	0	Ucrit2norm	
prpy	2	f	0.65	35.48	1.281	0.132	normoxia	2	0	0	Ucrit3norm	
prpy	2	f	0.65	35.48	1.281	0.132	normoxia	2	0	0	Ucrit1hyp	61.06108
prpy	2	f	0.65	35.48	1.281	0.132	normoxia	2	0	0	Ucrit2hyp	
prpy	2	f	0.65	35.48	1.281	0.132	normoxia	2	0	0	Ucrit3hyp	
prrg	2	m	0.668	34.89	0.882	0.147	hypoxia	1	0	0	Ucrit1norm	65.5932

prrg	2	m	0.668	34.89	0.882	0.147	hypoxia	1	0	0	Ucrit2norm	
prrg	2	m	0.668	34.89	0.882	0.147	hypoxia	1	0	0	Ucrit3norm	
prrg	2	m	0.668	34.89	0.882	0.147	hypoxia	1	0	0	Ucrit1hyp	56.27757
prrg	2	m	0.668	34.89	0.882	0.147	hypoxia	1	0	0	Ucrit2hyp	
prrg	2	m	0.668	34.89	0.882	0.147	hypoxia	1	0	0	Ucrit3hyp	
prrg	2	m	0.668	34.89	0.882	0.147	normoxia	1	0	0	Ucrit1norm	65.5932
prrg	2	m	0.668	34.89	0.882	0.147	normoxia	1	0	0	Ucrit2norm	
prrg	2	m	0.668	34.89	0.882	0.147	normoxia	1	0	0	Ucrit3norm	
prrg	2	m	0.668	34.89	0.882	0.147	normoxia	1	0	0	Ucrit1hyp	56.27757
prrg	2	m	0.668	34.89	0.882	0.147	normoxia	1	0	0	Ucrit2hyp	
prrg	2	m	0.668	34.89	0.882	0.147	normoxia	1	0	0	Ucrit3hyp	
prrg	2	m	0.668	34.89	0.882	0.147	hypoxia	2	0	0	Ucrit1norm	65.5932
prrg	2	m	0.668	34.89	0.882	0.147	hypoxia	2	0	0	Ucrit2norm	
prrg	2	m	0.668	34.89	0.882	0.147	hypoxia	2	0	0	Ucrit3norm	
prrg	2	m	0.668	34.89	0.882	0.147	hypoxia	2	0	0	Ucrit1hyp	56.27757
prrg	2	m	0.668	34.89	0.882	0.147	hypoxia	2	0	0	Ucrit2hyp	
prrg	2	m	0.668	34.89	0.882	0.147	hypoxia	2	0	0	Ucrit3hyp	
prrg	2	m	0.668	34.89	0.882	0.147	normoxia	2	0	0	Ucrit1norm	65.5932
prrg	2	m	0.668	34.89	0.882	0.147	normoxia	2	0	0	Ucrit2norm	
prrg	2	m	0.668	34.89	0.882	0.147	normoxia	2	0	0	Ucrit3norm	
prrg	2	m	0.668	34.89	0.882	0.147	normoxia	2	0	0	Ucrit1hyp	56.27757
prrg	2	m	0.668	34.89	0.882	0.147	normoxia	2	0	0	Ucrit2hyp	
prrg	2	m	0.668	34.89	0.882	0.147	normoxia	2	0	0	Ucrit3hyp	
prry	2	f	0.676	35.96	0.991	0.207	hypoxia	1	0	0	Ucrit1norm	70.62544
prry	2	f	0.676	35.96	0.991	0.207	hypoxia	1	0	0	Ucrit2norm	
prry	2	f	0.676	35.96	0.991	0.207	hypoxia	1	0	0	Ucrit3norm	
prry	2	f	0.676	35.96	0.991	0.207	hypoxia	1	0	0	Ucrit1hyp	58.83056
prry	2	f	0.676	35.96	0.991	0.207	hypoxia	1	0	0	Ucrit2hyp	
prry	2	f	0.676	35.96	0.991	0.207	hypoxia	1	0	0	Ucrit3hyp	
prry	2	f	0.676	35.96	0.991	0.207	normoxia	1	0	0	Ucrit1norm	70.62544
prry	2	f	0.676	35.96	0.991	0.207	normoxia	1	0	0	Ucrit2norm	
prry	2	f	0.676	35.96	0.991	0.207	normoxia	1	0	0	Ucrit3norm	
prry	2	f	0.676	35.96	0.991	0.207	normoxia	1	0	0	Ucrit1hyp	58.83056

prry	2	f	0.676	35.96	0.991	0.207	normoxia	1	0	0	Ucrit2hyp	
prry	2	f	0.676	35.96	0.991	0.207	normoxia	1	0	0	Ucrit3hyp	
prry	2	f	0.676	35.96	0.991	0.207	hypoxia	2	0	0	Ucrit1norm	70.62544
prry	2	f	0.676	35.96	0.991	0.207	hypoxia	2	0	0	Ucrit2norm	
prry	2	f	0.676	35.96	0.991	0.207	hypoxia	2	0	0	Ucrit3norm	
prry	2	f	0.676	35.96	0.991	0.207	hypoxia	2	0	0	Ucrit1hyp	58.83056
prry	2	f	0.676	35.96	0.991	0.207	hypoxia	2	0	0	Ucrit2hyp	
prry	2	f	0.676	35.96	0.991	0.207	hypoxia	2	0	0	Ucrit3hyp	
prry	2	f	0.676	35.96	0.991	0.207	normoxia	2	0	0	Ucrit1norm	70.62544
prry	2	f	0.676	35.96	0.991	0.207	normoxia	2	0	0	Ucrit2norm	
prry	2	f	0.676	35.96	0.991	0.207	normoxia	2	0	0	Ucrit3norm	
prry	2	f	0.676	35.96	0.991	0.207	normoxia	2	0	0	Ucrit1hyp	58.83056
prry	2	f	0.676	35.96	0.991	0.207	normoxia	2	0	0	Ucrit2hyp	
prry	2	f	0.676	35.96	0.991	0.207	normoxia	2	0	0	Ucrit3hyp	
pryg	4	m	0.623	34.28	1.096	0.147	normoxia	1	0	0	Ucrit1norm	75.7588
pryg	4	m	0.623	34.28	1.096	0.147	normoxia	1	0	0	Ucrit2norm	72.19368
pryg	4	m	0.623	34.28	1.096	0.147	normoxia	1	0	0	Ucrit3norm	73.73628
pryg	4	m	0.623	34.28	1.096	0.147	normoxia	1	0	0	Ucrit1hyp	66.09184
pryg	4	m	0.623	34.28	1.096	0.147	normoxia	1	0	0	Ucrit2hyp	64.68636
pryg	4	m	0.623	34.28	1.096	0.147	normoxia	1	0	0	Ucrit3hyp	60.57276
pryg	4	m	0.623	34.28	1.096	0.147	hypoxia	1	0.743333333	1	Ucrit1norm	75.7588
pryg	4	m	0.623	34.28	1.096	0.147	hypoxia	1	0.743333333	1	Ucrit2norm	72.19368
pryg	4	m	0.623	34.28	1.096	0.147	hypoxia	1	0.743333333	1	Ucrit3norm	73.73628
pryg	4	m	0.623	34.28	1.096	0.147	hypoxia	1	0.743333333	1	Ucrit1hyp	66.09184
pryg	4	m	0.623	34.28	1.096	0.147	hypoxia	1	0.743333333	1	Ucrit2hyp	64.68636
pryg	4	m	0.623	34.28	1.096	0.147	hypoxia	1	0.743333333	1	Ucrit3hyp	60.57276
pryg	4	m	0.623	34.28	1.096	0.147	normoxia	2	0	0	Ucrit1norm	75.7588
pryg	4	m	0.623	34.28	1.096	0.147	normoxia	2	0	0	Ucrit2norm	72.19368
pryg	4	m	0.623	34.28	1.096	0.147	normoxia	2	0	0	Ucrit3norm	73.73628
pryg	4	m	0.623	34.28	1.096	0.147	normoxia	2	0	0	Ucrit1hyp	66.09184
pryg	4	m	0.623	34.28	1.096	0.147	normoxia	2	0	0	Ucrit2hyp	64.68636
pryg	4	m	0.623	34.28	1.096	0.147	normoxia	2	0	0	Ucrit3hyp	60.57276
pryg	4	m	0.623	34.28	1.096	0.147	hypoxia	2	0.563333333	1	Ucrit1norm	75.7588

pryg	4	m	0.623	34.28	1.096	0.147	hypoxia	2	0.563333333	1	Ucrit2norm	72.19368
pryg	4	m	0.623	34.28	1.096	0.147	hypoxia	2	0.563333333	1	Ucrit3norm	73.73628
pryg	4	m	0.623	34.28	1.096	0.147	hypoxia	2	0.563333333	1	Ucrit1hyp	66.09184
pryg	4	m	0.623	34.28	1.096	0.147	hypoxia	2	0.563333333	1	Ucrit2hyp	64.68636
pryg	4	m	0.623	34.28	1.096	0.147	hypoxia	2	0.563333333	1	Ucrit3hyp	60.57276
pryp	4	m	0.381	29.47			normoxia	1	0.895	1	Ucrit1norm	64.00884
pryp	4	m	0.381	29.47			normoxia	1	0.895	1	Ucrit2norm	71.99521
pryp	4	m	0.381	29.47			normoxia	1	0.895	1	Ucrit3norm	69.69655
pryp	4	m	0.381	29.47			normoxia	1	0.895	1	Ucrit1hyp	58.76318
pryp	4	m	0.381	29.47			normoxia	1	0.895	1	Ucrit2hyp	63.09527
pryp	4	m	0.381	29.47			normoxia	1	0.895	1	Ucrit3hyp	59.73569
pryp	4	m	0.381	29.47			hypoxia	1	0.478333333	1	Ucrit1norm	64.00884
pryp	4	m	0.381	29.47			hypoxia	1	0.478333333	1	Ucrit2norm	71.99521
pryp	4	m	0.381	29.47			hypoxia	1	0.478333333	1	Ucrit3norm	69.69655
pryp	4	m	0.381	29.47			hypoxia	1	0.478333333	1	Ucrit1hyp	58.76318
pryp	4	m	0.381	29.47			hypoxia	1	0.478333333	1	Ucrit2hyp	63.09527
pryp	4	m	0.381	29.47			hypoxia	1	0.478333333	1	Ucrit3hyp	59.73569
pryp	4	m	0.381	29.47			normoxia	2	0.831666667	1	Ucrit1norm	64.00884
pryp	4	m	0.381	29.47			normoxia	2	0.831666667	1	Ucrit2norm	71.99521
pryp	4	m	0.381	29.47			normoxia	2	0.831666667	1	Ucrit3norm	69.69655
pryp	4	m	0.381	29.47			normoxia	2	0.831666667	1	Ucrit1hyp	58.76318
pryp	4	m	0.381	29.47			normoxia	2	0.831666667	1	Ucrit2hyp	63.09527
pryp	4	m	0.381	29.47			normoxia	2	0.831666667	1	Ucrit3hyp	59.73569
pryp	4	m	0.381	29.47			hypoxia	2	0.925	1	Ucrit1norm	64.00884
pryp	4	m	0.381	29.47			hypoxia	2	0.925	1	Ucrit2norm	71.99521
pryp	4	m	0.381	29.47			hypoxia	2	0.925	1	Ucrit3norm	69.69655
pryp	4	m	0.381	29.47			hypoxia	2	0.925	1	Ucrit1hyp	58.76318
pryp	4	m	0.381	29.47			hypoxia	2	0.925	1	Ucrit2hyp	63.09527
pryp	4	m	0.381	29.47			hypoxia	2	0.925	1	Ucrit3hyp	59.73569
pryr	1	f	0.593	30.58	0.816	0.252	normoxia	1	0	0	Ucrit1norm	73.05562
pryr	1	f	0.593	30.58	0.816	0.252	normoxia	1	0	0	Ucrit2norm	
pryr	1	f	0.593	30.58	0.816	0.252	normoxia	1	0	0	Ucrit3norm	
pryr	1	f	0.593	30.58	0.816	0.252	normoxia	1	0	0	Ucrit1hyp	66.3586

pryr	1	f	0.593	30.58	0.816	0.252	normoxia	1	0	0	Ucrit2hyp	
pryr	1	f	0.593	30.58	0.816	0.252	normoxia	1	0	0	Ucrit3hyp	
pryr	1	f	0.593	30.58	0.816	0.252	hypoxia	1	0	0	Ucrit1norm	73.05562
pryr	1	f	0.593	30.58	0.816	0.252	hypoxia	1	0	0	Ucrit2norm	
pryr	1	f	0.593	30.58	0.816	0.252	hypoxia	1	0	0	Ucrit3norm	
pryr	1	f	0.593	30.58	0.816	0.252	hypoxia	1	0	0	Ucrit1hyp	66.3586
pryr	1	f	0.593	30.58	0.816	0.252	hypoxia	1	0	0	Ucrit2hyp	
pryr	1	f	0.593	30.58	0.816	0.252	hypoxia	1	0	0	Ucrit3hyp	
pryr	1	f	0.593	30.58	0.816	0.252	normoxia	2	0	0	Ucrit1norm	73.05562
pryr	1	f	0.593	30.58	0.816	0.252	normoxia	2	0	0	Ucrit2norm	
pryr	1	f	0.593	30.58	0.816	0.252	normoxia	2	0	0	Ucrit3norm	
pryr	1	f	0.593	30.58	0.816	0.252	normoxia	2	0	0	Ucrit1hyp	66.3586
pryr	1	f	0.593	30.58	0.816	0.252	normoxia	2	0	0	Ucrit2hyp	
pryr	1	f	0.593	30.58	0.816	0.252	normoxia	2	0	0	Ucrit3hyp	
pryr	1	f	0.593	30.58	0.816	0.252	hypoxia	2	0	0	Ucrit1norm	73.05562
pryr	1	f	0.593	30.58	0.816	0.252	hypoxia	2	0	0	Ucrit2norm	
pryr	1	f	0.593	30.58	0.816	0.252	hypoxia	2	0	0	Ucrit3norm	
pryr	1	f	0.593	30.58	0.816	0.252	hypoxia	2	0	0	Ucrit1hyp	66.3586
pryr	1	f	0.593	30.58	0.816	0.252	hypoxia	2	0	0	Ucrit2hyp	
pryr	1	f	0.593	30.58	0.816	0.252	hypoxia	2	0	0	Ucrit3hyp	
pryy	3	f	0.712	34.32	1.274	0.199	hypoxia	1	0	0	Ucrit1norm	86.21184
pryy	3	f	0.712	34.32	1.274	0.199	hypoxia	1	0	0	Ucrit2norm	
pryy	3	f	0.712	34.32	1.274	0.199	hypoxia	1	0	0	Ucrit3norm	
pryy	3	f	0.712	34.32	1.274	0.199	hypoxia	1	0	0	Ucrit1hyp	83.26032
pryy	3	f	0.712	34.32	1.274	0.199	hypoxia	1	0	0	Ucrit2hyp	
pryy	3	f	0.712	34.32	1.274	0.199	hypoxia	1	0	0	Ucrit3hyp	
pryy	3	f	0.712	34.32	1.274	0.199	normoxia	1	0	0	Ucrit1norm	86.21184
pryy	3	f	0.712	34.32	1.274	0.199	normoxia	1	0	0	Ucrit2norm	
pryy	3	f	0.712	34.32	1.274	0.199	normoxia	1	0	0	Ucrit3norm	
pryy	3	f	0.712	34.32	1.274	0.199	normoxia	1	0	0	Ucrit1hyp	83.26032
pryy	3	f	0.712	34.32	1.274	0.199	normoxia	1	0	0	Ucrit2hyp	
pryy	3	f	0.712	34.32	1.274	0.199	normoxia	1	0	0	Ucrit3hyp	
pryy	3	f	0.712	34.32	1.274	0.199	hypoxia	2	0	0	Ucrit1norm	86.21184

pryy	3	f	0.712	34.32	1.274	0.199	hypoxia	2	0	0	Ucrit2norm	
pryy	3	f	0.712	34.32	1.274	0.199	hypoxia	2	0	0	Ucrit3norm	
pryy	3	f	0.712	34.32	1.274	0.199	hypoxia	2	0	0	Ucrit1hyp	83.26032
pryy	3	f	0.712	34.32	1.274	0.199	hypoxia	2	0	0	Ucrit2hyp	
pryy	3	f	0.712	34.32	1.274	0.199	hypoxia	2	0	0	Ucrit3hyp	
pryy	3	f	0.712	34.32	1.274	0.199	normoxia	2	0	0	Ucrit1norm	86.21184
pryy	3	f	0.712	34.32	1.274	0.199	normoxia	2	0	0	Ucrit2norm	
pryy	3	f	0.712	34.32	1.274	0.199	normoxia	2	0	0	Ucrit3norm	
pryy	3	f	0.712	34.32	1.274	0.199	normoxia	2	0	0	Ucrit1hyp	83.26032
pryy	3	f	0.712	34.32	1.274	0.199	normoxia	2	0	0	Ucrit2hyp	
pryy	3	f	0.712	34.32	1.274	0.199	normoxia	2	0	0	Ucrit3hyp	
pygp	4	f	0.891	37.31			normoxia	1	0	0	Ucrit1norm	88.01429
pygp	4	f	0.891	37.31			normoxia	1	0	0	Ucrit2norm	87.45464
pygp	4	f	0.891	37.31			normoxia	1	0	0	Ucrit3norm	91.18564
pygp	4	f	0.891	37.31			normoxia	1	0	0	Ucrit1hyp	72.45602
pygp	4	f	0.891	37.31			normoxia	1	0	0	Ucrit2hyp	73.68725
pygp	4	f	0.891	37.31			normoxia	1	0	0	Ucrit3hyp	66.26256
pygp	4	f	0.891	37.31			hypoxia	1	0	0	Ucrit1norm	88.01429
pygp	4	f	0.891	37.31			hypoxia	1	0	0	Ucrit2norm	87.45464
pygp	4	f	0.891	37.31			hypoxia	1	0	0	Ucrit3norm	91.18564
pygp	4	f	0.891	37.31			hypoxia	1	0	0	Ucrit1hyp	72.45602
pygp	4	f	0.891	37.31			hypoxia	1	0	0	Ucrit2hyp	73.68725
pygp	4	f	0.891	37.31			hypoxia	1	0	0	Ucrit3hyp	66.26256
pygp	4	f	0.891	37.31			normoxia	2	0	0	Ucrit1norm	88.01429
pygp	4	f	0.891	37.31			normoxia	2	0	0	Ucrit2norm	87.45464
pygp	4	f	0.891	37.31			normoxia	2	0	0	Ucrit3norm	91.18564
pygp	4	f	0.891	37.31			normoxia	2	0	0	Ucrit1hyp	72.45602
pygp	4	f	0.891	37.31			normoxia	2	0	0	Ucrit2hyp	73.68725
pygp	4	f	0.891	37.31			normoxia	2	0	0	Ucrit3hyp	66.26256
pygp	4	f	0.891	37.31			hypoxia	2	0	0	Ucrit1norm	88.01429
pygp	4	f	0.891	37.31			hypoxia	2	0	0	Ucrit2norm	87.45464
pygp	4	f	0.891	37.31			hypoxia	2	0	0	Ucrit3norm	91.18564
pygp	4	f	0.891	37.31			hypoxia	2	0	0	Ucrit1hyp	72.45602

pygp	4	f	0.891	37.31			hypoxia	2	0	0	Ucrit2hyp	73.68725
pygp	4	f	0.891	37.31			hypoxia	2	0	0	Ucrit3hyp	66.26256
pypp	3	f	0.472	31.68	1.185	0.253	hypoxia	1	0.716666667	1	Ucrit1norm	79.39008
pypp	3	f	0.472	31.68	1.185	0.253	hypoxia	1	0.716666667	1	Ucrit2norm	
pypp	3	f	0.472	31.68	1.185	0.253	hypoxia	1	0.716666667	1	Ucrit3norm	
pypp	3	f	0.472	31.68	1.185	0.253	hypoxia	1	0.716666667	1	Ucrit1hyp	62.28288
pypp	3	f	0.472	31.68	1.185	0.253	hypoxia	1	0.716666667	1	Ucrit2hyp	
pypp	3	f	0.472	31.68	1.185	0.253	hypoxia	1	0.716666667	1	Ucrit3hyp	
pypp	3	f	0.472	31.68	1.185	0.253	normoxia	1	0	0	Ucrit1norm	79.39008
pypp	3	f	0.472	31.68	1.185	0.253	normoxia	1	0	0	Ucrit2norm	
pypp	3	f	0.472	31.68	1.185	0.253	normoxia	1	0	0	Ucrit3norm	
pypp	3	f	0.472	31.68	1.185	0.253	normoxia	1	0	0	Ucrit1hyp	62.28288
pypp	3	f	0.472	31.68	1.185	0.253	normoxia	1	0	0	Ucrit2hyp	
pypp	3	f	0.472	31.68	1.185	0.253	normoxia	1	0	0	Ucrit3hyp	
pypp	3	f	0.472	31.68	1.185	0.253	hypoxia	2	0	0	Ucrit1norm	79.39008
pypp	3	f	0.472	31.68	1.185	0.253	hypoxia	2	0	0	Ucrit2norm	
pypp	3	f	0.472	31.68	1.185	0.253	hypoxia	2	0	0	Ucrit3norm	
pypp	3	f	0.472	31.68	1.185	0.253	hypoxia	2	0	0	Ucrit1hyp	62.28288
pypp	3	f	0.472	31.68	1.185	0.253	hypoxia	2	0	0	Ucrit2hyp	
pypp	3	f	0.472	31.68	1.185	0.253	hypoxia	2	0	0	Ucrit3hyp	
pypp	3	f	0.472	31.68	1.185	0.253	normoxia	2	0	0	Ucrit1norm	79.39008
pypp	3	f	0.472	31.68	1.185	0.253	normoxia	2	0	0	Ucrit2norm	
pypp	3	f	0.472	31.68	1.185	0.253	normoxia	2	0	0	Ucrit3norm	
pypp	3	f	0.472	31.68	1.185	0.253	normoxia	2	0	0	Ucrit1hyp	62.28288
pypp	3	f	0.472	31.68	1.185	0.253	normoxia	2	0	0	Ucrit2hyp	
pypp	3	f	0.472	31.68	1.185	0.253	normoxia	2	0	0	Ucrit3hyp	
pypr	1	f	0.807	34.75	1.836	0.267	normoxia	1	0	0	Ucrit1norm	88.8905
pypr	1	f	0.807	34.75	1.836	0.267	normoxia	1	0	0	Ucrit2norm	
pypr	1	f	0.807	34.75	1.836	0.267	normoxia	1	0	0	Ucrit3norm	
pypr	1	f	0.807	34.75	1.836	0.267	normoxia	1	0	0	Ucrit1hyp	65.3995
pypr	1	f	0.807	34.75	1.836	0.267	normoxia	1	0	0	Ucrit2hyp	
pypr	1	f	0.807	34.75	1.836	0.267	normoxia	1	0	0	Ucrit3hyp	
pypr	1	f	0.807	34.75	1.836	0.267	hypoxia	1	0	0	Ucrit1norm	88.8905

pypr	1	f	0.807	34.75	1.836	0.267	hypoxia	1	0	0	Ucrit2norm	
pypr	1	f	0.807	34.75	1.836	0.267	hypoxia	1	0	0	Ucrit3norm	
pypr	1	f	0.807	34.75	1.836	0.267	hypoxia	1	0	0	Ucrit1hyp	65.3995
pypr	1	f	0.807	34.75	1.836	0.267	hypoxia	1	0	0	Ucrit2hyp	
pypr	1	f	0.807	34.75	1.836	0.267	hypoxia	1	0	0	Ucrit3hyp	
pypr	1	f	0.807	34.75	1.836	0.267	normoxia	2	0	0	Ucrit1norm	88.8905
pypr	1	f	0.807	34.75	1.836	0.267	normoxia	2	0	0	Ucrit2norm	
pypr	1	f	0.807	34.75	1.836	0.267	normoxia	2	0	0	Ucrit3norm	
pypr	1	f	0.807	34.75	1.836	0.267	normoxia	2	0	0	Ucrit1hyp	65.3995
pypr	1	f	0.807	34.75	1.836	0.267	normoxia	2	0	0	Ucrit2hyp	
pypr	1	f	0.807	34.75	1.836	0.267	normoxia	2	0	0	Ucrit3hyp	
pypr	1	f	0.807	34.75	1.836	0.267	hypoxia	2	0	0	Ucrit1norm	88.8905
pypr	1	f	0.807	34.75	1.836	0.267	hypoxia	2	0	0	Ucrit2norm	
pypr	1	f	0.807	34.75	1.836	0.267	hypoxia	2	0	0	Ucrit3norm	
pypr	1	f	0.807	34.75	1.836	0.267	hypoxia	2	0	0	Ucrit1hyp	65.3995
pypr	1	f	0.807	34.75	1.836	0.267	hypoxia	2	0	0	Ucrit2hyp	
pypr	1	f	0.807	34.75	1.836	0.267	hypoxia	2	0	0	Ucrit3hyp	
pyrp	2	f	0.6	32.51	0.861	0.218	hypoxia	1	0.043333333	1	Ucrit1norm	75.32567
pyrp	2	f	0.6	32.51	0.861	0.218	hypoxia	1	0.043333333	1	Ucrit2norm	
pyrp	2	f	0.6	32.51	0.861	0.218	hypoxia	1	0.043333333	1	Ucrit3norm	
pyrp	2	f	0.6	32.51	0.861	0.218	hypoxia	1	0.043333333	1	Ucrit1hyp	66.58048
pyrp	2	f	0.6	32.51	0.861	0.218	hypoxia	1	0.043333333	1	Ucrit2hyp	
pyrp	2	f	0.6	32.51	0.861	0.218	hypoxia	1	0.043333333	1	Ucrit3hyp	
pyrp	2	f	0.6	32.51	0.861	0.218	normoxia	1	0	0	Ucrit1norm	75.32567
pyrp	2	f	0.6	32.51	0.861	0.218	normoxia	1	0	0	Ucrit2norm	
pyrp	2	f	0.6	32.51	0.861	0.218	normoxia	1	0	0	Ucrit3norm	
pyrp	2	f	0.6	32.51	0.861	0.218	normoxia	1	0	0	Ucrit1hyp	66.58048
pyrp	2	f	0.6	32.51	0.861	0.218	normoxia	1	0	0	Ucrit2hyp	
pyrp	2	f	0.6	32.51	0.861	0.218	normoxia	1	0	0	Ucrit3hyp	
pyrp	2	f	0.6	32.51	0.861	0.218	hypoxia	2	0.736666667	1	Ucrit1norm	75.32567
pyrp	2	f	0.6	32.51	0.861	0.218	hypoxia	2	0.736666667	1	Ucrit2norm	
pyrp	2	f	0.6	32.51	0.861	0.218	hypoxia	2	0.736666667	1	Ucrit3norm	
pyrp	2	f	0.6	32.51	0.861	0.218	hypoxia	2	0.736666667	1	Ucrit1hyp	66.58048

pyrp	2	f	0.6	32.51	0.861	0.218	hypoxia	2	0.736666667	1	Ucrit2hyp	
pyrp	2	f	0.6	32.51	0.861	0.218	hypoxia	2	0.736666667	1	Ucrit3hyp	
pyrp	2	f	0.6	32.51	0.861	0.218	normoxia	2	0	0	Ucrit1norm	75.32567
pyrp	2	f	0.6	32.51	0.861	0.218	normoxia	2	0	0	Ucrit2norm	
pyrp	2	f	0.6	32.51	0.861	0.218	normoxia	2	0	0	Ucrit3norm	
pyrp	2	f	0.6	32.51	0.861	0.218	normoxia	2	0	0	Ucrit1hyp	66.58048
pyrp	2	f	0.6	32.51	0.861	0.218	normoxia	2	0	0	Ucrit2hyp	
pyrp	2	f	0.6	32.51	0.861	0.218	normoxia	2	0	0	Ucrit3hyp	
pyry	1	m	0.44	31.47	1.011	0.168	normoxia	1	0	0	Ucrit1norm	90.53919
pyry	1	m	0.44	31.47	1.011	0.168	normoxia	1	0	0	Ucrit2norm	
pyry	1	m	0.44	31.47	1.011	0.168	normoxia	1	0	0	Ucrit3norm	
pyry	1	m	0.44	31.47	1.011	0.168	normoxia	1	0	0	Ucrit1hyp	73.26216
pyry	1	m	0.44	31.47	1.011	0.168	normoxia	1	0	0	Ucrit2hyp	
pyry	1	m	0.44	31.47	1.011	0.168	normoxia	1	0	0	Ucrit3hyp	
pyry	1	m	0.44	31.47	1.011	0.168	hypoxia	1	0	0	Ucrit1norm	90.53919
pyry	1	m	0.44	31.47	1.011	0.168	hypoxia	1	0	0	Ucrit2norm	
pyry	1	m	0.44	31.47	1.011	0.168	hypoxia	1	0	0	Ucrit3norm	
pyry	1	m	0.44	31.47	1.011	0.168	hypoxia	1	0	0	Ucrit1hyp	73.26216
pyry	1	m	0.44	31.47	1.011	0.168	hypoxia	1	0	0	Ucrit2hyp	
pyry	1	m	0.44	31.47	1.011	0.168	hypoxia	1	0	0	Ucrit3hyp	
pyry	1	m	0.44	31.47	1.011	0.168	normoxia	2	0	0	Ucrit1norm	90.53919
pyry	1	m	0.44	31.47	1.011	0.168	normoxia	2	0	0	Ucrit2norm	
pyry	1	m	0.44	31.47	1.011	0.168	normoxia	2	0	0	Ucrit3norm	
pyry	1	m	0.44	31.47	1.011	0.168	normoxia	2	0	0	Ucrit1hyp	73.26216
pyry	1	m	0.44	31.47	1.011	0.168	normoxia	2	0	0	Ucrit2hyp	
pyry	1	m	0.44	31.47	1.011	0.168	normoxia	2	0	0	Ucrit3hyp	
pyry	1	m	0.44	31.47	1.011	0.168	hypoxia	2	0	0	Ucrit1norm	90.53919
pyry	1	m	0.44	31.47	1.011	0.168	hypoxia	2	0	0	Ucrit2norm	
pyry	1	m	0.44	31.47	1.011	0.168	hypoxia	2	0	0	Ucrit3norm	
pyry	1	m	0.44	31.47	1.011	0.168	hypoxia	2	0	0	Ucrit1hyp	73.26216
pyry	1	m	0.44	31.47	1.011	0.168	hypoxia	2	0	0	Ucrit2hyp	
pyry	1	m	0.44	31.47	1.011	0.168	hypoxia	2	0	0	Ucrit3hyp	
pyyg	4	m	0.326	28.85	0.964	0.176	normoxia	1	0	0	Ucrit1norm	85.94415

pyyg	4	m	0.326	28.85	0.964	0.176	normoxia	1	0	0	Ucrit2norm	96.1282
pyyg	4	m	0.326	28.85	0.964	0.176	normoxia	1	0	0	Ucrit3norm	87.0693
pyyg	4	m	0.326	28.85	0.964	0.176	normoxia	1	0	0	Ucrit1hyp	70.4517
pyyg	4	m	0.326	28.85	0.964	0.176	normoxia	1	0	0	Ucrit2hyp	67.94175
pyyg	4	m	0.326	28.85	0.964	0.176	normoxia	1	0	0	Ucrit3hyp	73.53865
pyyg	4	m	0.326	28.85	0.964	0.176	hypoxia	1	0.765	1	Ucrit1norm	85.94415
pyyg	4	m	0.326	28.85	0.964	0.176	hypoxia	1	0.765	1	Ucrit2norm	96.1282
pyyg	4	m	0.326	28.85	0.964	0.176	hypoxia	1	0.765	1	Ucrit3norm	87.0693
pyyg	4	m	0.326	28.85	0.964	0.176	hypoxia	1	0.765	1	Ucrit1hyp	70.4517
pyyg	4	m	0.326	28.85	0.964	0.176	hypoxia	1	0.765	1	Ucrit2hyp	67.94175
pyyg	4	m	0.326	28.85	0.964	0.176	hypoxia	1	0.765	1	Ucrit3hyp	73.53865
pyyg	4	m	0.326	28.85	0.964	0.176	normoxia	2	0	0	Ucrit1norm	85.94415
pyyg	4	m	0.326	28.85	0.964	0.176	normoxia	2	0	0	Ucrit2norm	96.1282
pyyg	4	m	0.326	28.85	0.964	0.176	normoxia	2	0	0	Ucrit3norm	87.0693
pyyg	4	m	0.326	28.85	0.964	0.176	normoxia	2	0	0	Ucrit1hyp	70.4517
pyyg	4	m	0.326	28.85	0.964	0.176	normoxia	2	0	0	Ucrit2hyp	67.94175
pyyg	4	m	0.326	28.85	0.964	0.176	normoxia	2	0	0	Ucrit3hyp	73.53865
pyyg	4	m	0.326	28.85	0.964	0.176	hypoxia	2	0	0	Ucrit1norm	85.94415
pyyg	4	m	0.326	28.85	0.964	0.176	hypoxia	2	0	0	Ucrit2norm	96.1282
pyyg	4	m	0.326	28.85	0.964	0.176	hypoxia	2	0	0	Ucrit3norm	87.0693
pyyg	4	m	0.326	28.85	0.964	0.176	hypoxia	2	0	0	Ucrit1hyp	70.4517
pyyg	4	m	0.326	28.85	0.964	0.176	hypoxia	2	0	0	Ucrit2hyp	67.94175
pyyg	4	m	0.326	28.85	0.964	0.176	hypoxia	2	0	0	Ucrit3hyp	73.53865
pyyr	3	f	0.503	34.04	1.14	0.205	hypoxia	1	0.693333333	1	Ucrit1norm	72.02864
pyyr	3	f	0.503	34.04	1.14	0.205	hypoxia	1	0.693333333	1	Ucrit2norm	
pyyr	3	f	0.503	34.04	1.14	0.205	hypoxia	1	0.693333333	1	Ucrit3norm	
pyyr	3	f	0.503	34.04	1.14	0.205	hypoxia	1	0.693333333	1	Ucrit1hyp	59.53596
pyyr	3	f	0.503	34.04	1.14	0.205	hypoxia	1	0.693333333	1	Ucrit2hyp	
pyyr	3	f	0.503	34.04	1.14	0.205	hypoxia	1	0.693333333	1	Ucrit3hyp	
pyyr	3	f	0.503	34.04	1.14	0.205	normoxia	1	0	0	Ucrit1norm	72.02864
pyyr	3	f	0.503	34.04	1.14	0.205	normoxia	1	0	0	Ucrit2norm	
pyyr	3	f	0.503	34.04	1.14	0.205	normoxia	1	0	0	Ucrit3norm	
pyyr	3	f	0.503	34.04	1.14	0.205	normoxia	1	0	0	Ucrit1hyp	59.53596

pyyr	3	f	0.503	34.04	1.14	0.205	normoxia	1	0	0	Ucrit2hyp	
pyyr	3	f	0.503	34.04	1.14	0.205	normoxia	1	0	0	Ucrit3hyp	
pyyr	3	f	0.503	34.04	1.14	0.205	hypoxia	2	0	0	Ucrit1norm	72.02864
pyyr	3	f	0.503	34.04	1.14	0.205	hypoxia	2	0	0	Ucrit2norm	
pyyr	3	f	0.503	34.04	1.14	0.205	hypoxia	2	0	0	Ucrit3norm	
pyyr	3	f	0.503	34.04	1.14	0.205	hypoxia	2	0	0	Ucrit1hyp	59.53596
pyyr	3	f	0.503	34.04	1.14	0.205	hypoxia	2	0	0	Ucrit2hyp	
pyyr	3	f	0.503	34.04	1.14	0.205	hypoxia	2	0	0	Ucrit3hyp	
pyyr	3	f	0.503	34.04	1.14	0.205	normoxia	2	0	0	Ucrit1norm	72.02864
pyyr	3	f	0.503	34.04	1.14	0.205	normoxia	2	0	0	Ucrit2norm	
pyyr	3	f	0.503	34.04	1.14	0.205	normoxia	2	0	0	Ucrit3norm	
pyyr	3	f	0.503	34.04	1.14	0.205	normoxia	2	0	0	Ucrit1hyp	59.53596
pyyr	3	f	0.503	34.04	1.14	0.205	normoxia	2	0	0	Ucrit2hyp	
pyyr	3	f	0.503	34.04	1.14	0.205	normoxia	2	0	0	Ucrit3hyp	
rggg	2	m	0.443	31.59	0.829	0.065	hypoxia	1	0	0	Ucrit1norm	79.48044
rggg	2	m	0.443	31.59	0.829	0.065	hypoxia	1	0	0	Ucrit2norm	
rggg	2	m	0.443	31.59	0.829	0.065	hypoxia	1	0	0	Ucrit3norm	
rggg	2	m	0.443	31.59	0.829	0.065	hypoxia	1	0	0	Ucrit1hyp	57.84129
rggg	2	m	0.443	31.59	0.829	0.065	hypoxia	1	0	0	Ucrit2hyp	
rggg	2	m	0.443	31.59	0.829	0.065	hypoxia	1	0	0	Ucrit3hyp	
rggg	2	m	0.443	31.59	0.829	0.065	normoxia	1	0.173333333	1	Ucrit1norm	79.48044
rggg	2	m	0.443	31.59	0.829	0.065	normoxia	1	0.173333333	1	Ucrit2norm	
rggg	2	m	0.443	31.59	0.829	0.065	normoxia	1	0.173333333	1	Ucrit3norm	
rggg	2	m	0.443	31.59	0.829	0.065	normoxia	1	0.173333333	1	Ucrit1hyp	57.84129
rggg	2	m	0.443	31.59	0.829	0.065	normoxia	1	0.173333333	1	Ucrit2hyp	
rggg	2	m	0.443	31.59	0.829	0.065	normoxia	1	0.173333333	1	Ucrit3hyp	
rggg	2	m	0.443	31.59	0.829	0.065	hypoxia	2	0.928333333	1	Ucrit1norm	79.48044
rggg	2	m	0.443	31.59	0.829	0.065	hypoxia	2	0.928333333	1	Ucrit2norm	
rggg	2	m	0.443	31.59	0.829	0.065	hypoxia	2	0.928333333	1	Ucrit3norm	
rggg	2	m	0.443	31.59	0.829	0.065	hypoxia	2	0.928333333	1	Ucrit1hyp	57.84129
rggg	2	m	0.443	31.59	0.829	0.065	hypoxia	2	0.928333333	1	Ucrit2hyp	
rggg	2	m	0.443	31.59	0.829	0.065	hypoxia	2	0.928333333	1	Ucrit3hyp	
rggg	2	m	0.443	31.59	0.829	0.065	normoxia	2	0.908333333	1	Ucrit1norm	79.48044

rggg	2	m	0.443	31.59	0.829	0.065	normoxia	2	0.908333333	1	Ucrit2norm	
rggg	2	m	0.443	31.59	0.829	0.065	normoxia	2	0.908333333	1	Ucrit3norm	
rggg	2	m	0.443	31.59	0.829	0.065	normoxia	2	0.908333333	1	Ucrit1hyp	57.84129
rggg	2	m	0.443	31.59	0.829	0.065	normoxia	2	0.908333333	1	Ucrit2hyp	
rggg	2	m	0.443	31.59	0.829	0.065	normoxia	2	0.908333333	1	Ucrit3hyp	
rggp	2	f	0.347	27.49	0.795	0.154	hypoxia	1	0	0	Ucrit1norm	78.37399
rggp	2	f	0.347	27.49	0.795	0.154	hypoxia	1	0	0	Ucrit2norm	
rggp	2	f	0.347	27.49	0.795	0.154	hypoxia	1	0	0	Ucrit3norm	
rggp	2	f	0.347	27.49	0.795	0.154	hypoxia	1	0	0	Ucrit1hyp	72.68356
rggp	2	f	0.347	27.49	0.795	0.154	hypoxia	1	0	0	Ucrit2hyp	
rggp	2	f	0.347	27.49	0.795	0.154	hypoxia	1	0	0	Ucrit3hyp	
rggp	2	f	0.347	27.49	0.795	0.154	normoxia	1	0	0	Ucrit1norm	78.37399
rggp	2	f	0.347	27.49	0.795	0.154	normoxia	1	0	0	Ucrit2norm	
rggp	2	f	0.347	27.49	0.795	0.154	normoxia	1	0	0	Ucrit3norm	
rggp	2	f	0.347	27.49	0.795	0.154	normoxia	1	0	0	Ucrit1hyp	72.68356
rggp	2	f	0.347	27.49	0.795	0.154	normoxia	1	0	0	Ucrit2hyp	
rggp	2	f	0.347	27.49	0.795	0.154	normoxia	1	0	0	Ucrit3hyp	
rggp	2	f	0.347	27.49	0.795	0.154	hypoxia	2	0	0	Ucrit1norm	78.37399
rggp	2	f	0.347	27.49	0.795	0.154	hypoxia	2	0	0	Ucrit2norm	
rggp	2	f	0.347	27.49	0.795	0.154	hypoxia	2	0	0	Ucrit3norm	
rggp	2	f	0.347	27.49	0.795	0.154	hypoxia	2	0	0	Ucrit1hyp	72.68356
rggp	2	f	0.347	27.49	0.795	0.154	hypoxia	2	0	0	Ucrit2hyp	
rggp	2	f	0.347	27.49	0.795	0.154	hypoxia	2	0	0	Ucrit3hyp	
rggp	2	f	0.347	27.49	0.795	0.154	normoxia	2	0	0	Ucrit1norm	78.37399
rggp	2	f	0.347	27.49	0.795	0.154	normoxia	2	0	0	Ucrit2norm	
rggp	2	f	0.347	27.49	0.795	0.154	normoxia	2	0	0	Ucrit3norm	
rggp	2	f	0.347	27.49	0.795	0.154	normoxia	2	0	0	Ucrit1hyp	72.68356
rggp	2	f	0.347	27.49	0.795	0.154	normoxia	2	0	0	Ucrit2hyp	
rggp	2	f	0.347	27.49	0.795	0.154	normoxia	2	0	0	Ucrit3hyp	
rggr	1	m	0.361	30.45	1.172	0.132	normoxia	1	0	0	Ucrit1norm	86.7825
rggr	1	m	0.361	30.45	1.172	0.132	normoxia	1	0	0	Ucrit2norm	
rggr	1	m	0.361	30.45	1.172	0.132	normoxia	1	0	0	Ucrit3norm	
rggr	1	m	0.361	30.45	1.172	0.132	normoxia	1	0	0	Ucrit1hyp	92.7507

rggr	1	m	0.361	30.45	1.172	0.132	normoxia	1	0	0	Ucrit2hyp	
rggr	1	m	0.361	30.45	1.172	0.132	normoxia	1	0	0	Ucrit3hyp	
rggr	1	m	0.361	30.45	1.172	0.132	hypoxia	1	0	0	Ucrit1norm	86.7825
rggr	1	m	0.361	30.45	1.172	0.132	hypoxia	1	0	0	Ucrit2norm	
rggr	1	m	0.361	30.45	1.172	0.132	hypoxia	1	0	0	Ucrit3norm	
rggr	1	m	0.361	30.45	1.172	0.132	hypoxia	1	0	0	Ucrit1hyp	92.7507
rggr	1	m	0.361	30.45	1.172	0.132	hypoxia	1	0	0	Ucrit2hyp	
rggr	1	m	0.361	30.45	1.172	0.132	hypoxia	1	0	0	Ucrit3hyp	
rggr	1	m	0.361	30.45	1.172	0.132	normoxia	2	0	0	Ucrit1norm	86.7825
rggr	1	m	0.361	30.45	1.172	0.132	normoxia	2	0	0	Ucrit2norm	
rggr	1	m	0.361	30.45	1.172	0.132	normoxia	2	0	0	Ucrit3norm	
rggr	1	m	0.361	30.45	1.172	0.132	normoxia	2	0	0	Ucrit1hyp	92.7507
rggr	1	m	0.361	30.45	1.172	0.132	normoxia	2	0	0	Ucrit2hyp	
rggr	1	m	0.361	30.45	1.172	0.132	normoxia	2	0	0	Ucrit3hyp	
rggr	1	m	0.361	30.45	1.172	0.132	hypoxia	2	0	0	Ucrit1norm	86.7825
rggr	1	m	0.361	30.45	1.172	0.132	hypoxia	2	0	0	Ucrit2norm	
rggr	1	m	0.361	30.45	1.172	0.132	hypoxia	2	0	0	Ucrit3norm	
rggr	1	m	0.361	30.45	1.172	0.132	hypoxia	2	0	0	Ucrit1hyp	92.7507
rggr	1	m	0.361	30.45	1.172	0.132	hypoxia	2	0	0	Ucrit2hyp	
rggr	1	m	0.361	30.45	1.172	0.132	hypoxia	2	0	0	Ucrit3hyp	
rggy	2	m	0.438	30.89	0.869	0.091	hypoxia	1	0	0	Ucrit1norm	91.58885
rggy	2	m	0.438	30.89	0.869	0.091	hypoxia	1	0	0	Ucrit2norm	
rggy	2	m	0.438	30.89	0.869	0.091	hypoxia	1	0	0	Ucrit3norm	
rggy	2	m	0.438	30.89	0.869	0.091	hypoxia	1	0	0	Ucrit1hyp	71.23234
rggy	2	m	0.438	30.89	0.869	0.091	hypoxia	1	0	0	Ucrit2hyp	
rggy	2	m	0.438	30.89	0.869	0.091	hypoxia	1	0	0	Ucrit3hyp	
rggy	2	m	0.438	30.89	0.869	0.091	normoxia	1	0	0	Ucrit1norm	91.58885
rggy	2	m	0.438	30.89	0.869	0.091	normoxia	1	0	0	Ucrit2norm	
rggy	2	m	0.438	30.89	0.869	0.091	normoxia	1	0	0	Ucrit3norm	
rggy	2	m	0.438	30.89	0.869	0.091	normoxia	1	0	0	Ucrit1hyp	71.23234
rggy	2	m	0.438	30.89	0.869	0.091	normoxia	1	0	0	Ucrit2hyp	
rggy	2	m	0.438	30.89	0.869	0.091	normoxia	1	0	0	Ucrit3hyp	
rggy	2	m	0.438	30.89	0.869	0.091	hypoxia	2	0	0	Ucrit1norm	91.58885

rggy	2	m	0.438	30.89	0.869	0.091	hypoxia	2	0	0	Ucrit2norm	
rggy	2	m	0.438	30.89	0.869	0.091	hypoxia	2	0	0	Ucrit3norm	
rggy	2	m	0.438	30.89	0.869	0.091	hypoxia	2	0	0	Ucrit1hyp	71.23234
rggy	2	m	0.438	30.89	0.869	0.091	hypoxia	2	0	0	Ucrit2hyp	
rggy	2	m	0.438	30.89	0.869	0.091	hypoxia	2	0	0	Ucrit3hyp	
rggy	2	m	0.438	30.89	0.869	0.091	normoxia	2	0	0	Ucrit1norm	91.58885
rggy	2	m	0.438	30.89	0.869	0.091	normoxia	2	0	0	Ucrit2norm	
rggy	2	m	0.438	30.89	0.869	0.091	normoxia	2	0	0	Ucrit3norm	
rggy	2	m	0.438	30.89	0.869	0.091	normoxia	2	0	0	Ucrit1hyp	71.23234
rggy	2	m	0.438	30.89	0.869	0.091	normoxia	2	0	0	Ucrit2hyp	
rggy	2	m	0.438	30.89	0.869	0.091	normoxia	2	0	0	Ucrit3hyp	
rgpr	1	m	0.371	29.98	0.782	0.132	normoxia	1	0	0	Ucrit1norm	86.07258
rgpr	1	m	0.371	29.98	0.782	0.132	normoxia	1	0	0	Ucrit2norm	
rgpr	1	m	0.371	29.98	0.782	0.132	normoxia	1	0	0	Ucrit3norm	
rgpr	1	m	0.371	29.98	0.782	0.132	normoxia	1	0	0	Ucrit1hyp	76.77878
rgpr	1	m	0.371	29.98	0.782	0.132	normoxia	1	0	0	Ucrit2hyp	
rgpr	1	m	0.371	29.98	0.782	0.132	normoxia	1	0	0	Ucrit3hyp	
rgpr	1	m	0.371	29.98	0.782	0.132	hypoxia	1	0	0	Ucrit1norm	86.07258
rgpr	1	m	0.371	29.98	0.782	0.132	hypoxia	1	0	0	Ucrit2norm	
rgpr	1	m	0.371	29.98	0.782	0.132	hypoxia	1	0	0	Ucrit3norm	
rgpr	1	m	0.371	29.98	0.782	0.132	hypoxia	1	0	0	Ucrit1hyp	76.77878
rgpr	1	m	0.371	29.98	0.782	0.132	hypoxia	1	0	0	Ucrit2hyp	
rgpr	1	m	0.371	29.98	0.782	0.132	hypoxia	1	0	0	Ucrit3hyp	
rgpr	1	m	0.371	29.98	0.782	0.132	normoxia	2	0	0	Ucrit1norm	86.07258
rgpr	1	m	0.371	29.98	0.782	0.132	normoxia	2	0	0	Ucrit2norm	
rgpr	1	m	0.371	29.98	0.782	0.132	normoxia	2	0	0	Ucrit3norm	
rgpr	1	m	0.371	29.98	0.782	0.132	normoxia	2	0	0	Ucrit1hyp	76.77878
rgpr	1	m	0.371	29.98	0.782	0.132	normoxia	2	0	0	Ucrit2hyp	
rgpr	1	m	0.371	29.98	0.782	0.132	normoxia	2	0	0	Ucrit3hyp	
rgpr	1	m	0.371	29.98	0.782	0.132	hypoxia	2	0.82	1	Ucrit1norm	86.07258
rgpr	1	m	0.371	29.98	0.782	0.132	hypoxia	2	0.82	1	Ucrit2norm	
rgpr	1	m	0.371	29.98	0.782	0.132	hypoxia	2	0.82	1	Ucrit3norm	
rgpr	1	m	0.371	29.98	0.782	0.132	hypoxia	2	0.82	1	Ucrit1hyp	76.77878

rgpr	1	m	0.371	29.98	0.782	0.132	hypoxia	2	0.82	1	Ucrit2hyp	
rgpr	1	m	0.371	29.98	0.782	0.132	hypoxia	2	0.82	1	Ucrit3hyp	
rgrp	2	f	0.6	34.39	0.96	0.215	hypoxia	1	0.436666667	1	Ucrit1norm	82.63917
rgrp	2	f	0.6	34.39	0.96	0.215	hypoxia	1	0.436666667	1	Ucrit2norm	
rgrp	2	f	0.6	34.39	0.96	0.215	hypoxia	1	0.436666667	1	Ucrit3norm	
rgrp	2	f	0.6	34.39	0.96	0.215	hypoxia	1	0.436666667	1	Ucrit1hyp	59.63226
rgrp	2	f	0.6	34.39	0.96	0.215	hypoxia	1	0.436666667	1	Ucrit2hyp	
rgrp	2	f	0.6	34.39	0.96	0.215	hypoxia	1	0.436666667	1	Ucrit3hyp	
rgrp	2	f	0.6	34.39	0.96	0.215	normoxia	1	0	0	Ucrit1norm	82.63917
rgrp	2	f	0.6	34.39	0.96	0.215	normoxia	1	0	0	Ucrit2norm	
rgrp	2	f	0.6	34.39	0.96	0.215	normoxia	1	0	0	Ucrit3norm	
rgrp	2	f	0.6	34.39	0.96	0.215	normoxia	1	0	0	Ucrit1hyp	59.63226
rgrp	2	f	0.6	34.39	0.96	0.215	normoxia	1	0	0	Ucrit2hyp	
rgrp	2	f	0.6	34.39	0.96	0.215	normoxia	1	0	0	Ucrit3hyp	
rgrp	2	f	0.6	34.39	0.96	0.215	hypoxia	2	0	0	Ucrit1norm	82.63917
rgrp	2	f	0.6	34.39	0.96	0.215	hypoxia	2	0	0	Ucrit2norm	
rgrp	2	f	0.6	34.39	0.96	0.215	hypoxia	2	0	0	Ucrit3norm	
rgrp	2	f	0.6	34.39	0.96	0.215	hypoxia	2	0	0	Ucrit1hyp	59.63226
rgrp	2	f	0.6	34.39	0.96	0.215	hypoxia	2	0	0	Ucrit2hyp	
rgrp	2	f	0.6	34.39	0.96	0.215	hypoxia	2	0	0	Ucrit3hyp	
rgrp	2	f	0.6	34.39	0.96	0.215	normoxia	2	0	0	Ucrit1norm	82.63917
rgrp	2	f	0.6	34.39	0.96	0.215	normoxia	2	0	0	Ucrit2norm	
rgrp	2	f	0.6	34.39	0.96	0.215	normoxia	2	0	0	Ucrit3norm	
rgrp	2	f	0.6	34.39	0.96	0.215	normoxia	2	0	0	Ucrit1hyp	59.63226
rgrp	2	f	0.6	34.39	0.96	0.215	normoxia	2	0	0	Ucrit2hyp	
rgrp	2	f	0.6	34.39	0.96	0.215	normoxia	2	0	0	Ucrit3hyp	
rgry	2	m	0.545	34.01	1.066	0.21	hypoxia	1	0	0	Ucrit1norm	74.07378
rgry	2	m	0.545	34.01	1.066	0.21	hypoxia	1	0	0	Ucrit2norm	
rgry	2	m	0.545	34.01	1.066	0.21	hypoxia	1	0	0	Ucrit3norm	
rgry	2	m	0.545	34.01	1.066	0.21	hypoxia	1	0	0	Ucrit1hyp	58.02106
rgry	2	m	0.545	34.01	1.066	0.21	hypoxia	1	0	0	Ucrit2hyp	
rgry	2	m	0.545	34.01	1.066	0.21	hypoxia	1	0	0	Ucrit3hyp	
rgry	2	m	0.545	34.01	1.066	0.21	normoxia	1	0	0	Ucrit1norm	74.07378

rgry	2	m	0.545	34.01	1.066	0.21	normoxia	1	0	0	Ucrit2norm	
rgry	2	m	0.545	34.01	1.066	0.21	normoxia	1	0	0	Ucrit3norm	
rgry	2	m	0.545	34.01	1.066	0.21	normoxia	1	0	0	Ucrit1hyp	58.02106
rgry	2	m	0.545	34.01	1.066	0.21	normoxia	1	0	0	Ucrit2hyp	
rgry	2	m	0.545	34.01	1.066	0.21	normoxia	1	0	0	Ucrit3hyp	
rgry	2	m	0.545	34.01	1.066	0.21	hypoxia	2	0.031666667	1	Ucrit1norm	74.07378
rgry	2	m	0.545	34.01	1.066	0.21	hypoxia	2	0.031666667	1	Ucrit2norm	
rgry	2	m	0.545	34.01	1.066	0.21	hypoxia	2	0.031666667	1	Ucrit3norm	
rgry	2	m	0.545	34.01	1.066	0.21	hypoxia	2	0.031666667	1	Ucrit1hyp	58.02106
rgry	2	m	0.545	34.01	1.066	0.21	hypoxia	2	0.031666667	1	Ucrit2hyp	
rgry	2	m	0.545	34.01	1.066	0.21	hypoxia	2	0.031666667	1	Ucrit3hyp	
rgry	2	m	0.545	34.01	1.066	0.21	normoxia	2	0.88	1	Ucrit1norm	74.07378
rgry	2	m	0.545	34.01	1.066	0.21	normoxia	2	0.88	1	Ucrit2norm	
rgry	2	m	0.545	34.01	1.066	0.21	normoxia	2	0.88	1	Ucrit3norm	
rgry	2	m	0.545	34.01	1.066	0.21	normoxia	2	0.88	1	Ucrit1hyp	58.02106
rgry	2	m	0.545	34.01	1.066	0.21	normoxia	2	0.88	1	Ucrit2hyp	
rgry	2	m	0.545	34.01	1.066	0.21	normoxia	2	0.88	1	Ucrit3hyp	
rgyp	1	m	0.481	31.3	1.021	0.154	normoxia	1	0	0	Ucrit1norm	85.0734
rgyp	1	m	0.481	31.3	1.021	0.154	normoxia	1	0	0	Ucrit2norm	
rgyp	1	m	0.481	31.3	1.021	0.154	normoxia	1	0	0	Ucrit3norm	
rgyp	1	m	0.481	31.3	1.021	0.154	normoxia	1	0	0	Ucrit1hyp	71.9587
rgyp	1	m	0.481	31.3	1.021	0.154	normoxia	1	0	0	Ucrit2hyp	
rgyp	1	m	0.481	31.3	1.021	0.154	normoxia	1	0	0	Ucrit3hyp	
rgyp	1	m	0.481	31.3	1.021	0.154	hypoxia	1	0	0	Ucrit1norm	85.0734
rgyp	1	m	0.481	31.3	1.021	0.154	hypoxia	1	0	0	Ucrit2norm	
rgyp	1	m	0.481	31.3	1.021	0.154	hypoxia	1	0	0	Ucrit3norm	
rgyp	1	m	0.481	31.3	1.021	0.154	hypoxia	1	0	0	Ucrit1hyp	71.9587
rgyp	1	m	0.481	31.3	1.021	0.154	hypoxia	1	0	0	Ucrit2hyp	
rgyp	1	m	0.481	31.3	1.021	0.154	hypoxia	1	0	0	Ucrit3hyp	
rgyp	1	m	0.481	31.3	1.021	0.154	normoxia	2	0	0	Ucrit1norm	85.0734
rgyp	1	m	0.481	31.3	1.021	0.154	normoxia	2	0	0	Ucrit2norm	
rgyp	1	m	0.481	31.3	1.021	0.154	normoxia	2	0	0	Ucrit3norm	
rgyp	1	m	0.481	31.3	1.021	0.154	normoxia	2	0	0	Ucrit1hyp	71.9587

rgyp	1	m	0.481	31.3	1.021	0.154	normoxia	2	0	0	Ucrit2hyp	
rgyp	1	m	0.481	31.3	1.021	0.154	normoxia	2	0	0	Ucrit3hyp	
rgyp	1	m	0.481	31.3	1.021	0.154	hypoxia	2	0	0	Ucrit1norm	85.0734
rgyp	1	m	0.481	31.3	1.021	0.154	hypoxia	2	0	0	Ucrit2norm	
rgyp	1	m	0.481	31.3	1.021	0.154	hypoxia	2	0	0	Ucrit3norm	
rgyp	1	m	0.481	31.3	1.021	0.154	hypoxia	2	0	0	Ucrit1hyp	71.9587
rgyp	1	m	0.481	31.3	1.021	0.154	hypoxia	2	0	0	Ucrit2hyp	
rgyp	1	m	0.481	31.3	1.021	0.154	hypoxia	2	0	0	Ucrit3hyp	
rgyr	3	f	0.552	32.53			hypoxia	1	0	0	Ucrit1norm	85.61896
rgyr	3	f	0.552	32.53			hypoxia	1	0	0	Ucrit2norm	
rgyr	3	f	0.552	32.53			hypoxia	1	0	0	Ucrit3norm	
rgyr	3	f	0.552	32.53			hypoxia	1	0	0	Ucrit1hyp	
rgyr	3	f	0.552	32.53			hypoxia	1	0	0	Ucrit2hyp	
rgyr	3	f	0.552	32.53			hypoxia	1	0	0	Ucrit3hyp	
rgyr	3	f	0.552	32.53			normoxia	1	0	0	Ucrit1norm	85.61896
rgyr	3	f	0.552	32.53			normoxia	1	0	0	Ucrit2norm	
rgyr	3	f	0.552	32.53			normoxia	1	0	0	Ucrit3norm	
rgyr	3	f	0.552	32.53			normoxia	1	0	0	Ucrit1hyp	
rgyr	3	f	0.552	32.53			normoxia	1	0	0	Ucrit2hyp	
rgyr	3	f	0.552	32.53			normoxia	1	0	0	Ucrit3hyp	
rgyr	3	f	0.552	32.53			hypoxia	2	0	0	Ucrit1norm	85.61896
rgyr	3	f	0.552	32.53			hypoxia	2	0	0	Ucrit2norm	
rgyr	3	f	0.552	32.53			hypoxia	2	0	0	Ucrit3norm	
rgyr	3	f	0.552	32.53			hypoxia	2	0	0	Ucrit1hyp	
rgyr	3	f	0.552	32.53			hypoxia	2	0	0	Ucrit2hyp	
rgyr	3	f	0.552	32.53			hypoxia	2	0	0	Ucrit3hyp	
rgyr	3	f	0.552	32.53			normoxia	2	0	0	Ucrit1norm	85.61896
rgyr	3	f	0.552	32.53			normoxia	2	0	0	Ucrit2norm	
rgyr	3	f	0.552	32.53			normoxia	2	0	0	Ucrit3norm	
rgyr	3	f	0.552	32.53			normoxia	2	0	0	Ucrit1hyp	
rgyr	3	f	0.552	32.53			normoxia	2	0	0	Ucrit2hyp	
rgyr	3	f	0.552	32.53			normoxia	2	0	0	Ucrit3hyp	
rpgp	3	m	0.443	32.06	0.99	0.165	hypoxia	1	0	0	Ucrit1norm	82.58656

rpgp	3	m	0.443	32.06	0.99	0.165	hypoxia	1	0	0	Ucrit2norm	
rpgp	3	m	0.443	32.06	0.99	0.165	hypoxia	1	0	0	Ucrit3norm	
rpgp	3	m	0.443	32.06	0.99	0.165	hypoxia	1	0	0	Ucrit1hyp	71.6541
rpgp	3	m	0.443	32.06	0.99	0.165	hypoxia	1	0	0	Ucrit2hyp	
rpgp	3	m	0.443	32.06	0.99	0.165	hypoxia	1	0	0	Ucrit3hyp	
rpgp	3	m	0.443	32.06	0.99	0.165	normoxia	1	0	0	Ucrit1norm	82.58656
rpgp	3	m	0.443	32.06	0.99	0.165	normoxia	1	0	0	Ucrit2norm	
rpgp	3	m	0.443	32.06	0.99	0.165	normoxia	1	0	0	Ucrit3norm	
rpgp	3	m	0.443	32.06	0.99	0.165	normoxia	1	0	0	Ucrit1hyp	71.6541
rpgp	3	m	0.443	32.06	0.99	0.165	normoxia	1	0	0	Ucrit2hyp	
rpgp	3	m	0.443	32.06	0.99	0.165	normoxia	1	0	0	Ucrit3hyp	
rpgp	3	m	0.443	32.06	0.99	0.165	hypoxia	2	0.583333333	1	Ucrit1norm	82.58656
rpgp	3	m	0.443	32.06	0.99	0.165	hypoxia	2	0.583333333	1	Ucrit2norm	
rpgp	3	m	0.443	32.06	0.99	0.165	hypoxia	2	0.583333333	1	Ucrit3norm	
rpgp	3	m	0.443	32.06	0.99	0.165	hypoxia	2	0.583333333	1	Ucrit1hyp	71.6541
rpgp	3	m	0.443	32.06	0.99	0.165	hypoxia	2	0.583333333	1	Ucrit2hyp	
rpgp	3	m	0.443	32.06	0.99	0.165	hypoxia	2	0.583333333	1	Ucrit3hyp	
rpgp	3	m	0.443	32.06	0.99	0.165	normoxia	2	0	0	Ucrit1norm	82.58656
rpgp	3	m	0.443	32.06	0.99	0.165	normoxia	2	0	0	Ucrit2norm	
rpgp	3	m	0.443	32.06	0.99	0.165	normoxia	2	0	0	Ucrit3norm	
rpgp	3	m	0.443	32.06	0.99	0.165	normoxia	2	0	0	Ucrit1hyp	71.6541
rpgp	3	m	0.443	32.06	0.99	0.165	normoxia	2	0	0	Ucrit2hyp	
rpgp	3	m	0.443	32.06	0.99	0.165	normoxia	2	0	0	Ucrit3hyp	
rppg	2	f	0.651	33.9			hypoxia	1	0	0	Ucrit1norm	
rppg	2	f	0.651	33.9			hypoxia	1	0	0	Ucrit2norm	
rppg	2	f	0.651	33.9			hypoxia	1	0	0	Ucrit3norm	
rppg	2	f	0.651	33.9			hypoxia	1	0	0	Ucrit1hyp	
rppg	2	f	0.651	33.9			hypoxia	1	0	0	Ucrit2hyp	
rppg	2	f	0.651	33.9			hypoxia	1	0	0	Ucrit3hyp	
rppr	4	m	0.491	31.7			normoxia	1	0	0	Ucrit1norm	79.8523
rppr	4	m	0.491	31.7			normoxia	1	0	0	Ucrit2norm	82.7687
rppr	4	m	0.491	31.7			normoxia	1	0	0	Ucrit3norm	79.2183
rppr	4	m	0.491	31.7			normoxia	1	0	0	Ucrit1hyp	63.2732

rppr	4	m	0.491	31.7			normoxia	1	0	0	Ucrit2hyp	65.4288
rppr	4	m	0.491	31.7			normoxia	1	0	0	Ucrit3hyp	67.0455
rppr	4	m	0.491	31.7			hypoxia	1	0	0	Ucrit1norm	79.8523
rppr	4	m	0.491	31.7			hypoxia	1	0	0	Ucrit2norm	82.7687
rppr	4	m	0.491	31.7			hypoxia	1	0	0	Ucrit3norm	79.2183
rppr	4	m	0.491	31.7			hypoxia	1	0	0	Ucrit1hyp	63.2732
rppr	4	m	0.491	31.7			hypoxia	1	0	0	Ucrit2hyp	65.4288
rppr	4	m	0.491	31.7			hypoxia	1	0	0	Ucrit3hyp	67.0455
rppr	4	m	0.491	31.7			normoxia	2	0	0	Ucrit1norm	79.8523
rppr	4	m	0.491	31.7			normoxia	2	0	0	Ucrit2norm	82.7687
rppr	4	m	0.491	31.7			normoxia	2	0	0	Ucrit3norm	79.2183
rppr	4	m	0.491	31.7			normoxia	2	0	0	Ucrit1hyp	63.2732
rppr	4	m	0.491	31.7			normoxia	2	0	0	Ucrit2hyp	65.4288
rppr	4	m	0.491	31.7			normoxia	2	0	0	Ucrit3hyp	67.0455
rppr	4	m	0.491	31.7			hypoxia	2	0	0	Ucrit1norm	79.8523
rppr	4	m	0.491	31.7			hypoxia	2	0	0	Ucrit2norm	82.7687
rppr	4	m	0.491	31.7			hypoxia	2	0	0	Ucrit3norm	79.2183
rppr	4	m	0.491	31.7			hypoxia	2	0	0	Ucrit1hyp	63.2732
rppr	4	m	0.491	31.7			hypoxia	2	0	0	Ucrit2hyp	65.4288
rppr	4	m	0.491	31.7			hypoxia	2	0	0	Ucrit3hyp	67.0455
rppy	1	f	0.574	31.59	0.757	0.244	normoxia	1	0	0	Ucrit1norm	74.42604
rppy	1	f	0.574	31.59	0.757	0.244	normoxia	1	0	0	Ucrit2norm	
rppy	1	f	0.574	31.59	0.757	0.244	normoxia	1	0	0	Ucrit3norm	
rppy	1	f	0.574	31.59	0.757	0.244	normoxia	1	0	0	Ucrit1hyp	58.78899
rppy	1	f	0.574	31.59	0.757	0.244	normoxia	1	0	0	Ucrit2hyp	
rppy	1	f	0.574	31.59	0.757	0.244	normoxia	1	0	0	Ucrit3hyp	
rppy	1	f	0.574	31.59	0.757	0.244	hypoxia	1	0	0	Ucrit1norm	74.42604
rppy	1	f	0.574	31.59	0.757	0.244	hypoxia	1	0	0	Ucrit2norm	
rppy	1	f	0.574	31.59	0.757	0.244	hypoxia	1	0	0	Ucrit3norm	
rppy	1	f	0.574	31.59	0.757	0.244	hypoxia	1	0	0	Ucrit1hyp	58.78899
rppy	1	f	0.574	31.59	0.757	0.244	hypoxia	1	0	0	Ucrit2hyp	
rppy	1	f	0.574	31.59	0.757	0.244	hypoxia	1	0	0	Ucrit3hyp	
rppy	1	f	0.574	31.59	0.757	0.244	normoxia	2	0	0	Ucrit1norm	74.42604

rppy	1	f	0.574	31.59	0.757	0.244	normoxia	2	0	0	Ucrit2norm	
rppy	1	f	0.574	31.59	0.757	0.244	normoxia	2	0	0	Ucrit3norm	
rppy	1	f	0.574	31.59	0.757	0.244	normoxia	2	0	0	Ucrit1hyp	58.78899
rppy	1	f	0.574	31.59	0.757	0.244	normoxia	2	0	0	Ucrit2hyp	
rppy	1	f	0.574	31.59	0.757	0.244	normoxia	2	0	0	Ucrit3hyp	
rppy	1	f	0.574	31.59	0.757	0.244	hypoxia	2	0	0	Ucrit1norm	74.42604
rppy	1	f	0.574	31.59	0.757	0.244	hypoxia	2	0	0	Ucrit2norm	
rppy	1	f	0.574	31.59	0.757	0.244	hypoxia	2	0	0	Ucrit3norm	
rppy	1	f	0.574	31.59	0.757	0.244	hypoxia	2	0	0	Ucrit1hyp	58.78899
rppy	1	f	0.574	31.59	0.757	0.244	hypoxia	2	0	0	Ucrit2hyp	
rppy	1	f	0.574	31.59	0.757	0.244	hypoxia	2	0	0	Ucrit3hyp	
rpry	3	m	0.518	33.52	1.355	0.134	hypoxia	1	0.445	1	Ucrit1norm	89.09616
rpry	3	m	0.518	33.52	1.355	0.134	hypoxia	1	0.445	1	Ucrit2norm	
rpry	3	m	0.518	33.52	1.355	0.134	hypoxia	1	0.445	1	Ucrit3norm	
rpry	3	m	0.518	33.52	1.355	0.134	hypoxia	1	0.445	1	Ucrit1hyp	75.38648
rpry	3	m	0.518	33.52	1.355	0.134	hypoxia	1	0.445	1	Ucrit2hyp	
rpry	3	m	0.518	33.52	1.355	0.134	hypoxia	1	0.445	1	Ucrit3hyp	
rpry	3	m	0.518	33.52	1.355	0.134	normoxia	1	0	0	Ucrit1norm	89.09616
rpry	3	m	0.518	33.52	1.355	0.134	normoxia	1	0	0	Ucrit2norm	
rpry	3	m	0.518	33.52	1.355	0.134	normoxia	1	0	0	Ucrit3norm	
rpry	3	m	0.518	33.52	1.355	0.134	normoxia	1	0	0	Ucrit1hyp	75.38648
rpry	3	m	0.518	33.52	1.355	0.134	normoxia	1	0	0	Ucrit2hyp	
rpry	3	m	0.518	33.52	1.355	0.134	normoxia	1	0	0	Ucrit3hyp	
rpry	3	m	0.518	33.52	1.355	0.134	hypoxia	2	0	0	Ucrit1norm	89.09616
rpry	3	m	0.518	33.52	1.355	0.134	hypoxia	2	0	0	Ucrit2norm	
rpry	3	m	0.518	33.52	1.355	0.134	hypoxia	2	0	0	Ucrit3norm	
rpry	3	m	0.518	33.52	1.355	0.134	hypoxia	2	0	0	Ucrit1hyp	75.38648
rpry	3	m	0.518	33.52	1.355	0.134	hypoxia	2	0	0	Ucrit2hyp	
rpry	3	m	0.518	33.52	1.355	0.134	hypoxia	2	0	0	Ucrit3hyp	
rpry	3	m	0.518	33.52	1.355	0.134	normoxia	2	0	0	Ucrit1norm	89.09616
rpry	3	m	0.518	33.52	1.355	0.134	normoxia	2	0	0	Ucrit2norm	
rpry	3	m	0.518	33.52	1.355	0.134	normoxia	2	0	0	Ucrit3norm	
rpry	3	m	0.518	33.52	1.355	0.134	normoxia	2	0	0	Ucrit1hyp	75.38648

rpry	3	m	0.518	33.52	1.355	0.134	normoxia	2	0	0	Ucrit2hyp	
rpry	3	m	0.518	33.52	1.355	0.134	normoxia	2	0	0	Ucrit3hyp	
rpyg	2	f	0.52	28.1	1.454	0.269	hypoxia	1	0.786666667	1	Ucrit1norm	66.4565
rpyg	2	f	0.52	28.1	1.454	0.269	hypoxia	1	0.786666667	1	Ucrit2norm	
rpyg	2	f	0.52	28.1	1.454	0.269	hypoxia	1	0.786666667	1	Ucrit3norm	
rpyg	2	f	0.52	28.1	1.454	0.269	hypoxia	1	0.786666667	1	Ucrit1hyp	53.4462
rpyg	2	f	0.52	28.1	1.454	0.269	hypoxia	1	0.786666667	1	Ucrit2hyp	
rpyg	2	f	0.52	28.1	1.454	0.269	hypoxia	1	0.786666667	1	Ucrit3hyp	
rpyg	2	f	0.52	28.1	1.454	0.269	normoxia	1	0.765	1	Ucrit1norm	66.4565
rpyg	2	f	0.52	28.1	1.454	0.269	normoxia	1	0.765	1	Ucrit2norm	
rpyg	2	f	0.52	28.1	1.454	0.269	normoxia	1	0.765	1	Ucrit3norm	
rpyg	2	f	0.52	28.1	1.454	0.269	normoxia	1	0.765	1	Ucrit1hyp	53.4462
rpyg	2	f	0.52	28.1	1.454	0.269	normoxia	1	0.765	1	Ucrit2hyp	
rpyg	2	f	0.52	28.1	1.454	0.269	normoxia	1	0.765	1	Ucrit3hyp	
rpyg	2	f	0.52	28.1	1.454	0.269	hypoxia	2	0.836666667	1	Ucrit1norm	66.4565
rpyg	2	f	0.52	28.1	1.454	0.269	hypoxia	2	0.836666667	1	Ucrit2norm	
rpyg	2	f	0.52	28.1	1.454	0.269	hypoxia	2	0.836666667	1	Ucrit3norm	
rpyg	2	f	0.52	28.1	1.454	0.269	hypoxia	2	0.836666667	1	Ucrit1hyp	53.4462
rpyg	2	f	0.52	28.1	1.454	0.269	hypoxia	2	0.836666667	1	Ucrit2hyp	
rpyg	2	f	0.52	28.1	1.454	0.269	hypoxia	2	0.836666667	1	Ucrit3hyp	
rpyg	2	f	0.52	28.1	1.454	0.269	normoxia	2	0	0	Ucrit1norm	66.4565
rpyg	2	f	0.52	28.1	1.454	0.269	normoxia	2	0	0	Ucrit2norm	
rpyg	2	f	0.52	28.1	1.454	0.269	normoxia	2	0	0	Ucrit3norm	
rpyg	2	f	0.52	28.1	1.454	0.269	normoxia	2	0	0	Ucrit1hyp	53.4462
rpyg	2	f	0.52	28.1	1.454	0.269	normoxia	2	0	0	Ucrit2hyp	
rpyg	2	f	0.52	28.1	1.454	0.269	normoxia	2	0	0	Ucrit3hyp	
rpyy	1	f	0.414	30.34	1.163	0.185	normoxia	1	0	0	Ucrit1norm	90.92898
rpyy	1	f	0.414	30.34	1.163	0.185	normoxia	1	0	0	Ucrit2norm	
rpyy	1	f	0.414	30.34	1.163	0.185	normoxia	1	0	0	Ucrit3norm	
rpyy	1	f	0.414	30.34	1.163	0.185	normoxia	1	0	0	Ucrit1hyp	86.80274
rpyy	1	f	0.414	30.34	1.163	0.185	normoxia	1	0	0	Ucrit2hyp	
rpyy	1	f	0.414	30.34	1.163	0.185	normoxia	1	0	0	Ucrit3hyp	
rpyy	1	f	0.414	30.34	1.163	0.185	hypoxia	1	0	0	Ucrit1norm	90.92898

rpyy	1	f	0.414	30.34	1.163	0.185	hypoxia	1	0	0	Ucrit2norm	
rpyy	1	f	0.414	30.34	1.163	0.185	hypoxia	1	0	0	Ucrit3norm	
rpyy	1	f	0.414	30.34	1.163	0.185	hypoxia	1	0	0	Ucrit1hyp	86.80274
rpyy	1	f	0.414	30.34	1.163	0.185	hypoxia	1	0	0	Ucrit2hyp	
rpyy	1	f	0.414	30.34	1.163	0.185	hypoxia	1	0	0	Ucrit3hyp	
rpyy	1	f	0.414	30.34	1.163	0.185	normoxia	2	0	0	Ucrit1norm	90.92898
rpyy	1	f	0.414	30.34	1.163	0.185	normoxia	2	0	0	Ucrit2norm	
rpyy	1	f	0.414	30.34	1.163	0.185	normoxia	2	0	0	Ucrit3norm	
rpyy	1	f	0.414	30.34	1.163	0.185	normoxia	2	0	0	Ucrit1hyp	86.80274
rpyy	1	f	0.414	30.34	1.163	0.185	normoxia	2	0	0	Ucrit2hyp	
rpyy	1	f	0.414	30.34	1.163	0.185	normoxia	2	0	0	Ucrit3hyp	
rpyy	1	f	0.414	30.34	1.163	0.185	hypoxia	2	0.688333333	1	Ucrit1norm	90.92898
rpyy	1	f	0.414	30.34	1.163	0.185	hypoxia	2	0.688333333	1	Ucrit2norm	
rpyy	1	f	0.414	30.34	1.163	0.185	hypoxia	2	0.688333333	1	Ucrit3norm	
rpyy	1	f	0.414	30.34	1.163	0.185	hypoxia	2	0.688333333	1	Ucrit1hyp	86.80274
rpyy	1	f	0.414	30.34	1.163	0.185	hypoxia	2	0.688333333	1	Ucrit2hyp	
rpyy	1	f	0.414	30.34	1.163	0.185	hypoxia	2	0.688333333	1	Ucrit3hyp	
rrgp	2	f	0.649	33.67	0.859	0.066	hypoxia	1	0	0	Ucrit1norm	76.96962
rrgp	2	f	0.649	33.67	0.859	0.066	hypoxia	1	0	0	Ucrit2norm	
rrgp	2	f	0.649	33.67	0.859	0.066	hypoxia	1	0	0	Ucrit3norm	
rrgp	2	f	0.649	33.67	0.859	0.066	hypoxia	1	0	0	Ucrit1hyp	57.9124
rrgp	2	f	0.649	33.67	0.859	0.066	hypoxia	1	0	0	Ucrit2hyp	
rrgp	2	f	0.649	33.67	0.859	0.066	hypoxia	1	0	0	Ucrit3hyp	
rrgp	2	f	0.649	33.67	0.859	0.066	normoxia	1	0	0	Ucrit1norm	76.96962
rrgp	2	f	0.649	33.67	0.859	0.066	normoxia	1	0	0	Ucrit2norm	
rrgp	2	f	0.649	33.67	0.859	0.066	normoxia	1	0	0	Ucrit3norm	
rrgp	2	f	0.649	33.67	0.859	0.066	normoxia	1	0	0	Ucrit1hyp	57.9124
rrgp	2	f	0.649	33.67	0.859	0.066	normoxia	1	0	0	Ucrit2hyp	
rrgp	2	f	0.649	33.67	0.859	0.066	normoxia	1	0	0	Ucrit3hyp	
rrgp	2	f	0.649	33.67	0.859	0.066	hypoxia	2	0	0	Ucrit1norm	76.96962
rrgp	2	f	0.649	33.67	0.859	0.066	hypoxia	2	0	0	Ucrit2norm	
rrgp	2	f	0.649	33.67	0.859	0.066	hypoxia	2	0	0	Ucrit3norm	
rrgp	2	f	0.649	33.67	0.859	0.066	hypoxia	2	0	0	Ucrit1hyp	57.9124

rrgp	2	f	0.649	33.67	0.859	0.066	hypoxia	2	0	0	Ucrit2hyp	
rrgp	2	f	0.649	33.67	0.859	0.066	hypoxia	2	0	0	Ucrit3hyp	
rrgp	2	f	0.649	33.67	0.859	0.066	normoxia	2	0	0	Ucrit1norm	76.96962
rrgp	2	f	0.649	33.67	0.859	0.066	normoxia	2	0	0	Ucrit2norm	
rrgp	2	f	0.649	33.67	0.859	0.066	normoxia	2	0	0	Ucrit3norm	
rrgp	2	f	0.649	33.67	0.859	0.066	normoxia	2	0	0	Ucrit1hyp	57.9124
rrgp	2	f	0.649	33.67	0.859	0.066	normoxia	2	0	0	Ucrit2hyp	
rrgp	2	f	0.649	33.67	0.859	0.066	normoxia	2	0	0	Ucrit3hyp	
rrgy	4	m	0.497	31.82	1.086	0.183	normoxia	1	0	0	Ucrit1norm	90.97338
rrgy	4	m	0.497	31.82	1.086	0.183	normoxia	1	0	0	Ucrit2norm	85.37306
rrgy	4	m	0.497	31.82	1.086	0.183	normoxia	1	0	0	Ucrit3norm	86.7095
rrgy	4	m	0.497	31.82	1.086	0.183	normoxia	1	0	0	Ucrit1hyp	66.44016
rrgy	4	m	0.497	31.82	1.086	0.183	normoxia	1	0	0	Ucrit2hyp	73.44056
rrgy	4	m	0.497	31.82	1.086	0.183	normoxia	1	0	0	Ucrit3hyp	71.14952
rrgy	4	m	0.497	31.82	1.086	0.183	hypoxia	1	0	0	Ucrit1norm	90.97338
rrgy	4	m	0.497	31.82	1.086	0.183	hypoxia	1	0	0	Ucrit2norm	85.37306
rrgy	4	m	0.497	31.82	1.086	0.183	hypoxia	1	0	0	Ucrit3norm	86.7095
rrgy	4	m	0.497	31.82	1.086	0.183	hypoxia	1	0	0	Ucrit1hyp	66.44016
rrgy	4	m	0.497	31.82	1.086	0.183	hypoxia	1	0	0	Ucrit2hyp	73.44056
rrgy	4	m	0.497	31.82	1.086	0.183	hypoxia	1	0	0	Ucrit3hyp	71.14952
rrgy	4	m	0.497	31.82	1.086	0.183	normoxia	2	0	0	Ucrit1norm	90.97338
rrgy	4	m	0.497	31.82	1.086	0.183	normoxia	2	0	0	Ucrit2norm	85.37306
rrgy	4	m	0.497	31.82	1.086	0.183	normoxia	2	0	0	Ucrit3norm	86.7095
rrgy	4	m	0.497	31.82	1.086	0.183	normoxia	2	0	0	Ucrit1hyp	66.44016
rrgy	4	m	0.497	31.82	1.086	0.183	normoxia	2	0	0	Ucrit2hyp	73.44056
rrgy	4	m	0.497	31.82	1.086	0.183	normoxia	2	0	0	Ucrit3hyp	71.14952
rrgy	4	m	0.497	31.82	1.086	0.183	hypoxia	2	0	0	Ucrit1norm	90.97338
rrgy	4	m	0.497	31.82	1.086	0.183	hypoxia	2	0	0	Ucrit2norm	85.37306
rrgy	4	m	0.497	31.82	1.086	0.183	hypoxia	2	0	0	Ucrit3norm	86.7095
rrgy	4	m	0.497	31.82	1.086	0.183	hypoxia	2	0	0	Ucrit1hyp	66.44016
rrgy	4	m	0.497	31.82	1.086	0.183	hypoxia	2	0	0	Ucrit2hyp	73.44056
rrgy	4	m	0.497	31.82	1.086	0.183	hypoxia	2	0	0	Ucrit3hyp	71.14952
rrpg	4	m	0.464	31.96	0.96	0.142	normoxia	1	0	0	Ucrit1norm	76.32048

rrpg	4	m	0.464	31.96	0.96	0.142	normoxia	1	0	0	Ucrit2norm	76.76792
rrpg	4	m	0.464	31.96	0.96	0.142	normoxia	1	0	0	Ucrit3norm	79.93196
rrpg	4	m	0.464	31.96	0.96	0.142	normoxia	1	0	0	Ucrit1hyp	68.96968
rrpg	4	m	0.464	31.96	0.96	0.142	normoxia	1	0	0	Ucrit2hyp	71.71824
rrpg	4	m	0.464	31.96	0.96	0.142	normoxia	1	0	0	Ucrit3hyp	71.78216
rrpg	4	m	0.464	31.96	0.96	0.142	hypoxia	1	0	0	Ucrit1norm	76.32048
rrpg	4	m	0.464	31.96	0.96	0.142	hypoxia	1	0	0	Ucrit2norm	76.76792
rrpg	4	m	0.464	31.96	0.96	0.142	hypoxia	1	0	0	Ucrit3norm	79.93196
rrpg	4	m	0.464	31.96	0.96	0.142	hypoxia	1	0	0	Ucrit1hyp	68.96968
rrpg	4	m	0.464	31.96	0.96	0.142	hypoxia	1	0	0	Ucrit2hyp	71.71824
rrpg	4	m	0.464	31.96	0.96	0.142	hypoxia	1	0	0	Ucrit3hyp	71.78216
rrpg	4	m	0.464	31.96	0.96	0.142	normoxia	2	0	0	Ucrit1norm	76.32048
rrpg	4	m	0.464	31.96	0.96	0.142	normoxia	2	0	0	Ucrit2norm	76.76792
rrpg	4	m	0.464	31.96	0.96	0.142	normoxia	2	0	0	Ucrit3norm	79.93196
rrpg	4	m	0.464	31.96	0.96	0.142	normoxia	2	0	0	Ucrit1hyp	68.96968
rrpg	4	m	0.464	31.96	0.96	0.142	normoxia	2	0	0	Ucrit2hyp	71.71824
rrpg	4	m	0.464	31.96	0.96	0.142	normoxia	2	0	0	Ucrit3hyp	71.78216
rrpg	4	m	0.464	31.96	0.96	0.142	hypoxia	2	0	0	Ucrit1norm	76.32048
rrpg	4	m	0.464	31.96	0.96	0.142	hypoxia	2	0	0	Ucrit2norm	76.76792
rrpg	4	m	0.464	31.96	0.96	0.142	hypoxia	2	0	0	Ucrit3norm	79.93196
rrpg	4	m	0.464	31.96	0.96	0.142	hypoxia	2	0	0	Ucrit1hyp	68.96968
rrpg	4	m	0.464	31.96	0.96	0.142	hypoxia	2	0	0	Ucrit2hyp	71.71824
rrpg	4	m	0.464	31.96	0.96	0.142	hypoxia	2	0	0	Ucrit3hyp	71.78216
rrpp	4	f	0.727	34.32			normoxia	1	0	0	Ucrit1norm	91.05096
rrpp	4	f	0.727	34.32			normoxia	1	0	0	Ucrit2norm	79.89696
rrpp	4	f	0.727	34.32			normoxia	1	0	0	Ucrit3norm	81.09816
rrpp	4	f	0.727	34.32			normoxia	1	0	0	Ucrit1hyp	61.39848
rrpp	4	f	0.727	34.32			normoxia	1	0	0	Ucrit2hyp	67.74768
rrpp	4	f	0.727	34.32			normoxia	1	0	0	Ucrit3hyp	66.51216
rrpp	4	f	0.727	34.32			hypoxia	1	0.84	1	Ucrit1norm	91.05096
rrpp	4	f	0.727	34.32			hypoxia	1	0.84	1	Ucrit2norm	79.89696
rrpp	4	f	0.727	34.32			hypoxia	1	0.84	1	Ucrit3norm	81.09816
rrpp	4	f	0.727	34.32			hypoxia	1	0.84	1	Ucrit1hyp	61.39848

rrpp	4	f	0.727	34.32			hypoxia	1	0.84	1	Ucrit2hyp	67.74768
rrpp	4	f	0.727	34.32			hypoxia	1	0.84	1	Ucrit3hyp	66.51216
rrpp	4	f	0.727	34.32			normoxia	2	0	0	Ucrit1norm	91.05096
rrpp	4	f	0.727	34.32			normoxia	2	0	0	Ucrit2norm	79.89696
rrpp	4	f	0.727	34.32			normoxia	2	0	0	Ucrit3norm	81.09816
rrpp	4	f	0.727	34.32			normoxia	2	0	0	Ucrit1hyp	61.39848
rrpp	4	f	0.727	34.32			normoxia	2	0	0	Ucrit2hyp	67.74768
rrpp	4	f	0.727	34.32			normoxia	2	0	0	Ucrit3hyp	66.51216
rrpp	4	f	0.727	34.32			hypoxia	2	0.813333333	1	Ucrit1norm	91.05096
rrpp	4	f	0.727	34.32			hypoxia	2	0.813333333	1	Ucrit2norm	79.89696
rrpp	4	f	0.727	34.32			hypoxia	2	0.813333333	1	Ucrit3norm	81.09816
rrpp	4	f	0.727	34.32			hypoxia	2	0.813333333	1	Ucrit1hyp	61.39848
rrpp	4	f	0.727	34.32			hypoxia	2	0.813333333	1	Ucrit2hyp	67.74768
rrpp	4	f	0.727	34.32			hypoxia	2	0.813333333	1	Ucrit3hyp	66.51216
rrpy	3	f	0.372	28.91	0.655	0.097	hypoxia	1	0	0	Ucrit1norm	79.03994
rrpy	3	f	0.372	28.91	0.655	0.097	hypoxia	1	0	0	Ucrit2norm	
rrpy	3	f	0.372	28.91	0.655	0.097	hypoxia	1	0	0	Ucrit3norm	
rrpy	3	f	0.372	28.91	0.655	0.097	hypoxia	1	0	0	Ucrit1hyp	77.27643
rrpy	3	f	0.372	28.91	0.655	0.097	hypoxia	1	0	0	Ucrit2hyp	
rrpy	3	f	0.372	28.91	0.655	0.097	hypoxia	1	0	0	Ucrit3hyp	
rrpy	3	f	0.372	28.91	0.655	0.097	normoxia	1	0	0	Ucrit1norm	79.03994
rrpy	3	f	0.372	28.91	0.655	0.097	normoxia	1	0	0	Ucrit2norm	
rrpy	3	f	0.372	28.91	0.655	0.097	normoxia	1	0	0	Ucrit3norm	
rrpy	3	f	0.372	28.91	0.655	0.097	normoxia	1	0	0	Ucrit1hyp	77.27643
rrpy	3	f	0.372	28.91	0.655	0.097	normoxia	1	0	0	Ucrit2hyp	
rrpy	3	f	0.372	28.91	0.655	0.097	normoxia	1	0	0	Ucrit3hyp	
rrpy	3	f	0.372	28.91	0.655	0.097	hypoxia	2	0	0	Ucrit1norm	79.03994
rrpy	3	f	0.372	28.91	0.655	0.097	hypoxia	2	0	0	Ucrit2norm	
rrpy	3	f	0.372	28.91	0.655	0.097	hypoxia	2	0	0	Ucrit3norm	
rrpy	3	f	0.372	28.91	0.655	0.097	hypoxia	2	0	0	Ucrit1hyp	77.27643
rrpy	3	f	0.372	28.91	0.655	0.097	hypoxia	2	0	0	Ucrit2hyp	
rrpy	3	f	0.372	28.91	0.655	0.097	hypoxia	2	0	0	Ucrit3hyp	
rrpy	3	f	0.372	28.91	0.655	0.097	normoxia	2	0	0	Ucrit1norm	79.03994

rrpy	3	f	0.372	28.91	0.655	0.097	normoxia	2	0	0	Ucrit2norm	
rrpy	3	f	0.372	28.91	0.655	0.097	normoxia	2	0	0	Ucrit3norm	
rrpy	3	f	0.372	28.91	0.655	0.097	normoxia	2	0	0	Ucrit1hyp	77.27643
rrpy	3	f	0.372	28.91	0.655	0.097	normoxia	2	0	0	Ucrit2hyp	
rrpy	3	f	0.372	28.91	0.655	0.097	normoxia	2	0	0	Ucrit3hyp	
rrrp	3	m	0.401	30.08	1.059	0.137	hypoxia	1	0	0	Ucrit1norm	82.14848
rrrp	3	m	0.401	30.08	1.059	0.137	hypoxia	1	0	0	Ucrit2norm	
rrrp	3	m	0.401	30.08	1.059	0.137	hypoxia	1	0	0	Ucrit3norm	
rrrp	3	m	0.401	30.08	1.059	0.137	hypoxia	1	0	0	Ucrit1hyp	67.89056
rrrp	3	m	0.401	30.08	1.059	0.137	hypoxia	1	0	0	Ucrit2hyp	
rrrp	3	m	0.401	30.08	1.059	0.137	hypoxia	1	0	0	Ucrit3hyp	
rrrp	3	m	0.401	30.08	1.059	0.137	normoxia	1	0	0	Ucrit1norm	82.14848
rrrp	3	m	0.401	30.08	1.059	0.137	normoxia	1	0	0	Ucrit2norm	
rrrp	3	m	0.401	30.08	1.059	0.137	normoxia	1	0	0	Ucrit3norm	
rrrp	3	m	0.401	30.08	1.059	0.137	normoxia	1	0	0	Ucrit1hyp	67.89056
rrrp	3	m	0.401	30.08	1.059	0.137	normoxia	1	0	0	Ucrit2hyp	
rrrp	3	m	0.401	30.08	1.059	0.137	normoxia	1	0	0	Ucrit3hyp	
rrrp	3	m	0.401	30.08	1.059	0.137	hypoxia	2	0	0	Ucrit1norm	82.14848
rrrp	3	m	0.401	30.08	1.059	0.137	hypoxia	2	0	0	Ucrit2norm	
rrrp	3	m	0.401	30.08	1.059	0.137	hypoxia	2	0	0	Ucrit3norm	
rrrp	3	m	0.401	30.08	1.059	0.137	hypoxia	2	0	0	Ucrit1hyp	67.89056
rrrp	3	m	0.401	30.08	1.059	0.137	hypoxia	2	0	0	Ucrit2hyp	
rrrp	3	m	0.401	30.08	1.059	0.137	hypoxia	2	0	0	Ucrit3hyp	
rrrp	3	m	0.401	30.08	1.059	0.137	normoxia	2	0	0	Ucrit1norm	82.14848
rrrp	3	m	0.401	30.08	1.059	0.137	normoxia	2	0	0	Ucrit2norm	
rrrp	3	m	0.401	30.08	1.059	0.137	normoxia	2	0	0	Ucrit3norm	
rrrp	3	m	0.401	30.08	1.059	0.137	normoxia	2	0	0	Ucrit1hyp	67.89056
rrrp	3	m	0.401	30.08	1.059	0.137	normoxia	2	0	0	Ucrit2hyp	
rrrp	3	m	0.401	30.08	1.059	0.137	normoxia	2	0	0	Ucrit3hyp	
rrrr	4	f	0.554	33.49	1.417	0.212	normoxia	1	0	0	Ucrit1norm	82.55285
rrrr	4	f	0.554	33.49	1.417	0.212	normoxia	1	0	0	Ucrit2norm	79.20385
rrrr	4	f	0.554	33.49	1.417	0.212	normoxia	1	0	0	Ucrit3norm	88.01172
rrrr	4	f	0.554	33.49	1.417	0.212	normoxia	1	0	0	Ucrit1hyp	68.15215

rrrr	4	f	0.554	33.49	1.417	0.212	normoxia	1	0	0	Ucrit2hyp	73.17565
rrrr	4	f	0.554	33.49	1.417	0.212	normoxia	1	0	0	Ucrit3hyp	74.04639
rrrr	4	f	0.554	33.49	1.417	0.212	hypoxia	1	0	0	Ucrit1norm	82.55285
rrrr	4	f	0.554	33.49	1.417	0.212	hypoxia	1	0	0	Ucrit2norm	79.20385
rrrr	4	f	0.554	33.49	1.417	0.212	hypoxia	1	0	0	Ucrit3norm	88.01172
rrrr	4	f	0.554	33.49	1.417	0.212	hypoxia	1	0	0	Ucrit1hyp	68.15215
rrrr	4	f	0.554	33.49	1.417	0.212	hypoxia	1	0	0	Ucrit2hyp	73.17565
rrrr	4	f	0.554	33.49	1.417	0.212	hypoxia	1	0	0	Ucrit3hyp	74.04639
rrrr	4	f	0.554	33.49	1.417	0.212	normoxia	2	0	0	Ucrit1norm	82.55285
rrrr	4	f	0.554	33.49	1.417	0.212	normoxia	2	0	0	Ucrit2norm	79.20385
rrrr	4	f	0.554	33.49	1.417	0.212	normoxia	2	0	0	Ucrit3norm	88.01172
rrrr	4	f	0.554	33.49	1.417	0.212	normoxia	2	0	0	Ucrit1hyp	68.15215
rrrr	4	f	0.554	33.49	1.417	0.212	normoxia	2	0	0	Ucrit2hyp	73.17565
rrrr	4	f	0.554	33.49	1.417	0.212	normoxia	2	0	0	Ucrit3hyp	74.04639
rrrr	4	f	0.554	33.49	1.417	0.212	hypoxia	2	0	0	Ucrit1norm	82.55285
rrrr	4	f	0.554	33.49	1.417	0.212	hypoxia	2	0	0	Ucrit2norm	79.20385
rrrr	4	f	0.554	33.49	1.417	0.212	hypoxia	2	0	0	Ucrit3norm	88.01172
rrrr	4	f	0.554	33.49	1.417	0.212	hypoxia	2	0	0	Ucrit1hyp	68.15215
rrrr	4	f	0.554	33.49	1.417	0.212	hypoxia	2	0	0	Ucrit2hyp	73.17565
rrrr	4	f	0.554	33.49	1.417	0.212	hypoxia	2	0	0	Ucrit3hyp	74.04639
rrry	3	f	0.663	34.69	1.023	0.171	hypoxia	1	0	0	Ucrit1norm	78.08719
rrry	3	f	0.663	34.69	1.023	0.171	hypoxia	1	0	0	Ucrit2norm	
rrry	3	f	0.663	34.69	1.023	0.171	hypoxia	1	0	0	Ucrit3norm	
rrry	3	f	0.663	34.69	1.023	0.171	hypoxia	1	0	0	Ucrit1hyp	64.38464
rrry	3	f	0.663	34.69	1.023	0.171	hypoxia	1	0	0	Ucrit2hyp	
rrry	3	f	0.663	34.69	1.023	0.171	hypoxia	1	0	0	Ucrit3hyp	
rrry	3	f	0.663	34.69	1.023	0.171	normoxia	1	0	0	Ucrit1norm	78.08719
rrry	3	f	0.663	34.69	1.023	0.171	normoxia	1	0	0	Ucrit2norm	
rrry	3	f	0.663	34.69	1.023	0.171	normoxia	1	0	0	Ucrit3norm	
rrry	3	f	0.663	34.69	1.023	0.171	normoxia	1	0	0	Ucrit1hyp	64.38464
rrry	3	f	0.663	34.69	1.023	0.171	normoxia	1	0	0	Ucrit2hyp	
rrry	3	f	0.663	34.69	1.023	0.171	normoxia	1	0	0	Ucrit3hyp	
rrry	3	f	0.663	34.69	1.023	0.171	hypoxia	2	0.593333333	1	Ucrit1norm	78.08719

rrry	3	f	0.663	34.69	1.023	0.171	hypoxia	2	0.593333333	1	Ucrit2norm	
rrry	3	f	0.663	34.69	1.023	0.171	hypoxia	2	0.593333333	1	Ucrit3norm	
rrry	3	f	0.663	34.69	1.023	0.171	hypoxia	2	0.593333333	1	Ucrit1hyp	64.38464
rrry	3	f	0.663	34.69	1.023	0.171	hypoxia	2	0.593333333	1	Ucrit2hyp	
rrry	3	f	0.663	34.69	1.023	0.171	hypoxia	2	0.593333333	1	Ucrit3hyp	
rrry	3	f	0.663	34.69	1.023	0.171	normoxia	2	0	0	Ucrit1norm	78.08719
rrry	3	f	0.663	34.69	1.023	0.171	normoxia	2	0	0	Ucrit2norm	
rrry	3	f	0.663	34.69	1.023	0.171	normoxia	2	0	0	Ucrit3norm	
rrry	3	f	0.663	34.69	1.023	0.171	normoxia	2	0	0	Ucrit1hyp	64.38464
rrry	3	f	0.663	34.69	1.023	0.171	normoxia	2	0	0	Ucrit2hyp	
rrry	3	f	0.663	34.69	1.023	0.171	normoxia	2	0	0	Ucrit3hyp	
rryg	2				0.964	0.176	hypoxia	1	0	0		
rryg	2				0.964	0.176	normoxia	1	0	0		
rryg	2				0.964	0.176	hypoxia	2	0	0		
rryg	2				0.964	0.176	normoxia	2	0	0		
rryp	3	m	0.532	34.52	1.333	0.165	hypoxia	1	0	0	Ucrit1norm	79.84476
rryp	3	m	0.532	34.52	1.333	0.165	hypoxia	1	0	0	Ucrit2norm	
rryp	3	m	0.532	34.52	1.333	0.165	hypoxia	1	0	0	Ucrit3norm	
rryp	3	m	0.532	34.52	1.333	0.165	hypoxia	1	0	0	Ucrit1hyp	78.63656
rryp	3	m	0.532	34.52	1.333	0.165	hypoxia	1	0	0	Ucrit2hyp	
rryp	3	m	0.532	34.52	1.333	0.165	hypoxia	1	0	0	Ucrit3hyp	
rryp	3	m	0.532	34.52	1.333	0.165	normoxia	1	0	0	Ucrit1norm	79.84476
rryp	3	m	0.532	34.52	1.333	0.165	normoxia	1	0	0	Ucrit2norm	
rryp	3	m	0.532	34.52	1.333	0.165	normoxia	1	0	0	Ucrit3norm	
rryp	3	m	0.532	34.52	1.333	0.165	normoxia	1	0	0	Ucrit1hyp	78.63656
rryp	3	m	0.532	34.52	1.333	0.165	normoxia	1	0	0	Ucrit2hyp	
rryp	3	m	0.532	34.52	1.333	0.165	normoxia	1	0	0	Ucrit3hyp	
rryp	3	m	0.532	34.52	1.333	0.165	hypoxia	2	0	0	Ucrit1norm	79.84476
rryp	3	m	0.532	34.52	1.333	0.165	hypoxia	2	0	0	Ucrit2norm	
rryp	3	m	0.532	34.52	1.333	0.165	hypoxia	2	0	0	Ucrit3norm	
rryp	3	m	0.532	34.52	1.333	0.165	hypoxia	2	0	0	Ucrit1hyp	78.63656
rryp	3	m	0.532	34.52	1.333	0.165	hypoxia	2	0	0	Ucrit2hyp	
rryp	3	m	0.532	34.52	1.333	0.165	hypoxia	2	0	0	Ucrit3hyp	

rryp	3	m	0.532	34.52	1.333	0.165	normoxia	2	0	0	Ucrit1norm	79.84476
rryp	3	m	0.532	34.52	1.333	0.165	normoxia	2	0	0	Ucrit2norm	
rryp	3	m	0.532	34.52	1.333	0.165	normoxia	2	0	0	Ucrit3norm	
rryp	3	m	0.532	34.52	1.333	0.165	normoxia	2	0	0	Ucrit1hyp	78.63656
rryp	3	m	0.532	34.52	1.333	0.165	normoxia	2	0	0	Ucrit2hyp	
rryp	3	m	0.532	34.52	1.333	0.165	normoxia	2	0	0	Ucrit3hyp	
rryr	3	f	0.556	31.4	0.871	0.147	hypoxia	1	0	0	Ucrit1norm	76.2078
rryr	3	f	0.556	31.4	0.871	0.147	hypoxia	1	0	0	Ucrit2norm	
rryr	3	f	0.556	31.4	0.871	0.147	hypoxia	1	0	0	Ucrit3norm	
rryr	3	f	0.556	31.4	0.871	0.147	hypoxia	1	0	0	Ucrit1hyp	53.38
rryr	3	f	0.556	31.4	0.871	0.147	hypoxia	1	0	0	Ucrit2hyp	
rryr	3	f	0.556	31.4	0.871	0.147	hypoxia	1	0	0	Ucrit3hyp	
rryr	3	f	0.556	31.4	0.871	0.147	normoxia	1	0	0	Ucrit1norm	76.2078
rryr	3	f	0.556	31.4	0.871	0.147	normoxia	1	0	0	Ucrit2norm	
rryr	3	f	0.556	31.4	0.871	0.147	normoxia	1	0	0	Ucrit3norm	
rryr	3	f	0.556	31.4	0.871	0.147	normoxia	1	0	0	Ucrit1hyp	53.38
rryr	3	f	0.556	31.4	0.871	0.147	normoxia	1	0	0	Ucrit2hyp	
rryr	3	f	0.556	31.4	0.871	0.147	normoxia	1	0	0	Ucrit3hyp	
rryr	3	f	0.556	31.4	0.871	0.147	hypoxia	2	0.746666667	1	Ucrit1norm	76.2078
rryr	3	f	0.556	31.4	0.871	0.147	hypoxia	2	0.746666667	1	Ucrit2norm	
rryr	3	f	0.556	31.4	0.871	0.147	hypoxia	2	0.746666667	1	Ucrit3norm	
rryr	3	f	0.556	31.4	0.871	0.147	hypoxia	2	0.746666667	1	Ucrit1hyp	53.38
rryr	3	f	0.556	31.4	0.871	0.147	hypoxia	2	0.746666667	1	Ucrit2hyp	
rryr	3	f	0.556	31.4	0.871	0.147	hypoxia	2	0.746666667	1	Ucrit3hyp	
rryr	3	f	0.556	31.4	0.871	0.147	normoxia	2	0	0	Ucrit1norm	76.2078
rryr	3	f	0.556	31.4	0.871	0.147	normoxia	2	0	0	Ucrit2norm	
rryr	3	f	0.556	31.4	0.871	0.147	normoxia	2	0	0	Ucrit3norm	
rryr	3	f	0.556	31.4	0.871	0.147	normoxia	2	0	0	Ucrit1hyp	53.38
rryr	3	f	0.556	31.4	0.871	0.147	normoxia	2	0	0	Ucrit2hyp	
rryr	3	f	0.556	31.4	0.871	0.147	normoxia	2	0	0	Ucrit3hyp	
rryy	2	f	0.587	34.15	1.167	0.21	hypoxia	1	0	0	Ucrit1norm	79.8427
rryy	2	f	0.587	34.15	1.167	0.21	hypoxia	1	0	0	Ucrit2norm	
rryy	2	f	0.587	34.15	1.167	0.21	hypoxia	1	0	0	Ucrit3norm	

rryy	2	f	0.587	34.15	1.167	0.21	hypoxia	1	0	0	Ucrit1hyp	77.9986
rryy	2	f	0.587	34.15	1.167	0.21	hypoxia	1	0	0	Ucrit2hyp	
rryy	2	f	0.587	34.15	1.167	0.21	hypoxia	1	0	0	Ucrit3hyp	
rryy	2	f	0.587	34.15	1.167	0.21	normoxia	1	0	0	Ucrit1norm	79.8427
rryy	2	f	0.587	34.15	1.167	0.21	normoxia	1	0	0	Ucrit2norm	
rryy	2	f	0.587	34.15	1.167	0.21	normoxia	1	0	0	Ucrit3norm	
rryy	2	f	0.587	34.15	1.167	0.21	normoxia	1	0	0	Ucrit1hyp	77.9986
rryy	2	f	0.587	34.15	1.167	0.21	normoxia	1	0	0	Ucrit2hyp	
rryy	2	f	0.587	34.15	1.167	0.21	normoxia	1	0	0	Ucrit3hyp	
rryy	2	f	0.587	34.15	1.167	0.21	hypoxia	2	0.886666667	1	Ucrit1norm	79.8427
rryy	2	f	0.587	34.15	1.167	0.21	hypoxia	2	0.886666667	1	Ucrit2norm	
rryy	2	f	0.587	34.15	1.167	0.21	hypoxia	2	0.886666667	1	Ucrit3norm	
rryy	2	f	0.587	34.15	1.167	0.21	hypoxia	2	0.886666667	1	Ucrit1hyp	77.9986
rryy	2	f	0.587	34.15	1.167	0.21	hypoxia	2	0.886666667	1	Ucrit2hyp	
rryy	2	f	0.587	34.15	1.167	0.21	hypoxia	2	0.886666667	1	Ucrit3hyp	
rryy	2	f	0.587	34.15	1.167	0.21	normoxia	2	0	0	Ucrit1norm	79.8427
rryy	2	f	0.587	34.15	1.167	0.21	normoxia	2	0	0	Ucrit2norm	
rryy	2	f	0.587	34.15	1.167	0.21	normoxia	2	0	0	Ucrit3norm	
rryy	2	f	0.587	34.15	1.167	0.21	normoxia	2	0	0	Ucrit1hyp	77.9986
rryy	2	f	0.587	34.15	1.167	0.21	normoxia	2	0	0	Ucrit2hyp	
rryy	2	f	0.587	34.15	1.167	0.21	normoxia	2	0	0	Ucrit3hyp	
rygg	3	m	0.673	33.9	0.664	0.188	hypoxia	1	0	0	Ucrit1norm	81.8685
rygg	3	m	0.673	33.9	0.664	0.188	hypoxia	1	0	0	Ucrit2norm	
rygg	3	m	0.673	33.9	0.664	0.188	hypoxia	1	0	0	Ucrit3norm	
rygg	3	m	0.673	33.9	0.664	0.188	hypoxia	1	0	0	Ucrit1hyp	67.9356
rygg	3	m	0.673	33.9	0.664	0.188	hypoxia	1	0	0	Ucrit2hyp	
rygg	3	m	0.673	33.9	0.664	0.188	hypoxia	1	0	0	Ucrit3hyp	
rygg	3	m	0.673	33.9	0.664	0.188	normoxia	1	0	0	Ucrit1norm	81.8685
rygg	3	m	0.673	33.9	0.664	0.188	normoxia	1	0	0	Ucrit2norm	
rygg	3	m	0.673	33.9	0.664	0.188	normoxia	1	0	0	Ucrit3norm	
rygg	3	m	0.673	33.9	0.664	0.188	normoxia	1	0	0	Ucrit1hyp	67.9356
rygg	3	m	0.673	33.9	0.664	0.188	normoxia	1	0	0	Ucrit2hyp	
rygg	3	m	0.673	33.9	0.664	0.188	normoxia	1	0	0	Ucrit3hyp	

rygg	3	m	0.673	33.9	0.664	0.188	hypoxia	2	0	0	Ucrit1norm	81.8685
rygg	3	m	0.673	33.9	0.664	0.188	hypoxia	2	0	0	Ucrit2norm	
rygg	3	m	0.673	33.9	0.664	0.188	hypoxia	2	0	0	Ucrit3norm	
rygg	3	m	0.673	33.9	0.664	0.188	hypoxia	2	0	0	Ucrit1hyp	67.9356
rygg	3	m	0.673	33.9	0.664	0.188	hypoxia	2	0	0	Ucrit2hyp	
rygg	3	m	0.673	33.9	0.664	0.188	hypoxia	2	0	0	Ucrit3hyp	
rygg	3	m	0.673	33.9	0.664	0.188	normoxia	2	0	0	Ucrit1norm	81.8685
rygg	3	m	0.673	33.9	0.664	0.188	normoxia	2	0	0	Ucrit2norm	
rygg	3	m	0.673	33.9	0.664	0.188	normoxia	2	0	0	Ucrit3norm	
rygg	3	m	0.673	33.9	0.664	0.188	normoxia	2	0	0	Ucrit1hyp	67.9356
rygg	3	m	0.673	33.9	0.664	0.188	normoxia	2	0	0	Ucrit2hyp	
rygg	3	m	0.673	33.9	0.664	0.188	normoxia	2	0	0	Ucrit3hyp	
rygp	3	f	0.494	29.88	0.999	0.199	hypoxia	1	0	0	Ucrit1norm	75.8952
rygp	3	f	0.494	29.88	0.999	0.199	hypoxia	1	0	0	Ucrit2norm	
rygp	3	f	0.494	29.88	0.999	0.199	hypoxia	1	0	0	Ucrit3norm	
rygp	3	f	0.494	29.88	0.999	0.199	hypoxia	1	0	0	Ucrit1hyp	67.94712
rygp	3	f	0.494	29.88	0.999	0.199	hypoxia	1	0	0	Ucrit2hyp	
rygp	3	f	0.494	29.88	0.999	0.199	hypoxia	1	0	0	Ucrit3hyp	
rygp	3	f	0.494	29.88	0.999	0.199	normoxia	1	0	0	Ucrit1norm	75.8952
rygp	3	f	0.494	29.88	0.999	0.199	normoxia	1	0	0	Ucrit2norm	
rygp	3	f	0.494	29.88	0.999	0.199	normoxia	1	0	0	Ucrit3norm	
rygp	3	f	0.494	29.88	0.999	0.199	normoxia	1	0	0	Ucrit1hyp	67.94712
rygp	3	f	0.494	29.88	0.999	0.199	normoxia	1	0	0	Ucrit2hyp	
rygp	3	f	0.494	29.88	0.999	0.199	normoxia	1	0	0	Ucrit3hyp	
rygp	3	f	0.494	29.88	0.999	0.199	hypoxia	2	0	0	Ucrit1norm	75.8952
rygp	3	f	0.494	29.88	0.999	0.199	hypoxia	2	0	0	Ucrit2norm	
rygp	3	f	0.494	29.88	0.999	0.199	hypoxia	2	0	0	Ucrit3norm	
rygp	3	f	0.494	29.88	0.999	0.199	hypoxia	2	0	0	Ucrit1hyp	67.94712
rygp	3	f	0.494	29.88	0.999	0.199	hypoxia	2	0	0	Ucrit2hyp	
rygp	3	f	0.494	29.88	0.999	0.199	hypoxia	2	0	0	Ucrit3hyp	
rygp	3	f	0.494	29.88	0.999	0.199	normoxia	2	0	0	Ucrit1norm	75.8952
rygp	3	f	0.494	29.88	0.999	0.199	normoxia	2	0	0	Ucrit2norm	
rygp	3	f	0.494	29.88	0.999	0.199	normoxia	2	0	0	Ucrit3norm	

rygp	3	f	0.494	29.88	0.999	0.199	normoxia	2	0	0	Ucrit1hyp	67.94712
rygp	3	f	0.494	29.88	0.999	0.199	normoxia	2	0	0	Ucrit2hyp	
rygp	3	f	0.494	29.88	0.999	0.199	normoxia	2	0	0	Ucrit3hyp	
rygy	2	m	0.559	33.09	0.737	0.126	hypoxia	1	0	0	Ucrit1norm	73.7907
rygy	2	m	0.559	33.09	0.737	0.126	hypoxia	1	0	0	Ucrit2norm	
rygy	2	m	0.559	33.09	0.737	0.126	hypoxia	1	0	0	Ucrit3norm	
rygy	2	m	0.559	33.09	0.737	0.126	hypoxia	1	0	0	Ucrit1hyp	58.47003
rygy	2	m	0.559	33.09	0.737	0.126	hypoxia	1	0	0	Ucrit2hyp	
rygy	2	m	0.559	33.09	0.737	0.126	hypoxia	1	0	0	Ucrit3hyp	
rygy	2	m	0.559	33.09	0.737	0.126	normoxia	1	0	0	Ucrit1norm	73.7907
rygy	2	m	0.559	33.09	0.737	0.126	normoxia	1	0	0	Ucrit2norm	
rygy	2	m	0.559	33.09	0.737	0.126	normoxia	1	0	0	Ucrit3norm	
rygy	2	m	0.559	33.09	0.737	0.126	normoxia	1	0	0	Ucrit1hyp	58.47003
rygy	2	m	0.559	33.09	0.737	0.126	normoxia	1	0	0	Ucrit2hyp	
rygy	2	m	0.559	33.09	0.737	0.126	normoxia	1	0	0	Ucrit3hyp	
rygy	2	m	0.559	33.09	0.737	0.126	hypoxia	2	0	0	Ucrit1norm	73.7907
rygy	2	m	0.559	33.09	0.737	0.126	hypoxia	2	0	0	Ucrit2norm	
rygy	2	m	0.559	33.09	0.737	0.126	hypoxia	2	0	0	Ucrit3norm	
rygy	2	m	0.559	33.09	0.737	0.126	hypoxia	2	0	0	Ucrit1hyp	58.47003
rygy	2	m	0.559	33.09	0.737	0.126	hypoxia	2	0	0	Ucrit2hyp	
rygy	2	m	0.559	33.09	0.737	0.126	hypoxia	2	0	0	Ucrit3hyp	
rygy	2	m	0.559	33.09	0.737	0.126	normoxia	2	0	0	Ucrit1norm	73.7907
rygy	2	m	0.559	33.09	0.737	0.126	normoxia	2	0	0	Ucrit2norm	
rygy	2	m	0.559	33.09	0.737	0.126	normoxia	2	0	0	Ucrit3norm	
rygy	2	m	0.559	33.09	0.737	0.126	normoxia	2	0	0	Ucrit1hyp	58.47003
rygy	2	m	0.559	33.09	0.737	0.126	normoxia	2	0	0	Ucrit2hyp	
rygy	2	m	0.559	33.09	0.737	0.126	normoxia	2	0	0	Ucrit3hyp	
rypg	3	f	0.554	30.7	1.042	0.167	hypoxia	1	0	0	Ucrit1norm	75.215
rypg	3	f	0.554	30.7	1.042	0.167	hypoxia	1	0	0	Ucrit2norm	
rypg	3	f	0.554	30.7	1.042	0.167	hypoxia	1	0	0	Ucrit3norm	
rypg	3	f	0.554	30.7	1.042	0.167	hypoxia	1	0	0	Ucrit1hyp	59.9878
rypg	3	f	0.554	30.7	1.042	0.167	hypoxia	1	0	0	Ucrit2hyp	
rypg	3	f	0.554	30.7	1.042	0.167	hypoxia	1	0	0	Ucrit3hyp	

rypg	3	f	0.554	30.7	1.042	0.167	normoxia	1	0	0	Ucrit1norm	75.215
rypg	3	f	0.554	30.7	1.042	0.167	normoxia	1	0	0	Ucrit2norm	
rypg	3	f	0.554	30.7	1.042	0.167	normoxia	1	0	0	Ucrit3norm	
rypg	3	f	0.554	30.7	1.042	0.167	normoxia	1	0	0	Ucrit1hyp	59.9878
rypg	3	f	0.554	30.7	1.042	0.167	normoxia	1	0	0	Ucrit2hyp	
rypg	3	f	0.554	30.7	1.042	0.167	normoxia	1	0	0	Ucrit3hyp	
rypg	3	f	0.554	30.7	1.042	0.167	hypoxia	2	0.336666667	1	Ucrit1norm	75.215
rypg	3	f	0.554	30.7	1.042	0.167	hypoxia	2	0.336666667	1	Ucrit2norm	
rypg	3	f	0.554	30.7	1.042	0.167	hypoxia	2	0.336666667	1	Ucrit3norm	
rypg	3	f	0.554	30.7	1.042	0.167	hypoxia	2	0.336666667	1	Ucrit1hyp	59.9878
rypg	3	f	0.554	30.7	1.042	0.167	hypoxia	2	0.336666667	1	Ucrit2hyp	
rypg	3	f	0.554	30.7	1.042	0.167	hypoxia	2	0.336666667	1	Ucrit3hyp	
rypg	3	f	0.554	30.7	1.042	0.167	normoxia	2	0	0	Ucrit1norm	75.215
rypg	3	f	0.554	30.7	1.042	0.167	normoxia	2	0	0	Ucrit2norm	
rypg	3	f	0.554	30.7	1.042	0.167	normoxia	2	0	0	Ucrit3norm	
rypg	3	f	0.554	30.7	1.042	0.167	normoxia	2	0	0	Ucrit1hyp	59.9878
rypg	3	f	0.554	30.7	1.042	0.167	normoxia	2	0	0	Ucrit2hyp	
rypg	3	f	0.554	30.7	1.042	0.167	normoxia	2	0	0	Ucrit3hyp	
rypp	3	m	0.627	34.61		0.152	hypoxia	1	0.643333333	1	Ucrit1norm	78.59931
rypp	3	m	0.627	34.61		0.152	hypoxia	1	0.643333333	1	Ucrit2norm	
rypp	3	m	0.627	34.61		0.152	hypoxia	1	0.643333333	1	Ucrit3norm	
rypp	3	m	0.627	34.61		0.152	hypoxia	1	0.643333333	1	Ucrit1hyp	67.24723
rypp	3	m	0.627	34.61		0.152	hypoxia	1	0.643333333	1	Ucrit2hyp	
rypp	3	m	0.627	34.61		0.152	hypoxia	1	0.643333333	1	Ucrit3hyp	
rypp	3	m	0.627	34.61		0.152	normoxia	1	0	0	Ucrit1norm	78.59931
rypp	3	m	0.627	34.61		0.152	normoxia	1	0	0	Ucrit2norm	
rypp	3	m	0.627	34.61		0.152	normoxia	1	0	0	Ucrit3norm	
rypp	3	m	0.627	34.61		0.152	normoxia	1	0	0	Ucrit1hyp	67.24723
rypp	3	m	0.627	34.61		0.152	normoxia	1	0	0	Ucrit2hyp	
rypp	3	m	0.627	34.61		0.152	normoxia	1	0	0	Ucrit3hyp	
rypp	3	m	0.627	34.61		0.152	hypoxia	2	0	0	Ucrit1norm	78.59931
rypp	3	m	0.627	34.61		0.152	hypoxia	2	0	0	Ucrit2norm	
rypp	3	m	0.627	34.61		0.152	hypoxia	2	0	0	Ucrit3norm	

rypp	3	m	0.627	34.61		0.152	hypoxia	2	0	0	Ucrit1hyp	67.24723
rypp	3	m	0.627	34.61		0.152	hypoxia	2	0	0	Ucrit2hyp	
rypp	3	m	0.627	34.61		0.152	hypoxia	2	0	0	Ucrit3hyp	
rypp	3	m	0.627	34.61		0.152	normoxia	2	0.191666667	1	Ucrit1norm	78.59931
rypp	3	m	0.627	34.61		0.152	normoxia	2	0.191666667	1	Ucrit2norm	
rypp	3	m	0.627	34.61		0.152	normoxia	2	0.191666667	1	Ucrit3norm	
rypp	3	m	0.627	34.61		0.152	normoxia	2	0.191666667	1	Ucrit1hyp	67.24723
rypp	3	m	0.627	34.61		0.152	normoxia	2	0.191666667	1	Ucrit2hyp	
rypp	3	m	0.627	34.61		0.152	normoxia	2	0.191666667	1	Ucrit3hyp	
ryrg	1	m	0.437	29.87	0.962	0.142	normoxia	1	0	0	Ucrit1norm	73.65942
ryrg	1	m	0.437	29.87	0.962	0.142	normoxia	1	0	0	Ucrit2norm	
ryrg	1	m	0.437	29.87	0.962	0.142	normoxia	1	0	0	Ucrit3norm	
ryrg	1	m	0.437	29.87	0.962	0.142	normoxia	1	0	0	Ucrit1hyp	67.23737
ryrg	1	m	0.437	29.87	0.962	0.142	normoxia	1	0	0	Ucrit2hyp	
ryrg	1	m	0.437	29.87	0.962	0.142	normoxia	1	0	0	Ucrit3hyp	
ryrg	1	m	0.437	29.87	0.962	0.142	hypoxia	1	0	0	Ucrit1norm	73.65942
ryrg	1	m	0.437	29.87	0.962	0.142	hypoxia	1	0	0	Ucrit2norm	
ryrg	1	m	0.437	29.87	0.962	0.142	hypoxia	1	0	0	Ucrit3norm	
ryrg	1	m	0.437	29.87	0.962	0.142	hypoxia	1	0	0	Ucrit1hyp	67.23737
ryrg	1	m	0.437	29.87	0.962	0.142	hypoxia	1	0	0	Ucrit2hyp	
ryrg	1	m	0.437	29.87	0.962	0.142	hypoxia	1	0	0	Ucrit3hyp	
ryrg	1	m	0.437	29.87	0.962	0.142	normoxia	2	0	0	Ucrit1norm	73.65942
ryrg	1	m	0.437	29.87	0.962	0.142	normoxia	2	0	0	Ucrit2norm	
ryrg	1	m	0.437	29.87	0.962	0.142	normoxia	2	0	0	Ucrit3norm	
ryrg	1	m	0.437	29.87	0.962	0.142	normoxia	2	0	0	Ucrit1hyp	67.23737
ryrg	1	m	0.437	29.87	0.962	0.142	normoxia	2	0	0	Ucrit2hyp	
ryrg	1	m	0.437	29.87	0.962	0.142	normoxia	2	0	0	Ucrit3hyp	
ryrg	1	m	0.437	29.87	0.962	0.142	hypoxia	2	0.913333333	1	Ucrit1norm	73.65942
ryrg	1	m	0.437	29.87	0.962	0.142	hypoxia	2	0.913333333	1	Ucrit2norm	
ryrg	1	m	0.437	29.87	0.962	0.142	hypoxia	2	0.913333333	1	Ucrit3norm	
ryrg	1	m	0.437	29.87	0.962	0.142	hypoxia	2	0.913333333	1	Ucrit1hyp	67.23737
ryrg	1	m	0.437	29.87	0.962	0.142	hypoxia	2	0.913333333	1	Ucrit2hyp	
ryrg	1	m	0.437	29.87	0.962	0.142	hypoxia	2	0.913333333	1	Ucrit3hyp	

ryyg	4	f	0.722	34.23			normoxia	1	0	0	Ucrit1norm	74.92947
ryyg	4	f	0.722	34.23			normoxia	1	0	0	Ucrit2norm	72.97836
ryyg	4	f	0.722	34.23			normoxia	1	0	0	Ucrit3norm	77.42826
ryyg	4	f	0.722	34.23			normoxia	1	0	0	Ucrit1hyp	64.3524
ryyg	4	f	0.722	34.23			normoxia	1	0	0	Ucrit2hyp	64.52355
ryyg	4	f	0.722	34.23			normoxia	1	0	0	Ucrit3hyp	75.9906
ryyg	4	f	0.722	34.23			hypoxia	1	0	0	Ucrit1norm	74.92947
ryyg	4	f	0.722	34.23			hypoxia	1	0	0	Ucrit2norm	72.97836
ryyg	4	f	0.722	34.23			hypoxia	1	0	0	Ucrit3norm	77.42826
ryyg	4	f	0.722	34.23			hypoxia	1	0	0	Ucrit1hyp	64.3524
ryyg	4	f	0.722	34.23			hypoxia	1	0	0	Ucrit2hyp	64.52355
ryyg	4	f	0.722	34.23			hypoxia	1	0	0	Ucrit3hyp	75.9906
ryyg	4	f	0.722	34.23			normoxia	2	0	0	Ucrit1norm	74.92947
ryyg	4	f	0.722	34.23			normoxia	2	0	0	Ucrit2norm	72.97836
ryyg	4	f	0.722	34.23			normoxia	2	0	0	Ucrit3norm	77.42826
ryyg	4	f	0.722	34.23			normoxia	2	0	0	Ucrit1hyp	64.3524
ryyg	4	f	0.722	34.23			normoxia	2	0	0	Ucrit2hyp	64.52355
ryyg	4	f	0.722	34.23			normoxia	2	0	0	Ucrit3hyp	75.9906
ryyg	4	f	0.722	34.23			hypoxia	2	0	0	Ucrit1norm	74.92947
ryyg	4	f	0.722	34.23			hypoxia	2	0	0	Ucrit2norm	72.97836
ryyg	4	f	0.722	34.23			hypoxia	2	0	0	Ucrit3norm	77.42826
ryyg	4	f	0.722	34.23			hypoxia	2	0	0	Ucrit1hyp	64.3524
ryyg	4	f	0.722	34.23			hypoxia	2	0	0	Ucrit2hyp	64.52355
ryyg	4	f	0.722	34.23			hypoxia	2	0	0	Ucrit3hyp	75.9906
ryyp	2	f	0.441	30.42	0.611	0.153	hypoxia	1	0	0	Ucrit1norm	71.24364
ryyp	2	f	0.441	30.42	0.611	0.153	hypoxia	1	0	0	Ucrit2norm	
ryyp	2	f	0.441	30.42	0.611	0.153	hypoxia	1	0	0	Ucrit3norm	
ryyp	2	f	0.441	30.42	0.611	0.153	hypoxia	1	0	0	Ucrit1hyp	63.2736
ryyp	2	f	0.441	30.42	0.611	0.153	hypoxia	1	0	0	Ucrit2hyp	
ryyp	2	f	0.441	30.42	0.611	0.153	hypoxia	1	0	0	Ucrit3hyp	
ryyp	2	f	0.441	30.42	0.611	0.153	normoxia	1	0	0	Ucrit1norm	71.24364
ryyp	2	f	0.441	30.42	0.611	0.153	normoxia	1	0	0	Ucrit2norm	
ryyp	2	f	0.441	30.42	0.611	0.153	normoxia	1	0	0	Ucrit3norm	

ryyp	2	f	0.441	30.42	0.611	0.153	normoxia	1	0	0	Ucrit1hyp	63.2736
ryyp	2	f	0.441	30.42	0.611	0.153	normoxia	1	0	0	Ucrit2hyp	
ryyp	2	f	0.441	30.42	0.611	0.153	normoxia	1	0	0	Ucrit3hyp	
ryyp	2	f	0.441	30.42	0.611	0.153	hypoxia	2	0.871666667	1	Ucrit1norm	71.24364
ryyp	2	f	0.441	30.42	0.611	0.153	hypoxia	2	0.871666667	1	Ucrit2norm	
ryyp	2	f	0.441	30.42	0.611	0.153	hypoxia	2	0.871666667	1	Ucrit3norm	
ryyp	2	f	0.441	30.42	0.611	0.153	hypoxia	2	0.871666667	1	Ucrit1hyp	63.2736
ryyp	2	f	0.441	30.42	0.611	0.153	hypoxia	2	0.871666667	1	Ucrit2hyp	
ryyp	2	f	0.441	30.42	0.611	0.153	hypoxia	2	0.871666667	1	Ucrit3hyp	
ryyp	2	f	0.441	30.42	0.611	0.153	normoxia	2	0	0	Ucrit1norm	71.24364
ryyp	2	f	0.441	30.42	0.611	0.153	normoxia	2	0	0	Ucrit2norm	
ryyp	2	f	0.441	30.42	0.611	0.153	normoxia	2	0	0	Ucrit3norm	
ryyp	2	f	0.441	30.42	0.611	0.153	normoxia	2	0	0	Ucrit1hyp	63.2736
ryyp	2	f	0.441	30.42	0.611	0.153	normoxia	2	0	0	Ucrit2hyp	
ryyp	2	f	0.441	30.42	0.611	0.153	normoxia	2	0	0	Ucrit3hyp	
ryyy	4	f	0.637	33.85	1.561	0.257	normoxia	1	0	0	Ucrit1norm	81.3077
ryyy	4	f	0.637	33.85	1.561	0.257	normoxia	1	0	0	Ucrit2norm	84.72655
ryyy	4	f	0.637	33.85	1.561	0.257	normoxia	1	0	0	Ucrit3norm	87.4684
ryyy	4	f	0.637	33.85	1.561	0.257	normoxia	1	0	0	Ucrit1hyp	66.346
ryyy	4	f	0.637	33.85	1.561	0.257	normoxia	1	0	0	Ucrit2hyp	71.52505
ryyy	4	f	0.637	33.85	1.561	0.257	normoxia	1	0	0	Ucrit3hyp	75.4178
ryyy	4	f	0.637	33.85	1.561	0.257	hypoxia	1	0.463333333	1	Ucrit1norm	81.3077
ryyy	4	f	0.637	33.85	1.561	0.257	hypoxia	1	0.463333333	1	Ucrit2norm	84.72655
ryyy	4	f	0.637	33.85	1.561	0.257	hypoxia	1	0.463333333	1	Ucrit3norm	87.4684
ryyy	4	f	0.637	33.85	1.561	0.257	hypoxia	1	0.463333333	1	Ucrit1hyp	66.346
ryyy	4	f	0.637	33.85	1.561	0.257	hypoxia	1	0.463333333	1	Ucrit2hyp	71.52505
ryyy	4	f	0.637	33.85	1.561	0.257	hypoxia	1	0.463333333	1	Ucrit3hyp	75.4178
ryyy	4	f	0.637	33.85	1.561	0.257	normoxia	2	0	0	Ucrit1norm	81.3077
ryyy	4	f	0.637	33.85	1.561	0.257	normoxia	2	0	0	Ucrit2norm	84.72655
ryyy	4	f	0.637	33.85	1.561	0.257	normoxia	2	0	0	Ucrit3norm	87.4684
ryyy	4	f	0.637	33.85	1.561	0.257	normoxia	2	0	0	Ucrit1hyp	66.346
ryyy	4	f	0.637	33.85	1.561	0.257	normoxia	2	0	0	Ucrit2hyp	71.52505
ryyy	4	f	0.637	33.85	1.561	0.257	normoxia	2	0	0	Ucrit3hyp	75.4178

ryyy	4	f	0.637	33.85	1.561	0.257	hypoxia	2	0	0	Ucrit1norm	81.3077
ryyy	4	f	0.637	33.85	1.561	0.257	hypoxia	2	0	0	Ucrit2norm	84.72655
ryyy	4	f	0.637	33.85	1.561	0.257	hypoxia	2	0	0	Ucrit3norm	87.4684
ryyy	4	f	0.637	33.85	1.561	0.257	hypoxia	2	0	0	Ucrit1hyp	66.346
ryyy	4	f	0.637	33.85	1.561	0.257	hypoxia	2	0	0	Ucrit2hyp	71.52505
ryyy	4	f	0.637	33.85	1.561	0.257	hypoxia	2	0	0	Ucrit3hyp	75.4178
yggp	1	f	0.566	31.55	0.732	0.194	normoxia	1	0	0	Ucrit1norm	77.3606
yggp	1	f	0.566	31.55	0.732	0.194	normoxia	1	0	0	Ucrit2norm	
yggp	1	f	0.566	31.55	0.732	0.194	normoxia	1	0	0	Ucrit3norm	
yggp	1	f	0.566	31.55	0.732	0.194	normoxia	1	0	0	Ucrit1hyp	61.42785
yggp	1	f	0.566	31.55	0.732	0.194	normoxia	1	0	0	Ucrit2hyp	
yggp	1	f	0.566	31.55	0.732	0.194	normoxia	1	0	0	Ucrit3hyp	
yggp	1	f	0.566	31.55	0.732	0.194	hypoxia	1	0.886666667	1	Ucrit1norm	77.3606
yggp	1	f	0.566	31.55	0.732	0.194	hypoxia	1	0.886666667	1	Ucrit2norm	
yggp	1	f	0.566	31.55	0.732	0.194	hypoxia	1	0.886666667	1	Ucrit3norm	
yggp	1	f	0.566	31.55	0.732	0.194	hypoxia	1	0.886666667	1	Ucrit1hyp	61.42785
yggp	1	f	0.566	31.55	0.732	0.194	hypoxia	1	0.886666667	1	Ucrit2hyp	
yggp	1	f	0.566	31.55	0.732	0.194	hypoxia	1	0.886666667	1	Ucrit3hyp	
yggp	1	f	0.566	31.55	0.732	0.194	normoxia	2	0	0	Ucrit1norm	77.3606
yggp	1	f	0.566	31.55	0.732	0.194	normoxia	2	0	0	Ucrit2norm	
yggp	1	f	0.566	31.55	0.732	0.194	normoxia	2	0	0	Ucrit3norm	
yggp	1	f	0.566	31.55	0.732	0.194	normoxia	2	0	0	Ucrit1hyp	61.42785
yggp	1	f	0.566	31.55	0.732	0.194	normoxia	2	0	0	Ucrit2hyp	
yggp	1	f	0.566	31.55	0.732	0.194	normoxia	2	0	0	Ucrit3hyp	
yggp	1	f	0.566	31.55	0.732	0.194	hypoxia	2	0.835	1	Ucrit1norm	77.3606
yggp	1	f	0.566	31.55	0.732	0.194	hypoxia	2	0.835	1	Ucrit2norm	
yggp	1	f	0.566	31.55	0.732	0.194	hypoxia	2	0.835	1	Ucrit3norm	
yggp	1	f	0.566	31.55	0.732	0.194	hypoxia	2	0.835	1	Ucrit1hyp	61.42785
yggp	1	f	0.566	31.55	0.732	0.194	hypoxia	2	0.835	1	Ucrit2hyp	
yggp	1	f	0.566	31.55	0.732	0.194	hypoxia	2	0.835	1	Ucrit3hyp	
yggr	2	m	0.403	29.49	0.949	0.179	hypoxia	1	0	0	Ucrit1norm	88.70592
yggr	2	m	0.403	29.49	0.949	0.179	hypoxia	1	0	0	Ucrit2norm	
yggr	2	m	0.403	29.49	0.949	0.179	hypoxia	1	0	0	Ucrit3norm	

yggr	2	m	0.403	29.49	0.949	0.179	hypoxia	1	0	0	Ucrit1hyp	87.99816
yggr	2	m	0.403	29.49	0.949	0.179	hypoxia	1	0	0	Ucrit2hyp	
yggr	2	m	0.403	29.49	0.949	0.179	hypoxia	1	0	0	Ucrit3hyp	
yggr	2	m	0.403	29.49	0.949	0.179	normoxia	1	0	0	Ucrit1norm	88.70592
yggr	2	m	0.403	29.49	0.949	0.179	normoxia	1	0	0	Ucrit2norm	
yggr	2	m	0.403	29.49	0.949	0.179	normoxia	1	0	0	Ucrit3norm	
yggr	2	m	0.403	29.49	0.949	0.179	normoxia	1	0	0	Ucrit1hyp	87.99816
yggr	2	m	0.403	29.49	0.949	0.179	normoxia	1	0	0	Ucrit2hyp	
yggr	2	m	0.403	29.49	0.949	0.179	normoxia	1	0	0	Ucrit3hyp	
yggr	2	m	0.403	29.49	0.949	0.179	hypoxia	2	0	0	Ucrit1norm	88.70592
yggr	2	m	0.403	29.49	0.949	0.179	hypoxia	2	0	0	Ucrit2norm	
yggr	2	m	0.403	29.49	0.949	0.179	hypoxia	2	0	0	Ucrit3norm	
yggr	2	m	0.403	29.49	0.949	0.179	hypoxia	2	0	0	Ucrit1hyp	87.99816
yggr	2	m	0.403	29.49	0.949	0.179	hypoxia	2	0	0	Ucrit2hyp	
yggr	2	m	0.403	29.49	0.949	0.179	hypoxia	2	0	0	Ucrit3hyp	
yggr	2	m	0.403	29.49	0.949	0.179	normoxia	2	0	0	Ucrit1norm	88.70592
yggr	2	m	0.403	29.49	0.949	0.179	normoxia	2	0	0	Ucrit2norm	
yggr	2	m	0.403	29.49	0.949	0.179	normoxia	2	0	0	Ucrit3norm	
yggr	2	m	0.403	29.49	0.949	0.179	normoxia	2	0	0	Ucrit1hyp	87.99816
yggr	2	m	0.403	29.49	0.949	0.179	normoxia	2	0	0	Ucrit2hyp	
yggr	2	m	0.403	29.49	0.949	0.179	normoxia	2	0	0	Ucrit3hyp	
yggy	1	m	0.451	31.01	1.317	0.239	normoxia	1	0	0	Ucrit1norm	88.40951
yggy	1	m	0.451	31.01	1.317	0.239	normoxia	1	0	0	Ucrit2norm	
yggy	1	m	0.451	31.01	1.317	0.239	normoxia	1	0	0	Ucrit3norm	
yggy	1	m	0.451	31.01	1.317	0.239	normoxia	1	0	0	Ucrit1hyp	69.36937
yggy	1	m	0.451	31.01	1.317	0.239	normoxia	1	0	0	Ucrit2hyp	
yggy	1	m	0.451	31.01	1.317	0.239	normoxia	1	0	0	Ucrit3hyp	
yggy	1	m	0.451	31.01	1.317	0.239	hypoxia	1	0.71	1	Ucrit1norm	88.40951
yggy	1	m	0.451	31.01	1.317	0.239	hypoxia	1	0.71	1	Ucrit2norm	
yggy	1	m	0.451	31.01	1.317	0.239	hypoxia	1	0.71	1	Ucrit3norm	
yggy	1	m	0.451	31.01	1.317	0.239	hypoxia	1	0.71	1	Ucrit1hyp	69.36937
yggy	1	m	0.451	31.01	1.317	0.239	hypoxia	1	0.71	1	Ucrit2hyp	
yggy	1	m	0.451	31.01	1.317	0.239	hypoxia	1	0.71	1	Ucrit3hyp	

yggy	1	m	0.451	31.01	1.317	0.239	normoxia	2	0	0	Ucrit1norm	88.40951
yggy	1	m	0.451	31.01	1.317	0.239	normoxia	2	0	0	Ucrit2norm	
yggy	1	m	0.451	31.01	1.317	0.239	normoxia	2	0	0	Ucrit3norm	
yggy	1	m	0.451	31.01	1.317	0.239	normoxia	2	0	0	Ucrit1hyp	69.36937
yggy	1	m	0.451	31.01	1.317	0.239	normoxia	2	0	0	Ucrit2hyp	
yggy	1	m	0.451	31.01	1.317	0.239	normoxia	2	0	0	Ucrit3hyp	
yggy	1	m	0.451	31.01	1.317	0.239	hypoxia	2	0.626666667	1	Ucrit1norm	88.40951
yggy	1	m	0.451	31.01	1.317	0.239	hypoxia	2	0.626666667	1	Ucrit2norm	
yggy	1	m	0.451	31.01	1.317	0.239	hypoxia	2	0.626666667	1	Ucrit3norm	
yggy	1	m	0.451	31.01	1.317	0.239	hypoxia	2	0.626666667	1	Ucrit1hyp	69.36937
yggy	1	m	0.451	31.01	1.317	0.239	hypoxia	2	0.626666667	1	Ucrit2hyp	
yggy	1	m	0.451	31.01	1.317	0.239	hypoxia	2	0.626666667	1	Ucrit3hyp	
ygpg	1	f	0.529	34.5	0.648	0.164	normoxia	1	0	0	Ucrit1norm	80.6955
ygpg	1	f	0.529	34.5	0.648	0.164	normoxia	1	0	0	Ucrit2norm	
ygpg	1	f	0.529	34.5	0.648	0.164	normoxia	1	0	0	Ucrit3norm	
ygpg	1	f	0.529	34.5	0.648	0.164	normoxia	1	0	0	Ucrit1hyp	62.0655
ygpg	1	f	0.529	34.5	0.648	0.164	normoxia	1	0	0	Ucrit2hyp	
ygpg	1	f	0.529	34.5	0.648	0.164	normoxia	1	0	0	Ucrit3hyp	
ygpg	1	f	0.529	34.5	0.648	0.164	hypoxia	1	0.628333333	1	Ucrit1norm	80.6955
ygpg	1	f	0.529	34.5	0.648	0.164	hypoxia	1	0.628333333	1	Ucrit2norm	
ygpg	1	f	0.529	34.5	0.648	0.164	hypoxia	1	0.628333333	1	Ucrit3norm	
ygpg	1	f	0.529	34.5	0.648	0.164	hypoxia	1	0.628333333	1	Ucrit1hyp	62.0655
ygpg	1	f	0.529	34.5	0.648	0.164	hypoxia	1	0.628333333	1	Ucrit2hyp	
ygpg	1	f	0.529	34.5	0.648	0.164	hypoxia	1	0.628333333	1	Ucrit3hyp	
ygpg	1	f	0.529	34.5	0.648	0.164	normoxia	2	0	0	Ucrit1norm	80.6955
ygpg	1	f	0.529	34.5	0.648	0.164	normoxia	2	0	0	Ucrit2norm	
ygpg	1	f	0.529	34.5	0.648	0.164	normoxia	2	0	0	Ucrit3norm	
ygpg	1	f	0.529	34.5	0.648	0.164	normoxia	2	0	0	Ucrit1hyp	62.0655
ygpg	1	f	0.529	34.5	0.648	0.164	normoxia	2	0	0	Ucrit2hyp	
ygpg	1	f	0.529	34.5	0.648	0.164	normoxia	2	0	0	Ucrit3hyp	
ygpg	1	f	0.529	34.5	0.648	0.164	hypoxia	2	0	0	Ucrit1norm	80.6955
ygpg	1	f	0.529	34.5	0.648	0.164	hypoxia	2	0	0	Ucrit2norm	
ygpg	1	f	0.529	34.5	0.648	0.164	hypoxia	2	0	0	Ucrit3norm	

ygpg	1	f	0.529	34.5	0.648	0.164	hypoxia	2	0	0	Ucrit1hyp	62.0655
ygpg	1	f	0.529	34.5	0.648	0.164	hypoxia	2	0	0	Ucrit2hyp	
ygpg	1	f	0.529	34.5	0.648	0.164	hypoxia	2	0	0	Ucrit3hyp	
ygpy	1	m	0.42	30.76	0.919	0.147	normoxia	1	0	0	Ucrit1norm	84.40544
ygpy	1	m	0.42	30.76	0.919	0.147	normoxia	1	0	0	Ucrit2norm	
ygpy	1	m	0.42	30.76	0.919	0.147	normoxia	1	0	0	Ucrit3norm	
ygpy	1	m	0.42	30.76	0.919	0.147	normoxia	1	0	0	Ucrit1hyp	72.7474
ygpy	1	m	0.42	30.76	0.919	0.147	normoxia	1	0	0	Ucrit2hyp	
ygpy	1	m	0.42	30.76	0.919	0.147	normoxia	1	0	0	Ucrit3hyp	
ygpy	1	m	0.42	30.76	0.919	0.147	hypoxia	1	0.675	1	Ucrit1norm	84.40544
ygpy	1	m	0.42	30.76	0.919	0.147	hypoxia	1	0.675	1	Ucrit2norm	
ygpy	1	m	0.42	30.76	0.919	0.147	hypoxia	1	0.675	1	Ucrit3norm	
ygpy	1	m	0.42	30.76	0.919	0.147	hypoxia	1	0.675	1	Ucrit1hyp	72.7474
ygpy	1	m	0.42	30.76	0.919	0.147	hypoxia	1	0.675	1	Ucrit2hyp	
ygpy	1	m	0.42	30.76	0.919	0.147	hypoxia	1	0.675	1	Ucrit3hyp	
ygpy	1	m	0.42	30.76	0.919	0.147	normoxia	2	0	0	Ucrit1norm	84.40544
ygpy	1	m	0.42	30.76	0.919	0.147	normoxia	2	0	0	Ucrit2norm	
ygpy	1	m	0.42	30.76	0.919	0.147	normoxia	2	0	0	Ucrit3norm	
ygpy	1	m	0.42	30.76	0.919	0.147	normoxia	2	0	0	Ucrit1hyp	72.7474
ygpy	1	m	0.42	30.76	0.919	0.147	normoxia	2	0	0	Ucrit2hyp	
ygpy	1	m	0.42	30.76	0.919	0.147	normoxia	2	0	0	Ucrit3hyp	
ygpy	1	m	0.42	30.76	0.919	0.147	hypoxia	2	0.87	1	Ucrit1norm	84.40544
ygpy	1	m	0.42	30.76	0.919	0.147	hypoxia	2	0.87	1	Ucrit2norm	
ygpy	1	m	0.42	30.76	0.919	0.147	hypoxia	2	0.87	1	Ucrit3norm	
ygpy	1	m	0.42	30.76	0.919	0.147	hypoxia	2	0.87	1	Ucrit1hyp	72.7474
ygpy	1	m	0.42	30.76	0.919	0.147	hypoxia	2	0.87	1	Ucrit2hyp	
ygpy	1	m	0.42	30.76	0.919	0.147	hypoxia	2	0.87	1	Ucrit3hyp	
ygrg	2	f	0.679	33.98	0.948	0.171	hypoxia	1	0	0	Ucrit1norm	82.2316
ygrg	2	f	0.679	33.98	0.948	0.171	hypoxia	1	0	0	Ucrit2norm	
ygrg	2	f	0.679	33.98	0.948	0.171	hypoxia	1	0	0	Ucrit3norm	
ygrg	2	f	0.679	33.98	0.948	0.171	hypoxia	1	0	0	Ucrit1hyp	64.08628
ygrg	2	f	0.679	33.98	0.948	0.171	hypoxia	1	0	0	Ucrit2hyp	
ygrg	2	f	0.679	33.98	0.948	0.171	hypoxia	1	0	0	Ucrit3hyp	

ygrg	2	f	0.679	33.98	0.948	0.171	normoxia	1	0	0	Ucrit1norm	82.2316
ygrg	2	f	0.679	33.98	0.948	0.171	normoxia	1	0	0	Ucrit2norm	
ygrg	2	f	0.679	33.98	0.948	0.171	normoxia	1	0	0	Ucrit3norm	
ygrg	2	f	0.679	33.98	0.948	0.171	normoxia	1	0	0	Ucrit1hyp	64.08628
ygrg	2	f	0.679	33.98	0.948	0.171	normoxia	1	0	0	Ucrit2hyp	
ygrg	2	f	0.679	33.98	0.948	0.171	normoxia	1	0	0	Ucrit3hyp	
ygrg	2	f	0.679	33.98	0.948	0.171	hypoxia	2	0.886666667	1	Ucrit1norm	82.2316
ygrg	2	f	0.679	33.98	0.948	0.171	hypoxia	2	0.886666667	1	Ucrit2norm	
ygrg	2	f	0.679	33.98	0.948	0.171	hypoxia	2	0.886666667	1	Ucrit3norm	
ygrg	2	f	0.679	33.98	0.948	0.171	hypoxia	2	0.886666667	1	Ucrit1hyp	64.08628
ygrg	2	f	0.679	33.98	0.948	0.171	hypoxia	2	0.886666667	1	Ucrit2hyp	
ygrg	2	f	0.679	33.98	0.948	0.171	hypoxia	2	0.886666667	1	Ucrit3hyp	
ygrg	2	f	0.679	33.98	0.948	0.171	normoxia	2	0.546666667	1	Ucrit1norm	82.2316
ygrg	2	f	0.679	33.98	0.948	0.171	normoxia	2	0.546666667	1	Ucrit2norm	
ygrg	2	f	0.679	33.98	0.948	0.171	normoxia	2	0.546666667	1	Ucrit3norm	
ygrg	2	f	0.679	33.98	0.948	0.171	normoxia	2	0.546666667	1	Ucrit1hyp	64.08628
ygrg	2	f	0.679	33.98	0.948	0.171	normoxia	2	0.546666667	1	Ucrit2hyp	
ygrg	2	f	0.679	33.98	0.948	0.171	normoxia	2	0.546666667	1	Ucrit3hyp	
ygrp	1	f	0.619	32.32	0.656	0.174	normoxia	1	0	0	Ucrit1norm	72.62304
ygrp	1	f	0.619	32.32	0.656	0.174	normoxia	1	0	0	Ucrit2norm	
ygrp	1	f	0.619	32.32	0.656	0.174	normoxia	1	0	0	Ucrit3norm	
ygrp	1	f	0.619	32.32	0.656	0.174	normoxia	1	0	0	Ucrit1hyp	63.92896
ygrp	1	f	0.619	32.32	0.656	0.174	normoxia	1	0	0	Ucrit2hyp	
ygrp	1	f	0.619	32.32	0.656	0.174	normoxia	1	0	0	Ucrit3hyp	
ygrp	1	f	0.619	32.32	0.656	0.174	hypoxia	1	0	0	Ucrit1norm	72.62304
ygrp	1	f	0.619	32.32	0.656	0.174	hypoxia	1	0	0	Ucrit2norm	
ygrp	1	f	0.619	32.32	0.656	0.174	hypoxia	1	0	0	Ucrit3norm	
ygrp	1	f	0.619	32.32	0.656	0.174	hypoxia	1	0	0	Ucrit1hyp	63.92896
ygrp	1	f	0.619	32.32	0.656	0.174	hypoxia	1	0	0	Ucrit2hyp	
ygrp	1	f	0.619	32.32	0.656	0.174	hypoxia	1	0	0	Ucrit3hyp	
ygrp	1	f	0.619	32.32	0.656	0.174	normoxia	2	0	0	Ucrit1norm	72.62304
ygrp	1	f	0.619	32.32	0.656	0.174	normoxia	2	0	0	Ucrit2norm	
ygrp	1	f	0.619	32.32	0.656	0.174	normoxia	2	0	0	Ucrit3norm	

ygrp	1	f	0.619	32.32	0.656	0.174	normoxia	2	0	0	Ucrit1hyp	63.92896
ygrp	1	f	0.619	32.32	0.656	0.174	normoxia	2	0	0	Ucrit2hyp	
ygrp	1	f	0.619	32.32	0.656	0.174	normoxia	2	0	0	Ucrit3hyp	
ygrp	1	f	0.619	32.32	0.656	0.174	hypoxia	2	0.758333333	1	Ucrit1norm	72.62304
ygrp	1	f	0.619	32.32	0.656	0.174	hypoxia	2	0.758333333	1	Ucrit2norm	
ygrp	1	f	0.619	32.32	0.656	0.174	hypoxia	2	0.758333333	1	Ucrit3norm	
ygrp	1	f	0.619	32.32	0.656	0.174	hypoxia	2	0.758333333	1	Ucrit1hyp	63.92896
ygrp	1	f	0.619	32.32	0.656	0.174	hypoxia	2	0.758333333	1	Ucrit2hyp	
ygrp	1	f	0.619	32.32	0.656	0.174	hypoxia	2	0.758333333	1	Ucrit3hyp	
ygry	4	f	0.814	34.9			normoxia	1	0	0	Ucrit1norm	80.5492
ygry	4	f	0.814	34.9			normoxia	1	0	0	Ucrit2norm	77.2337
ygry	4	f	0.814	34.9			normoxia	1	0	0	Ucrit3norm	87.4594
ygry	4	f	0.814	34.9			normoxia	1	0	0	Ucrit1hyp	68.5785
ygry	4	f	0.814	34.9			normoxia	1	0	0	Ucrit2hyp	83.3063
ygry	4	f	0.814	34.9			normoxia	1	0	0	Ucrit3hyp	84.4231
ygry	4	f	0.814	34.9			hypoxia	1	0	0	Ucrit1norm	80.5492
ygry	4	f	0.814	34.9			hypoxia	1	0	0	Ucrit2norm	77.2337
ygry	4	f	0.814	34.9			hypoxia	1	0	0	Ucrit3norm	87.4594
ygry	4	f	0.814	34.9			hypoxia	1	0	0	Ucrit1hyp	68.5785
ygry	4	f	0.814	34.9			hypoxia	1	0	0	Ucrit2hyp	83.3063
ygry	4	f	0.814	34.9			hypoxia	1	0	0	Ucrit3hyp	84.4231
ygry	4	f	0.814	34.9			normoxia	2	0	0	Ucrit1norm	80.5492
ygry	4	f	0.814	34.9			normoxia	2	0	0	Ucrit2norm	77.2337
ygry	4	f	0.814	34.9			normoxia	2	0	0	Ucrit3norm	87.4594
ygry	4	f	0.814	34.9			normoxia	2	0	0	Ucrit1hyp	68.5785
ygry	4	f	0.814	34.9			normoxia	2	0	0	Ucrit2hyp	83.3063
ygry	4	f	0.814	34.9			normoxia	2	0	0	Ucrit3hyp	84.4231
ygry	4	f	0.814	34.9			hypoxia	2	0	0	Ucrit1norm	80.5492
ygry	4	f	0.814	34.9			hypoxia	2	0	0	Ucrit2norm	77.2337
ygry	4	f	0.814	34.9			hypoxia	2	0	0	Ucrit3norm	87.4594
ygry	4	f	0.814	34.9			hypoxia	2	0	0	Ucrit1hyp	68.5785
ygry	4	f	0.814	34.9			hypoxia	2	0	0	Ucrit2hyp	83.3063
ygry	4	f	0.814	34.9			hypoxia	2	0	0	Ucrit3hyp	84.4231

ygyg	2	f	0.59	31.08	1.223	0.198	hypoxia	1	0.716666667	1	Ucrit1norm	75.61764
ygyg	2	f	0.59	31.08	1.223	0.198	hypoxia	1	0.716666667	1	Ucrit2norm	
ygyg	2	f	0.59	31.08	1.223	0.198	hypoxia	1	0.716666667	1	Ucrit3norm	
ygyg	2	f	0.59	31.08	1.223	0.198	hypoxia	1	0.716666667	1	Ucrit1hyp	71.39076
ygyg	2	f	0.59	31.08	1.223	0.198	hypoxia	1	0.716666667	1	Ucrit2hyp	
ygyg	2	f	0.59	31.08	1.223	0.198	hypoxia	1	0.716666667	1	Ucrit3hyp	
ygyg	2	f	0.59	31.08	1.223	0.198	normoxia	1	0	0	Ucrit1norm	75.61764
ygyg	2	f	0.59	31.08	1.223	0.198	normoxia	1	0	0	Ucrit2norm	
ygyg	2	f	0.59	31.08	1.223	0.198	normoxia	1	0	0	Ucrit3norm	
ygyg	2	f	0.59	31.08	1.223	0.198	normoxia	1	0	0	Ucrit1hyp	71.39076
ygyg	2	f	0.59	31.08	1.223	0.198	normoxia	1	0	0	Ucrit2hyp	
ygyg	2	f	0.59	31.08	1.223	0.198	normoxia	1	0	0	Ucrit3hyp	
ygyg	2	f	0.59	31.08	1.223	0.198	hypoxia	2	0.823333333	1	Ucrit1norm	75.61764
ygyg	2	f	0.59	31.08	1.223	0.198	hypoxia	2	0.823333333	1	Ucrit2norm	
ygyg	2	f	0.59	31.08	1.223	0.198	hypoxia	2	0.823333333	1	Ucrit3norm	
ygyg	2	f	0.59	31.08	1.223	0.198	hypoxia	2	0.823333333	1	Ucrit1hyp	71.39076
ygyg	2	f	0.59	31.08	1.223	0.198	hypoxia	2	0.823333333	1	Ucrit2hyp	
ygyg	2	f	0.59	31.08	1.223	0.198	hypoxia	2	0.823333333	1	Ucrit3hyp	
ygyg	2	f	0.59	31.08	1.223	0.198	normoxia	2	0	0	Ucrit1norm	75.61764
ygyg	2	f	0.59	31.08	1.223	0.198	normoxia	2	0	0	Ucrit2norm	
ygyg	2	f	0.59	31.08	1.223	0.198	normoxia	2	0	0	Ucrit3norm	
ygyg	2	f	0.59	31.08	1.223	0.198	normoxia	2	0	0	Ucrit1hyp	71.39076
ygyg	2	f	0.59	31.08	1.223	0.198	normoxia	2	0	0	Ucrit2hyp	
ygyg	2	f	0.59	31.08	1.223	0.198	normoxia	2	0	0	Ucrit3hyp	
ygyp	4	m	0.595	35.14			normoxia	1	0	0	Ucrit1norm	75.41044
ygyp	4	m	0.595	35.14			normoxia	1	0	0	Ucrit2norm	80.40032
ygyp	4	m	0.595	35.14			normoxia	1	0	0	Ucrit3norm	79.4164
ygyp	4	m	0.595	35.14			normoxia	1	0	0	Ucrit1hyp	67.18768
ygyp	4	m	0.595	35.14			normoxia	1	0	0	Ucrit2hyp	67.89048
ygyp	4	m	0.595	35.14			normoxia	1	0	0	Ucrit3hyp	67.2931
ygyp	4	m	0.595	35.14			hypoxia	1	0.915	1	Ucrit1norm	75.41044
ygyp	4	m	0.595	35.14			hypoxia	1	0.915	1	Ucrit2norm	80.40032
ygyp	4	m	0.595	35.14			hypoxia	1	0.915	1	Ucrit3norm	79.4164

ygyp	4	m	0.595	35.14			hypoxia	1	0.915	1	Ucrit1hyp	67.18768
ygyp	4	m	0.595	35.14			hypoxia	1	0.915	1	Ucrit2hyp	67.89048
ygyp	4	m	0.595	35.14			hypoxia	1	0.915	1	Ucrit3hyp	67.2931
ygyp	4	m	0.595	35.14			normoxia	2	0	0	Ucrit1norm	75.41044
ygyp	4	m	0.595	35.14			normoxia	2	0	0	Ucrit2norm	80.40032
ygyp	4	m	0.595	35.14			normoxia	2	0	0	Ucrit3norm	79.4164
ygyp	4	m	0.595	35.14			normoxia	2	0	0	Ucrit1hyp	67.18768
ygyp	4	m	0.595	35.14			normoxia	2	0	0	Ucrit2hyp	67.89048
ygyp	4	m	0.595	35.14			normoxia	2	0	0	Ucrit3hyp	67.2931
ygyp	4	m	0.595	35.14			hypoxia	2	0.903333333	1	Ucrit1norm	75.41044
ygyp	4	m	0.595	35.14			hypoxia	2	0.903333333	1	Ucrit2norm	80.40032
ygyp	4	m	0.595	35.14			hypoxia	2	0.903333333	1	Ucrit3norm	79.4164
ygyp	4	m	0.595	35.14			hypoxia	2	0.903333333	1	Ucrit1hyp	67.18768
ygyp	4	m	0.595	35.14			hypoxia	2	0.903333333	1	Ucrit2hyp	67.89048
ygyp	4	m	0.595	35.14			hypoxia	2	0.903333333	1	Ucrit3hyp	67.2931
ypgg	3	m	0.319	29.68	1.015	0.172	hypoxia	1	0	0	Ucrit1norm	99.90288
ypgg	3	m	0.319	29.68	1.015	0.172	hypoxia	1	0	0	Ucrit2norm	
ypgg	3	m	0.319	29.68	1.015	0.172	hypoxia	1	0	0	Ucrit3norm	
ypgg	3	m	0.319	29.68	1.015	0.172	hypoxia	1	0	0	Ucrit1hyp	75.41688
ypgg	3	m	0.319	29.68	1.015	0.172	hypoxia	1	0	0	Ucrit2hyp	
ypgg	3	m	0.319	29.68	1.015	0.172	hypoxia	1	0	0	Ucrit3hyp	
ypgg	3	m	0.319	29.68	1.015	0.172	normoxia	1	0	0	Ucrit1norm	99.90288
ypgg	3	m	0.319	29.68	1.015	0.172	normoxia	1	0	0	Ucrit2norm	
ypgg	3	m	0.319	29.68	1.015	0.172	normoxia	1	0	0	Ucrit3norm	
ypgg	3	m	0.319	29.68	1.015	0.172	normoxia	1	0	0	Ucrit1hyp	75.41688
ypgg	3	m	0.319	29.68	1.015	0.172	normoxia	1	0	0	Ucrit2hyp	
ypgg	3	m	0.319	29.68	1.015	0.172	normoxia	1	0	0	Ucrit3hyp	
ypgg	3	m	0.319	29.68	1.015	0.172	hypoxia	2	0	0	Ucrit1norm	99.90288
ypgg	3	m	0.319	29.68	1.015	0.172	hypoxia	2	0	0	Ucrit2norm	
ypgg	3	m	0.319	29.68	1.015	0.172	hypoxia	2	0	0	Ucrit3norm	
ypgg	3	m	0.319	29.68	1.015	0.172	hypoxia	2	0	0	Ucrit1hyp	75.41688
ypgg	3	m	0.319	29.68	1.015	0.172	hypoxia	2	0	0	Ucrit2hyp	
ypgg	3	m	0.319	29.68	1.015	0.172	hypoxia	2	0	0	Ucrit3hyp	

ypgg	3	m	0.319	29.68	1.015	0.172	normoxia	2	0	0	Ucrit1norm	99.90288
ypgg	3	m	0.319	29.68	1.015	0.172	normoxia	2	0	0	Ucrit2norm	
ypgg	3	m	0.319	29.68	1.015	0.172	normoxia	2	0	0	Ucrit3norm	
ypgg	3	m	0.319	29.68	1.015	0.172	normoxia	2	0	0	Ucrit1hyp	75.41688
ypgg	3	m	0.319	29.68	1.015	0.172	normoxia	2	0	0	Ucrit2hyp	
ypgg	3	m	0.319	29.68	1.015	0.172	normoxia	2	0	0	Ucrit3hyp	
ypgp	4	f	0.578	32.76			normoxia	1	0	0	Ucrit1norm	94.41432
ypgp	4	f	0.578	32.76			normoxia	1	0	0	Ucrit2norm	82.35864
ypgp	4	f	0.578	32.76			normoxia	1	0	0	Ucrit3norm	80.45856
ypgp	4	f	0.578	32.76			normoxia	1	0	0	Ucrit1hyp	70.40124
ypgp	4	f	0.578	32.76			normoxia	1	0	0	Ucrit2hyp	68.23908
ypgp	4	f	0.578	32.76			normoxia	1	0	0	Ucrit3hyp	72.072
ypgp	4	f	0.578	32.76			hypoxia	1	0.873333333	1	Ucrit1norm	94.41432
ypgp	4	f	0.578	32.76			hypoxia	1	0.873333333	1	Ucrit2norm	82.35864
ypgp	4	f	0.578	32.76			hypoxia	1	0.873333333	1	Ucrit3norm	80.45856
ypgp	4	f	0.578	32.76			hypoxia	1	0.873333333	1	Ucrit1hyp	70.40124
ypgp	4	f	0.578	32.76			hypoxia	1	0.873333333	1	Ucrit2hyp	68.23908
ypgp	4	f	0.578	32.76			hypoxia	1	0.873333333	1	Ucrit3hyp	72.072
ypgp	4	f	0.578	32.76			normoxia	2	0	0	Ucrit1norm	94.41432
ypgp	4	f	0.578	32.76			normoxia	2	0	0	Ucrit2norm	82.35864
ypgp	4	f	0.578	32.76			normoxia	2	0	0	Ucrit3norm	80.45856
ypgp	4	f	0.578	32.76			normoxia	2	0	0	Ucrit1hyp	70.40124
ypgp	4	f	0.578	32.76			normoxia	2	0	0	Ucrit2hyp	68.23908
ypgp	4	f	0.578	32.76			normoxia	2	0	0	Ucrit3hyp	72.072
ypgp	4	f	0.578	32.76			hypoxia	2	0	0	Ucrit1norm	94.41432
ypgp	4	f	0.578	32.76			hypoxia	2	0	0	Ucrit2norm	82.35864
ypgp	4	f	0.578	32.76			hypoxia	2	0	0	Ucrit3norm	80.45856
ypgp	4	f	0.578	32.76			hypoxia	2	0	0	Ucrit1hyp	70.40124
ypgp	4	f	0.578	32.76			hypoxia	2	0	0	Ucrit2hyp	68.23908
ypgp	4	f	0.578	32.76			hypoxia	2	0	0	Ucrit3hyp	72.072
yppr	3	f	0.581	31.72	0.929	0.152	hypoxia	1	0.785	1	Ucrit1norm	72.03612
yppr	3	f	0.581	31.72	0.929	0.152	hypoxia	1	0.785	1	Ucrit2norm	
yppr	3	f	0.581	31.72	0.929	0.152	hypoxia	1	0.785	1	Ucrit3norm	

yppr	3	f	0.581	31.72	0.929	0.152	hypoxia	1	0.785	1	Ucrit1hyp	61.34648
yppr	3	f	0.581	31.72	0.929	0.152	hypoxia	1	0.785	1	Ucrit2hyp	
yppr	3	f	0.581	31.72	0.929	0.152	hypoxia	1	0.785	1	Ucrit3hyp	
yppr	3	f	0.581	31.72	0.929	0.152	normoxia	1	0	0	Ucrit1norm	72.03612
yppr	3	f	0.581	31.72	0.929	0.152	normoxia	1	0	0	Ucrit2norm	
yppr	3	f	0.581	31.72	0.929	0.152	normoxia	1	0	0	Ucrit3norm	
yppr	3	f	0.581	31.72	0.929	0.152	normoxia	1	0	0	Ucrit1hyp	61.34648
yppr	3	f	0.581	31.72	0.929	0.152	normoxia	1	0	0	Ucrit2hyp	
yppr	3	f	0.581	31.72	0.929	0.152	normoxia	1	0	0	Ucrit3hyp	
yppr	3	f	0.581	31.72	0.929	0.152	hypoxia	2	0.823333333	1	Ucrit1norm	72.03612
yppr	3	f	0.581	31.72	0.929	0.152	hypoxia	2	0.823333333	1	Ucrit2norm	
yppr	3	f	0.581	31.72	0.929	0.152	hypoxia	2	0.823333333	1	Ucrit3norm	
yppr	3	f	0.581	31.72	0.929	0.152	hypoxia	2	0.823333333	1	Ucrit1hyp	61.34648
yppr	3	f	0.581	31.72	0.929	0.152	hypoxia	2	0.823333333	1	Ucrit2hyp	
yppr	3	f	0.581	31.72	0.929	0.152	hypoxia	2	0.823333333	1	Ucrit3hyp	
yppr	3	f	0.581	31.72	0.929	0.152	normoxia	2	0	0	Ucrit1norm	72.03612
yppr	3	f	0.581	31.72	0.929	0.152	normoxia	2	0	0	Ucrit2norm	
yppr	3	f	0.581	31.72	0.929	0.152	normoxia	2	0	0	Ucrit3norm	
yppr	3	f	0.581	31.72	0.929	0.152	normoxia	2	0	0	Ucrit1hyp	61.34648
yppr	3	f	0.581	31.72	0.929	0.152	normoxia	2	0	0	Ucrit2hyp	
yppr	3	f	0.581	31.72	0.929	0.152	normoxia	2	0	0	Ucrit3hyp	
yprr	1	m	0.518	32.64	1.013	0.219	normoxia	1	0	0	Ucrit1norm	72.1344
yprr	1	m	0.518	32.64	1.013	0.219	normoxia	1	0	0	Ucrit2norm	
yprr	1	m	0.518	32.64	1.013	0.219	normoxia	1	0	0	Ucrit3norm	
yprr	1	m	0.518	32.64	1.013	0.219	normoxia	1	0	0	Ucrit1hyp	61.6896
yprr	1	m	0.518	32.64	1.013	0.219	normoxia	1	0	0	Ucrit2hyp	
yprr	1	m	0.518	32.64	1.013	0.219	normoxia	1	0	0	Ucrit3hyp	
yprr	1	m	0.518	32.64	1.013	0.219	hypoxia	1	0.666666667	1	Ucrit1norm	72.1344
yprr	1	m	0.518	32.64	1.013	0.219	hypoxia	1	0.666666667	1	Ucrit2norm	
yprr	1	m	0.518	32.64	1.013	0.219	hypoxia	1	0.666666667	1	Ucrit3norm	
yprr	1	m	0.518	32.64	1.013	0.219	hypoxia	1	0.666666667	1	Ucrit1hyp	61.6896
yprr	1	m	0.518	32.64	1.013	0.219	hypoxia	1	0.666666667	1	Ucrit2hyp	
yprr	1	m	0.518	32.64	1.013	0.219	hypoxia	1	0.666666667	1	Ucrit3hyp	

yprr	1	m	0.518	32.64	1.013	0.219	normoxia	2	0	0	Ucrit1norm	72.1344
yprr	1	m	0.518	32.64	1.013	0.219	normoxia	2	0	0	Ucrit2norm	
yprr	1	m	0.518	32.64	1.013	0.219	normoxia	2	0	0	Ucrit3norm	
yprr	1	m	0.518	32.64	1.013	0.219	normoxia	2	0	0	Ucrit1hyp	61.6896
yprr	1	m	0.518	32.64	1.013	0.219	normoxia	2	0	0	Ucrit2hyp	
yprr	1	m	0.518	32.64	1.013	0.219	normoxia	2	0	0	Ucrit3hyp	
yprr	1	m	0.518	32.64	1.013	0.219	hypoxia	2	0	0	Ucrit1norm	72.1344
yprr	1	m	0.518	32.64	1.013	0.219	hypoxia	2	0	0	Ucrit2norm	
yprr	1	m	0.518	32.64	1.013	0.219	hypoxia	2	0	0	Ucrit3norm	
yprr	1	m	0.518	32.64	1.013	0.219	hypoxia	2	0	0	Ucrit1hyp	61.6896
yprr	1	m	0.518	32.64	1.013	0.219	hypoxia	2	0	0	Ucrit2hyp	
yprr	1	m	0.518	32.64	1.013	0.219	hypoxia	2	0	0	Ucrit3hyp	
ypyg	3	m	0.788	36.97	1.323	0.256	hypoxia	1	0	0	Ucrit1norm	80.89036
ypyg	3	m	0.788	36.97	1.323	0.256	hypoxia	1	0	0	Ucrit2norm	
ypyg	3	m	0.788	36.97	1.323	0.256	hypoxia	1	0	0	Ucrit3norm	
ypyg	3	m	0.788	36.97	1.323	0.256	hypoxia	1	0	0	Ucrit1hyp	71.57392
ypyg	3	m	0.788	36.97	1.323	0.256	hypoxia	1	0	0	Ucrit2hyp	
ypyg	3	m	0.788	36.97	1.323	0.256	hypoxia	1	0	0	Ucrit3hyp	
ypyg	3	m	0.788	36.97	1.323	0.256	normoxia	1	0	0	Ucrit1norm	80.89036
ypyg	3	m	0.788	36.97	1.323	0.256	normoxia	1	0	0	Ucrit2norm	
ypyg	3	m	0.788	36.97	1.323	0.256	normoxia	1	0	0	Ucrit3norm	
ypyg	3	m	0.788	36.97	1.323	0.256	normoxia	1	0	0	Ucrit1hyp	71.57392
ypyg	3	m	0.788	36.97	1.323	0.256	normoxia	1	0	0	Ucrit2hyp	
ypyg	3	m	0.788	36.97	1.323	0.256	normoxia	1	0	0	Ucrit3hyp	
ypyg	3	m	0.788	36.97	1.323	0.256	hypoxia	2	0	0	Ucrit1norm	80.89036
ypyg	3	m	0.788	36.97	1.323	0.256	hypoxia	2	0	0	Ucrit2norm	
ypyg	3	m	0.788	36.97	1.323	0.256	hypoxia	2	0	0	Ucrit3norm	
ypyg	3	m	0.788	36.97	1.323	0.256	hypoxia	2	0	0	Ucrit1hyp	71.57392
ypyg	3	m	0.788	36.97	1.323	0.256	hypoxia	2	0	0	Ucrit2hyp	
ypyg	3	m	0.788	36.97	1.323	0.256	hypoxia	2	0	0	Ucrit3hyp	
ypyg	3	m	0.788	36.97	1.323	0.256	normoxia	2	0	0	Ucrit1norm	80.89036
ypyg	3	m	0.788	36.97	1.323	0.256	normoxia	2	0	0	Ucrit2norm	
ypyg	3	m	0.788	36.97	1.323	0.256	normoxia	2	0	0	Ucrit3norm	

ypyg	3	m	0.788	36.97	1.323	0.256	normoxia	2	0	0	Ucrit1hyp	71.57392
ypyg	3	m	0.788	36.97	1.323	0.256	normoxia	2	0	0	Ucrit2hyp	
ypyg	3	m	0.788	36.97	1.323	0.256	normoxia	2	0	0	Ucrit3hyp	
ypyp	1	f	0.52	30.16	1.277	0.247	normoxia	1	0	0	Ucrit1norm	72.14272
ypyp	1	f	0.52	30.16	1.277	0.247	normoxia	1	0	0	Ucrit2norm	
ypyp	1	f	0.52	30.16	1.277	0.247	normoxia	1	0	0	Ucrit3norm	
ypyp	1	f	0.52	30.16	1.277	0.247	normoxia	1	0	0	Ucrit1hyp	59.4152
ypyp	1	f	0.52	30.16	1.277	0.247	normoxia	1	0	0	Ucrit2hyp	
ypyp	1	f	0.52	30.16	1.277	0.247	normoxia	1	0	0	Ucrit3hyp	
ypyp	1	f	0.52	30.16	1.277	0.247	hypoxia	1	0.888333333	1	Ucrit1norm	72.14272
ypyp	1	f	0.52	30.16	1.277	0.247	hypoxia	1	0.888333333	1	Ucrit2norm	
ypyp	1	f	0.52	30.16	1.277	0.247	hypoxia	1	0.888333333	1	Ucrit3norm	
ypyp	1	f	0.52	30.16	1.277	0.247	hypoxia	1	0.888333333	1	Ucrit1hyp	59.4152
ypyp	1	f	0.52	30.16	1.277	0.247	hypoxia	1	0.888333333	1	Ucrit2hyp	
ypyp	1	f	0.52	30.16	1.277	0.247	hypoxia	1	0.888333333	1	Ucrit3hyp	
ypyp	1	f	0.52	30.16	1.277	0.247	normoxia	2	0	0	Ucrit1norm	72.14272
ypyp	1	f	0.52	30.16	1.277	0.247	normoxia	2	0	0	Ucrit2norm	
ypyp	1	f	0.52	30.16	1.277	0.247	normoxia	2	0	0	Ucrit3norm	
ypyp	1	f	0.52	30.16	1.277	0.247	normoxia	2	0	0	Ucrit1hyp	59.4152
ypyp	1	f	0.52	30.16	1.277	0.247	normoxia	2	0	0	Ucrit2hyp	
ypyp	1	f	0.52	30.16	1.277	0.247	normoxia	2	0	0	Ucrit3hyp	
ypyp	1	f	0.52	30.16	1.277	0.247	hypoxia	2	0	0	Ucrit1norm	72.14272
ypyp	1	f	0.52	30.16	1.277	0.247	hypoxia	2	0	0	Ucrit2norm	
ypyp	1	f	0.52	30.16	1.277	0.247	hypoxia	2	0	0	Ucrit3norm	
ypyp	1	f	0.52	30.16	1.277	0.247	hypoxia	2	0	0	Ucrit1hyp	59.4152
ypyp	1	f	0.52	30.16	1.277	0.247	hypoxia	2	0	0	Ucrit2hyp	
ypyp	1	f	0.52	30.16	1.277	0.247	hypoxia	2	0	0	Ucrit3hyp	
ypyr	3	m	0.485	30.81	0.949	0.183	hypoxia	1	0	0	Ucrit1norm	86.17557
ypyr	3	m	0.485	30.81	0.949	0.183	hypoxia	1	0	0	Ucrit2norm	
ypyr	3	m	0.485	30.81	0.949	0.183	hypoxia	1	0	0	Ucrit3norm	
ypyr	3	m	0.485	30.81	0.949	0.183	hypoxia	1	0	0	Ucrit1hyp	70.52409
ypyr	3	m	0.485	30.81	0.949	0.183	hypoxia	1	0	0	Ucrit2hyp	
ypyr	3	m	0.485	30.81	0.949	0.183	hypoxia	1	0	0	Ucrit3hyp	

ypyr	3	m	0.485	30.81	0.949	0.183	normoxia	1	0	0	Ucrit1norm	86.17557
ypyr	3	m	0.485	30.81	0.949	0.183	normoxia	1	0	0	Ucrit2norm	
ypyr	3	m	0.485	30.81	0.949	0.183	normoxia	1	0	0	Ucrit3norm	
ypyr	3	m	0.485	30.81	0.949	0.183	normoxia	1	0	0	Ucrit1hyp	70.52409
ypyr	3	m	0.485	30.81	0.949	0.183	normoxia	1	0	0	Ucrit2hyp	
ypyr	3	m	0.485	30.81	0.949	0.183	normoxia	1	0	0	Ucrit3hyp	
ypyr	3	m	0.485	30.81	0.949	0.183	hypoxia	2	0	0	Ucrit1norm	86.17557
ypyr	3	m	0.485	30.81	0.949	0.183	hypoxia	2	0	0	Ucrit2norm	
ypyr	3	m	0.485	30.81	0.949	0.183	hypoxia	2	0	0	Ucrit3norm	
ypyr	3	m	0.485	30.81	0.949	0.183	hypoxia	2	0	0	Ucrit1hyp	70.52409
ypyr	3	m	0.485	30.81	0.949	0.183	hypoxia	2	0	0	Ucrit2hyp	
ypyr	3	m	0.485	30.81	0.949	0.183	hypoxia	2	0	0	Ucrit3hyp	
ypyr	3	m	0.485	30.81	0.949	0.183	normoxia	2	0	0	Ucrit1norm	86.17557
ypyr	3	m	0.485	30.81	0.949	0.183	normoxia	2	0	0	Ucrit2norm	
ypyr	3	m	0.485	30.81	0.949	0.183	normoxia	2	0	0	Ucrit3norm	
ypyr	3	m	0.485	30.81	0.949	0.183	normoxia	2	0	0	Ucrit1hyp	70.52409
ypyr	3	m	0.485	30.81	0.949	0.183	normoxia	2	0	0	Ucrit2hyp	
ypyr	3	m	0.485	30.81	0.949	0.183	normoxia	2	0	0	Ucrit3hyp	
yrgg	4	f	0.623	35.18	1.106	0.159	normoxia	1	0	0	Ucrit1norm	87.14086
yrgg	4	f	0.623	35.18	1.106	0.159	normoxia	1	0	0	Ucrit2norm	78.9791
yrgg	4	f	0.623	35.18	1.106	0.159	normoxia	1	0	0	Ucrit3norm	80.63256
yrgg	4	f	0.623	35.18	1.106	0.159	normoxia	1	0	0	Ucrit1hyp	62.51486
yrgg	4	f	0.623	35.18	1.106	0.159	normoxia	1	0	0	Ucrit2hyp	64.52012
yrgg	4	f	0.623	35.18	1.106	0.159	normoxia	1	0	0	Ucrit3hyp	67.75668
yrgg	4	f	0.623	35.18	1.106	0.159	hypoxia	1	0	0	Ucrit1norm	87.14086
yrgg	4	f	0.623	35.18	1.106	0.159	hypoxia	1	0	0	Ucrit2norm	78.9791
yrgg	4	f	0.623	35.18	1.106	0.159	hypoxia	1	0	0	Ucrit3norm	80.63256
yrgg	4	f	0.623	35.18	1.106	0.159	hypoxia	1	0	0	Ucrit1hyp	62.51486
yrgg	4	f	0.623	35.18	1.106	0.159	hypoxia	1	0	0	Ucrit2hyp	64.52012
yrgg	4	f	0.623	35.18	1.106	0.159	hypoxia	1	0	0	Ucrit3hyp	67.75668
yrgg	4	f	0.623	35.18	1.106	0.159	normoxia	2	0	0	Ucrit1norm	87.14086
yrgg	4	f	0.623	35.18	1.106	0.159	normoxia	2	0	0	Ucrit2norm	78.9791
yrgg	4	f	0.623	35.18	1.106	0.159	normoxia	2	0	0	Ucrit3norm	80.63256

yrgg	4	f	0.623	35.18	1.106	0.159	normoxia	2	0	0	Ucrit1hyp	62.51486
yrgg	4	f	0.623	35.18	1.106	0.159	normoxia	2	0	0	Ucrit2hyp	64.52012
yrgg	4	f	0.623	35.18	1.106	0.159	normoxia	2	0	0	Ucrit3hyp	67.75668
yrgg	4	f	0.623	35.18	1.106	0.159	hypoxia	2	0.771666667	1	Ucrit1norm	87.14086
yrgg	4	f	0.623	35.18	1.106	0.159	hypoxia	2	0.771666667	1	Ucrit2norm	78.9791
yrgg	4	f	0.623	35.18	1.106	0.159	hypoxia	2	0.771666667	1	Ucrit3norm	80.63256
yrgg	4	f	0.623	35.18	1.106	0.159	hypoxia	2	0.771666667	1	Ucrit1hyp	62.51486
yrgg	4	f	0.623	35.18	1.106	0.159	hypoxia	2	0.771666667	1	Ucrit2hyp	64.52012
yrgg	4	f	0.623	35.18	1.106	0.159	hypoxia	2	0.771666667	1	Ucrit3hyp	67.75668
yrgp	1	f	0.594	32.25	0.836	0.199	normoxia	1	0	0	Ucrit1norm	74.14275
yrgp	1	f	0.594	32.25	0.836	0.199	normoxia	1	0	0	Ucrit2norm	
yrgp	1	f	0.594	32.25	0.836	0.199	normoxia	1	0	0	Ucrit3norm	
yrgp	1	f	0.594	32.25	0.836	0.199	normoxia	1	0	0	Ucrit1hyp	71.11125
yrgp	1	f	0.594	32.25	0.836	0.199	normoxia	1	0	0	Ucrit2hyp	
yrgp	1	f	0.594	32.25	0.836	0.199	normoxia	1	0	0	Ucrit3hyp	
yrgp	1	f	0.594	32.25	0.836	0.199	hypoxia	1	0.735	1	Ucrit1norm	74.14275
yrgp	1	f	0.594	32.25	0.836	0.199	hypoxia	1	0.735	1	Ucrit2norm	
yrgp	1	f	0.594	32.25	0.836	0.199	hypoxia	1	0.735	1	Ucrit3norm	
yrgp	1	f	0.594	32.25	0.836	0.199	hypoxia	1	0.735	1	Ucrit1hyp	71.11125
yrgp	1	f	0.594	32.25	0.836	0.199	hypoxia	1	0.735	1	Ucrit2hyp	
yrgp	1	f	0.594	32.25	0.836	0.199	hypoxia	1	0.735	1	Ucrit3hyp	
yrgp	1	f	0.594	32.25	0.836	0.199	normoxia	2	0	0	Ucrit1norm	74.14275
yrgp	1	f	0.594	32.25	0.836	0.199	normoxia	2	0	0	Ucrit2norm	
yrgp	1	f	0.594	32.25	0.836	0.199	normoxia	2	0	0	Ucrit3norm	
yrgp	1	f	0.594	32.25	0.836	0.199	normoxia	2	0	0	Ucrit1hyp	71.11125
yrgp	1	f	0.594	32.25	0.836	0.199	normoxia	2	0	0	Ucrit2hyp	
yrgp	1	f	0.594	32.25	0.836	0.199	normoxia	2	0	0	Ucrit3hyp	
yrgp	1	f	0.594	32.25	0.836	0.199	hypoxia	2	0	0	Ucrit1norm	74.14275
yrgp	1	f	0.594	32.25	0.836	0.199	hypoxia	2	0	0	Ucrit2norm	
yrgp	1	f	0.594	32.25	0.836	0.199	hypoxia	2	0	0	Ucrit3norm	
yrgp	1	f	0.594	32.25	0.836	0.199	hypoxia	2	0	0	Ucrit1hyp	71.11125
yrgp	1	f	0.594	32.25	0.836	0.199	hypoxia	2	0	0	Ucrit2hyp	
yrgp	1	f	0.594	32.25	0.836	0.199	hypoxia	2	0	0	Ucrit3hyp	

yrpg	3	m	0.636	34.47			hypoxia	1	0	0	Ucrit1norm	79.55676
yrpg	3	m	0.636	34.47			hypoxia	1	0	0	Ucrit2norm	
yrpg	3	m	0.636	34.47			hypoxia	1	0	0	Ucrit3norm	
yrpg	3	m	0.636	34.47			hypoxia	1	0	0	Ucrit1hyp	
yrpg	3	m	0.636	34.47			hypoxia	1	0	0	Ucrit2hyp	
yrpg	3	m	0.636	34.47			hypoxia	1	0	0	Ucrit3hyp	
yrpg	3	m	0.636	34.47			normoxia	1	0	0	Ucrit1norm	79.55676
yrpg	3	m	0.636	34.47			normoxia	1	0	0	Ucrit2norm	
yrpg	3	m	0.636	34.47			normoxia	1	0	0	Ucrit3norm	
yrpg	3	m	0.636	34.47			normoxia	1	0	0	Ucrit1hyp	
yrpg	3	m	0.636	34.47			normoxia	1	0	0	Ucrit2hyp	
yrpg	3	m	0.636	34.47			normoxia	1	0	0	Ucrit3hyp	
yrpg	3	m	0.636	34.47			hypoxia	2	0.533333333	1	Ucrit1norm	79.55676
yrpg	3	m	0.636	34.47			hypoxia	2	0.533333333	1	Ucrit2norm	
yrpg	3	m	0.636	34.47			hypoxia	2	0.533333333	1	Ucrit3norm	
yrpg	3	m	0.636	34.47			hypoxia	2	0.533333333	1	Ucrit1hyp	
yrpg	3	m	0.636	34.47			hypoxia	2	0.533333333	1	Ucrit2hyp	
yrpg	3	m	0.636	34.47			hypoxia	2	0.533333333	1	Ucrit3hyp	
yrpg	3	m	0.636	34.47			normoxia	2	0	0	Ucrit1norm	79.55676
yrpg	3	m	0.636	34.47			normoxia	2	0	0	Ucrit2norm	
yrpg	3	m	0.636	34.47			normoxia	2	0	0	Ucrit3norm	
yrpg	3	m	0.636	34.47			normoxia	2	0	0	Ucrit1hyp	
yrpg	3	m	0.636	34.47			normoxia	2	0	0	Ucrit2hyp	
yrpg	3	m	0.636	34.47			normoxia	2	0	0	Ucrit3hyp	
yrpp	2	f	0.6	32.16	1.066	0.181	hypoxia	1	0.876666667	1	Ucrit1norm	73.87152
yrpp	2	f	0.6	32.16	1.066	0.181	hypoxia	1	0.876666667	1	Ucrit2norm	
yrpp	2	f	0.6	32.16	1.066	0.181	hypoxia	1	0.876666667	1	Ucrit3norm	
yrpp	2	f	0.6	32.16	1.066	0.181	hypoxia	1	0.876666667	1	Ucrit1hyp	56.08704
yrpp	2	f	0.6	32.16	1.066	0.181	hypoxia	1	0.876666667	1	Ucrit2hyp	
yrpp	2	f	0.6	32.16	1.066	0.181	hypoxia	1	0.876666667	1	Ucrit3hyp	
yrpp	2	f	0.6	32.16	1.066	0.181	normoxia	1	0.85	1	Ucrit1norm	73.87152
yrpp	2	f	0.6	32.16	1.066	0.181	normoxia	1	0.85	1	Ucrit2norm	
yrpp	2	f	0.6	32.16	1.066	0.181	normoxia	1	0.85	1	Ucrit3norm	

yrpp	2	f	0.6	32.16	1.066	0.181	normoxia	1	0.85	1	Ucrit1hyp	56.08704
yrpp	2	f	0.6	32.16	1.066	0.181	normoxia	1	0.85	1	Ucrit2hyp	
yrpp	2	f	0.6	32.16	1.066	0.181	normoxia	1	0.85	1	Ucrit3hyp	
yrpp	2	f	0.6	32.16	1.066	0.181	hypoxia	2	0.901666667	1	Ucrit1norm	73.87152
yrpp	2	f	0.6	32.16	1.066	0.181	hypoxia	2	0.901666667	1	Ucrit2norm	
yrpp	2	f	0.6	32.16	1.066	0.181	hypoxia	2	0.901666667	1	Ucrit3norm	
yrpp	2	f	0.6	32.16	1.066	0.181	hypoxia	2	0.901666667	1	Ucrit1hyp	56.08704
yrpp	2	f	0.6	32.16	1.066	0.181	hypoxia	2	0.901666667	1	Ucrit2hyp	
yrpp	2	f	0.6	32.16	1.066	0.181	hypoxia	2	0.901666667	1	Ucrit3hyp	
yrpp	2	f	0.6	32.16	1.066	0.181	normoxia	2	0.571666667	1	Ucrit1norm	73.87152
yrpp	2	f	0.6	32.16	1.066	0.181	normoxia	2	0.571666667	1	Ucrit2norm	
yrpp	2	f	0.6	32.16	1.066	0.181	normoxia	2	0.571666667	1	Ucrit3norm	
yrpp	2	f	0.6	32.16	1.066	0.181	normoxia	2	0.571666667	1	Ucrit1hyp	56.08704
yrpp	2	f	0.6	32.16	1.066	0.181	normoxia	2	0.571666667	1	Ucrit2hyp	
yrpp	2	f	0.6	32.16	1.066	0.181	normoxia	2	0.571666667	1	Ucrit3hyp	
yrpr	4	m	0.573	32.68	1	0.107	normoxia	1	0	0	Ucrit1norm	86.602
yrpr	4	m	0.573	32.68	1	0.107	normoxia	1	0	0	Ucrit2norm	78.13788
yrpr	4	m	0.573	32.68	1	0.107	normoxia	1	0	0	Ucrit3norm	95.85044
yrpr	4	m	0.573	32.68	1	0.107	normoxia	1	0	0	Ucrit1hyp	70.45808
yrpr	4	m	0.573	32.68	1	0.107	normoxia	1	0	0	Ucrit2hyp	68.69336
yrpr	4	m	0.573	32.68	1	0.107	normoxia	1	0	0	Ucrit3hyp	76.4712
yrpr	4	m	0.573	32.68	1	0.107	hypoxia	1	0.91	1	Ucrit1norm	86.602
yrpr	4	m	0.573	32.68	1	0.107	hypoxia	1	0.91	1	Ucrit2norm	78.13788
yrpr	4	m	0.573	32.68	1	0.107	hypoxia	1	0.91	1	Ucrit3norm	95.85044
yrpr	4	m	0.573	32.68	1	0.107	hypoxia	1	0.91	1	Ucrit1hyp	70.45808
yrpr	4	m	0.573	32.68	1	0.107	hypoxia	1	0.91	1	Ucrit2hyp	68.69336
yrpr	4	m	0.573	32.68	1	0.107	hypoxia	1	0.91	1	Ucrit3hyp	76.4712
yrpr	4	m	0.573	32.68	1	0.107	normoxia	2	0	0	Ucrit1norm	86.602
yrpr	4	m	0.573	32.68	1	0.107	normoxia	2	0	0	Ucrit2norm	78.13788
yrpr	4	m	0.573	32.68	1	0.107	normoxia	2	0	0	Ucrit3norm	95.85044
yrpr	4	m	0.573	32.68	1	0.107	normoxia	2	0	0	Ucrit1hyp	70.45808
yrpr	4	m	0.573	32.68	1	0.107	normoxia	2	0	0	Ucrit2hyp	68.69336
yrpr	4	m	0.573	32.68	1	0.107	normoxia	2	0	0	Ucrit3hyp	76.4712

yrpr	4	m	0.573	32.68	1	0.107	hypoxia	2	0	0	Ucrit1norm	86.602
yrpr	4	m	0.573	32.68	1	0.107	hypoxia	2	0	0	Ucrit2norm	78.13788
yrpr	4	m	0.573	32.68	1	0.107	hypoxia	2	0	0	Ucrit3norm	95.85044
yrpr	4	m	0.573	32.68	1	0.107	hypoxia	2	0	0	Ucrit1hyp	70.45808
yrpr	4	m	0.573	32.68	1	0.107	hypoxia	2	0	0	Ucrit2hyp	68.69336
yrpr	4	m	0.573	32.68	1	0.107	hypoxia	2	0	0	Ucrit3hyp	76.4712
yrpy	1	m	0.52	32.61	1.331	0.202	normoxia	1	0	0	Ucrit1norm	84.98166
yrpy	1	m	0.52	32.61	1.331	0.202	normoxia	1	0	0	Ucrit2norm	
yrpy	1	m	0.52	32.61	1.331	0.202	normoxia	1	0	0	Ucrit3norm	
yrpy	1	m	0.52	32.61	1.331	0.202	normoxia	1	0	0	Ucrit1hyp	70.07889
yrpy	1	m	0.52	32.61	1.331	0.202	normoxia	1	0	0	Ucrit2hyp	
yrpy	1	m	0.52	32.61	1.331	0.202	normoxia	1	0	0	Ucrit3hyp	
yrpy	1	m	0.52	32.61	1.331	0.202	hypoxia	1	0.011666667	1	Ucrit1norm	84.98166
yrpy	1	m	0.52	32.61	1.331	0.202	hypoxia	1	0.011666667	1	Ucrit2norm	
yrpy	1	m	0.52	32.61	1.331	0.202	hypoxia	1	0.011666667	1	Ucrit3norm	
yrpy	1	m	0.52	32.61	1.331	0.202	hypoxia	1	0.011666667	1	Ucrit1hyp	70.07889
yrpy	1	m	0.52	32.61	1.331	0.202	hypoxia	1	0.011666667	1	Ucrit2hyp	
yrpy	1	m	0.52	32.61	1.331	0.202	hypoxia	1	0.011666667	1	Ucrit3hyp	
yrpy	1	m	0.52	32.61	1.331	0.202	normoxia	2	0	0	Ucrit1norm	84.98166
yrpy	1	m	0.52	32.61	1.331	0.202	normoxia	2	0	0	Ucrit2norm	
yrpy	1	m	0.52	32.61	1.331	0.202	normoxia	2	0	0	Ucrit3norm	
yrpy	1	m	0.52	32.61	1.331	0.202	normoxia	2	0	0	Ucrit1hyp	70.07889
yrpy	1	m	0.52	32.61	1.331	0.202	normoxia	2	0	0	Ucrit2hyp	
yrpy	1	m	0.52	32.61	1.331	0.202	normoxia	2	0	0	Ucrit3hyp	
yrpy	1	m	0.52	32.61	1.331	0.202	hypoxia	2	0	0	Ucrit1norm	84.98166
yrpy	1	m	0.52	32.61	1.331	0.202	hypoxia	2	0	0	Ucrit2norm	
yrpy	1	m	0.52	32.61	1.331	0.202	hypoxia	2	0	0	Ucrit3norm	
yrpy	1	m	0.52	32.61	1.331	0.202	hypoxia	2	0	0	Ucrit1hyp	70.07889
yrpy	1	m	0.52	32.61	1.331	0.202	hypoxia	2	0	0	Ucrit2hyp	
yrpy	1	m	0.52	32.61	1.331	0.202	hypoxia	2	0	0	Ucrit3hyp	
yrrp	4	m	0.379	29.6	0.959	0.142	normoxia	1	0	0	Ucrit1norm	90.0728
yrrp	4	m	0.379	29.6	0.959	0.142	normoxia	1	0	0	Ucrit2norm	82.8504
yrrp	4	m	0.379	29.6	0.959	0.142	normoxia	1	0	0	Ucrit3norm	89.1256

yrrp	4	m	0.379	29.6	0.959	0.142	normoxia	1	0	0	Ucrit1hyp	65.3568
yrrp	4	m	0.379	29.6	0.959	0.142	normoxia	1	0	0	Ucrit2hyp	67.6064
yrrp	4	m	0.379	29.6	0.959	0.142	normoxia	1	0	0	Ucrit3hyp	
yrrp	4	m	0.379	29.6	0.959	0.142	hypoxia	1	0	0	Ucrit1norm	90.0728
yrrp	4	m	0.379	29.6	0.959	0.142	hypoxia	1	0	0	Ucrit2norm	82.8504
yrrp	4	m	0.379	29.6	0.959	0.142	hypoxia	1	0	0	Ucrit3norm	89.1256
yrrp	4	m	0.379	29.6	0.959	0.142	hypoxia	1	0	0	Ucrit1hyp	65.3568
yrrp	4	m	0.379	29.6	0.959	0.142	hypoxia	1	0	0	Ucrit2hyp	67.6064
yrrp	4	m	0.379	29.6	0.959	0.142	hypoxia	1	0	0	Ucrit3hyp	
yrrp	4	m	0.379	29.6	0.959	0.142	normoxia	2	0	0	Ucrit1norm	90.0728
yrrp	4	m	0.379	29.6	0.959	0.142	normoxia	2	0	0	Ucrit2norm	82.8504
yrrp	4	m	0.379	29.6	0.959	0.142	normoxia	2	0	0	Ucrit3norm	89.1256
yrrp	4	m	0.379	29.6	0.959	0.142	normoxia	2	0	0	Ucrit1hyp	65.3568
yrrp	4	m	0.379	29.6	0.959	0.142	normoxia	2	0	0	Ucrit2hyp	67.6064
yrrp	4	m	0.379	29.6	0.959	0.142	normoxia	2	0	0	Ucrit3hyp	
yrrp	4	m	0.379	29.6	0.959	0.142	hypoxia	2	0	0	Ucrit1norm	90.0728
yrrp	4	m	0.379	29.6	0.959	0.142	hypoxia	2	0	0	Ucrit2norm	82.8504
yrrp	4	m	0.379	29.6	0.959	0.142	hypoxia	2	0	0	Ucrit3norm	89.1256
yrrp	4	m	0.379	29.6	0.959	0.142	hypoxia	2	0	0	Ucrit1hyp	65.3568
yrrp	4	m	0.379	29.6	0.959	0.142	hypoxia	2	0	0	Ucrit2hyp	67.6064
yrrp	4	m	0.379	29.6	0.959	0.142	hypoxia	2	0	0	Ucrit3hyp	
yrrr	4	f	0.603	32.03	1.389	0.27	normoxia	1	0	0	Ucrit1norm	78.34538
yrrr	4	f	0.603	32.03	1.389	0.27	normoxia	1	0	0	Ucrit2norm	83.63033
yrrr	4	f	0.603	32.03	1.389	0.27	normoxia	1	0	0	Ucrit3norm	83.02176
yrrr	4	f	0.603	32.03	1.389	0.27	normoxia	1	0	0	Ucrit1hyp	72.48389
yrrr	4	f	0.603	32.03	1.389	0.27	normoxia	1	0	0	Ucrit2hyp	68.60826
yrrr	4	f	0.603	32.03	1.389	0.27	normoxia	1	0	0	Ucrit3hyp	77.64072
yrrr	4	f	0.603	32.03	1.389	0.27	hypoxia	1	0	0	Ucrit1norm	78.34538
yrrr	4	f	0.603	32.03	1.389	0.27	hypoxia	1	0	0	Ucrit2norm	83.63033
yrrr	4	f	0.603	32.03	1.389	0.27	hypoxia	1	0	0	Ucrit3norm	83.02176
yrrr	4	f	0.603	32.03	1.389	0.27	hypoxia	1	0	0	Ucrit1hyp	72.48389
yrrr	4	f	0.603	32.03	1.389	0.27	hypoxia	1	0	0	Ucrit2hyp	68.60826
yrrr	4	f	0.603	32.03	1.389	0.27	hypoxia	1	0	0	Ucrit3hyp	77.64072

yrrr	4	f	0.603	32.03	1.389	0.27	normoxia	2	0	0	Ucrit1norm	78.34538
yrrr	4	f	0.603	32.03	1.389	0.27	normoxia	2	0	0	Ucrit2norm	83.63033
yrrr	4	f	0.603	32.03	1.389	0.27	normoxia	2	0	0	Ucrit3norm	83.02176
yrrr	4	f	0.603	32.03	1.389	0.27	normoxia	2	0	0	Ucrit1hyp	72.48389
yrrr	4	f	0.603	32.03	1.389	0.27	normoxia	2	0	0	Ucrit2hyp	68.60826
yrrr	4	f	0.603	32.03	1.389	0.27	normoxia	2	0	0	Ucrit3hyp	77.64072
yrrr	4	f	0.603	32.03	1.389	0.27	hypoxia	2	0	0	Ucrit1norm	78.34538
yrrr	4	f	0.603	32.03	1.389	0.27	hypoxia	2	0	0	Ucrit2norm	83.63033
yrrr	4	f	0.603	32.03	1.389	0.27	hypoxia	2	0	0	Ucrit3norm	83.02176
yrrr	4	f	0.603	32.03	1.389	0.27	hypoxia	2	0	0	Ucrit1hyp	72.48389
yrrr	4	f	0.603	32.03	1.389	0.27	hypoxia	2	0	0	Ucrit2hyp	68.60826
yrrr	4	f	0.603	32.03	1.389	0.27	hypoxia	2	0	0	Ucrit3hyp	77.64072
yrry	3	f	0.801	35.14	1.552	0.247	hypoxia	1	0	0	Ucrit1norm	78.92444
yrry	3	f	0.801	35.14	1.552	0.247	hypoxia	1	0	0	Ucrit2norm	
yrry	3	f	0.801	35.14	1.552	0.247	hypoxia	1	0	0	Ucrit3norm	
yrry	3	f	0.801	35.14	1.552	0.247	hypoxia	1	0	0	Ucrit1hyp	59.70286
yrry	3	f	0.801	35.14	1.552	0.247	hypoxia	1	0	0	Ucrit2hyp	
yrry	3	f	0.801	35.14	1.552	0.247	hypoxia	1	0	0	Ucrit3hyp	
yrry	3	f	0.801	35.14	1.552	0.247	normoxia	1	0	0	Ucrit1norm	78.92444
yrry	3	f	0.801	35.14	1.552	0.247	normoxia	1	0	0	Ucrit2norm	
yrry	3	f	0.801	35.14	1.552	0.247	normoxia	1	0	0	Ucrit3norm	
yrry	3	f	0.801	35.14	1.552	0.247	normoxia	1	0	0	Ucrit1hyp	59.70286
yrry	3	f	0.801	35.14	1.552	0.247	normoxia	1	0	0	Ucrit2hyp	
yrry	3	f	0.801	35.14	1.552	0.247	normoxia	1	0	0	Ucrit3hyp	
yrry	3	f	0.801	35.14	1.552	0.247	hypoxia	2	0	0	Ucrit1norm	78.92444
yrry	3	f	0.801	35.14	1.552	0.247	hypoxia	2	0	0	Ucrit2norm	
yrry	3	f	0.801	35.14	1.552	0.247	hypoxia	2	0	0	Ucrit3norm	
yrry	3	f	0.801	35.14	1.552	0.247	hypoxia	2	0	0	Ucrit1hyp	59.70286
yrry	3	f	0.801	35.14	1.552	0.247	hypoxia	2	0	0	Ucrit2hyp	
yrry	3	f	0.801	35.14	1.552	0.247	hypoxia	2	0	0	Ucrit3hyp	
yrry	3	f	0.801	35.14	1.552	0.247	normoxia	2	0	0	Ucrit1norm	78.92444
yrry	3	f	0.801	35.14	1.552	0.247	normoxia	2	0	0	Ucrit2norm	
yrry	3	f	0.801	35.14	1.552	0.247	normoxia	2	0	0	Ucrit3norm	

yrry	3	f	0.801	35.14	1.552	0.247	normoxia	2	0	0	Ucrit1hyp	59.70286
yrry	3	f	0.801	35.14	1.552	0.247	normoxia	2	0	0	Ucrit2hyp	
yrry	3	f	0.801	35.14	1.552	0.247	normoxia	2	0	0	Ucrit3hyp	
yryg	3	m	0.516	32.81	1.15	0.185	hypoxia	1	0	0	Ucrit1norm	91.17899
yryg	3	m	0.516	32.81	1.15	0.185	hypoxia	1	0	0	Ucrit2norm	
yryg	3	m	0.516	32.81	1.15	0.185	hypoxia	1	0	0	Ucrit3norm	
yryg	3	m	0.516	32.81	1.15	0.185	hypoxia	1	0	0	Ucrit1hyp	66.73554
yryg	3	m	0.516	32.81	1.15	0.185	hypoxia	1	0	0	Ucrit2hyp	
yryg	3	m	0.516	32.81	1.15	0.185	hypoxia	1	0	0	Ucrit3hyp	
yryg	3	m	0.516	32.81	1.15	0.185	normoxia	1	0	0	Ucrit1norm	91.17899
yryg	3	m	0.516	32.81	1.15	0.185	normoxia	1	0	0	Ucrit2norm	
yryg	3	m	0.516	32.81	1.15	0.185	normoxia	1	0	0	Ucrit3norm	
yryg	3	m	0.516	32.81	1.15	0.185	normoxia	1	0	0	Ucrit1hyp	66.73554
yryg	3	m	0.516	32.81	1.15	0.185	normoxia	1	0	0	Ucrit2hyp	
yryg	3	m	0.516	32.81	1.15	0.185	normoxia	1	0	0	Ucrit3hyp	
yryg	3	m	0.516	32.81	1.15	0.185	hypoxia	2	0	0	Ucrit1norm	91.17899
yryg	3	m	0.516	32.81	1.15	0.185	hypoxia	2	0	0	Ucrit2norm	
yryg	3	m	0.516	32.81	1.15	0.185	hypoxia	2	0	0	Ucrit3norm	
yryg	3	m	0.516	32.81	1.15	0.185	hypoxia	2	0	0	Ucrit1hyp	66.73554
yryg	3	m	0.516	32.81	1.15	0.185	hypoxia	2	0	0	Ucrit2hyp	
yryg	3	m	0.516	32.81	1.15	0.185	hypoxia	2	0	0	Ucrit3hyp	
yryg	3	m	0.516	32.81	1.15	0.185	normoxia	2	0	0	Ucrit1norm	91.17899
yryg	3	m	0.516	32.81	1.15	0.185	normoxia	2	0	0	Ucrit2norm	
yryg	3	m	0.516	32.81	1.15	0.185	normoxia	2	0	0	Ucrit3norm	
yryg	3	m	0.516	32.81	1.15	0.185	normoxia	2	0	0	Ucrit1hyp	66.73554
yryg	3	m	0.516	32.81	1.15	0.185	normoxia	2	0	0	Ucrit2hyp	
yryg	3	m	0.516	32.81	1.15	0.185	normoxia	2	0	0	Ucrit3hyp	
yryp	3	m	0.545	34.87	1.195	0.182	hypoxia	1	0.666666667	1	Ucrit1norm	78.63185
yryp	3	m	0.545	34.87	1.195	0.182	hypoxia	1	0.666666667	1	Ucrit2norm	
yryp	3	m	0.545	34.87	1.195	0.182	hypoxia	1	0.666666667	1	Ucrit3norm	
yryp	3	m	0.545	34.87	1.195	0.182	hypoxia	1	0.666666667	1	Ucrit1hyp	61.82451
yryp	3	m	0.545	34.87	1.195	0.182	hypoxia	1	0.666666667	1	Ucrit2hyp	
yryp	3	m	0.545	34.87	1.195	0.182	hypoxia	1	0.666666667	1	Ucrit3hyp	

yryp	3	m	0.545	34.87	1.195	0.182	normoxia	1	0	0	Ucrit1norm	78.63185
yryp	3	m	0.545	34.87	1.195	0.182	normoxia	1	0	0	Ucrit2norm	
yryp	3	m	0.545	34.87	1.195	0.182	normoxia	1	0	0	Ucrit3norm	
yryp	3	m	0.545	34.87	1.195	0.182	normoxia	1	0	0	Ucrit1hyp	61.82451
yryp	3	m	0.545	34.87	1.195	0.182	normoxia	1	0	0	Ucrit2hyp	
yryp	3	m	0.545	34.87	1.195	0.182	normoxia	1	0	0	Ucrit3hyp	
yryp	3	m	0.545	34.87	1.195	0.182	hypoxia	2	0	0	Ucrit1norm	78.63185
yryp	3	m	0.545	34.87	1.195	0.182	hypoxia	2	0	0	Ucrit2norm	
yryp	3	m	0.545	34.87	1.195	0.182	hypoxia	2	0	0	Ucrit3norm	
yryp	3	m	0.545	34.87	1.195	0.182	hypoxia	2	0	0	Ucrit1hyp	61.82451
yryp	3	m	0.545	34.87	1.195	0.182	hypoxia	2	0	0	Ucrit2hyp	
yryp	3	m	0.545	34.87	1.195	0.182	hypoxia	2	0	0	Ucrit3hyp	
yryp	3	m	0.545	34.87	1.195	0.182	normoxia	2	0	0	Ucrit1norm	78.63185
yryp	3	m	0.545	34.87	1.195	0.182	normoxia	2	0	0	Ucrit2norm	
yryp	3	m	0.545	34.87	1.195	0.182	normoxia	2	0	0	Ucrit3norm	
yryp	3	m	0.545	34.87	1.195	0.182	normoxia	2	0	0	Ucrit1hyp	61.82451
yryp	3	m	0.545	34.87	1.195	0.182	normoxia	2	0	0	Ucrit2hyp	
yryp	3	m	0.545	34.87	1.195	0.182	normoxia	2	0	0	Ucrit3hyp	
yryr	2	m	0.578	32.65	1.061	0.056	hypoxia	1	0.346666667	1	Ucrit1norm	80.41695
yryr	2	m	0.578	32.65	1.061	0.056	hypoxia	1	0.346666667	1	Ucrit2norm	
yryr	2	m	0.578	32.65	1.061	0.056	hypoxia	1	0.346666667	1	Ucrit3norm	
yryr	2	m	0.578	32.65	1.061	0.056	hypoxia	1	0.346666667	1	Ucrit1hyp	69.8057
yryr	2	m	0.578	32.65	1.061	0.056	hypoxia	1	0.346666667	1	Ucrit2hyp	
yryr	2	m	0.578	32.65	1.061	0.056	hypoxia	1	0.346666667	1	Ucrit3hyp	
yryr	2	m	0.578	32.65	1.061	0.056	normoxia	1	0	0	Ucrit1norm	80.41695
yryr	2	m	0.578	32.65	1.061	0.056	normoxia	1	0	0	Ucrit2norm	
yryr	2	m	0.578	32.65	1.061	0.056	normoxia	1	0	0	Ucrit3norm	
yryr	2	m	0.578	32.65	1.061	0.056	normoxia	1	0	0	Ucrit1hyp	69.8057
yryr	2	m	0.578	32.65	1.061	0.056	normoxia	1	0	0	Ucrit2hyp	
yryr	2	m	0.578	32.65	1.061	0.056	normoxia	1	0	0	Ucrit3hyp	
yryr	2	m	0.578	32.65	1.061	0.056	hypoxia	2	0.861666667	1	Ucrit1norm	80.41695
yryr	2	m	0.578	32.65	1.061	0.056	hypoxia	2	0.861666667	1	Ucrit2norm	
yryr	2	m	0.578	32.65	1.061	0.056	hypoxia	2	0.861666667	1	Ucrit3norm	

yryr	2	m	0.578	32.65	1.061	0.056	hypoxia	2	0.861666667	1	Ucrit1hyp	69.8057
yryr	2	m	0.578	32.65	1.061	0.056	hypoxia	2	0.861666667	1	Ucrit2hyp	
yryr	2	m	0.578	32.65	1.061	0.056	hypoxia	2	0.861666667	1	Ucrit3hyp	
yryr	2	m	0.578	32.65	1.061	0.056	normoxia	2	0	0	Ucrit1norm	80.41695
yryr	2	m	0.578	32.65	1.061	0.056	normoxia	2	0	0	Ucrit2norm	
yryr	2	m	0.578	32.65	1.061	0.056	normoxia	2	0	0	Ucrit3norm	
yryr	2	m	0.578	32.65	1.061	0.056	normoxia	2	0	0	Ucrit1hyp	69.8057
yryr	2	m	0.578	32.65	1.061	0.056	normoxia	2	0	0	Ucrit2hyp	
yryr	2	m	0.578	32.65	1.061	0.056	normoxia	2	0	0	Ucrit3hyp	
yryy	1	f	0.667	33.5	0.814	0.215	normoxia	1	0	0	Ucrit1norm	85.2575
yryy	1	f	0.667	33.5	0.814	0.215	normoxia	1	0	0	Ucrit2norm	
yryy	1	f	0.667	33.5	0.814	0.215	normoxia	1	0	0	Ucrit3norm	
yryy	1	f	0.667	33.5	0.814	0.215	normoxia	1	0	0	Ucrit1hyp	62.444
yryy	1	f	0.667	33.5	0.814	0.215	normoxia	1	0	0	Ucrit2hyp	
yryy	1	f	0.667	33.5	0.814	0.215	normoxia	1	0	0	Ucrit3hyp	
yryy	1	f	0.667	33.5	0.814	0.215	hypoxia	1	0	0	Ucrit1norm	85.2575
yryy	1	f	0.667	33.5	0.814	0.215	hypoxia	1	0	0	Ucrit2norm	
yryy	1	f	0.667	33.5	0.814	0.215	hypoxia	1	0	0	Ucrit3norm	
yryy	1	f	0.667	33.5	0.814	0.215	hypoxia	1	0	0	Ucrit1hyp	62.444
yryy	1	f	0.667	33.5	0.814	0.215	hypoxia	1	0	0	Ucrit2hyp	
yryy	1	f	0.667	33.5	0.814	0.215	hypoxia	1	0	0	Ucrit3hyp	
yryy	1	f	0.667	33.5	0.814	0.215	normoxia	2	0	0	Ucrit1norm	85.2575
yryy	1	f	0.667	33.5	0.814	0.215	normoxia	2	0	0	Ucrit2norm	
yryy	1	f	0.667	33.5	0.814	0.215	normoxia	2	0	0	Ucrit3norm	
yryy	1	f	0.667	33.5	0.814	0.215	normoxia	2	0	0	Ucrit1hyp	62.444
yryy	1	f	0.667	33.5	0.814	0.215	normoxia	2	0	0	Ucrit2hyp	
yryy	1	f	0.667	33.5	0.814	0.215	normoxia	2	0	0	Ucrit3hyp	
yryy	1	f	0.667	33.5	0.814	0.215	hypoxia	2	0	0	Ucrit1norm	85.2575
yryy	1	f	0.667	33.5	0.814	0.215	hypoxia	2	0	0	Ucrit2norm	
yryy	1	f	0.667	33.5	0.814	0.215	hypoxia	2	0	0	Ucrit3norm	
yryy	1	f	0.667	33.5	0.814	0.215	hypoxia	2	0	0	Ucrit1hyp	62.444
yryy	1	f	0.667	33.5	0.814	0.215	hypoxia	2	0	0	Ucrit2hyp	
yryy	1	f	0.667	33.5	0.814	0.215	hypoxia	2	0	0	Ucrit3hyp	

yygg	1	m	0.441	32	0.895	0.169	normoxia	1	0	0	Ucrit1norm	82.752
yygg	1	m	0.441	32	0.895	0.169	normoxia	1	0	0	Ucrit2norm	
yygg	1	m	0.441	32	0.895	0.169	normoxia	1	0	0	Ucrit3norm	
yygg	1	m	0.441	32	0.895	0.169	normoxia	1	0	0	Ucrit1hyp	61.696
yygg	1	m	0.441	32	0.895	0.169	normoxia	1	0	0	Ucrit2hyp	
yygg	1	m	0.441	32	0.895	0.169	normoxia	1	0	0	Ucrit3hyp	
yygg	1	m	0.441	32	0.895	0.169	hypoxia	1	0.721666667	1	Ucrit1norm	82.752
yygg	1	m	0.441	32	0.895	0.169	hypoxia	1	0.721666667	1	Ucrit2norm	
yygg	1	m	0.441	32	0.895	0.169	hypoxia	1	0.721666667	1	Ucrit3norm	
yygg	1	m	0.441	32	0.895	0.169	hypoxia	1	0.721666667	1	Ucrit1hyp	61.696
yygg	1	m	0.441	32	0.895	0.169	hypoxia	1	0.721666667	1	Ucrit2hyp	
yygg	1	m	0.441	32	0.895	0.169	hypoxia	1	0.721666667	1	Ucrit3hyp	
yygg	1	m	0.441	32	0.895	0.169	normoxia	2	0	0	Ucrit1norm	82.752
yygg	1	m	0.441	32	0.895	0.169	normoxia	2	0	0	Ucrit2norm	
yygg	1	m	0.441	32	0.895	0.169	normoxia	2	0	0	Ucrit3norm	
yygg	1	m	0.441	32	0.895	0.169	normoxia	2	0	0	Ucrit1hyp	61.696
yygg	1	m	0.441	32	0.895	0.169	normoxia	2	0	0	Ucrit2hyp	
yygg	1	m	0.441	32	0.895	0.169	normoxia	2	0	0	Ucrit3hyp	
yygg	1	m	0.441	32	0.895	0.169	hypoxia	2	0	0	Ucrit1norm	82.752
yygg	1	m	0.441	32	0.895	0.169	hypoxia	2	0	0	Ucrit2norm	
yygg	1	m	0.441	32	0.895	0.169	hypoxia	2	0	0	Ucrit3norm	
yygg	1	m	0.441	32	0.895	0.169	hypoxia	2	0	0	Ucrit1hyp	61.696
yygg	1	m	0.441	32	0.895	0.169	hypoxia	2	0	0	Ucrit2hyp	
yygg	1	m	0.441	32	0.895	0.169	hypoxia	2	0	0	Ucrit3hyp	
yygp	2	f	0.58	33.48	0.82	0.202	hypoxia	1	0	0	Ucrit1norm	81.189
yygp	2	f	0.58	33.48	0.82	0.202	hypoxia	1	0	0	Ucrit2norm	
yygp	2	f	0.58	33.48	0.82	0.202	hypoxia	1	0	0	Ucrit3norm	
yygp	2	f	0.58	33.48	0.82	0.202	hypoxia	1	0	0	Ucrit1hyp	61.4358
yygp	2	f	0.58	33.48	0.82	0.202	hypoxia	1	0	0	Ucrit2hyp	
yygp	2	f	0.58	33.48	0.82	0.202	hypoxia	1	0	0	Ucrit3hyp	
yygp	2	f	0.58	33.48	0.82	0.202	normoxia	1	0	0	Ucrit1norm	81.189
yygp	2	f	0.58	33.48	0.82	0.202	normoxia	1	0	0	Ucrit2norm	
yygp	2	f	0.58	33.48	0.82	0.202	normoxia	1	0	0	Ucrit3norm	

yygp	2	f	0.58	33.48	0.82	0.202	normoxia	1	0	0	Ucrit1hyp	61.4358
yygp	2	f	0.58	33.48	0.82	0.202	normoxia	1	0	0	Ucrit2hyp	
yygp	2	f	0.58	33.48	0.82	0.202	normoxia	1	0	0	Ucrit3hyp	
yygp	2	f	0.58	33.48	0.82	0.202	hypoxia	2	0	0	Ucrit1norm	81.189
yygp	2	f	0.58	33.48	0.82	0.202	hypoxia	2	0	0	Ucrit2norm	
yygp	2	f	0.58	33.48	0.82	0.202	hypoxia	2	0	0	Ucrit3norm	
yygp	2	f	0.58	33.48	0.82	0.202	hypoxia	2	0	0	Ucrit1hyp	61.4358
yygp	2	f	0.58	33.48	0.82	0.202	hypoxia	2	0	0	Ucrit2hyp	
yygp	2	f	0.58	33.48	0.82	0.202	hypoxia	2	0	0	Ucrit3hyp	
yygp	2	f	0.58	33.48	0.82	0.202	normoxia	2	0	0	Ucrit1norm	81.189
yygp	2	f	0.58	33.48	0.82	0.202	normoxia	2	0	0	Ucrit2norm	
yygp	2	f	0.58	33.48	0.82	0.202	normoxia	2	0	0	Ucrit3norm	
yygp	2	f	0.58	33.48	0.82	0.202	normoxia	2	0	0	Ucrit1hyp	61.4358
yygp	2	f	0.58	33.48	0.82	0.202	normoxia	2	0	0	Ucrit2hyp	
yygp	2	f	0.58	33.48	0.82	0.202	normoxia	2	0	0	Ucrit3hyp	
yygr	3	f	0.588	31.86	0.977	0.206	hypoxia	1	0	0	Ucrit1norm	75.12588
yygr	3	f	0.588	31.86	0.977	0.206	hypoxia	1	0	0	Ucrit2norm	
yygr	3	f	0.588	31.86	0.977	0.206	hypoxia	1	0	0	Ucrit3norm	
yygr	3	f	0.588	31.86	0.977	0.206	hypoxia	1	0	0	Ucrit1hyp	59.70564
yygr	3	f	0.588	31.86	0.977	0.206	hypoxia	1	0	0	Ucrit2hyp	
yygr	3	f	0.588	31.86	0.977	0.206	hypoxia	1	0	0	Ucrit3hyp	
yygr	3	f	0.588	31.86	0.977	0.206	normoxia	1	0	0	Ucrit1norm	75.12588
yygr	3	f	0.588	31.86	0.977	0.206	normoxia	1	0	0	Ucrit2norm	
yygr	3	f	0.588	31.86	0.977	0.206	normoxia	1	0	0	Ucrit3norm	
yygr	3	f	0.588	31.86	0.977	0.206	normoxia	1	0	0	Ucrit1hyp	59.70564
yygr	3	f	0.588	31.86	0.977	0.206	normoxia	1	0	0	Ucrit2hyp	
yygr	3	f	0.588	31.86	0.977	0.206	normoxia	1	0	0	Ucrit3hyp	
yygr	3	f	0.588	31.86	0.977	0.206	hypoxia	2	0	0	Ucrit1norm	75.12588
yygr	3	f	0.588	31.86	0.977	0.206	hypoxia	2	0	0	Ucrit2norm	
yygr	3	f	0.588	31.86	0.977	0.206	hypoxia	2	0	0	Ucrit3norm	
yygr	3	f	0.588	31.86	0.977	0.206	hypoxia	2	0	0	Ucrit1hyp	59.70564
yygr	3	f	0.588	31.86	0.977	0.206	hypoxia	2	0	0	Ucrit2hyp	
yygr	3	f	0.588	31.86	0.977	0.206	hypoxia	2	0	0	Ucrit3hyp	

yygr	3	f	0.588	31.86	0.977	0.206	normoxia	2	0	0	Ucrit1norm	75.12588
yygr	3	f	0.588	31.86	0.977	0.206	normoxia	2	0	0	Ucrit2norm	
yygr	3	f	0.588	31.86	0.977	0.206	normoxia	2	0	0	Ucrit3norm	
yygr	3	f	0.588	31.86	0.977	0.206	normoxia	2	0	0	Ucrit1hyp	59.70564
yygr	3	f	0.588	31.86	0.977	0.206	normoxia	2	0	0	Ucrit2hyp	
yygr	3	f	0.588	31.86	0.977	0.206	normoxia	2	0	0	Ucrit3hyp	
yygy	2	f	0.721	36.97	1.055	0.207	hypoxia	1	0.326666667	1	Ucrit1norm	57.93199
yygy	2	f	0.721	36.97	1.055	0.207	hypoxia	1	0.326666667	1	Ucrit2norm	
yygy	2	f	0.721	36.97	1.055	0.207	hypoxia	1	0.326666667	1	Ucrit3norm	
yygy	2	f	0.721	36.97	1.055	0.207	hypoxia	1	0.326666667	1	Ucrit1hyp	49.79859
yygy	2	f	0.721	36.97	1.055	0.207	hypoxia	1	0.326666667	1	Ucrit2hyp	
yygy	2	f	0.721	36.97	1.055	0.207	hypoxia	1	0.326666667	1	Ucrit3hyp	
yygy	2	f	0.721	36.97	1.055	0.207	normoxia	1	0	0	Ucrit1norm	57.93199
yygy	2	f	0.721	36.97	1.055	0.207	normoxia	1	0	0	Ucrit2norm	
yygy	2	f	0.721	36.97	1.055	0.207	normoxia	1	0	0	Ucrit3norm	
yygy	2	f	0.721	36.97	1.055	0.207	normoxia	1	0	0	Ucrit1hyp	49.79859
yygy	2	f	0.721	36.97	1.055	0.207	normoxia	1	0	0	Ucrit2hyp	
yygy	2	f	0.721	36.97	1.055	0.207	normoxia	1	0	0	Ucrit3hyp	
yygy	2	f	0.721	36.97	1.055	0.207	hypoxia	2	0.038333333	1	Ucrit1norm	57.93199
yygy	2	f	0.721	36.97	1.055	0.207	hypoxia	2	0.038333333	1	Ucrit2norm	
yygy	2	f	0.721	36.97	1.055	0.207	hypoxia	2	0.038333333	1	Ucrit3norm	
yygy	2	f	0.721	36.97	1.055	0.207	hypoxia	2	0.038333333	1	Ucrit1hyp	49.79859
yygy	2	f	0.721	36.97	1.055	0.207	hypoxia	2	0.038333333	1	Ucrit2hyp	
yygy	2	f	0.721	36.97	1.055	0.207	hypoxia	2	0.038333333	1	Ucrit3hyp	
yygy	2	f	0.721	36.97	1.055	0.207	normoxia	2	0.186666667	1	Ucrit1norm	57.93199
yygy	2	f	0.721	36.97	1.055	0.207	normoxia	2	0.186666667	1	Ucrit2norm	
yygy	2	f	0.721	36.97	1.055	0.207	normoxia	2	0.186666667	1	Ucrit3norm	
yygy	2	f	0.721	36.97	1.055	0.207	normoxia	2	0.186666667	1	Ucrit1hyp	49.79859
yygy	2	f	0.721	36.97	1.055	0.207	normoxia	2	0.186666667	1	Ucrit2hyp	
yygy	2	f	0.721	36.97	1.055	0.207	normoxia	2	0.186666667	1	Ucrit3hyp	
yypy	1	f	0.538	33.38	0.895	0.185	normoxia	1	0	0	Ucrit1norm	75.47218
yypy	1	f	0.538	33.38	0.895	0.185	normoxia	1	0	0	Ucrit2norm	
yypy	1	f	0.538	33.38	0.895	0.185	normoxia	1	0	0	Ucrit3norm	

yypy	1	f	0.538	33.38	0.895	0.185	normoxia	1	0	0	Ucrit1hyp	59.78358
yypy	1	f	0.538	33.38	0.895	0.185	normoxia	1	0	0	Ucrit2hyp	
yypy	1	f	0.538	33.38	0.895	0.185	normoxia	1	0	0	Ucrit3hyp	
yypy	1	f	0.538	33.38	0.895	0.185	hypoxia	1	0	0	Ucrit1norm	75.47218
yypy	1	f	0.538	33.38	0.895	0.185	hypoxia	1	0	0	Ucrit2norm	
yypy	1	f	0.538	33.38	0.895	0.185	hypoxia	1	0	0	Ucrit3norm	
yypy	1	f	0.538	33.38	0.895	0.185	hypoxia	1	0	0	Ucrit1hyp	59.78358
yypy	1	f	0.538	33.38	0.895	0.185	hypoxia	1	0	0	Ucrit2hyp	
yypy	1	f	0.538	33.38	0.895	0.185	hypoxia	1	0	0	Ucrit3hyp	
yypy	1	f	0.538	33.38	0.895	0.185	normoxia	2	0	0	Ucrit1norm	75.47218
yypy	1	f	0.538	33.38	0.895	0.185	normoxia	2	0	0	Ucrit2norm	
yypy	1	f	0.538	33.38	0.895	0.185	normoxia	2	0	0	Ucrit3norm	
yypy	1	f	0.538	33.38	0.895	0.185	normoxia	2	0	0	Ucrit1hyp	59.78358
yypy	1	f	0.538	33.38	0.895	0.185	normoxia	2	0	0	Ucrit2hyp	
yypy	1	f	0.538	33.38	0.895	0.185	normoxia	2	0	0	Ucrit3hyp	
yypy	1	f	0.538	33.38	0.895	0.185	hypoxia	2	0.471666667	1	Ucrit1norm	75.47218
yypy	1	f	0.538	33.38	0.895	0.185	hypoxia	2	0.471666667	1	Ucrit2norm	
yypy	1	f	0.538	33.38	0.895	0.185	hypoxia	2	0.471666667	1	Ucrit3norm	
yypy	1	f	0.538	33.38	0.895	0.185	hypoxia	2	0.471666667	1	Ucrit1hyp	59.78358
yypy	1	f	0.538	33.38	0.895	0.185	hypoxia	2	0.471666667	1	Ucrit2hyp	
yypy	1	f	0.538	33.38	0.895	0.185	hypoxia	2	0.471666667	1	Ucrit3hyp	
yyrg	1	m	0.555	31.07	0.928	0.17	normoxia	1	0	0	Ucrit1norm	73.54269
yyrg	1	m	0.555	31.07	0.928	0.17	normoxia	1	0	0	Ucrit2norm	
yyrg	1	m	0.555	31.07	0.928	0.17	normoxia	1	0	0	Ucrit3norm	
yyrg	1	m	0.555	31.07	0.928	0.17	normoxia	1	0	0	Ucrit1hyp	69.56573
yyrg	1	m	0.555	31.07	0.928	0.17	normoxia	1	0	0	Ucrit2hyp	
yyrg	1	m	0.555	31.07	0.928	0.17	normoxia	1	0	0	Ucrit3hyp	
yyrg	1	m	0.555	31.07	0.928	0.17	hypoxia	1	0	0	Ucrit1norm	73.54269
yyrg	1	m	0.555	31.07	0.928	0.17	hypoxia	1	0	0	Ucrit2norm	
yyrg	1	m	0.555	31.07	0.928	0.17	hypoxia	1	0	0	Ucrit3norm	
yyrg	1	m	0.555	31.07	0.928	0.17	hypoxia	1	0	0	Ucrit1hyp	69.56573
yyrg	1	m	0.555	31.07	0.928	0.17	hypoxia	1	0	0	Ucrit2hyp	
yyrg	1	m	0.555	31.07	0.928	0.17	hypoxia	1	0	0	Ucrit3hyp	

yyrg	1	m	0.555	31.07	0.928	0.17	normoxia	2	0	0	Ucrit1norm	73.54269
yyrg	1	m	0.555	31.07	0.928	0.17	normoxia	2	0	0	Ucrit2norm	
yyrg	1	m	0.555	31.07	0.928	0.17	normoxia	2	0	0	Ucrit3norm	
yyrg	1	m	0.555	31.07	0.928	0.17	normoxia	2	0	0	Ucrit1hyp	69.56573
yyrg	1	m	0.555	31.07	0.928	0.17	normoxia	2	0	0	Ucrit2hyp	
yyrg	1	m	0.555	31.07	0.928	0.17	normoxia	2	0	0	Ucrit3hyp	
yyrg	1	m	0.555	31.07	0.928	0.17	hypoxia	2	0.826666667	1	Ucrit1norm	73.54269
yyrg	1	m	0.555	31.07	0.928	0.17	hypoxia	2	0.826666667	1	Ucrit2norm	
yyrg	1	m	0.555	31.07	0.928	0.17	hypoxia	2	0.826666667	1	Ucrit3norm	
yyrg	1	m	0.555	31.07	0.928	0.17	hypoxia	2	0.826666667	1	Ucrit1hyp	69.56573
yyrg	1	m	0.555	31.07	0.928	0.17	hypoxia	2	0.826666667	1	Ucrit2hyp	
yyrg	1	m	0.555	31.07	0.928	0.17	hypoxia	2	0.826666667	1	Ucrit3hyp	
yyrp	4	m	0.447	32.14	0.625	0.107	normoxia	1	0	0	Ucrit1norm	67.84754
yyrp	4	m	0.447	32.14	0.625	0.107	normoxia	1	0	0	Ucrit2norm	67.87968
yyrp	4	m	0.447	32.14	0.625	0.107	normoxia	1	0	0	Ucrit3norm	64.95494
yyrp	4	m	0.447	32.14	0.625	0.107	normoxia	1	0	0	Ucrit1hyp	62.0302
yyrp	4	m	0.447	32.14	0.625	0.107	normoxia	1	0	0	Ucrit2hyp	66.24054
yyrp	4	m	0.447	32.14	0.625	0.107	normoxia	1	0	0	Ucrit3hyp	61.99806
yyrp	4	m	0.447	32.14	0.625	0.107	hypoxia	1	0.756666667	1	Ucrit1norm	67.84754
yyrp	4	m	0.447	32.14	0.625	0.107	hypoxia	1	0.756666667	1	Ucrit2norm	67.87968
yyrp	4	m	0.447	32.14	0.625	0.107	hypoxia	1	0.756666667	1	Ucrit3norm	64.95494
yyrp	4	m	0.447	32.14	0.625	0.107	hypoxia	1	0.756666667	1	Ucrit1hyp	62.0302
yyrp	4	m	0.447	32.14	0.625	0.107	hypoxia	1	0.756666667	1	Ucrit2hyp	66.24054
yyrp	4	m	0.447	32.14	0.625	0.107	hypoxia	1	0.756666667	1	Ucrit3hyp	61.99806
yyrp	4	m	0.447	32.14	0.625	0.107	normoxia	2	0	0	Ucrit1norm	67.84754
yyrp	4	m	0.447	32.14	0.625	0.107	normoxia	2	0	0	Ucrit2norm	67.87968
yyrp	4	m	0.447	32.14	0.625	0.107	normoxia	2	0	0	Ucrit3norm	64.95494
yyrp	4	m	0.447	32.14	0.625	0.107	normoxia	2	0	0	Ucrit1hyp	62.0302
yyrp	4	m	0.447	32.14	0.625	0.107	normoxia	2	0	0	Ucrit2hyp	66.24054
yyrp	4	m	0.447	32.14	0.625	0.107	normoxia	2	0	0	Ucrit3hyp	61.99806
yyrp	4	m	0.447	32.14	0.625	0.107	hypoxia	2	0	0	Ucrit1norm	67.84754
yyrp	4	m	0.447	32.14	0.625	0.107	hypoxia	2	0	0	Ucrit2norm	67.87968
yyrp	4	m	0.447	32.14	0.625	0.107	hypoxia	2	0	0	Ucrit3norm	64.95494

yyrp	4	m	0.447	32.14	0.625	0.107	hypoxia	2	0	0	Ucrit1hyp	62.0302
yyrp	4	m	0.447	32.14	0.625	0.107	hypoxia	2	0	0	Ucrit2hyp	66.24054
yyrp	4	m	0.447	32.14	0.625	0.107	hypoxia	2	0	0	Ucrit3hyp	61.99806
yyry	2	f	0.708	35.03	1.118	0.211	hypoxia	1	0	0	Ucrit1norm	77.03097
yyry	2	f	0.708	35.03	1.118	0.211	hypoxia	1	0	0	Ucrit2norm	
yyry	2	f	0.708	35.03	1.118	0.211	hypoxia	1	0	0	Ucrit3norm	
yyry	2	f	0.708	35.03	1.118	0.211	hypoxia	1	0	0	Ucrit1hyp	71.70641
yyry	2	f	0.708	35.03	1.118	0.211	hypoxia	1	0	0	Ucrit2hyp	
yyry	2	f	0.708	35.03	1.118	0.211	hypoxia	1	0	0	Ucrit3hyp	
yyry	2	f	0.708	35.03	1.118	0.211	normoxia	1	0	0	Ucrit1norm	77.03097
yyry	2	f	0.708	35.03	1.118	0.211	normoxia	1	0	0	Ucrit2norm	
yyry	2	f	0.708	35.03	1.118	0.211	normoxia	1	0	0	Ucrit3norm	
yyry	2	f	0.708	35.03	1.118	0.211	normoxia	1	0	0	Ucrit1hyp	71.70641
yyry	2	f	0.708	35.03	1.118	0.211	normoxia	1	0	0	Ucrit2hyp	
yyry	2	f	0.708	35.03	1.118	0.211	normoxia	1	0	0	Ucrit3hyp	
yyry	2	f	0.708	35.03	1.118	0.211	hypoxia	2	0	0	Ucrit1norm	77.03097
yyry	2	f	0.708	35.03	1.118	0.211	hypoxia	2	0	0	Ucrit2norm	
yyry	2	f	0.708	35.03	1.118	0.211	hypoxia	2	0	0	Ucrit3norm	
yyry	2	f	0.708	35.03	1.118	0.211	hypoxia	2	0	0	Ucrit1hyp	71.70641
yyry	2	f	0.708	35.03	1.118	0.211	hypoxia	2	0	0	Ucrit2hyp	
yyry	2	f	0.708	35.03	1.118	0.211	hypoxia	2	0	0	Ucrit3hyp	
yyry	2	f	0.708	35.03	1.118	0.211	normoxia	2	0	0	Ucrit1norm	77.03097
yyry	2	f	0.708	35.03	1.118	0.211	normoxia	2	0	0	Ucrit2norm	
yyry	2	f	0.708	35.03	1.118	0.211	normoxia	2	0	0	Ucrit3norm	
yyry	2	f	0.708	35.03	1.118	0.211	normoxia	2	0	0	Ucrit1hyp	71.70641
yyry	2	f	0.708	35.03	1.118	0.211	normoxia	2	0	0	Ucrit2hyp	
yyry	2	f	0.708	35.03	1.118	0.211	normoxia	2	0	0	Ucrit3hyp	
yyyp	4	m	0.573	32.17	0.769	0.099	normoxia	1	0	0	Ucrit1norm	84.86446
yyyp	4	m	0.573	32.17	0.769	0.099	normoxia	1	0	0	Ucrit2norm	88.27448
yyyp	4	m	0.573	32.17	0.769	0.099	normoxia	1	0	0	Ucrit3norm	81.29359
yyyp	4	m	0.573	32.17	0.769	0.099	normoxia	1	0	0	Ucrit1hyp	63.88962
yyyp	4	m	0.573	32.17	0.769	0.099	normoxia	1	0	0	Ucrit2hyp	72.63986
yyyp	4	m	0.573	32.17	0.769	0.099	normoxia	1	0	0	Ucrit3hyp	68.74729

yyyp	4	m	0.573	32.17	0.769	0.099	hypoxia	1	0	0	Ucrit1norm	84.86446
yyyp	4	m	0.573	32.17	0.769	0.099	hypoxia	1	0	0	Ucrit2norm	88.27448
yyyp	4	m	0.573	32.17	0.769	0.099	hypoxia	1	0	0	Ucrit3norm	81.29359
yyyp	4	m	0.573	32.17	0.769	0.099	hypoxia	1	0	0	Ucrit1hyp	63.88962
yyyp	4	m	0.573	32.17	0.769	0.099	hypoxia	1	0	0	Ucrit2hyp	72.63986
yyyp	4	m	0.573	32.17	0.769	0.099	hypoxia	1	0	0	Ucrit3hyp	68.74729
yyyp	4	m	0.573	32.17	0.769	0.099	normoxia	2	0	0	Ucrit1norm	84.86446
yyyp	4	m	0.573	32.17	0.769	0.099	normoxia	2	0	0	Ucrit2norm	88.27448
yyyp	4	m	0.573	32.17	0.769	0.099	normoxia	2	0	0	Ucrit3norm	81.29359
yyyp	4	m	0.573	32.17	0.769	0.099	normoxia	2	0	0	Ucrit1hyp	63.88962
yyyp	4	m	0.573	32.17	0.769	0.099	normoxia	2	0	0	Ucrit2hyp	72.63986
yyyp	4	m	0.573	32.17	0.769	0.099	normoxia	2	0	0	Ucrit3hyp	68.74729
yyyp	4	m	0.573	32.17	0.769	0.099	hypoxia	2	0	0	Ucrit1norm	84.86446
yyyp	4	m	0.573	32.17	0.769	0.099	hypoxia	2	0	0	Ucrit2norm	88.27448
yyyp	4	m	0.573	32.17	0.769	0.099	hypoxia	2	0	0	Ucrit3norm	81.29359
yyyp	4	m	0.573	32.17	0.769	0.099	hypoxia	2	0	0	Ucrit1hyp	63.88962
yyyp	4	m	0.573	32.17	0.769	0.099	hypoxia	2	0	0	Ucrit2hyp	72.63986
yyyp	4	m	0.573	32.17	0.769	0.099	hypoxia	2	0	0	Ucrit3hyp	68.74729
yyyy	3	f	0.724	35.06	1.093	0.185	hypoxia	1	0.698333333	1	Ucrit1norm	82.9169
yyyy	3	f	0.724	35.06	1.093	0.185	hypoxia	1	0.698333333	1	Ucrit2norm	
yyyy	3	f	0.724	35.06	1.093	0.185	hypoxia	1	0.698333333	1	Ucrit3norm	
yyyy	3	f	0.724	35.06	1.093	0.185	hypoxia	1	0.698333333	1	Ucrit1hyp	62.5821
yyyy	3	f	0.724	35.06	1.093	0.185	hypoxia	1	0.698333333	1	Ucrit2hyp	
yyyy	3	f	0.724	35.06	1.093	0.185	hypoxia	1	0.698333333	1	Ucrit3hyp	
yyyy	3	f	0.724	35.06	1.093	0.185	normoxia	1	0	0	Ucrit1norm	82.9169
yyyy	3	f	0.724	35.06	1.093	0.185	normoxia	1	0	0	Ucrit2norm	
yyyy	3	f	0.724	35.06	1.093	0.185	normoxia	1	0	0	Ucrit3norm	
yyyy	3	f	0.724	35.06	1.093	0.185	normoxia	1	0	0	Ucrit1hyp	62.5821
yyyy	3	f	0.724	35.06	1.093	0.185	normoxia	1	0	0	Ucrit2hyp	
yyyy	3	f	0.724	35.06	1.093	0.185	normoxia	1	0	0	Ucrit3hyp	
yyyy	3	f	0.724	35.06	1.093	0.185	hypoxia	2	0	0	Ucrit1norm	82.9169
yyyy	3	f	0.724	35.06	1.093	0.185	hypoxia	2	0	0	Ucrit2norm	
yyyy	3	f	0.724	35.06	1.093	0.185	hypoxia	2	0	0	Ucrit3norm	

yyyy	3	f	0.724	35.06	1.093	0.185	hypoxia	2	0	0	Ucrit1hyp	62.5821
yyyy	3	f	0.724	35.06	1.093	0.185	hypoxia	2	0	0	Ucrit2hyp	
yyyy	3	f	0.724	35.06	1.093	0.185	hypoxia	2	0	0	Ucrit3hyp	
yyyy	3	f	0.724	35.06	1.093	0.185	normoxia	2	0	0	Ucrit1norm	82.9169
yyyy	3	f	0.724	35.06	1.093	0.185	normoxia	2	0	0	Ucrit2norm	
yyyy	3	f	0.724	35.06	1.093	0.185	normoxia	2	0	0	Ucrit3norm	
yyyy	3	f	0.724	35.06	1.093	0.185	normoxia	2	0	0	Ucrit1hyp	62.5821
yyyy	3	f	0.724	35.06	1.093	0.185	normoxia	2	0	0	Ucrit2hyp	
yyyy	3	f	0.724	35.06	1.093	0.185	normoxia	2	0	0	Ucrit3hyp	
