# Supplementary material for: Hypoxia alters vulnerability to capture and the potential for trait-based selection in a scaled-down trawl fishery
Source: Conserv Physiol. 2019 Nov 27;7(1):coz082. doi: 10.1093/conphys/coz082 (PMC6880855; doi:10.1093/conphys/coz082)
Supplement: Supplementary_materials_3of3_coz082 [file supplementary_materials_3of3_coz082.docx]

**Table S1. Full statistical results for models.** LMM refers to Linear Mixed Model, GLMM bin. to Generalized Linear Mixed Model with a binomial error distribution, and GLMM β to a Generalized Linear Mixed Model with a beta error distribution. Unless otherwise stated, the 1st Step of a model includes all two-way interactions, further steps are indicative of more parsimonious models with parameters removed. Full colons denote interaction terms. For the first two models Fish ID is included as a random effect, whilst it was removed from the last model based on LRT testing. Effects with P < 0.05 are indicated in bold font.

| **Response Variable** | **Test** | **Random effect** | **step** | **Fixed effect** | **statistic** | **p-value** |
| --- | --- | --- | --- | --- | --- | --- |
| *U_crit_* | LMM | Fish ID | 1st | Oxygen availability - normoxia  SMR  MMR  Mass  Sex - male  Oxygen availability:SMR  Oxygen availability:MMR  Oxygen availability:Mass  Oxygen availability:Sex | 13.447  -1.934  3.123  -2.922 0.972  0.922  0.657  0.765  1.885 | **0.0000**  0.0555  **0.0022**  **0.0042**  0.3326  0.3576  0.5115  0.4449  0.0608 |
| *U_crit_* | LMM | Fish ID | 2nd | Oxygen availability - normoxia  SMR  MMR  Mass  Sex - male  Oxygen availability:SMR  Oxygen availability:Mass  Oxygen availability:Sex | 13.536  -2.175  4.005  -3.042  0.907  1.441  0.961  2.082 | **0.0000**  **0.0316**  **0.0001**  **0.0029**  0.3659  0.1511  0.3373  **0.0385** |
| *U_crit_* | LMM | Fish ID | 3rd | Oxygen availability - normoxia  SMR  MMR  Mass  Sex - male  Oxygen availability:SMR  Oxygen availability:Sex | 14.464  -2.286  4.013  -2.946  1.117  1.702  1.850 | **0.0000**  **0.0240**  **0.0001**  **0.0039**  0.2660  0.0902  0.0657 |
| *U_crit_* | LMM | Fish ID | 4th | Oxygen availability - normoxia  SMR  MMR  Mass  Sex - male  Oxygen availability:Sex | 15.490  -1.716  4.023  -2.950  1.449  1.267 | **0.0000**  0.0888  **0.0001**  **0.0038**  0.1498  0.2064 |
| *U_crit_* | LMM | Fish ID | 5th | Oxygen availability - normoxia  SMR  MMR  Mass  Sex - male  Oxygen availability:Sex | 21.990  -1.718  4.031  -2.957  2.197 | **0.0000**  0.0883  0.0001  0.0037  0.0299 |
| *U_crit_* | LMM | Fish ID | 6^th^ | Oxygen availability - normoxia  MMR  Mass  Sex - male | 22.014  3.607  -3.122  2.906 | **0.0000**  **0.0005**  **0.0022**  **0.0044** |
|  |  |  |  |  |  |  |
| Capture | GLMM bin. | Fish ID | 1^st^ | Oxygen availability - normoxia  SMR  MMR  Mass  *U_crit_*  Sex - male  Oxygen availability:SMR  Oxygen availability:MMR  Oxygen availability:Mass  Oxygen availability:*U_crit_*  Oxygen availability:Sex | -4.540  -1.051  1.345  -1.179  -2.594  -0.097  -1.131  1.002  -0.058  -0.535  -0.691 | **0.0000**  0.2933  0.1785  0.2382  **0.0094**  0.9229  0.2581  0.3165  0.9540  0.5924  0.4895 |
| Capture | GLMM bin. | Fish ID | 2^nd^ | Oxygen availability  SMR  MMR  Mass  *U_crit_*  Sex  Oxygen availability:SMR  Oxygen availability:MMR  Oxygen availability:*U_crit_*  Oxygen availability:Sex | -4.639  -1.050  1.357  -1.277  -2.601  -0.105  -1.131  1.019  -0.533  -0.730 | **0.0000**  0.2938  0.1748  0.2017  **0.0092**  0.9165  0.2579  0.3082  0.5942  0.4655 |
| Capture | GLMM bin. | Fish ID | 3^rd^ | Oxygen availability - normoxia  SMR  MMR  Mass  *U_crit_*  Sex - male  Oxygen availability:SMR  Oxygen availability:MMR  Oxygen availability:Sex | -5.011  -1.075  1.422  -1.285  -3.010  -0.052  -1.050  0.915  -0.897 | **0.0000**  0.2822  0.1549  0.1989  **0.0026**  0.9582  0.2939  0.3603  0.3697 |
| Capture | GLMM bin. | Fish ID | 4^th^ | Oxygen availability - normoxia  SMR  MMR  Mass  *U_crit_*  Sex - normoxia  Oxygen availability:SMR  Oxygen availability:MMR | -6.734  -1.218  1.492  -1.272  -3.005  -0.354  -0.711  0.686 | **0.0000**  0.2230  0.1356  0.2032  **0.0026**  0.7230  0.4771  0.4927 |
| Capture | GLMM bin. | Fish ID | 5^th^ | Oxygen availability  SMR  MMR  Mass  *U_crit_*  Sex  Oxygen availability:SMR | -6.801  -1.315  1.790  -1.282  -3.001  -0.348  -0.427 | **0.0000**  0.1884  0.0735  0.1998  **0.0026**  0.7276  0.6690 |
| Capture | GLMM bin. | Fish ID | 6^th^ | Oxygen availability - normoxia  SMR  MMR  Mass  *U_crit_*  Sex - normoxia | -6.855  -1.512  1.781  -1.266  -2.996  -0.342 | **0.0000**  0.1304  0.0749  0.2056  **0.0027**  0.7323 |
| Capture | GLMM bin. | Fish ID | 7^th^ | Oxygen availability - normoxia  SMR  MMR  Mass  *U_crit_* | -6.854  -1.484  1.754  -1.230  -3.140 | **0.0000**  0.1376  0.0793  0.2187  **0.0016** |
| Capture | GLMM bin. | Fish ID | 8^th^ | Oxygen availability - normoxia  MMR  Mass  *U_crit_* | -6.854  1.238  -1.529  -2.912 | **0.0000**  0.2158  0.1262  **0.0035** |
| Capture | GLMM bin. | Fish ID | 9^th^ | Oxygen availability - normoxia  Mass  *U_crit_* | -6.855  -1.194  -2.674 | **0.0000**  0.2324  **0.0075** |
| Capture | GLMM bin. | Fish ID | 9^th^ | Oxygen availability - normoxia  *U_crit_* | -6.851  -2.465 | **0.0000**  **0.0137** |
|  |  |  |  |  |  |  |
| T*_net_* | GLMM β | - | 1st | Oxygen availability - normoxia  SMR  MMR  Mass  *U_crit_*  Sex - male  Oxygen availability:SMR  Oxygen availability:MMR  Oxygen availability:Mass  Oxygen availability:*U_crit_*  Oxygen availability:Sex | 0.549  -1.709  0.064  -2.286  1.478  -2.192  1.993  -1.306  -0.976  0.704  0.004 | 0.5828  0.0874  0.9488  **0.0222**  0.1395  **0.0284**  **0.0463**  0.1914  0.3289  0.4814  0.9967 |
| T*_net_* | GLMM β | - | 2^nd^ | Oxygen availability - normoxia  SMR  MMR  Mass  *U_crit_*  Sex - male  Oxygen availability:SMR  Oxygen availability:MMR  Oxygen availability:Mass  Oxygen availability:*U_crit_* | 1.076  -1.716  0.064  -2.299  1.479  -2.241  2.156  -1.571  -1.644  0.706 | 0.2818  0.0862 .  0.9489  **0.0215**  0.1392  **0.0251**  **0.0311**  0.1162  0.1003  0.4803 |
| T*_net_* | GLMM β | - | 2^nd^ | Oxygen availability - normoxia  SMR  MMR  Mass  *U_crit_*  Sex - male  Oxygen availability:SMR  Oxygen availability:MMR  Oxygen availability:Mass | 0.913  -1.680  0.001  -2.266  1.823  -2.281  2.063  -1.459  -1.729 | 0.3612  0.0930  0.9992  **0.0234**  0.0683  **0.0226**  **0.0391**  0.1445  0.0839 |
| T*_net_* | GLMM β | - | 3^rd^ | Oxygen availability - normoxia  SMR  MMR  Mass  *U_crit_*  Sex - male  Oxygen availability:SMR  Oxygen availability:Mass | 0.194  -1.461  -0.290  -2.091  1.684  -1.969  1.794  -0.991 | 0.8465  0.1440  0.7718  **0.0365**  0.0921  **0.0489**  0.0727  0.3217 |
| T*_net_* | GLMM β | - | 4^th^ | Oxygen availability - normoxia  SMR  MMR  Mass  *U_crit_*  Sex - male  Oxygen availability:SMR | 0.002  -1.473  -0.148  -2.431  1.695  -1.951  1.569 | 0.9987  0.1407  0.8826  **0.0151**  0.0901  0.0511  0.1166 |
| T*_net_* | GLMM β | - | 5^th^ | Oxygen availability - normoxia  SMR  MMR  Mass  *U_crit_*  Sex - male | -0.067  -1.058  -0.059  -2.316  1.504  -1.875 | 0.9469  0.2899  0.9527  **0.0205**  0.1327  0.0607 |
| T*_net_* | GLMM β | - | 6^th^ | Oxygen availability - normoxia  MMR  Mass  *U_crit_*  Sex - male | 0.027  -0.845  -2.193  1.709  -1.565 | 0.9788  0.3981  **0.0283**  0.0875  0.1176 |
| T*_net_* | GLMM β | - | 7^th^ | MMR  Mass  *U_crit_*  Sex - male | -0.846  -2.193  1.713  -1.566 | 0.3975  **0.0283**  0.0866  0.1173 |
| T*_net_* | GLMM β | - | 8^th^ | MMR  Mass  Sex - male | -0.494  -2.291  -1.108 | 0.6212  **0.0219**  0.2677 |
| T*_net_* | GLMM β | - | 9^th^ | Mass  Sex - male | -2.370  -1.179 | **0.0178**  0.2385 |
| T*_net_* | GLMM β | - | 10^th^ | Mass | -2.055 | **0.0399** |
